# The PEG-responding desiccome of the alder microsymbiont *Frankia alni*

**DOI:** 10.1038/s41598-017-18839-0

**Published:** 2018-01-15

**Authors:** Kais Ghedira, Emna Harigua-Souiai, Cherif Ben Hamda, Pascale Fournier, Petar Pujic, Sihem Guesmi, Ikram Guizani, Guylaine Miotello, Jean Armengaud, Philippe Normand, Haïtham Sghaier

**Affiliations:** 1 Laboratory of Bioinformatics, Biomathematics and Biostatistics - LR16IPT09, Institut Pasteur de Tunis, Université de Tunis el Manar, Tunis, 1002 Tunisia; 2 Laboratory of Molecular Epidemiology and Experimental Pathology - LR11IPT04, Institut Pasteur de Tunis, Université de Tunis el Manar, Tunis, 1002 Tunisia; 3 Université de Carthage, Faculté des Sciences de Bizerte, Tunis, 7021 Tunisia; 4 Université de Lyon, Université Lyon 1, Lyon; CNRS, UMR 5557, Ecologie Microbienne, UMR1418, INRA, 69622 Cedex Villeurbanne, France; 5 Laboratory “Energy and Matter for Development of Nuclear Sciences” (LR16CNSTN02), National Center for Nuclear Sciences and Technology (CNSTN), Sidi Thabet Technopark, 2020 Tunisia; 6 National Agronomy Institute (INAT), Avenue Charles Nicolle, 1082 Tunis, Mahrajène Tunisia; 7 Laboratoire Innovations Technologiques pour la Détection et le Diagnostic (Li2D), Service de Pharmacologie et Immunoanalyse (SPI), CEA, INRA, F-30207 Bagnols sur Cèze, France; 8 Associated with Laboratory “Biotechnology and Nuclear Technology” (LR16CNSTN01) & Laboratory “Biotechnology and Bio-Geo Resources Valorization” (LR11ES31), Sidi Thabet Technopark, 2020 Tunisia

## Abstract

Actinorhizal plants are ecologically and economically important. Symbiosis with nitrogen-fixing bacteria allows these woody dicotyledonous plants to colonise soils under nitrogen deficiency, water-stress or other extreme conditions. However, proteins involved in xerotolerance of symbiotic microorganisms have yet to be identified. Here we characterise the polyethylene glycol (PEG)-responding desiccome from the most geographically widespread Gram-positive nitrogen-fixing plant symbiont, *Frankia alni*, by next-generation proteomics, taking advantage of a Q-Exactive HF tandem mass spectrometer equipped with an ultra-high-field Orbitrap analyser. A total of 2,052 proteins were detected and quantified. Under osmotic stress, PEG-grown *F. alni* cells increased the abundance of envelope-associated proteins like ABC transporters, mechano-sensitive ion channels and Clustered Regularly Interspaced Short Palindromic Repeats CRISPR-associated (*cas*) components. Conjointly, dispensable pathways, like nitrogen fixation, aerobic respiration and homologous recombination, were markedly down-regulated. Molecular modelling and docking simulations suggested that the PEG is acting on *Frankia* partly by filling the inner part of an up-regulated osmotic-stress large conductance mechanosensitive channel.

## Introduction

Actinobacteria belonging to the genus *Frankia* do establish nitrogen-fixing nodular symbiosis with the roots of 23 angiosperm genera that are collectively called “actinorhizals”^1^. These plants form root nodules in which *Frankia* fixes nitrogen, thus permitting them to thrive in pioneer soils poor in nitrogen and organic matter, such as glacial moraines, lava fields, forest burnouts or anthropogenic sites such as mine spoils or hydrodam dykes^2^. *Frankia* establishes a symbiotic association with the roots of several dicotyledonous plants. The different *Frankia* lineages form a coherent cluster at the root of the aerobic actinobacteria phylum^3^, and *F. alni* in particular establishes symbiosis with alder (*Alnus*) and bayberry (*Morella*) species^4^.

The interaction has evolved over several million years with a sophisticated dialogue that does not imply acylated N-acetyl-glucosamine oligomeric Nod factors^5^. The *Frankia* determinants of symbiosis are still poorly known, which is for the most part due to the lack of a genetic transformation system. Transcriptomics has shown genes coding for nitrogenase (*nif*), hydrogenase uptake (*hup*), hopanoids (*shc, hpn*), iron-sulfur (*suf*) clusters as among the most up-regulated^6^. Proteomics has also been used to analyze the symbiosis^7^ to identify up-regulated peptides and it has shown the presence of Nif, Hup, Suf, Hop proteins as expected but also several transporters, regulators and various proteins involved in stress responses.

Osmotic stress is a constant challenge for bacteria living in a range of soils^8,9^, from those having low salinity in rainy cold latitudes to highly saline ones in warm and dry parts of the world. It also affects symbionts^10^ that must alternate between two main biotopes, the soil and the root tissues. Soils, especially poor soils, have low osmotic potential while plant tissues have a much higher osmotic potential. Most studies on the effect of salinity on actinorhizal symbionts have focused on the plant, especially *Casuarina* to illustrate various adaptations such as sodium partitioning or proline accumulation^11^. Only a few recent studies on *Frankia* have shown the effects of salinity on ammonium (NH_4_^+^) assimilation^12^, on cell wall/membrane biogenesis functions and on some transport proteins^13^. *Alnus-*, *Casuarina*- and *Elaeagnus*- infective isolates grow best at 50 mM but are nevertheless able to grow well in medium containing up to 200 mM NaCl, but not at 500 mM^14^. The effect of NaCl on one strain, CcI6, was apparently less severe than that of another osmolyte, sucrose^15^. Nevertheless, little is known about the molecular adaptations and the present study was undertaken to better understand how *Frankia* coped with osmolytes using proteogenomics, an approach recently used to better decipher how *Geodermatophilaceae* coped with desiccation^16^.

NaCl causes various impairments to cells, some related to osmotic stress and some related to ionic stress and these two effects are clearly distinguishable^17^. PEG is a non-ionic polymer of various molecular weights (MW) that has been used often for osmotic studies when it is sought to have only the osmotic effect.

Mechanisms involved in desiccation tolerance have been well studied in Cyanobacteria^18^, Actinobacteria^19,20^ as well as xerotolerant members of the *Bosea*, *Chelatococcus*, *Deinococcus* and *Methylobacterium* genera^21^. Briefly, the different studies have shown that desiccation damages cell membranes, proteins and DNA due to oxidative stress and production of reactive oxygen species (ROS)^20,21^. Strategies adopted by these microorganisms for desiccation tolerance include prevention of ROS damage, osmoprotection through the accumulation of sucrose and/or trehalose^10,22^, the uptake of exogenous glycine betaine^23^, the production of extracellular polysaccharides (EPS) that reduce water loss^24^, or the ability to limit protein oxidation during dehydration^21^. Previous studies^8,20,21,25–27^ highlighted changes in expression of several genes following water stress including genes for synthesis of trehalose/sucrose, sugar transporters, chaperone genes (*groES*/*EL*, *dnaK*/*J*), oxidative stress protection genes (*dps*, thioredoxin), ABC transporters, dehydrogenases, esterases, proteases, hydrolases and lyases. A *Casuarina*-infective *Frankia* strain was recently shown to have few proteins upregulated under salt stress, among which cell wall/membrane biogenesis functions and some transport proteins^13^.

The desiccome, a term coined by Potts and colleagues^28^, can be defined as the set of genes, proteins and metabolites that are necessary for desiccation tolerance^29^. Here we report the first PEG-responding desiccome of a nitrogen-fixing symbiotic bacterium, *F. alni*. We highlight the role of ABC transporters, mechanosensitive ion channels and Cas components in response to this stress.

## Results

### Growth in polyethylene glycol (PEG)

Upon inoculation in BAP- medium at a 0.1 OD_600_, growth of *F. alni* strain ACN14a was inhibited at all concentrations of PEG tested. Nitrogen fixation was active without PEG but even the lowest concentration of PEG completely inhibited it (data not shown). We chose an inoculation density of 0.1 to have sufficient biomass and after 7 days, growth without PEG had reached an OD_600_ of 0.677, *i.e*. a 0.74 fold in density increase. In the presence of PEG 0.9%, OD_600_ reached 0.42 or a 0.47 fold increase. Growth in the presence of PEG was thus 63% that without PEG. Hyphae, vesicles and sporangia looked similar under both conditions.

### Comprehensive proteome coverage of *F. alni* cells

Thanks to the high speed and high resolution of this analyser, a total of 295,788 high quality MS/MS spectra were recorded. Amongst these, a set of 208,018 MS/MS spectra (70.3%) was assigned to peptide sequences. This dataset allowed detecting a total of 20,825 peptide sequences, which pointed at 2,454 polypeptides, with 2,052 proteins certified with at least two distinct peptide sequences. These proteins and their characteristics are listed in Supplementary Data, Table S1. On the average, a total of 20,801 spectral counts were measured per sample with a remarkably low standard deviation (5.3%). With an average of 10 peptides and 100 spectral counts per polypeptide, an extensive coverage of the proteome was reached that compares favourably to most proteomic studies of bacteria^16,30,31^.

### Differentially expressed proteins

**Figure 1:**
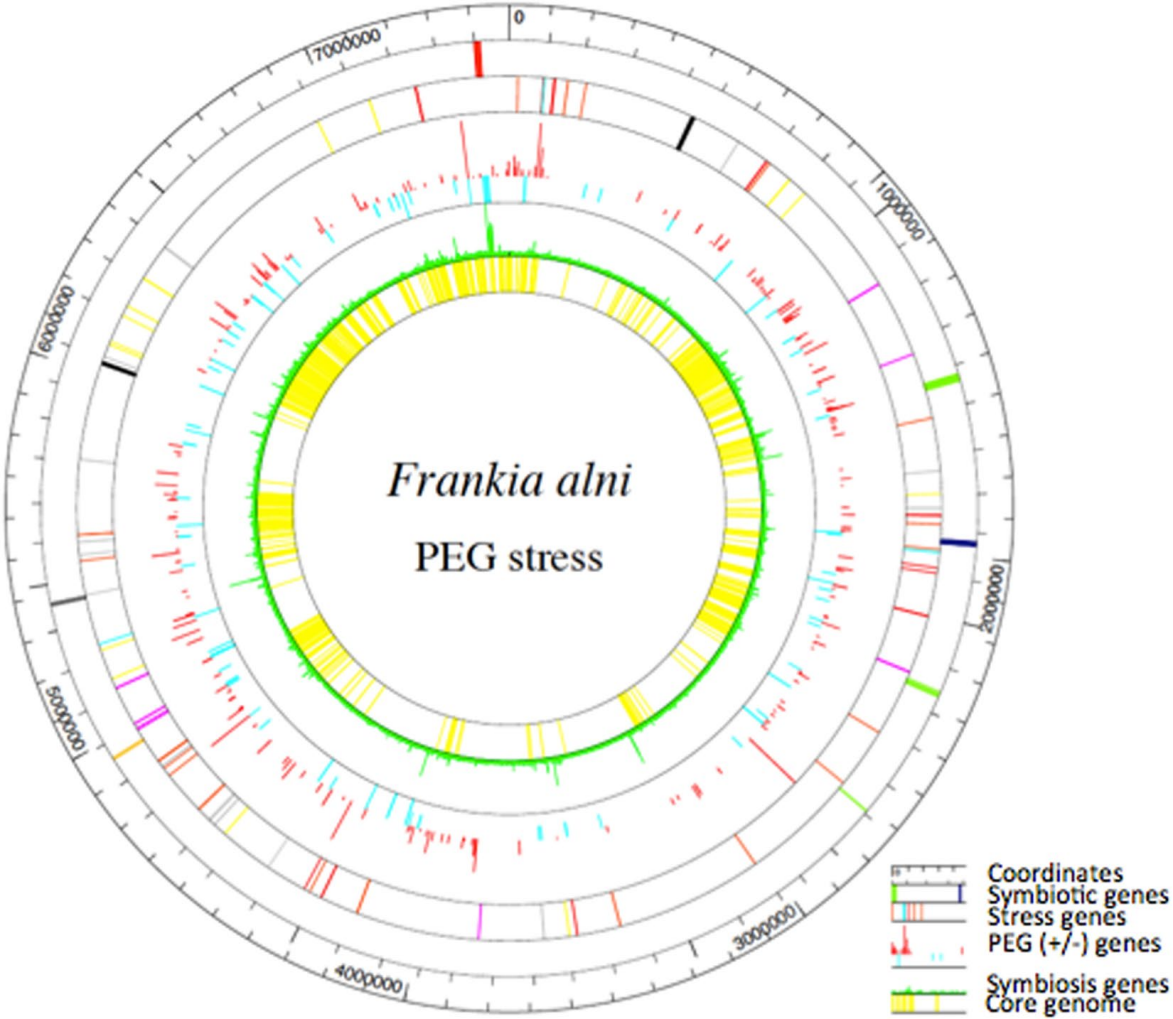
*F. alni* proteome expression following polyethylene glycol (PEG) treatment. From the outside in are 1-the coordinates in bp; 2-the coordinates in 100 genes; 3-the symbiotic genes (in green the hopanoid genes, in blue the uptake hydrogenase genes, in yellow the iron-sulfur genes, in gray the cellulase genes, in red the nitrogenase genes); 4-selected genes that play a role in stress response with oxidative stress genes (orange), heat-resistance genes (red), osmotic-resistance genes (cyan), UV-resistance (violet), metals-resistance (grey), Clustered Regularly Interspaced Short Palindromic Repeats (CRISPR) genes (black), poorly characterised or universal stress genes (yellow); 5-Genes coding for proteins more abundant upon PEG-induction (red), or more abundant in the control (cyan); 6-Symbiosis up-regulated genes (in green) from Alloisio *et al*.^6^; 7-Core genome at a level of 70% of conserved amino acids over 80% of the length of the shortest sequence in selected symbiotic *Frankia* strains of cluster1a (ACN14a, CpI1, QA3), cluster1c (CcI3, CcI6), cluster2 (Dg1, BMG5.1) and cluster3 (EAN1pec, BMG5.12, BCU110501), in yellow.

When comparing both conditions and compiling the five biological replicates of each condition, a total of 1,951 proteins were detected whatever the conditions. A set of 29 proteins was specifically detected only in the reference while 85 were detected only in the PEG-treated cells. Differentially detected protein abundances were identified using a Fold Change (FC) threshold of 1.5 and *p*-value below 0.05. Four classes were delineated as follows: i) Blue class proteins for which identifications satisfied both, the fold change (>1.5) and statistical criteria (*p*-value < 0.05); ii) Orange class proteins for which identifications did not meet the fold criterion but have low *p*-values; iii) Green class proteins for which identifications satisfied the fold criterion but not the statistical criterion; iv) and finally Red class for which identifications did not meet the fold and *p*-value criteria. The Blue class comprised 294 proteins. A total of 211 proteins were more abundant in PEG-treated cells compared to the reference while 83 were found less abundant. Figure 1 shows the distribution of the proteins affected by the PEG treatment compared to the control within the *F. alni* genome and their expression levels. Genes coding for more abundant proteins upon PEG-induction are highlighted in red whereas those more abundant in the control were highlighted in cyan in Fig. 1. Tables 1 and 2 list the most up- and down-regulated PEG-modulated proteins of *F. alni*, respectively. Twelve ribosome-associated translation proteins (FRAAL1099–50S ribosomal subunit protein L30 (2.10), *etc*.) were also up-regulated by PEG but are not shown in Table 1.

**Table 1:** Summary of the most up-regulated polyethylene glycol (PEG)-induced proteins of *F. alni*.

Response mechanism	Protein	Product	Tfold
Membrane transport proteins	FRAAL2567	Putative autotransporter adhesion	5.67
	FRAAL2549	Secondary metabolite secretion ATP-binding transport	4.80
	FRAAL1420	Nitrate/sulfonate ATP-binding (ABC) transporter	2.89
	FRAAL2085	Trk system potassium uptake protein TrkA	1.65
Intracellular osmolytes concentration	FRAAL0095	Osmotic-stress mechanosensitive channel MscL	4.30
	FRAAL1888	Glutamine amidotransferase	2.20
	FRAAL1197	2-deoxyribose-5-phosphate aldolase, NAD(P)-linked	2.15
	FRAAL5155	Peptidase S51, dipeptidase E	1.91
	FRAAL2547	Non-ribosomal peptide synthase	2.20
	FRAAL2542	Non-ribosomal peptide synthase	1.80
	FRAAL2545	Non-ribosomal peptide synthase	1.60
	FRAAL2500	Polyketidecyclase	1.79
Molecular chaperones	FRAAL4431	Clp-family ATP-binding protease	4.00
Membrane lipid biosynthesis	FRAAL4781	Acyl-coenzyme A thioesterase	3.00
	FRAAL6258	Acyl carrier protein (ACP)	2.33
	FRAAL1744	Phospholipase D	1.82
	FRAAL3585	Esterase	1.75
	FRAAL5087	1-acylglycerol-3-phosphate O-acyltransferase	1.50
	FRAAL2171	Acyl-CoA N-acyltransferase	1.50
CO_2_ sensing and metabolism	FRAAL1222	Carbonic anhydrase	2.80
Detoxification of Reactive-Oxygen Species (ROS) and DNA repair	FRAAL6022	Gamma-aminobutyraldehyde dehydrogenase	2.17
	FRAAL1417	Methylmalonate-semialdehyde dehydrogenase (acylating)	1.50
	FRAAL5703	Global regulator (repressor) for SOS regulon LexA	1.56

**Table 2:** Summary of the most down-regulated polyethylene glycol (PEG)-modulated proteins of *F. alni*.

Response mechanism	Protein	Product	Tfold
Nitrogen fixation	FRAAL6814	Nitrogenase-associated homocitrate synthase NifV	−6.50
	FRAAL6803	FeMo cofactor biosynthesis protein NifB	−5.24
	FRAAL6810	Nitrogenase iron-molybdenum cofactor biosynthesis NifE	−3.80
	FRAAL6809	Nitrogenase iron-molybdenum cofactor biosynthesis NifN	−3.50
	FRAAL2491	squalene-hopenecyclase Shc2	−2.17
	FRAAL6797	Ferredoxin	−2.00
	FRAAL6799	2-oxoglutarate ferredoxinoxidoreductase beta-subunit KorA	−1.74
	FRAAL1432	Squalene-hopenecyclase Shc1	−1.62
	FRAAL4563	Transport protein associated with Fe-S cluster assembly SufB	−1.60
Respiration and energy production and conversion	FRAAL4147	Cytochrome c oxidase polypeptide I (AA3 subunit 1) CtaD	−3.70
	FRAAL5625	Poly(3-hydroxybutyrate) depolymerasePha	−2.83
	FRAAL6005	Biotin carboxyl carrier protein	−2.00
	FRAAL6082	Succinate-semialdehyde dehydrogenase I, NADP-dependent	−2.00
	FRAAL5481	Malate synthase G AceB	−1.75
	FRAAL0315	Aldehyde dehydrogenase	−1.71
	FRAAL1829	[NiFe] uptake hydrogenase, large subunit HupL	−1.70
	FRAAL1039	NADH-quinoneoxidoreductase chain H NuoH	−1.50
DNA repair system	FRAAL6528	ATP-dependent protease, DNA repair protein	−2.36
	FRAAL5811	ATP-dependent DNA helicase RecG	−1.50

### Re-annotation of differentially expressed proteins following polyethylene glycol (PEG) treatment

Among the blue class differentially expressed proteins (294 proteins) identified under PEG treatment, 53 were characterised as hypothetical proteins and/or conserved hypothetical proteins. We therefore conducted a new analysis using PSI-BLAST tool to reannotate them. Seventeen ORFs remained as proteins with unknown function because of their low similarity to characterised proteins and the function of 36 ORFs could be deduced from closely-related known proteins (Supplementary Data, Table S2. Re-annotation of differentially expressed proteins following polyethylene glycol (PEG) treatment based on a Psi-Blast approach.). Among re-annotated ORFs, several envelope-associated proteins were identified including the most up-regulated protein (FRAAL2567 (5.67)), a putative auto-transporter adhesin. These results consolidate previous data (see Table 1) that have evidenced the extreme importance of envelope-associated proteins in response to water stress.

### Functional analysis

**Figure 2:**
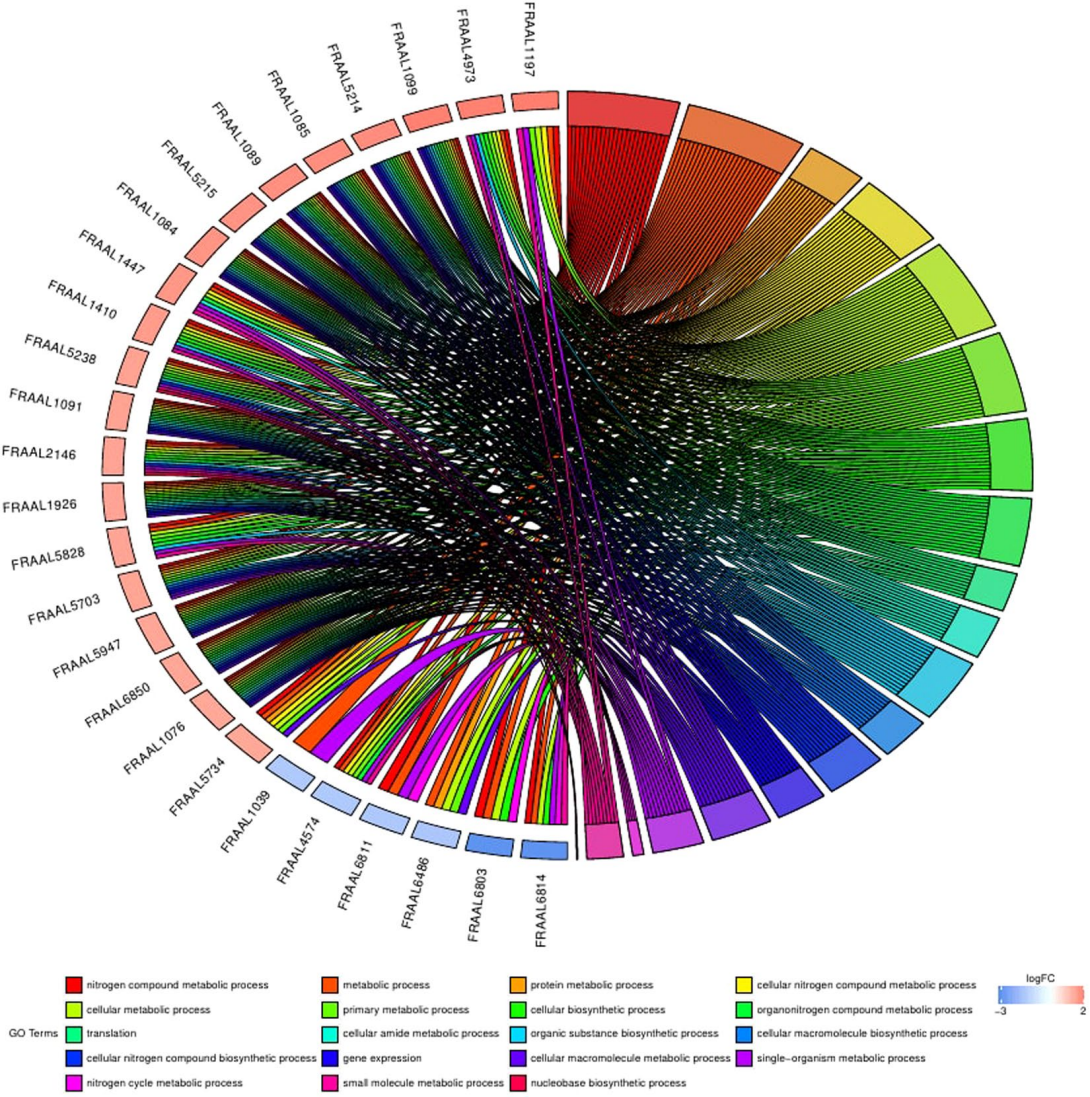
Chord diagram showing the most enriched biological processes (GO terms) with their differentially expressed blue class proteins following PEG treatment. In each chord, enriched GO biological processes are shown on the right, and *F. alni* blue class differentially expressed proteins contributing to this enrichment are shown on the left. On the left side of the circle, each differentially expressed protein (locus tag (FRAAL)) is represented by a rectangle which colour is correlated to the value of the Tfold (logFC). PEG up-regulated proteins are displayed in red whereas down-regulated proteins are displayed in blue. Chords connect protein names with biological process GO term groups. Each GO term is represented by one coloured line.

The functional enrichment analysis applied on the lists of differentially expressed proteins following PEG treatment, covering Gene Ontology and pathway annotations, was performed through the STRING database^32^. Among the enriched KEGG pathways within the blue class differentially expressed proteins, we denote the general metabolic pathways (map01100, FDR = 0.000147), ribosome (map03010, FDR = 0.000147) and sesquiterpenoid and triterpenoid biosynthesis (map00909, FDR = 0.0032). Among the most significantly enriched biological processes associated to the blue class of differentially expressed proteins, we found: nitrogen compound metabolic process (GO.0006807, FDR = 7.79e^−05^), metabolic process (GO.0008152, FDR = 0.00022), protein metabolic process (GO.0019538, FDR = 0.00022), cellular nitrogen compound metabolic process (GO.0034641, FDR = 0.00022), cellular metabolic process (GO.0044237, FDR = 0.00022) and translation (GO.0006412, FDR = 0.000706). Outputs of biological processes enrichment data were analysed with GOPlot^33^ to generate insightful plots. Figure 2 and Supplementary Figures 1 and 2 highlight the most enriched biological processes associated to the blue differentially expressed, orange and red class proteins, respectively. There are no significantly enriched biological processes in the green class proteins.

Among the top blue class enriched biological processes (Fig. 2), we denote the nitrogen compound metabolic process, which includes proteins belonging to Nif family (*e.g*., FRAAL6811/NifK), Rpm family (*e.g*., FRAAL1926/RpmA), Rpl family (*e.g*., FRAAL5214/RplT) and other proteins like FRAAL1410/MtnD, FRAAL5703/LexA, FRAAL5734/RimO, FRAAL1085/RpsS, FRAAL1076/RpsL, FRAAL1447/UreC, FRAAL5238/PyrB, FRAAL5828/LeuD, FRAAL4973/HisL, FRAAL2146/Apt, FRAAL4574/Tal and FRAAL1197/DeoC.

### *F. alni* protein-protein interactions

Supplementary Figures 3, 4, 5 and 6 represent *F. alni* protein-protein interactions within the blue, orange, green and red class proteins, respectively. Green nodes colour in Supplementary Figure 3 denotes up-regulated proteins whereas red nodes colour denotes down-regulated proteins. For instance, the network corresponding to the blue class proteins (Supplementary Figure 3) highlights an interaction between an up-regulated putative Clp-family ATP-binding protease (FRAAL4431) and a down-regulated putative signal peptide (FRAAL4430).

### Cluster of Orthologous Genes (COGs)

**Figure 3:**
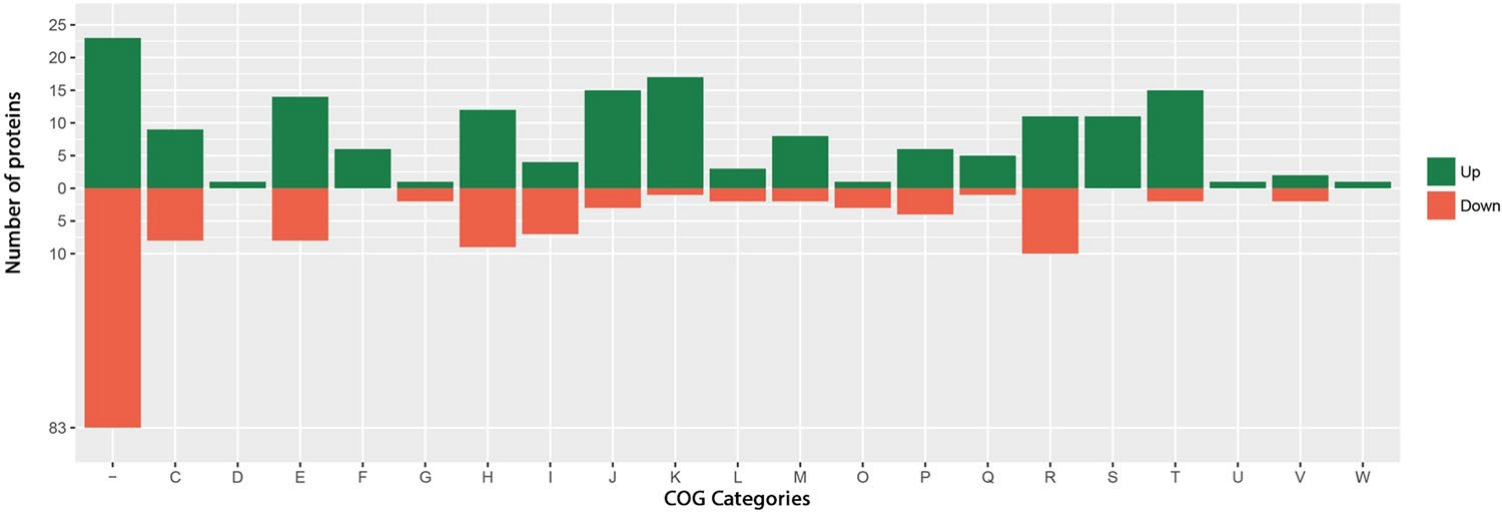
Predicted COG classes of up- and down-regulated *F. alni* blue class proteins. Up- and down-regulated COG classes in green and red colours, respectively. A: RNA processing and modification, B: Chromatin structure and dynamics, C: Energy production and conversion, D: Cell cycle control and mitosis, E: Amino Acid metabolism and transport, F: Nucleotide metabolism and transport, G: Carbohydrate metabolism and transport, H: Coenzyme metabolism, I: Lipid metabolism, J: Translation, K: Transcription, L: Replication and repair, M: Cell wall/membrane/envelope biogenesis, N: Cell motility, O: Post-translational modification, protein turnover, chaperone functions, P: Inorganic ion transport and metabolism, Q: Secondary Structure, R: General Functional Prediction only, S: Function Unknown, T: Signal Transduction, U: Intracellular trafficking and secretion, V: Defence mechanisms, W: Extracellular structures, Y: Nuclear structure, Z: Cytoskeleton, (-): No COG assigned.

Figure 3 and Supplementary Figures 7, 8 and 9 show the distribution of the blue, orange, green and red classes proteins into the COG functional categories, respectively. Green colour within the Fig. 3 denotes up-regulated proteins whereas red colour denotes down-regulated proteins. We were not able to assign several hypothetical proteins to any COG category. For the blue class (Fig. 3), five COG categories/classes contain only up-regulated proteins—D (cell cycle control, cell division, chromosome partitioning), F (nucleotide transport and metabolism), S (function unknown), U (intracellular trafficking, secretion and vesicular transport) and W (extracellular structures). These results provide parallel pieces of evidence (see also Table 1) in support of the importance of protein clusters related to cellular processes and signalling and metabolism as well as some poorly characterised proteins during growth of *F. alni* under water stress.

### Comparison between differentially expressed proteins following polyethylene glycol (PEG)-induced water stress and symbiosis

**Figure 4:**
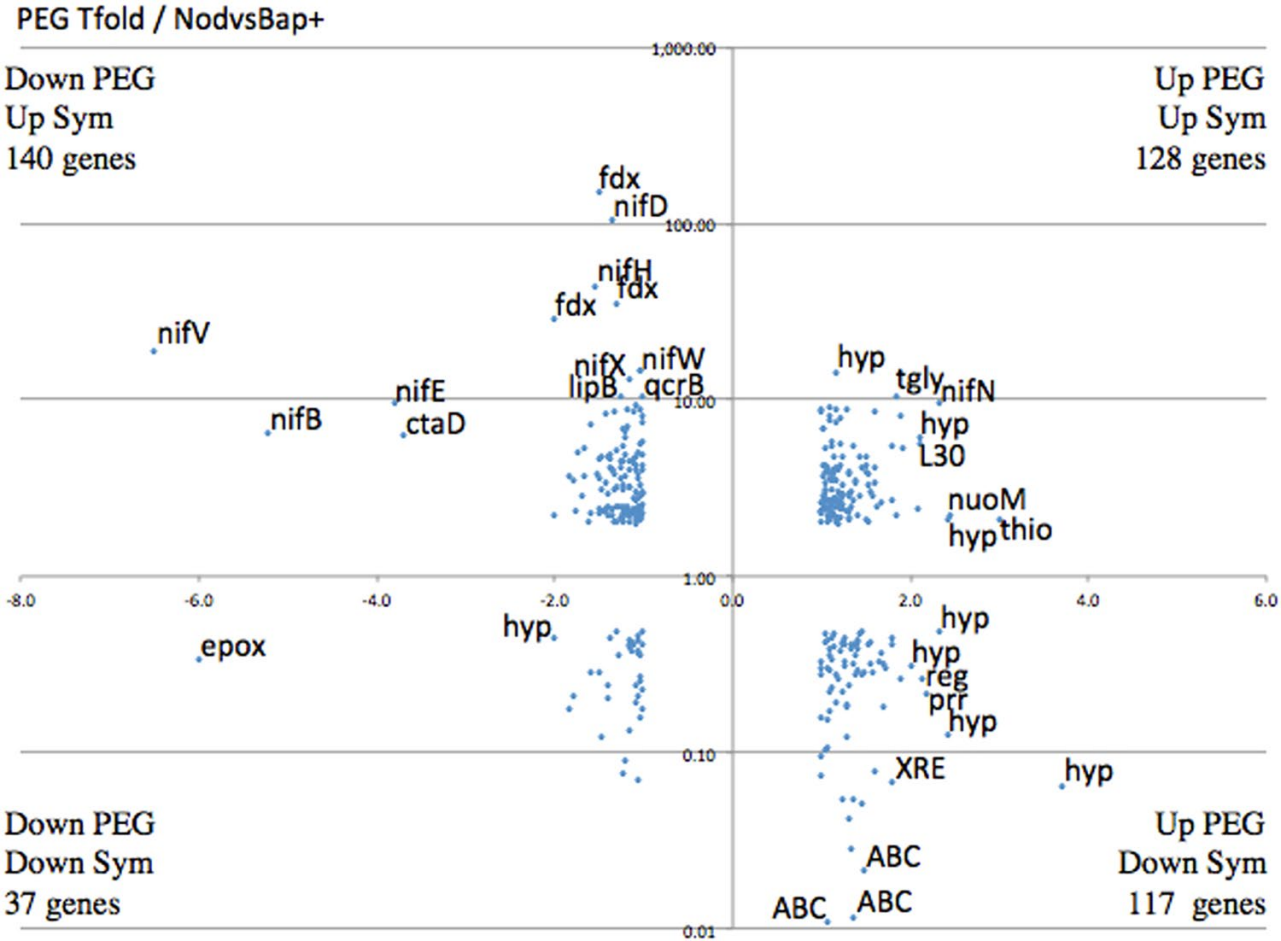
Differentially expressed proteins following polyethylene glycol (PEG) treatment versus differentially expressed proteins involved in symbiosis. Proteomic data of *in vitro* PEG-treated cells on the x-axis with the symbiotic transcriptome^6^ (21 dpi) on the y-axis (scale log10). The symbiotic transcriptome data presented here are those with a FC above 2 and below 0.5 are represented together with those proteomic with FC values above 1 and below −1.

We compared up- and down-regulated proteins related to tolerance to PEG-induced water stress and symbiosis in *F. alni*. Common up- (128) and down-regulated (37) proteins were identified and highlighted in Fig. 4, yielding evidence of common pathways between xerotolerance and symbiosis in *F. alni*.

### FRAAL0095-polyethylene glycol (PEG) interaction investigation

Among the most up-regulated proteins, we chose the FRAAL0095 (Q0RUG3) as a case study. The amino acid sequence of this putative mechanosensitive channel of large conductance (MscL) was submitted to the Swiss Model server^34^. Templates search identified the structure of the MscL of *Mycobacterium tuberculosis* (PDBid: 2OAR) as the template with the highest sequence identity (47.6%) with the target. It is crystallised as a homo-pentamer with three gold ions. This template was selected for model building with respect to its oligo-state. The resulting structure model shown in Supplementary Figure 10 exhibited satisfactory quality scores with a Global Model Quality Estimation (GMQE) score of 0.72. This metric, comprised between zero and one, reflects the expected accuracy of the model with respect to the target-template alignment^34^. The higher the GMQE the more accurate is the model. Another quality assessment was performed through the Ramachandran diagrams (see Supplementary Figure 11) of the homo-pentamer model of FRAAL0095. It presented 11 residues (1.7%) within the outliers region, distributed on all five chains. They mainly occur at the top of the channel (extra-cellular part), or in the trans-membrane region. The global quality of the model was thus considered as satisfactory.

**Figure 5:**
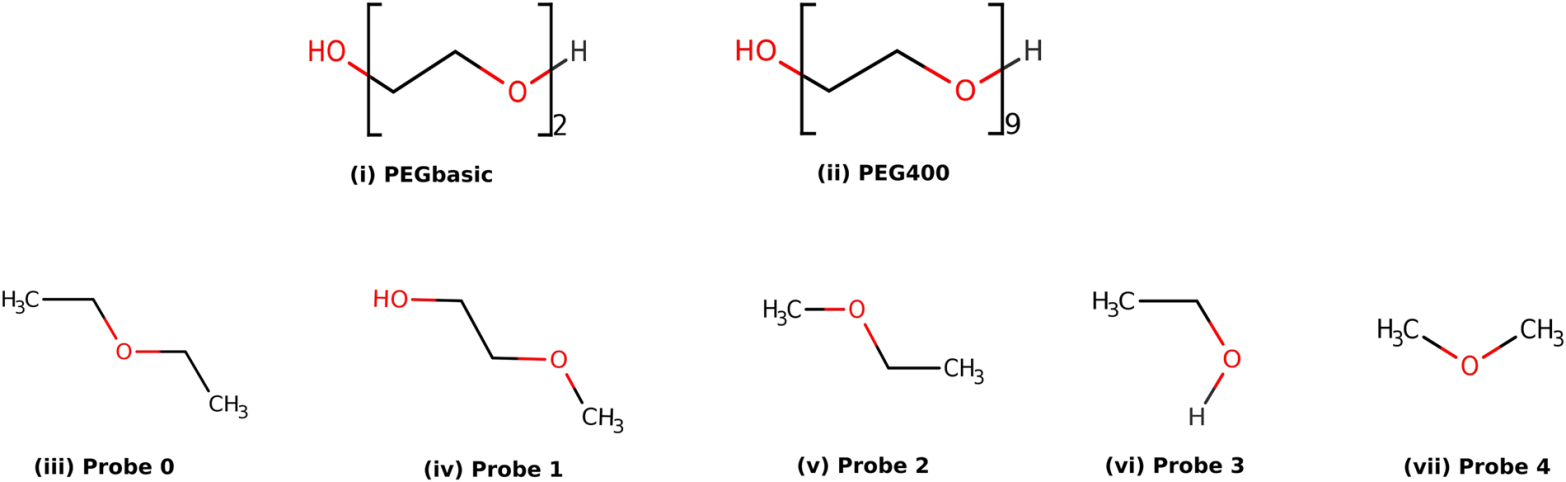
Chemical structures of the PEG400, PEGbasic and derived probes. Probes (0–4) are chemical substructures of PEG.

**Figure 6:**
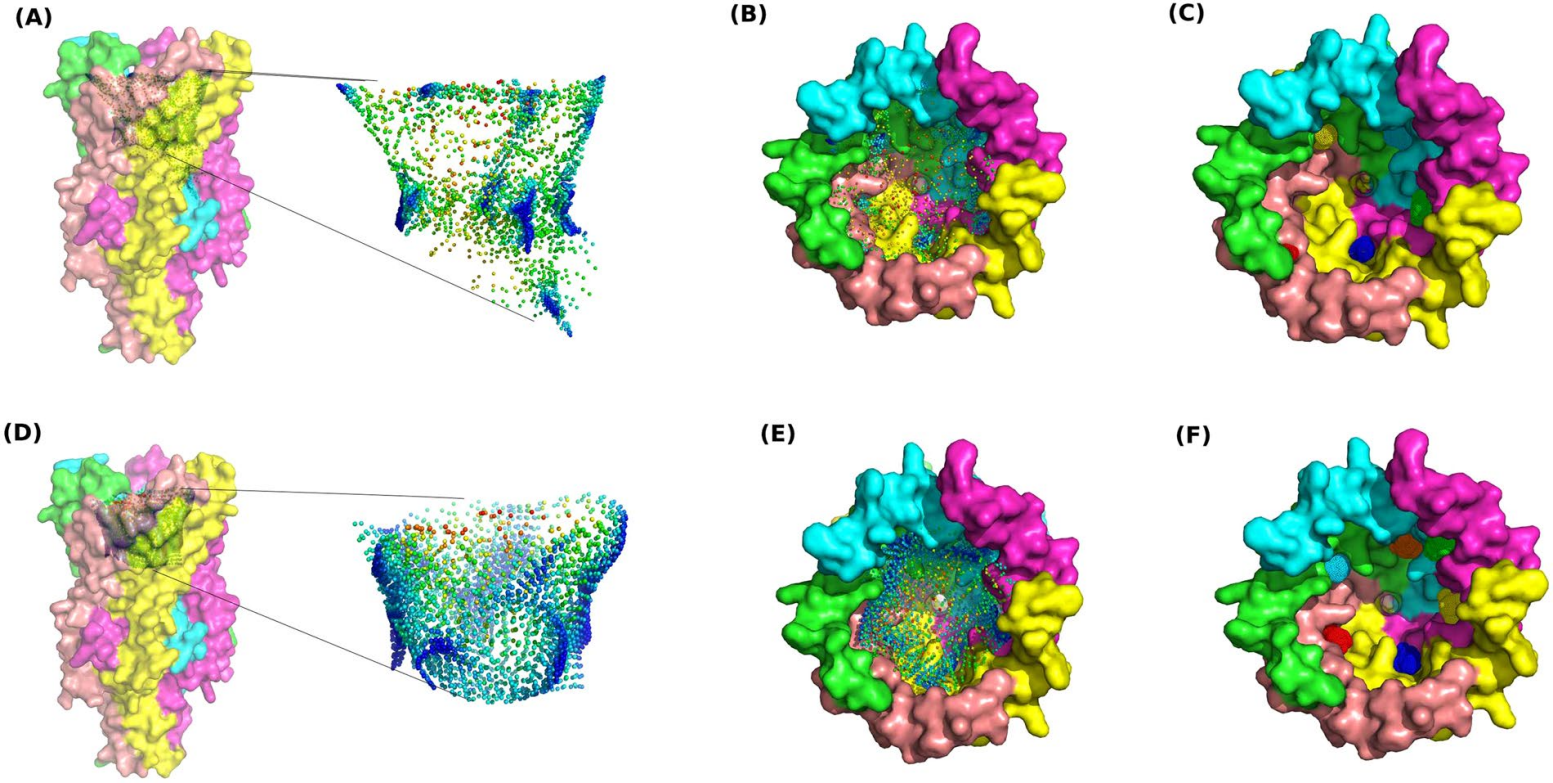
Surface mapping of the FRAAL0095 and identified hot-spots. Panels in the first line present results obtained with the docking of PEG basic and derived probes. Panels in the second line present results obtained with the docking of PEG 400. (**A**) A face-view of the protein structure along with the u-Matrix obtained with SOM-BSfinder analysis of the PEGbasic docking results, with a zoom on the u-Matrix. (**B**) A top-view of the protein structure along the u-Matrix of the PEGbasic docking results. (**C**) A top-view of the protein structure along with the CCs (hot-spots) identified using the SOM-BSfinder analysis of the PEGbasic docking results. (**D**) A face-view of the protein structure along with the u-Matrix obtained with SOM-BSfinder analysis of the PEG400 docking results, with a zoom on the u-Matrix. (**E**) A top-view of the protein structure along the u-Matrix of the PEG400 docking results. (**F**) A top-view of the protein structure along with the CCs (hot-spots) identified using the SOM-BSfinder analysis of the PEG400 docking results.

Docking of the PEGbasic and related probes (Fig. 5) was performed on this structure model. The resulting docking poses were merged and used by the SOM-BSfinder tuned version to define a 3D map of the preferential interaction spots of the PEG and its related probes with the protein surface. The resulting map and hot spots are shown in Fig. 6(A–F). Three populated regions, called Consensual Clusters (CCs), could be identified at equivalent positions between equivalent helices from pairs of adjacent chains of the homo-pentamer (Fig. 6(C)). A fourth CC, ranked as least important and coloured in red on Fig. 6(C), was detected in a higher position than the other CCs.

Moderate and long chain PEG molecules are polymers with the repeating unit -CH_2_CH_2_O-. Their length determines their molecular weights which are computed as follows: MW = 18.02 + 44.05*n, where n in the number of the repeating units. For instance, PEG400 is composed of 8–9 repeating units and PEG8000 is composed of 181–182 repeating units. PEG400 was herein used as a basic model for moderate to long chain PEGs to conduct molecular docking simulations. In fact, the moderate length of PEG400 can be handled by docking programs for accurate calculations while larger sizes (*e.g*., PEG8000) would be time consuming with poor quality (data not shown). We hypothesised that data collected with PEG400 along with the different probes will provide insightful results on the PEG-protein interactions for our case study. PEG400 (see Fig. 5) was thus docked on the structure model of the FRAAL0095. The SOM-BSfinder analysis returned a 3D map with a different shape as compared to the one obtained with the PEGbasic and related probes. It presented higher global density, which can be explained by the length and the flexibility of the polyethylene chain of PEG400. Still, a CC was detected between each two equivalent helices from adjacent chains (Fig. 6(F)), which is consistent with the results obtained with the PEG-basic and the probes. Moreover, an additional CC (coloured in green on Fig. 6(F)) was identified in a higher position than the other CCs. The docking results suggested a folding of the PEG400 inside the channel formed by the five chains. This is consistent with the literature^35,36^. In fact, it was proposed that moderate (PEG800) to long chain (PEG4700 and higher) PEGs would fold themselves and form a corona that mainly establishes hydrophobic interactions with proteins^35–38^. We further identified the residues that may interact with the PEG400 as those located at a maximal distance of 4.0 Å from any of its atoms; and obtained residues alanine 25 (A25), alanine 28 (A28), valine 29 (V29), threonine 31 (T31), valine 34 (V34), lysine 35 (K35), arginine 65 (R65), glutamate 67 (E67), asparagine 70 (N70), serine 71 (S71), alanine 74 (A74) and phenylalanine 75 (F75). These interacting residues were mostly hydrophobic (A, V, F) or polar with neutral side chains (T, S, N). On the protein surface, they constituted a series of symmetric hydrophobic pockets (see Supplementary Figure 10) that define what have previously been described as the hydrophobic core of MscL^39^. Hydrophobic and polar residues appeared to be central to the PEG-FRAAL0095 interactions.

## Discussion

Little is known about the means by which *Frankia* cells tolerate desiccation in the soil^10^. In view of the fact that increasing concentrations of greenhouse gases in the atmosphere and climate change are expected to modify the global water cycle and to increase the odds of worsening drought in the next decades^40^, an understanding of the responses of *F. alni* to desiccation and thus to osmotic stress becomes an important issue in the attempt to adapt symbiosis to desiccated niches. In the present work we have analysed the expression of proteins in the alder symbiont *F. alni* in response to PEG-induced water stress to characterise the impact of desiccation on nitrogen-fixing symbiotic bacteria. We used liquid medium since it easier to have reproducible conditions with healthy cells devoid of agar.

The results of the experiments summarised in Tables 1 and 2 indicated six most up-regulated and three most down-regulated PEG-modulated response mechanisms, respectively. The most remarkable differences in the analysed proteomes of *F. alni* cells grown with and without PEG could be assigned to envelope-associated proteins (*e.g*., FRAAL2567 (5.67) and FRAAL2549 (4.8); *cf*. Table 1). FRAAL2567 is a putative autotransporter adhesin, which is concordant with previous studies indicating the importance of proteins associated with the modulation of the structure and function of the three-dimensional extracellular matrix under stress conditions—desiccation, UV irradiation and oxidation. In addition, cell envelope remodelling, transcriptional and translational regulation and salt-stress responsive hypothetical proteins were shown to be important for salt-tolerant *Frankia* strains^13^. A previous study^20^ that concords with present results (Table 1), showed that *Rhodococcus jostii* RHA1 desiccation-specific transcriptome had several genes associated with cellular processes including lipid metabolism and cell envelope modification. ABC transporters, like FRAAL2549, are integral membrane proteins that typically use cellular energy to translocate solutes across cellular membranes in all phyla^41^. Through the transport of molecules, ABC transporters are involved in diverse cellular processes including osmotic homeostasis and resistance to xenotoxins^42^. Moreover, up-regulation of a potassium transporter (FRAAL2085 (1.65)) was observed. Orthologs of this protein were reported to scavenge K^+^ ions from the environment and maintain cell hydrostatic pressure in response to desiccation. In addition, MscL, one of the best studied highly conserved mechanosensitive channels throughout the bacterial kingdom^43^, was found to be highly up-regulated in *F. alni* following exposure to PEG (FRAAL0095 (4.30)). This transmembrane channel acts through switching from closed to open conformation under severe osmotic challenges by opening a water-filled pore^44,45^. Molecular modelling revealed potential interacting sites of the PEG molecule inside the channel formed by five monomers of MscL of *F. alni*. These sites were located between each pair of adjacent chains in a symmetric frame. Interactions with the PEG mainly included hydrophobic and polar residues that define the hydrophobic core that is *sine qua none* to the channel activity^46^. Recently, the co-crystal structure of the C-terminal SH3 domain of myosin IB from *Entamoeba histolytica* with PEG was reported^47^. Gautam and colleagues^47^ brought insightful elements for a better understanding of the interactions of PEG with protein surfaces and reported in particular the corona-like binding mode previously described^48–51^. Our findings are consistent with the literature, which adds confidence to our computational approach. In fact, long-chain PEGs are flexible polymers with multiple rotatable bonds and high degrees of freedom, which makes them a challenging case for molecular docking software. In the present work, we established a protocol to circumvent this issue by using the repeating unit of the PEG and its chemical features as probes in order to infer a simplistic model for docking. Then, the SOM-based method permitted to generate an accurate map of the binding hot spots on the MscL surface, along with a prediction of the overall binding mode. Our results suggest that long-chain PEGs may act as an inner coat of the channel^39,52^. This mode of binding may either induce an increase in water molecules conductance through the channel or induce a mechanistic blocking of its entrance. Our findings constitute a response element to the role of MscL (FRAAL0095) in the *F. alni* desiccome, and a starting point to conduct further investigations on the PEG-FRAAL0095 interactions.

As expected, envelope stress would induce a cascade of other defence pathways. Most notable is the induction of one CRISPR-associated proteins (FRAAL0457 (1.67) being part of the Green class) supporting published observations that expression of CRISPR-Cas components in several bacterial species can be induced following envelope stress^53^. Supplementary Table S3 lists the CRISPR-associated PEG-induced proteins of *F. alni*. Also, the present analysis revealed the up-regulation of proteins associated with cell osmoprotection. For instance, FRAAL1888 (2.20) and FRAAL2171 (1.5) are involved in the synthesis of the dipeptide N-acetylglutaminylglutamine amide (NAGGN) that plays a key role in osmoprotection^54^. In addition to its role in NAGGN biosynthesis, FRAAL1888 is involved in the conversion of glutamine into glutamate, one of the most abundant and up-regulated amino acids in *Alnus* and *Casuarina* nodules, for which a role as osmolyte was postulated by Brooks and Benson^55^. Also, the FRAAL5155 peptidase (1.91), by the cleavage of dipeptides into single peptides, would provide needed intracellular osmolytes to offset the high extracellular osmotic pressure.

In addition, consistent with previous studies, we observed a significant up-regulation of FRAAL4431 (4) encoding a Clp protease. Indeed, ClpP proteases play an essential role in removing damaged proteins from low GC Gram-positive bacteria under stress conditions such as osmotic shock^56^. Furthermore, proteolysis of such proteins would release amino acids that would in turn provide osmolytes to help compensate the osmotic shock. Similarly, a Clp-protease was induced during desiccation and dehydration in *Pyropia columbina*^57^. FRAAL1222 encoding a carbonic anhydrase and presumably involved in stress response was quantified in larger amount (2.8) in PEG-grown *F. alni* cells. It is expected that this enzyme, by hydrating CO_2_ to carbonate (H_2_CO_3_), would increase the concentration of protons within the cell and decrease the pH. A previous study^18^ showed an increase in the expression of an orphan carbonic anhydrase in the cyanobacterium *Microcoleus vaginatus* using a whole genome transcriptional time course assay in response to hydration. The observed increase in several ROS-scavenging proteins and translation-associated ribosome proteins provided evidence in support of a higher demand of detoxification and protein synthesis during growth under PEG-induced water stress conditions. For example, FRAAL6022 (2.17) encodes a gamma-aminobutyraldehyde dehydrogenase (GABA synthesis). GABA can act as a hydroxyl radical scavenger^58^.

*Deinococcus radiodurans*’ xerotolerance was explained by the phenomenon of gene sharing^59^: during land colonization by *Terrabacteria*, gene products (*e.g*., DNA repair genes, *etc*.) contributing to this bacterium’s ionizing radiation were recruited to serve an additional function that is desiccation tolerance^60^. In *F. alni*, we observed an upregulation of the SOS response genes transcriptional repressor LexA (1.56) with an expected downregulation of some DNA repair proteins (Table 2). Furthermore, the investigation of the relationship between desiccation tolerance and nitrogen metabolism in *Anabaena* sp. PCC 7120 suggested that terrestrial cyanobacteria may acclimate to desiccation stress *via* nitrogen (N_2_) fixation by using desiccation inducible genes^61^. In our study, TFold analysis pinpointed three classes of differentially down-regulated proteins (*e.g*., FRAAL6814 (−6.5), FRAAL4147 (−3.7) and FRAAL6528 (−2.36); *cf*. Table 2). The remarkable decrease in protein expression related to nitrogen fixation (more than sixfold), aerobic respiration (more than threefold) and homologous recombination (more than twofold) quantitatively reflects the magnitude of the impact of nitrogen-, oxygen- and homologous-recombination-associated energy demands in PEG-grown *F. alni* cells.

Identified common pathways between symbiosis^6^ and desiccation tolerance (Fig. 4) are in accordance with the fact that plant-growth-promoting (PGP) rhizobacteria augment plant tolerance to drought^62^. In line with this, it was recently demonstrated that bacterial endophytic communities promote date palm (*Phoenix dactylifera* L.) growth under drought conditions^63^. Also, it was shown that plant growth promotion ability exerted by bacteria is a drought-induced effect^64^. In addition, a previous work^25^ showed an induction of the threonine dehydratase (DR_0567)—converting threonine into NH_3_/NH_4_^+^—and the nodulation efficiency protein D (NfeD, DR_2142 (WP_010888773.1)) following desiccation of *D. radiodurans* R_1_.

Penetration into host tissues implies many stresses, one of which is osmotic since *Alnus glutinosa*, as most alder species, thrives on river banks where water has low salt levels, while root tissues have a higher, isotonic osmotic potential. Furthermore, alder synthesises many peptides upon entry of *Frankia*, many of which are defensins that bind to the cell membrane and modify its porosity^65^ and thus the ability to cope with osmolytes. Rhizobia have been shown to have many determinants associated with desiccation tolerance, many of which have similarities to those seen upregulated in *Frankia* such as transporters^66^, the mechanosensitive channel^67^ or disaccharide accumulation^68^. However the link between osmotic response and symbiosis establishment was not very strong, implying the responses seen here would have more usefulness for saprotrophic soil existence.

## Conclusions

The present paper is the first high-throughput proteomic study of *F. alni* subjected to water-related stress. This research, analysing the most representative proteome of a *Frankia* strain, contributes to a better comprehension of environmental stress adaptation particularly in desiccated soils. In the future, our data might be used in further comparative proteogenomic studies of nitrogen-fixing plant symbionts.

## Materials and Methods

### Bacterial growth and treatment with polyethylene glycol (PEG)

*F. alni* strain ACN14a^69^ was grown in liquid BAP- medium without ammonium as described earlier^70^. The cells were then syringed thoroughly with needles of decreasing width (21G–27G), the cells OD_600_ measured and the suspension diluted prior to inoculation. An OD_600_ inoculation of 0.1 was used to make a growth dynamic and to monitor ARA activity at 0.3, 0.9 and 2.8% PEG8000 (Sigma). Inoculations were then made at the same OD_600_ in fresh BAP- medium without or with PEG8000 (Sigma) added to a final concentration of 0.9% w.vol^−1^. The cells were grown in 125 mL Erlenmeyers with 40 mL medium at 28 °C on an orbital shaker at 200 rpm for 7 days. The cells were harvested, their OD_600_ measured, an aliquot inoculated onto LB agar plates to detect contaminations and the cells observed under the microscope. The cells were then sedimented (1,500 × g for 5 minutes) and the pellets frozen until proteomics analysis.

### Proteomic sample preparation and nanoscale liquid chromatography coupled to tandem mass spectrometry (nano LC-MS/MS) analysis of tryptic peptides

The protein content of cells grown without PEG and in the presence of PEG was established by a shotgun procedure. Following trypsin proteolysis, peptides were analysed by nanoLC-MS/MS with a Q-Exactive HF tandem mass spectrometer incorporating an ultra-high field Orbitrap analyser. Five independent biological replicates were analysed for both conditions: PEG-treated bacteria and untreated bacteria. A volume of 5 µL of LDS (1X) was added per mg of bacterial pellet prior a 5 min heat treatment at 99 °C followed by a treatment for 5 min in an ultrasonic bath. Cells and debris were transferred into a 2 mL Precellys (Bertin Technologies, F-78180 Montigny le Bretonneux) tube containing 200 mg of glass beads and the samples were subjected to 3 cycles of grinding at 6,500 rpm for 20 sec by means of a Precellys grinder (Bertin technologies). Samples were then centrifuged for 40 sec at 16,000 g. The resulting supernatants were incubated for 10 min at 99 °C and were subjected to a short SDS-PAGE migration as previously described^71^. The polyacrylamide bands containing the whole solubilised protein content of each sample were processed as previously described^72^. Briefly, they were subjected to DTT reduction, treated with iodoacetamide and then, proteolysed with Sequencing Grade Trypsin (Roche, F-38240 Meylan) using 0.01% of proteaseMAX detergent (Promega, F-69260 Charbonnières-les-Bains). The resulting peptides (10 μL) were analysed in data-dependent mode with an ESI-Q Exactive HF mass spectrometer (ThermoFisher Scientific, F-91963, Courtaboeuf) equipped with an ultra-high field Orbitrap analyser and coupled to an Ultimate 3000 176 RSL Nano LC System (ThermoFisher). Peptides were injected onto a reverse phase Acclaim PepMap 100 C18 column (3 µm, 100 Å, 75 µm id × 500 mm) and resolved at a flow rate of 0.2 µL/min with a 60 min gradient of CH3CN in presence of 0.1% formic acid. A dataset of 10 nanoLC-MS/MS runs were recorded. The Q-Exactive HF instrument was operated with Top20 standard parameters and a dynamic exclusion of 10 sec as previously described^73^. MS/MS spectra were searched using MASCOT 2.2.04 software (Matrix Science, London, W1U 7GB, UK) against the *F. alni* database (5,804 protein sequences) with the following parameters: full-trypsin specificity, maximum of two missed cleavages, mass tolerances of 5 ppm on the parent ion and 0.02 Da on the higher energy collisional dissociation-induced peptidic fragments, fixed modification of carboxyamidomethylated cysteine (+57.0215), and oxidised methionine (+15.9949) as dynamic modifications. Peptide-to-MS/MS spectrum matching with a MASCOT score below a *p*-value of 0.05 were selected and assigned to unique peptide sequence following the parsimony principle. A protein was considered valid when at least two different peptides were detected. The false-positive rate for protein identification was estimated by a search with a reverse decoy database to be below 0.1% using the same parameters. Proteins were quantified based on their spectral counts. The normalised spectral abundance factor (NSAF) was calculated by dividing the spectral count for each observed protein by the polypeptide theoretical mass, as described previously^30^ and is presented as percentage of the NSAF sum considering all proteins. Proteome comparison between both conditions was done taking into account the five biological replicates with the TFold module from the PatternLab software and standard normalisation, as previously described^71^.

### Homology-based functional analysis and Clusters of Orthologous Groups of proteins (COGs) prediction

Protein sequences of the PEG-responding differentially expressed proteins were retrieved from the National Center for Biotechnology Information (NCBI) database^74^ and uploaded into the STRING database^32^ analysis tool for Gene Ontologies and metabolic pathways functional enrichments. Biological processes and metabolic pathways with False Discovery Rate (FDR) ≤ 0.005 were considered as significant. *F. alni* Clusters of Orthologous Groups of proteins (COGs) were inferred for an important set of proteins based on BLASTP^75^ best reciprocal hits with *Frankia* sp. EAN1pec for which these clusters are known and available in the database of COGs^76^.

### Protein-protein interaction network

The sequences of the 294 proteins of *F. alni* belonging to the PatternLab blue class (for which identifications satisfied both the fold (>1.5) and statistical criteria (*p*-value < 0.05)) were downloaded and saved in a multi-FASTA file. This file was then uploaded in the STRING database^32^ and *F. alni* was chosen as a query microorganism. A total of 294 hits with 100% identity were detected and their protein-protein interactions were predicted. In order to generate protein-protein interactions integrating the expression data information of each protein, data were integrated into the Cytoscape tool^77^. Expression values were imported as node attributes. The same steps were followed to analyse protein-protein interaction networks of the other detected proteins (belonging to the PatternLab orange, green and red classes (see Results section for details)). All single nodes were not represented with Cytoscape which considers only interacting proteins.

### Re-annotation of differentially expressed hypothetical proteins

Differentially expressed proteins that were annotated as hypothetical (or conserved hypothetical) were re-annotated. For this, we conducted new analyses using the Position-Specific Iterated BLAST (PSI-BLAST) program^78^ at http://blast.ncbi.nlm.nih.gov/ with default parameters against a database of non-redundant protein sequences (nr).

### Molecular modelling and docking

#### Comparative modelling

Protein sequence of the FRAAL0095, one of the most up-regulated *Frankia* genes (4.30), identified as coding for an osmotic-stress large conductance mechanosensitive channel, was submitted to the Swiss Model Server^34^. Search for templates of experimentally determined protein structures within the Swiss-Model Template Library (SMTL) was performed. Identified templates, ranked according to their sequence identity rate (IR) and their coverage rate (CR) with the target sequence, were examined. The template(s) with the highest IR and CR were selected for model building. The model with the most satisfactory quality assessment scores was considered for the subsequent analysis. Ramachandran diagrams of the selected model were generated using the RAMPAGE server^79^, and used to assess the 3D model quality.

#### Molecular docking and surface mapping

Molecular docking of the PEG and related molecules (herein called ligands) on the structure model of the protein target (herein called receptor) was performed using AutoDock Vina 1.1.2^80^. The latter program requires input files of the ligands and the receptor in PDBQT (Protein Data Bank^81^, Partial Charge (‘Q’), and Atom Type (‘T’)) format. Default parameters were used and a maximum of 20 lowest-energy poses were kept for each ligand. Two forms of the PEG were considered: (i) a short form having the formula C_2_H_10_O_3_ and is referred to as PEGbasic and (ii) a longer form having the formula C_18_H_38_O_10_ to which we refer as PEG400. Additionally, five chemical substructures of the PEG were generated and used as probes to map hot spots on the protein surface. Simplified Molecular Input Line Entry System (SMILES) conformations of PEGbasic, PEG400 and the probes were generated manually, then converted to PDBQT format using the OpenBabel package (http://openbabel.org)^82^. The PDB file of the target was used to generate the corresponding PDBQT format by adding hydrogen atoms and atomic partial charges using the OpenBabel package^82^. Each ligand was docked 500 times on the protein. Docking results were analysed using a customised version of the SOM-BSfinder method^83^. Herein, input data consisted in atomic coordinates of the docked PEGbasic, PEG400, and the five probes. Otherwise, default parameters were used as described previously^83^. PyMol 1.8.2.1 (Schrodinger, LLC)^84^ was used to visualise the results and generate the figures.

### Proteomics data repository

The mass spectrometry proteomic data were deposited at the ProteomeXchange Consortium (http://proteomecentral.proteomexchange.org) *via* the PRIDE partner repository with the data set identifiers PXD007226 and DOI 10.6019/PXD007226.

## Electronic supplementary material

Dataset1
