# Supplementary material for: The PEG-responding desiccome of the alder microsymbiont Frankia alni
Source: Sci Rep. 2018 Jan 15;8:759. doi: 10.1038/s41598-017-18839-0 (PMC5768760; doi:10.1038/s41598-017-18839-0)
Supplement: Supplementary file 1 — Dataset1 [file 41598_2017_18839_MOESM1_ESM.doc]

# The PEG-responding desiccome of the alder microsymbiont *Frankia alni*

Kais Ghedira, Emna Harigua-Souiai, Cherif Ben hamda, Pascale Fournier, Petar Pujic, Sihem Guesmi, Ikram Guizani, Guylaine Miotello, Jean Armengaud, Philippe Normand, Haïtham Sghaier

| Additional data Table S1. List of detected proteins and their spectral count. | | | | |  |  |  |  |  |  |  |  |  |  |  |  |  |  |  |  |  |  |  |  |  |  |  |  |  |
| --- | --- | --- | --- | --- | --- | --- | --- | --- | --- | --- | --- | --- | --- | --- | --- | --- | --- | --- | --- | --- | --- | --- | --- | --- | --- | --- | --- | --- | --- |
|  | **Database :** | **Frankia.alni.reduite_20160419.fasta 5804 seq** |  |  |  |  |  |  |  |  |  |  |  |  |  |  |  |  |  |  | PatternLab's TFold module | |  |  |  |  |  |  |  |
|  | **Enzyme :** | **Trypsin - Max missed cleavages: 2** |  |  |  |  |  |  |  |  |  |  |  |  |  |  |  |  |  |  | Blue class: Identifications satisfied both. the fold (<1.5) and statistical criteria (p value <0.05). | | | | | | |  |  |
|  | **Fixed modifications :** | **Carbamidomethyl (C)** |  |  |  |  |  |  |  |  |  |  |  |  |  |  |  |  |  |  | Orange class: These identifications did not meet the fold criterion but they deserve a second look because they have low p-values. | | | | | | | | |
|  | **Variable modifications :** | **Deamidated (NQ). Oxidation (M)** |  |  |  |  |  |  |  |  |  |  |  |  |  |  |  |  |  |  | Green class: These identifications satisfied the fold criteria but. most likely. this happened by chance (should be disconsidered). | | | | | | | | |
|  | **MS/ MS² mass tol. :** | **± 5 ppm / ± 0.02 Da** |  |  |  |  |  |  |  |  |  |  |  |  |  |  |  |  |  |  | Red class: These identifications did not meet the fold and p-value criteria (should be disconsidered). | | | | | | |  |  |
|  | **Instrument type :** | **ESI Qexactive** |  |  |  |  |  |  |  |  |  |  |  |  |  |  |  |  |  |  |  |  |  |  |  |  |  |  |  |
|  |  |  |  |  |  |  |  |  |  |  |  |  |  |  |  |  |  |  |  |  |  |  |  |  |  |  |  |  |  |
|  |  |  | **All Total peptides** | **All Total SC** |  |  |  |  |  |  |  |  |  |  |  |  |  |  |  |  |  |  |  |  |  |  |  |  |  |
|  |  |  | 20825 | 208014 |  |  |  |  |  |  |  |  |  |  |  |  |  |  |  |  |  |  |  |  |  |  |  |  |  |
|  |  |  |  |  |  |  |  |  |  |  |  |  |  | **Spectral count** | |  |  |  |  | **Spectral count** | |  |  |  |  | **PatternLab's TFold module** | |  |  |
|  |  |  |  |  |  |  | **Best hit** |  |  |  |  |  |  | **Frankia-1** | **Frankia-2** | **Frankia-3** | **Frankia-4** | **Frankia-5** |  | **Frankia-6** | **Frankia-7** | **Frankia-8** | **Frankia-9** | **Frankia-10** |  |  |  |  |  |
| **Protein accession** | **FRAAL** | **Functional description** | **Molecular weight (Da)** | **pI** | **Total peptides** | **Total Spectral count** | **Best sample** | **.DAT file** | **MASCOT score** | **Sequence coverage (%)** | **#peptides** | **#specific peptides** | **SC** | **BAP-1(Ref)** | **BAP-2 (Ref)** | **BAP-3 (Ref)** | **BAP-4 (Ref)** | **BAP-5 (Ref)** | **% NSAF** | **BAP-PEG 1** | **BAP-PEG 2** | **BAP-PEG 3** | **BAP-PEG 4** | **BAP-PEG 5** | **% NSAF** | **Tfold** | **Ln(Tfold)*sign** | **p value** | **PatternLab Class** |
| WP_011603724.1 | FRAAL2567 | hypothetical protein | 28798 | 5.98 | 12 | 30 | Frankia-8 | F201371.dat | 554 | 44 | 9 | 9 | 9 | 0 | 1 | 0 | 0 | 0 | 0.001% | 0 | 7 | 9 | 7 | 6 | 0.034% | 5.67 | 1.73 | 3.6E-03 | Blue |
| WP_041939153.1 | FRAAL2549 | daunorubicin resistance protein DrrA family ABC transporter ATP-binding protein | 33359 | 5.37 | 5 | 19 | Frankia-8 | F201371.dat | 216 | 16 | 5 | 5 | 6 | 0 | 0 | 0 | 0 | 0 | 0.000% | 2 | 4 | 6 | 3 | 4 | 0.019% | 4.80 | 1.57 | 4.5E-04 | Blue |
| WP_009741588.1 | FRAAL6743 | cold-shock protein | 14313 | 9.68 | 9 | 34 | Frankia-9 | F201372.dat | 267 | 64 | 9 | 9 | 9 | 0 | 0 | 0 | 3 | 0 | 0.007% | 3 | 4 | 7 | 9 | 8 | 0.073% | 4.50 | 1.50 | 1.3E-03 | Blue |
| WP_011601362.1 | FRAAL0095 | large mechanosensitive ion channel protein MscL | 14395 | 9.22 | 2 | 43 | Frankia-8 | F201371.dat | 115 | 21 | 2 | 2 | 7 | 2 | 1 | 0 | 1 | 1 | 0.012% | 6 | 7 | 7 | 10 | 8 | 0.089% | 4.30 | 1.46 | 1.1E-05 | Blue |
| WP_011605553.1 | FRAAL4431 | Clp protease | 20785 | 5.65 | 4 | 15 | Frankia-8 | F201371.dat | 231 | 29 | 4 | 4 | 7 | 0 | 0 | 0 | 0 | 0 | 0.000% | 0 | 1 | 7 | 4 | 3 | 0.024% | 4.00 | 1.39 | 2.3E-02 | Blue |
| WP_011605162.1 | FRAAL4031 | hypothetical protein | 15857 | 5.73 | 8 | 23 | Frankia-10 | F201373.dat | 171 | 32 | 4 | 4 | 7 | 0 | 0 | 2 | 0 | 0 | 0.005% | 2 | 4 | 3 | 5 | 7 | 0.045% | 3.71 | 1.31 | 2.0E-03 | Blue |
| WP_050997139.1 | FRAAL3563 | TetR family transcriptional regulator | 23374 | 6.14 | 3 | 15 | Frankia-10 | F201373.dat | 156 | 15 | 3 | 3 | 4 | 0 | 0 | 1 | 0 | 0 | 0.002% | 0 | 3 | 4 | 3 | 4 | 0.020% | 3.17 | 1.15 | 6.1E-03 | Blue |
| WP_011605896.1 | FRAAL4781 | thioesterase | 20641 | 5.61 | 5 | 10 | Frankia-8 | F201371.dat | 136 | 15 | 4 | 4 | 4 | 0 | 0 | 0 | 0 | 0 | 0.000% | 1 | 2 | 4 | 1 | 2 | 0.016% | 3.00 | 1.10 | 7.1E-03 | Blue |
| WP_011601373.1 | FRAAL0107 | ATP-binding protein | 13755 | 6.02 | 4 | 10 | Frankia-10 | F201373.dat | 90 | 30 | 4 | 4 | 4 | 0 | 0 | 0 | 0 | 0 | 0.000% | 0 | 2 | 1 | 3 | 4 | 0.024% | 3.00 | 1.10 | 1.7E-02 | Blue |
| WP_009742328.1 | FRAAL5034 | hypothetical protein | 8497 | 10.93 | 6 | 41 | Frankia-10 | F201373.dat | 261 | 64 | 6 | 6 | 8 | 0 | 1 | 2 | 2 | 3 | 0.034% | 4 | 3 | 9 | 9 | 8 | 0.131% | 2.92 | 1.07 | 3.4E-03 | Blue |
| WP_011602615.1 | FRAAL1420 | nitrate ABC transporter ATP-binding protein | 28426 | 9.08 | 6 | 25 | Frankia-10 | F201373.dat | 221 | 24 | 5 | 5 | 5 | 1 | 2 | 0 | 1 | 0 | 0.005% | 3 | 4 | 4 | 5 | 5 | 0.025% | 2.89 | 1.06 | 1.0E-04 | Blue |
| WP_011606932.1 | FRAAL5864 | MarR family transcriptional regulator | 18382 | 8.28 | 6 | 9 | Frankia-10 | F201373.dat | 103 | 28 | 4 | 4 | 4 | 0 | 0 | 0 | 0 | 0 | 0.000% | 0 | 1 | 2 | 2 | 4 | 0.016% | 2.80 | 1.03 | 2.1E-02 | Blue |
| WP_011602423.1 | FRAAL1222 | carbonic anhydrase | 21311 | 5.09 | 9 | 47 | Frankia-10 | F201373.dat | 562 | 69 | 9 | 9 | 14 | 0 | 2 | 0 | 7 | 1 | 0.017% | 8 | 6 | 0 | 9 | 14 | 0.058% | 2.80 | 1.03 | 3.7E-02 | Blue |
| WP_011605928.1 | FRAAL4814 | hypothetical protein | 22950 | 10.97 | 4 | 16 | Frankia-7 | F201370.dat | 172 | 22 | 4 | 4 | 4 | 0 | 0 | 1 | 1 | 0 | 0.003% | 3 | 4 | 2 | 2 | 3 | 0.021% | 2.71 | 1.00 | 6.7E-04 | Blue |
| WP_011607117.1 | FRAAL6065 | hypothetical protein | 24323 | 6.51 | 3 | 8 | Frankia-10 | F201373.dat | 75 | 11 | 2 | 2 | 2 | 0 | 0 | 0 | 0 | 0 | 0.000% | 1 | 1 | 2 | 2 | 2 | 0.011% | 2.60 | 0.96 | 4.6E-03 | Blue |
| WP_011606354.1 | FRAAL5264 | hypothetical protein | 9942 | 11.32 | 2 | 8 | Frankia-8 | F201371.dat | 138 | 32 | 2 | 2 | 2 | 0 | 0 | 0 | 0 | 0 | 0.000% | 1 | 2 | 2 | 2 | 1 | 0.027% | 2.60 | 0.96 | 4.6E-03 | Blue |
| WP_035922554.1 | FRAAL3761 | AsnC family transcriptional regulator | 15941 | 5.89 | 3 | 8 | Frankia-8 | F201371.dat | 132 | 17 | 2 | 2 | 2 | 0 | 0 | 0 | 0 | 0 | 0.000% | 0 | 2 | 2 | 2 | 2 | 0.017% | 2.60 | 0.96 | 7.5E-03 | Blue |
| WP_011607077.1 | FRAAL6025 | AsnC family transcriptional regulator | 17832 | 5.19 | 6 | 26 | Frankia-9 | F201372.dat | 385 | 47 | 6 | 6 | 7 | 2 | 0 | 3 | 0 | 0 | 0.010% | 2 | 4 | 5 | 7 | 3 | 0.040% | 2.60 | 0.96 | 8.6E-03 | Blue |
| WP_011605954.1 | FRAAL4839 | peptide ABC transporter | 53497 | 6.47 | 3 | 15 | Frankia-7 | F201370.dat | 158 | 7 | 3 | 3 | 3 | 0 | 0 | 0 | 2 | 0 | 0.001% | 1 | 3 | 3 | 3 | 3 | 0.008% | 2.57 | 0.94 | 2.3E-03 | Blue |
| WP_011606387.1 | FRAAL5296 | hypothetical protein | 43454 | 5.95 | 5 | 15 | Frankia-7 | F201370.dat | 146 | 13 | 5 | 5 | 5 | 0 | 0 | 0 | 0 | 2 | 0.002% | 1 | 5 | 2 | 2 | 3 | 0.010% | 2.57 | 0.94 | 1.2E-02 | Blue |
| WP_041939763.1 | FRAAL5819 | phosphomethylpyrimidine kinase | 28431 | 6.44 | 5 | 15 | Frankia-8 | F201371.dat | 255 | 31 | 5 | 5 | 5 | 0 | 0 | 1 | 1 | 0 | 0.003% | 3 | 1 | 5 | 3 | 1 | 0.015% | 2.57 | 0.94 | 1.4E-02 | Blue |
| WP_011602297.1 | FRAAL1075 | DNA glycosylase | 21281 | 6.98 | 6 | 29 | Frankia-10 | F201373.dat | 249 | 33 | 5 | 5 | 7 | 2 | 0 | 2 | 2 | 0 | 0.010% | 3 | 3 | 4 | 6 | 7 | 0.036% | 2.55 | 0.93 | 3.6E-03 | Blue |
| WP_011601287.1 | FRAAL0019 | peptide deformylase | 24664 | 5.30 | 6 | 18 | Frankia-8 | F201371.dat | 146 | 25 | 4 | 4 | 4 | 0 | 0 | 0 | 2 | 1 | 0.004% | 0 | 3 | 4 | 4 | 4 | 0.020% | 2.50 | 0.92 | 1.2E-02 | Blue |
| WP_011605344.1 | FRAAL4217 | methyltransferase type 11 | 29334 | 10.74 | 4 | 11 | Frankia-8 | F201371.dat | 129 | 17 | 4 | 4 | 4 | 0 | 0 | 1 | 0 | 0 | 0.001% | 0 | 2 | 4 | 1 | 3 | 0.011% | 2.50 | 0.92 | 2.5E-02 | Blue |
| WP_011602271.1 | FRAAL1044 | NADH:ubiquinone oxidoreductase subunit M | 62926 | 6.87 | 5 | 21 | Frankia-6 | F201369.dat | 154 | 9 | 5 | 5 | 5 | 2 | 0 | 1 | 1 | 0 | 0.002% | 5 | 3 | 4 | 2 | 3 | 0.009% | 2.44 | 0.89 | 1.7E-03 | Blue |
| WP_011603266.1 | FRAAL2100 | membrane protein | 13461 | 11.36 | 2 | 14 | Frankia-10 | F201373.dat | 87 | 35 | 2 | 2 | 3 | 0 | 0 | 0 | 2 | 0 | 0.005% | 2 | 3 | 3 | 1 | 3 | 0.030% | 2.43 | 0.89 | 3.8E-03 | Blue |
| WP_011605591.1 | FRAAL4469 | hypothetical protein | 19003 | 10.41 | 4 | 31 | Frankia-7 | F201370.dat | 127 | 15 | 3 | 3 | 6 | 1 | 1 | 2 | 2 | 1 | 0.013% | 5 | 6 | 5 | 4 | 4 | 0.043% | 2.42 | 0.88 | 6.8E-05 | Blue |
| WP_011605459.1 | FRAAL4335 | C4-dicarboxylate ABC transporter substrate-binding protein | 38764 | 5.28 | 6 | 24 | Frankia-9 | F201372.dat | 268 | 22 | 5 | 5 | 6 | 0 | 0 | 2 | 2 | 1 | 0.005% | 3 | 4 | 3 | 6 | 3 | 0.017% | 2.40 | 0.88 | 2.6E-03 | Blue |
| WP_011602506.1 | FRAAL1308 | hypothetical protein | 47106 | 5.71 | 3 | 7 | Frankia-10 | F201373.dat | 85 | 6 | 2 | 2 | 2 | 0 | 0 | 0 | 0 | 0 | 0.000% | 0 | 1 | 2 | 2 | 2 | 0.005% | 2.40 | 0.88 | 1.4E-02 | Blue |
| WP_011607440.1 | FRAAL6395 | molybdenum cofactor biosynthesis protein MoaC | 19762 | 5.95 | 3 | 7 | Frankia-8 | F201371.dat | 53 | 10 | 2 | 2 | 3 | 0 | 0 | 0 | 0 | 0 | 0.000% | 1 | 0 | 3 | 1 | 2 | 0.012% | 2.40 | 0.88 | 2.5E-02 | Blue |
| WP_011605215.1 | FRAAL4085 | cytochrome P450 | 43949 | 5.26 | 4 | 7 | Frankia-8 | F201371.dat | 76 | 10 | 3 | 3 | 3 | 0 | 0 | 0 | 0 | 0 | 0.000% | 2 | 2 | 3 | 0 | 0 | 0.005% | 2.40 | 0.88 | 3.8E-02 | Blue |
| WP_041938913.1 | FRAAL1418 | F420-dependent oxidoreductase | 40100 | 5.63 | 12 | 85 | Frankia-9 | F201372.dat | 460 | 36 | 11 | 11 | 14 | 3 | 4 | 3 | 5 | 8 | 0.020% | 10 | 8 | 17 | 14 | 13 | 0.052% | 2.39 | 0.87 | 1.3E-03 | Blue |
| WP_011602282.1 | FRAAL1055 | orotate phosphoribosyltransferase | 18655 | 5.31 | 4 | 17 | Frankia-10 | F201373.dat | 138 | 31 | 4 | 4 | 4 | 1 | 0 | 0 | 1 | 1 | 0.006% | 2 | 2 | 4 | 2 | 4 | 0.025% | 2.38 | 0.86 | 2.9E-03 | Blue |
| WP_041938850.1 | FRAAL1097 | 50S ribosomal protein L18 | 13187 | 11.49 | 11 | 60 | Frankia-10 | F201373.dat | 364 | 60 | 7 | 7 | 10 | 3 | 3 | 3 | 3 | 4 | 0.043% | 7 | 8 | 9 | 10 | 10 | 0.112% | 2.33 | 0.85 | 1.6E-05 | Blue |
| WP_011604707.1 | FRAAL3566 | TetR family transcriptional regulator | 24406 | 6.45 | 4 | 20 | Frankia-9 | F201372.dat | 127 | 22 | 4 | 4 | 4 | 0 | 0 | 1 | 1 | 2 | 0.006% | 3 | 3 | 3 | 4 | 3 | 0.022% | 2.33 | 0.85 | 6.7E-04 | Blue |
| WP_011605869.1 | FRAAL4754 | hypothetical protein | 27960 | 5.93 | 5 | 10 | Frankia-10 | F201373.dat | 89 | 18 | 3 | 3 | 3 | 0 | 1 | 0 | 0 | 0 | 0.001% | 0 | 2 | 2 | 2 | 3 | 0.011% | 2.33 | 0.85 | 1.3E-02 | Blue |
| WP_011604716.1 | FRAAL3575 | hypothetical protein | 28008 | 6.30 | 4 | 10 | Frankia-9 | F201372.dat | 101 | 16 | 3 | 3 | 3 | 0 | 0 | 0 | 0 | 1 | 0.001% | 2 | 0 | 2 | 3 | 2 | 0.011% | 2.33 | 0.85 | 1.3E-02 | Blue |
| WP_041939858.1 | FRAAL6258 | acyl carrier protein | 8903 | 4.15 | 3 | 10 | Frankia-7 | F201370.dat | 153 | 40 | 3 | 3 | 3 | 0 | 0 | 1 | 0 | 0 | 0.004% | 1 | 3 | 1 | 3 | 1 | 0.034% | 2.33 | 0.85 | 1.3E-02 | Blue |
| WP_041939395.1 | FRAAL3816 | glyoxalase | 15394 | 5.40 | 4 | 10 | Frankia-8 | F201371.dat | 125 | 20 | 3 | 3 | 3 | 0 | 0 | 1 | 0 | 0 | 0.002% | 1 | 0 | 3 | 3 | 2 | 0.020% | 2.33 | 0.85 | 2.2E-02 | Blue |
| WP_041939018.1 | FRAAL1922 | radical SAM protein | 27231 | 10.47 | 5 | 13 | Frankia-8 | F201371.dat | 139 | 13 | 3 | 3 | 3 | 0 | 0 | 1 | 1 | 0 | 0.003% | 2 | 2 | 3 | 3 | 1 | 0.014% | 2.29 | 0.83 | 3.5E-03 | Blue |
| WP_011601970.1 | FRAAL0729 | branched-chain amino acid ABC transporter ATP-binding protein | 27720 | 6.54 | 3 | 6 | Frankia-8 | F201371.dat | 179 | 13 | 2 | 2 | 2 | 0 | 0 | 0 | 0 | 0 | 0.000% | 1 | 1 | 2 | 1 | 1 | 0.007% | 2.20 | 0.79 | 1.7E-02 | Blue |
| WP_011601300.1 | FRAAL0032 | nitrogen regulatory protein P-II 1 | 12090 | 6.27 | 2 | 6 | Frankia-8 | F201371.dat | 51 | 20 | 2 | 2 | 2 | 0 | 0 | 0 | 0 | 0 | 0.000% | 1 | 1 | 2 | 1 | 1 | 0.017% | 2.20 | 0.79 | 1.7E-02 | Blue |
| WP_041939795.1 | FRAAL5988 | arginine ABC transporter ATP-binding protein | 28161 | 9.88 | 4 | 6 | Frankia-10 | F201373.dat | 96 | 14 | 2 | 2 | 2 | 0 | 0 | 0 | 0 | 0 | 0.000% | 1 | 0 | 2 | 1 | 2 | 0.007% | 2.20 | 0.79 | 2.2E-02 | Blue |
| WP_011603065.1 | FRAAL1888 | glutamine amidotransferase | 29796 | 8.34 | 3 | 6 | Frankia-9 | F201372.dat | 131 | 12 | 2 | 2 | 2 | 0 | 0 | 0 | 0 | 0 | 0.000% | 1 | 1 | 2 | 2 | 0 | 0.007% | 2.20 | 0.79 | 2.2E-02 | Blue |
| WP_011603154.1 | FRAAL1981 | transcriptional regulator | 54853 | 7.20 | 3 | 6 | Frankia-9 | F201372.dat | 94 | 5 | 2 | 2 | 2 | 0 | 0 | 0 | 0 | 0 | 0.000% | 0 | 1 | 2 | 2 | 1 | 0.004% | 2.20 | 0.79 | 2.2E-02 | Blue |
| WP_041938764.1 | FRAAL0756 | C4-dicarboxylate ABC transporter substrate-binding protein | 41531 | 6.80 | 3 | 6 | Frankia-9 | F201372.dat | 61 | 5 | 2 | 2 | 2 | 0 | 0 | 0 | 0 | 0 | 0.000% | 1 | 2 | 0 | 2 | 1 | 0.005% | 2.20 | 0.79 | 2.2E-02 | Blue |
| WP_011607916.1 | FRAAL6881 | membrane protein | 48316 | 11.56 | 9 | 41 | Frankia-8 | F201371.dat | 314 | 24 | 8 | 8 | 9 | 3 | 2 | 4 | 0 | 2 | 0.008% | 8 | 2 | 9 | 6 | 5 | 0.021% | 2.19 | 0.78 | 1.3E-02 | Blue |
| WP_050997251.1 | FRAAL5858 | amino acid-binding protein | 24453 | 4.72 | 3 | 9 | Frankia-8 | F201371.dat | 190 | 14 | 3 | 3 | 3 | 0 | 1 | 0 | 0 | 0 | 0.001% | 1 | 1 | 3 | 1 | 2 | 0.011% | 2.17 | 0.77 | 1.4E-02 | Blue |
| WP_011603981.1 | FRAAL2830 | GntR family transcriptional regulator | 24588 | 7.16 | 2 | 9 | Frankia-6 | F201369.dat | 136 | 14 | 2 | 2 | 3 | 0 | 1 | 0 | 0 | 0 | 0.001% | 3 | 1 | 1 | 1 | 2 | 0.011% | 2.17 | 0.77 | 1.4E-02 | Blue |
| WP_011602479.1 | FRAAL1279 | two-component system sensor histidine kinase/response regulator | 64971 | 4.90 | 5 | 9 | Frankia-10 | F201373.dat | 149 | 6 | 3 | 3 | 3 | 0 | 0 | 0 | 1 | 0 | 0.001% | 1 | 2 | 2 | 0 | 3 | 0.004% | 2.17 | 0.77 | 2.5E-02 | Blue |
| WP_011607074.1 | FRAAL6022 | gamma-aminobutyraldehyde dehydrogenase | 50016 | 4.90 | 4 | 9 | Frankia-8 | F201371.dat | 119 | 8 | 3 | 3 | 3 | 0 | 0 | 0 | 0 | 1 | 0.001% | 0 | 2 | 3 | 2 | 1 | 0.005% | 2.17 | 0.77 | 2.5E-02 | Blue |
| WP_011606474.1 | FRAAL5391 | hypothetical protein | 24339 | 7.00 | 2 | 9 | Frankia-10 | F201373.dat | 132 | 11 | 2 | 2 | 2 | 0 | 0 | 0 | 1 | 0 | 0.001% | 0 | 2 | 1 | 3 | 2 | 0.011% | 2.17 | 0.77 | 2.5E-02 | Blue |
| WP_011605682.1 | FRAAL4565 | hypothetical protein | 24773 | 9.44 | 9 | 31 | Frankia-7 | F201370.dat | 325 | 28 | 6 | 6 | 7 | 1 | 2 | 2 | 1 | 2 | 0.012% | 4 | 7 | 6 | 4 | 2 | 0.031% | 2.15 | 0.77 | 6.1E-03 | Blue |
| WP_041938873.1 | FRAAL1197 | deoxyribose-phosphate aldolase | 26667 | 5.31 | 7 | 31 | Frankia-8 | F201371.dat | 186 | 27 | 5 | 5 | 7 | 2 | 0 | 0 | 2 | 4 | 0.011% | 2 | 3 | 7 | 6 | 5 | 0.029% | 2.15 | 0.77 | 1.8E-02 | Blue |
| WP_041938866.1 | FRAAL1164 | hypothetical protein | 11331 | 5.55 | 9 | 116 | Frankia-7 | F201370.dat | 339 | 77 | 9 | 9 | 14 | 3 | 7 | 8 | 10 | 7 | 0.110% | 13 | 14 | 17 | 20 | 17 | 0.241% | 2.15 | 0.77 | 3.0E-04 | Blue |
| WP_050997130.1 | FRAAL3428 | TetR family transcriptional regulator | 26769 | 8.14 | 3 | 12 | Frankia-9 | F201372.dat | 162 | 16 | 3 | 3 | 3 | 0 | 0 | 1 | 0 | 1 | 0.003% | 1 | 3 | 2 | 3 | 1 | 0.013% | 2.14 | 0.76 | 1.0E-02 | Blue |
| WP_011605365.1 | FRAAL4239 | hypothetical protein | 21567 | 10.07 | 11 | 62 | Frankia-7 | F201370.dat | 385 | 52 | 9 | 9 | 10 | 2 | 5 | 3 | 3 | 5 | 0.030% | 6 | 10 | 9 | 9 | 10 | 0.069% | 2.13 | 0.76 | 2.9E-04 | Blue |
| WP_011602951.1 | FRAAL1768 | transcriptional regulator | 23932 | 9.56 | 3 | 15 | Frankia-6 | F201369.dat | 125 | 24 | 3 | 3 | 3 | 1 | 1 | 1 | 0 | 0 | 0.004% | 3 | 3 | 2 | 2 | 2 | 0.017% | 2.13 | 0.75 | 2.5E-03 | Blue |
| WP_011606087.1 | FRAAL4973 | phosphoribosyl-AMP cyclohydrolase | 14318 | 6.03 | 5 | 18 | Frankia-9 | F201372.dat | 131 | 30 | 3 | 3 | 3 | 1 | 2 | 0 | 0 | 1 | 0.010% | 3 | 3 | 3 | 3 | 2 | 0.033% | 2.11 | 0.75 | 2.0E-03 | Blue |
| WP_011606325.1 | FRAAL5232 | hypothetical protein | 12140 | 10.15 | 13 | 220 | Frankia-10 | F201373.dat | 645 | 74 | 12 | 12 | 35 | 10 | 14 | 14 | 15 | 16 | 0.203% | 27 | 25 | 31 | 33 | 35 | 0.419% | 2.11 | 0.75 | 2.7E-05 | Blue |
| WP_011602312.1 | FRAAL1099 | 50S ribosomal protein L30 | 6891 | 11.64 | 7 | 52 | Frankia-9 | F201372.dat | 222 | 62 | 7 | 7 | 11 | 2 | 3 | 3 | 3 | 4 | 0.078% | 5 | 3 | 8 | 11 | 10 | 0.181% | 2.10 | 0.74 | 1.1E-02 | Blue |
| WP_041939818.1 | FRAAL6056 | ATP-binding protein | 7646 | 4.93 | 3 | 30 | Frankia-9 | F201372.dat | 170 | 47 | 3 | 3 | 6 | 2 | 2 | 1 | 1 | 2 | 0.037% | 4 | 3 | 4 | 6 | 5 | 0.097% | 2.08 | 0.73 | 8.7E-04 | Blue |
| WP_011603450.1 | FRAAL2285 | phosphohistidine phosphatase | 19422 | 5.94 | 6 | 33 | Frankia-10 | F201373.dat | 382 | 44 | 5 | 5 | 6 | 1 | 2 | 2 | 4 | 0 | 0.017% | 4 | 5 | 4 | 5 | 6 | 0.042% | 2.07 | 0.73 | 2.2E-03 | Blue |
| WP_041939066.1 | FRAAL2163 | 5.10-methylenetetrahydrofolate reductase | 32045 | 7.07 | 9 | 45 | Frankia-6 | F201369.dat | 268 | 28 | 6 | 6 | 7 | 2 | 2 | 1 | 4 | 4 | 0.014% | 7 | 7 | 6 | 6 | 6 | 0.034% | 2.06 | 0.72 | 2.7E-04 | Blue |
| WP_011603972.1 | FRAAL2821 | peptide-binding protein | 63379 | 8.31 | 25 | 181 | Frankia-7 | F201370.dat | 1084 | 44 | 19 | 19 | 27 | 12 | 11 | 11 | 12 | 12 | 0.033% | 23 | 27 | 22 | 25 | 26 | 0.065% | 2.03 | 0.71 | 5.2E-07 | Blue |
| WP_041938959.1 | FRAAL1649 | hypothetical protein | 12234 | 6.71 | 5 | 65 | Frankia-10 | F201373.dat | 231 | 30 | 5 | 5 | 11 | 4 | 4 | 4 | 4 | 4 | 0.058% | 7 | 8 | 8 | 11 | 11 | 0.124% | 2.00 | 0.69 | 2.7E-04 | Blue |
| WP_041939430.1 | FRAAL3977 | TetR family transcriptional regulator | 27204 | 5.52 | 5 | 20 | Frankia-8 | F201371.dat | 171 | 21 | 4 | 4 | 4 | 1 | 1 | 1 | 1 | 1 | 0.007% | 3 | 2 | 4 | 3 | 3 | 0.019% | 2.00 | 0.69 | 1.4E-03 | Blue |
| WP_011601656.1 | FRAAL0400 | hypothetical protein | 24128 | 5.23 | 4 | 23 | Frankia-8 | F201371.dat | 173 | 29 | 4 | 4 | 4 | 1 | 0 | 1 | 2 | 2 | 0.009% | 2 | 4 | 4 | 3 | 4 | 0.024% | 2.00 | 0.69 | 1.9E-03 | Blue |
| WP_011607871.1 | FRAAL6836 | molybdopterin oxidoreductase | 12278 | 5.53 | 3 | 17 | Frankia-8 | F201371.dat | 147 | 29 | 3 | 3 | 3 | 1 | 1 | 0 | 1 | 1 | 0.012% | 2 | 2 | 3 | 3 | 3 | 0.036% | 2.00 | 0.69 | 2.5E-03 | Blue |
| WP_041941123.1 | FRAAL6560 | hypothetical protein | 12628 | 6.23 | 3 | 29 | Frankia-9 | F201372.dat | 186 | 25 | 3 | 3 | 4 | 1 | 1 | 4 | 1 | 1 | 0.023% | 4 | 4 | 4 | 4 | 5 | 0.056% | 2.00 | 0.69 | 2.6E-03 | Blue |
| WP_011601356.1 | FRAAL0089 | LuxR family transcriptional regulator | 23079 | 6.53 | 6 | 20 | Frankia-10 | F201373.dat | 236 | 31 | 4 | 4 | 4 | 1 | 1 | 2 | 0 | 1 | 0.008% | 2 | 2 | 3 | 4 | 4 | 0.022% | 2.00 | 0.69 | 3.5E-03 | Blue |
| WP_011601894.1 | FRAAL0646 | hypothetical protein | 65090 | 5.18 | 10 | 53 | Frankia-10 | F201373.dat | 411 | 17 | 9 | 9 | 11 | 4 | 3 | 2 | 3 | 4 | 0.009% | 6 | 6 | 9 | 5 | 11 | 0.019% | 2.00 | 0.69 | 3.8E-03 | Blue |
| WP_011602699.1 | FRAAL1509 | oxidoreductase | 29983 | 5.32 | 5 | 17 | Frankia-8 | F201371.dat | 169 | 14 | 4 | 4 | 4 | 0 | 1 | 0 | 1 | 2 | 0.005% | 2 | 3 | 4 | 2 | 2 | 0.015% | 2.00 | 0.69 | 5.5E-03 | Blue |
| WP_041938800.1 | FRAAL0887 | deaminase/reductase | 23115 | 6.38 | 3 | 14 | Frankia-10 | F201373.dat | 96 | 25 | 3 | 3 | 3 | 0 | 1 | 0 | 0 | 2 | 0.005% | 2 | 2 | 2 | 2 | 3 | 0.016% | 2.00 | 0.69 | 7.5E-03 | Blue |
| WP_050997327.1 | FRAAL1386 | ketosteroid isomerase | 16846 | 5.71 | 4 | 20 | Frankia-10 | F201373.dat | 113 | 39 | 4 | 4 | 4 | 0 | 2 | 1 | 0 | 2 | 0.011% | 1 | 2 | 4 | 4 | 4 | 0.030% | 2.00 | 0.69 | 1.6E-02 | Blue |
| WP_041940093.1 | FRAAL0558 | Fe-S cluster assembly protein HesB | 20292 | 6.31 | 3 | 11 | Frankia-8 | F201371.dat | 89 | 11 | 2 | 2 | 3 | 1 | 1 | 0 | 0 | 0 | 0.004% | 0 | 2 | 3 | 2 | 2 | 0.015% | 2.00 | 0.69 | 2.3E-02 | Blue |
| WP_050997141.1 | FRAAL3618 | oxidoreductase | 24529 | 5.57 | 2 | 5 | Frankia-8 | F201371.dat | 117 | 14 | 2 | 2 | 2 | 0 | 0 | 0 | 0 | 0 | 0.000% | 0 | 1 | 2 | 1 | 1 | 0.007% | 2.00 | 0.69 | 3.3E-02 | Blue |
| WP_011605258.1 | FRAAL4130 | hypothetical protein | 29230 | 6.14 | 2 | 5 | Frankia-7 | F201370.dat | 95 | 10 | 2 | 2 | 2 | 0 | 0 | 0 | 0 | 0 | 0.000% | 1 | 2 | 0 | 1 | 1 | 0.006% | 2.00 | 0.69 | 3.3E-02 | Blue |
| WP_011602209.1 | FRAAL0982 | REX family transcriptional regulator | 38518 | 8.78 | 2 | 5 | Frankia-10 | F201373.dat | 87 | 7 | 2 | 2 | 2 | 0 | 0 | 0 | 0 | 0 | 0.000% | 1 | 0 | 1 | 1 | 2 | 0.004% | 2.00 | 0.69 | 3.3E-02 | Blue |
| WP_011602153.1 | FRAAL0923 | cupin | 12974 | 4.76 | 2 | 5 | Frankia-10 | F201373.dat | 81 | 33 | 2 | 2 | 2 | 0 | 0 | 0 | 0 | 0 | 0.000% | 0 | 1 | 1 | 1 | 2 | 0.013% | 2.00 | 0.69 | 3.3E-02 | Blue |
| WP_041939553.1 | FRAAL4675 | acyl-CoA transferase | 82053 | 5.32 | 5 | 14 | Frankia-8 | F201371.dat | 155 | 5 | 3 | 3 | 4 | 1 | 0 | 2 | 0 | 0 | 0.001% | 0 | 2 | 4 | 3 | 2 | 0.005% | 2.00 | 0.69 | 3.6E-02 | Blue |
| WP_011602611.1 | FRAAL1416 | phenylhydantoinase | 51219 | 5.58 | 17 | 103 | Frankia-8 | F201371.dat | 587 | 38 | 14 | 14 | 23 | 6 | 7 | 7 | 6 | 7 | 0.023% | 8 | 12 | 23 | 12 | 15 | 0.046% | 1.97 | 0.68 | 9.6E-03 | Blue |
| WP_041938614.1 | FRAAL0115 | MarR family transcriptional regulator | 15617 | 8.54 | 7 | 40 | Frankia-9 | F201372.dat | 464 | 66 | 7 | 7 | 7 | 0 | 3 | 3 | 3 | 3 | 0.027% | 5 | 4 | 6 | 7 | 6 | 0.060% | 1.94 | 0.66 | 1.8E-03 | Blue |
| WP_041938998.1 | FRAAL1814 | histidine kinase | 50249 | 7.27 | 6 | 34 | Frankia-7 | F201370.dat | 245 | 19 | 6 | 6 | 6 | 2 | 2 | 2 | 2 | 2 | 0.007% | 3 | 6 | 5 | 5 | 5 | 0.016% | 1.93 | 0.66 | 7.4E-04 | Blue |
| WP_041938995.1 | FRAAL1804 | hypothetical protein | 24462 | 6.31 | 6 | 31 | Frankia-7 | F201370.dat | 217 | 26 | 5 | 5 | 5 | 2 | 2 | 1 | 2 | 2 | 0.013% | 3 | 5 | 5 | 4 | 5 | 0.030% | 1.93 | 0.66 | 5.2E-04 | Blue |
| WP_011606308.1 | FRAAL5214 | 50S ribosomal protein L20 | 14142 | 11.25 | 10 | 92 | Frankia-9 | F201372.dat | 404 | 61 | 9 | 9 | 13 | 6 | 6 | 7 | 6 | 5 | 0.076% | 9 | 11 | 13 | 13 | 16 | 0.148% | 1.91 | 0.65 | 3.7E-04 | Blue |
| WP_041939651.1 | FRAAL5155 | peptidase | 25914 | 5.70 | 5 | 22 | Frankia-7 | F201370.dat | 213 | 24 | 4 | 4 | 4 | 0 | 2 | 2 | 1 | 1 | 0.008% | 3 | 4 | 4 | 3 | 2 | 0.021% | 1.91 | 0.65 | 2.7E-03 | Blue |
| WP_011604890.1 | FRAAL3751 | PPOX class F420-dependent enzyme | 15243 | 6.12 | 5 | 22 | Frankia-8 | F201371.dat | 119 | 44 | 4 | 4 | 4 | 1 | 0 | 2 | 1 | 2 | 0.014% | 3 | 3 | 4 | 2 | 4 | 0.035% | 1.91 | 0.65 | 2.7E-03 | Blue |
| WP_011601292.1 | FRAAL0024 | type 11 methyltransferase | 26664 | 6.79 | 5 | 22 | Frankia-9 | F201372.dat | 160 | 19 | 4 | 4 | 4 | 0 | 1 | 2 | 0 | 3 | 0.008% | 2 | 3 | 4 | 4 | 3 | 0.020% | 1.91 | 0.65 | 1.0E-02 | Blue |
| WP_011602164.1 | FRAAL0934 | anti-sigma factor antagonist | 12743 | 5.73 | 3 | 19 | Frankia-8 | F201371.dat | 152 | 35 | 3 | 3 | 3 | 0 | 1 | 2 | 2 | 0 | 0.014% | 2 | 2 | 3 | 4 | 3 | 0.037% | 1.90 | 0.64 | 7.5E-03 | Blue |
| WP_009740524.1 | FRAAL1085 | 30S ribosomal protein S19 | 10598 | 11.00 | 14 | 106 | Frankia-8 | F201371.dat | 486 | 66 | 10 | 10 | 18 | 5 | 5 | 5 | 9 | 11 | 0.118% | 9 | 8 | 18 | 19 | 17 | 0.226% | 1.90 | 0.64 | 1.4E-02 | Blue |
| WP_011603564.1 | FRAAL2404 | hypothetical protein | 27458 | 6.14 | 4 | 16 | Frankia-10 | F201373.dat | 184 | 18 | 4 | 4 | 4 | 1 | 0 | 1 | 1 | 1 | 0.005% | 2 | 2 | 2 | 2 | 4 | 0.015% | 1.89 | 0.64 | 7.5E-03 | Blue |
| WP_011602304.1 | FRAAL1089 | 50S ribosomal protein L29 | 9844 | 5.13 | 6 | 42 | Frankia-9 | F201372.dat | 402 | 70 | 6 | 6 | 9 | 2 | 3 | 4 | 2 | 2 | 0.047% | 5 | 4 | 4 | 9 | 7 | 0.099% | 1.89 | 0.64 | 7.9E-03 | Blue |
| WP_011606740.1 | FRAAL5664 | protein-tyrosine phosphatase | 27409 | 5.58 | 5 | 39 | Frankia-9 | F201372.dat | 217 | 25 | 5 | 5 | 8 | 2 | 1 | 4 | 4 | 1 | 0.016% | 6 | 4 | 3 | 8 | 6 | 0.033% | 1.88 | 0.63 | 1.3E-02 | Blue |
| WP_041939917.1 | FRAAL6545 | glutamyl-tRNA amidotransferase | 16735 | 8.01 | 7 | 39 | Frankia-10 | F201373.dat | 362 | 57 | 7 | 7 | 9 | 2 | 2 | 2 | 3 | 3 | 0.026% | 3 | 2 | 5 | 8 | 9 | 0.054% | 1.88 | 0.63 | 3.3E-02 | Blue |
| WP_041940722.1 | FRAAL4251 | ABC transporter ATP-binding protein | 29091 | 5.71 | 8 | 33 | Frankia-7 | F201370.dat | 210 | 25 | 6 | 6 | 6 | 1 | 2 | 3 | 2 | 2 | 0.012% | 4 | 6 | 5 | 3 | 5 | 0.027% | 1.87 | 0.62 | 1.3E-03 | Blue |
| WP_041939940.1 | FRAAL6657 | transglycosylase | 23166 | 10.14 | 5 | 44 | Frankia-5 | F201368.dat | 195 | 32 | 4 | 4 | 5 | 0 | 3 | 3 | 3 | 5 | 0.022% | 4 | 2 | 6 | 10 | 8 | 0.044% | 1.84 | 0.61 | 4.2E-02 | Blue |
| WP_041939021.1 | FRAAL1928 | gamma-glutamyl kinase | 38082 | 6.67 | 9 | 41 | Frankia-7 | F201370.dat | 351 | 26 | 6 | 6 | 6 | 3 | 3 | 3 | 3 | 1 | 0.012% | 5 | 6 | 6 | 6 | 5 | 0.025% | 1.83 | 0.61 | 2.1E-04 | Blue |
| WP_041939577.1 | FRAAL4844 | methyltransferase | 30334 | 5.59 | 2 | 7 | Frankia-10 | F201373.dat | 210 | 20 | 4 | 2 | 1 | 0 | 0 | 0 | 1 | 0 | 0.001% | 2 | 1 | 1 | 1 | 1 | 0.007% | 1.83 | 0.61 | 3.3E-02 | Blue |
| WP_011606309.1 | FRAAL5215 | 50S ribosomal protein L35 | 7281 | 12.31 | 2 | 7 | Frankia-8 | F201371.dat | 57 | 16 | 2 | 2 | 2 | 0 | 1 | 0 | 0 | 0 | 0.005% | 1 | 1 | 2 | 1 | 1 | 0.028% | 1.83 | 0.61 | 3.3E-02 | Blue |
| WP_041939810.1 | FRAAL6034 | ABC transporter | 67639 | 9.19 | 3 | 7 | Frankia-8 | F201371.dat | 110 | 5 | 2 | 2 | 2 | 1 | 0 | 0 | 0 | 0 | 0.001% | 0 | 2 | 2 | 1 | 1 | 0.003% | 1.83 | 0.61 | 4.0E-02 | Blue |
| WP_011604851.1 | FRAAL3712 | DSBA oxidoreductase | 24072 | 6.45 | 2 | 7 | Frankia-8 | F201371.dat | 125 | 13 | 2 | 2 | 2 | 0 | 0 | 0 | 0 | 1 | 0.001% | 0 | 1 | 2 | 2 | 1 | 0.008% | 1.83 | 0.61 | 4.0E-02 | Blue |
| WP_011605301.1 | FRAAL4173 | deaminase reductase | 20735 | 4.97 | 10 | 55 | Frankia-8 | F201371.dat | 456 | 52 | 9 | 9 | 10 | 3 | 3 | 3 | 6 | 3 | 0.031% | 7 | 5 | 10 | 7 | 8 | 0.060% | 1.83 | 0.60 | 2.8E-03 | Blue |
| WP_041939378.1 | FRAAL3686 | methylenetetrahydrofolate reductase | 29021 | 5.91 | 10 | 55 | Frankia-9 | F201372.dat | 355 | 46 | 9 | 9 | 9 | 5 | 7 | 3 | 3 | 0 | 0.022% | 5 | 6 | 9 | 9 | 8 | 0.043% | 1.83 | 0.60 | 1.4E-02 | Blue |
| WP_011606457.1 | FRAAL5373 | regulator | 16023 | 6.41 | 3 | 21 | Frankia-8 | F201371.dat | 102 | 15 | 3 | 3 | 4 | 1 | 1 | 1 | 2 | 1 | 0.013% | 2 | 3 | 4 | 3 | 3 | 0.032% | 1.82 | 0.60 | 2.5E-03 | Blue |
| WP_041940275.1 | FRAAL1744 | phospholipase D | 59341 | 9.05 | 5 | 21 | Frankia-7 | F201370.dat | 116 | 11 | 4 | 4 | 4 | 1 | 2 | 1 | 2 | 0 | 0.004% | 3 | 4 | 4 | 2 | 2 | 0.009% | 1.82 | 0.60 | 7.5E-03 | Blue |
| WP_011605640.1 | FRAAL4521 | pyruvate dehydrogenase | 106435 | 5.46 | 6 | 21 | Frankia-8 | F201371.dat | 283 | 5 | 4 | 4 | 4 | 1 | 2 | 1 | 1 | 1 | 0.002% | 4 | 1 | 4 | 3 | 3 | 0.005% | 1.82 | 0.60 | 1.1E-02 | Blue |
| WP_011606263.1 | FRAAL5159 | membrane protein | 31144 | 11.89 | 9 | 46 | Frankia-6 | F201369.dat | 354 | 27 | 7 | 7 | 7 | 3 | 3 | 3 | 3 | 3 | 0.017% | 7 | 5 | 6 | 7 | 6 | 0.034% | 1.80 | 0.59 | 1.0E-04 | Blue |
| WP_009740525.1 | FRAAL1084 | 50S ribosomal protein L2 | 30314 | 11.42 | 20 | 186 | Frankia-8 | F201371.dat | 942 | 64 | 19 | 19 | 31 | 12 | 14 | 11 | 10 | 18 | 0.077% | 25 | 21 | 31 | 24 | 20 | 0.134% | 1.80 | 0.59 | 8.0E-04 | Blue |
| WP_041939543.1 | FRAAL4608 | dephospho-CoA kinase | 22101 | 5.16 | 3 | 18 | Frankia-8 | F201371.dat | 134 | 15 | 3 | 3 | 3 | 1 | 1 | 1 | 2 | 0 | 0.008% | 2 | 3 | 3 | 3 | 2 | 0.020% | 1.80 | 0.59 | 4.6E-03 | Blue |
| WP_041939399.1 | FRAAL3835 | molybdate-binding protein | 26718 | 9.06 | 9 | 85 | Frankia-10 | F201373.dat | 467 | 42 | 8 | 8 | 11 | 5 | 6 | 7 | 5 | 6 | 0.039% | 7 | 13 | 12 | 13 | 11 | 0.071% | 1.79 | 0.58 | 8.8E-04 | Blue |
| WP_011603657.1 | FRAAL2500 | ketosteroid isomerase | 29555 | 5.75 | 7 | 43 | Frankia-10 | F201373.dat | 317 | 28 | 6 | 6 | 7 | 3 | 2 | 2 | 3 | 4 | 0.017% | 5 | 5 | 7 | 5 | 7 | 0.033% | 1.79 | 0.58 | 6.2E-04 | Blue |
| WP_050997456.1 | FRAAL6068 | chorismate mutase | 11233 | 5.33 | 3 | 15 | Frankia-6 | F201369.dat | 181 | 42 | 3 | 3 | 3 | 0 | 0 | 1 | 2 | 1 | 0.013% | 3 | 2 | 2 | 2 | 2 | 0.033% | 1.78 | 0.58 | 1.2E-02 | Blue |
| WP_050997205.1 | FRAAL4843 | mechanosensitive ion channel protein MscL | 19048 | 8.10 | 3 | 15 | Frankia-10 | F201373.dat | 115 | 18 | 3 | 3 | 3 | 1 | 1 | 1 | 1 | 0 | 0.007% | 2 | 1 | 3 | 2 | 3 | 0.019% | 1.78 | 0.58 | 1.2E-02 | Blue |
| WP_041939184.1 | FRAAL2692 | hypothetical protein | 25642 | 9.87 | 4 | 15 | Frankia-7 | F201370.dat | 176 | 24 | 4 | 4 | 4 | 0 | 1 | 1 | 2 | 0 | 0.006% | 2 | 4 | 1 | 2 | 2 | 0.014% | 1.78 | 0.58 | 2.6E-02 | Blue |
| WP_041938598.1 | FRAAL0048 | twin-arginine translocation pathway signal protein | 53942 | 8.16 | 7 | 26 | Frankia-7 | F201370.dat | 201 | 13 | 4 | 4 | 5 | 2 | 2 | 2 | 1 | 1 | 0.005% | 4 | 5 | 3 | 4 | 2 | 0.011% | 1.77 | 0.57 | 5.5E-03 | Blue |
| WP_011607460.1 | FRAAL6415 | serine phosphatase | 72686 | 5.01 | 6 | 26 | Frankia-6 | F201369.dat | 164 | 8 | 4 | 4 | 4 | 4 | 1 | 2 | 1 | 0 | 0.004% | 4 | 5 | 2 | 4 | 3 | 0.008% | 1.77 | 0.57 | 2.3E-02 | Blue |
| WP_011602637.1 | FRAAL1447 | urease subunit alpha | 60154 | 5.28 | 13 | 59 | Frankia-8 | F201371.dat | 579 | 28 | 10 | 10 | 12 | 4 | 5 | 2 | 5 | 4 | 0.012% | 6 | 8 | 12 | 5 | 8 | 0.022% | 1.76 | 0.57 | 1.0E-02 | Blue |
| WP_011604051.1 | FRAAL2905 | F420-dependent oxidoreductase | 28699 | 4.57 | 6 | 23 | Frankia-8 | F201371.dat | 174 | 21 | 5 | 5 | 5 | 2 | 1 | 2 | 0 | 2 | 0.009% | 3 | 3 | 5 | 2 | 3 | 0.019% | 1.75 | 0.56 | 1.1E-02 | Blue |
| WP_041939363.1 | FRAAL3585 | esterase | 26595 | 7.93 | 3 | 12 | Frankia-10 | F201373.dat | 158 | 11 | 3 | 3 | 3 | 0 | 1 | 0 | 0 | 2 | 0.004% | 1 | 1 | 2 | 2 | 3 | 0.011% | 1.75 | 0.56 | 3.0E-02 | Blue |
| WP_050997024.1 | FRAAL1457 | molybdopterin dehydrogenase | 31075 | 9.55 | 3 | 12 | Frankia-7 | F201370.dat | 117 | 8 | 2 | 2 | 2 | 0 | 0 | 0 | 1 | 2 | 0.003% | 1 | 2 | 3 | 1 | 2 | 0.010% | 1.75 | 0.56 | 3.0E-02 | Blue |
| WP_009741405.1 | FRAAL0068 | hypothetical protein | 8652 | 4.95 | 2 | 12 | Frankia-9 | F201372.dat | 107 | 26 | 2 | 2 | 3 | 0 | 2 | 0 | 1 | 0 | 0.012% | 2 | 1 | 2 | 3 | 1 | 0.035% | 1.75 | 0.56 | 3.0E-02 | Blue |
| WP_011604910.1 | FRAAL3772 | hypothetical protein | 14517 | 11.88 | 3 | 31 | Frankia-9 | F201372.dat | 156 | 30 | 3 | 3 | 5 | 1 | 3 | 3 | 1 | 2 | 0.025% | 3 | 5 | 3 | 5 | 5 | 0.049% | 1.73 | 0.55 | 5.3E-03 | Blue |
| WP_041939661.1 | FRAAL5219 | 6.7-dimethyl-8-ribityllumazine synthase | 16376 | 5.52 | 7 | 31 | Frankia-10 | F201373.dat | 274 | 47 | 6 | 6 | 6 | 1 | 3 | 2 | 2 | 2 | 0.022% | 2 | 4 | 5 | 4 | 6 | 0.043% | 1.73 | 0.55 | 9.0E-03 | Blue |
| WP_041941162.1 | FRAAL6875 | GCN5 family acetyltransferase | 23388 | 5.80 | 4 | 20 | Frankia-7 | F201370.dat | 180 | 16 | 3 | 3 | 4 | 1 | 1 | 2 | 0 | 2 | 0.009% | 2 | 4 | 2 | 4 | 2 | 0.020% | 1.73 | 0.55 | 1.6E-02 | Blue |
| WP_041941067.1 | FRAAL6248 | methyltransferase | 23509 | 5.80 | 4 | 20 | Frankia-8 | F201371.dat | 147 | 16 | 3 | 3 | 4 | 2 | 0 | 0 | 2 | 2 | 0.009% | 3 | 3 | 4 | 2 | 2 | 0.020% | 1.73 | 0.55 | 1.6E-02 | Blue |
| WP_011604275.1 | FRAAL3126 | GntR family transcriptional regulator | 28282 | 6.32 | 13 | 77 | Frankia-8 | F201371.dat | 462 | 31 | 11 | 11 | 13 | 3 | 4 | 6 | 7 | 7 | 0.034% | 9 | 9 | 13 | 10 | 9 | 0.060% | 1.72 | 0.54 | 1.7E-03 | Blue |
| WP_011607495.1 | FRAAL6450 | short-chain dehydrogenase | 25898 | 5.85 | 5 | 28 | Frankia-6 | F201369.dat | 240 | 22 | 4 | 4 | 4 | 2 | 2 | 2 | 2 | 1 | 0.012% | 4 | 4 | 3 | 4 | 4 | 0.025% | 1.71 | 0.54 | 1.4E-03 | Blue |
| WP_011606117.1 | FRAAL5006 | methyltransferase | 65640 | 6.38 | 3 | 9 | Frankia-9 | F201372.dat | 121 | 4 | 2 | 2 | 2 | 1 | 0 | 1 | 0 | 0 | 0.001% | 1 | 1 | 2 | 2 | 1 | 0.004% | 1.71 | 0.54 | 3.3E-02 | Blue |
| WP_011602371.1 | FRAAL1169 | geranylgeranyl reductase | 46675 | 10.42 | 3 | 9 | Frankia-9 | F201372.dat | 69 | 3 | 2 | 2 | 2 | 1 | 0 | 0 | 1 | 0 | 0.002% | 1 | 2 | 1 | 2 | 1 | 0.005% | 1.71 | 0.54 | 3.3E-02 | Blue |
| WP_011606977.1 | FRAAL5916 | hypothetical protein | 20487 | 11.18 | 7 | 36 | Frankia-8 | F201371.dat | 312 | 37 | 6 | 6 | 8 | 2 | 2 | 3 | 2 | 3 | 0.021% | 3 | 5 | 8 | 4 | 4 | 0.039% | 1.71 | 0.53 | 1.6E-02 | Blue |
| WP_041938908.1 | FRAAL1410 | acireductone dioxygenase | 22156 | 4.77 | 4 | 17 | Frankia-10 | F201373.dat | 153 | 17 | 3 | 3 | 3 | 1 | 1 | 1 | 1 | 1 | 0.008% | 2 | 2 | 3 | 2 | 3 | 0.018% | 1.70 | 0.53 | 8.7E-03 | Blue |
| WP_041939138.1 | FRAAL2477 | PPOX class F420-dependent enzyme | 14823 | 8.20 | 7 | 57 | Frankia-8 | F201371.dat | 313 | 54 | 7 | 5 | 9 | 3 | 4 | 4 | 4 | 5 | 0.048% | 6 | 6 | 9 | 7 | 9 | 0.084% | 1.68 | 0.52 | 9.9E-04 | Blue |
| WP_011602135.1 | FRAAL0904 | nitrogen regulatory protein P-II 1 | 12114 | 6.09 | 9 | 89 | Frankia-8 | F201371.dat | 390 | 85 | 8 | 8 | 13 | 5 | 6 | 5 | 8 | 8 | 0.094% | 10 | 10 | 13 | 12 | 12 | 0.158% | 1.68 | 0.52 | 2.8E-04 | Blue |
| WP_009739724.1 | FRAAL2022 | membrane protein | 20364 | 7.99 | 9 | 113 | Frankia-10 | F201373.dat | 332 | 51 | 9 | 9 | 16 | 8 | 8 | 8 | 9 | 8 | 0.072% | 10 | 15 | 15 | 16 | 16 | 0.119% | 1.67 | 0.52 | 3.7E-04 | Blue |
| WP_011602184.1 | FRAAL0955 | hypothetical protein | 19461 | 11.58 | 12 | 121 | Frankia-9 | F201372.dat | 583 | 60 | 10 | 10 | 19 | 4 | 8 | 9 | 9 | 14 | 0.081% | 15 | 8 | 18 | 19 | 17 | 0.133% | 1.67 | 0.51 | 1.6E-02 | Blue |
| WP_050997426.1 | FRAAL4915 | GntR family transcriptional regulator | 29015 | 6.30 | 8 | 70 | Frankia-9 | F201372.dat | 383 | 39 | 8 | 8 | 11 | 5 | 5 | 5 | 5 | 5 | 0.031% | 7 | 10 | 9 | 11 | 8 | 0.052% | 1.67 | 0.51 | 4.5E-04 | Blue |
| WP_011603476.1 | FRAAL2312 | 4'-phosphopantetheinyl transferase | 25269 | 5.33 | 5 | 30 | Frankia-8 | F201371.dat | 262 | 31 | 5 | 5 | 6 | 3 | 3 | 1 | 1 | 2 | 0.014% | 2 | 4 | 6 | 4 | 4 | 0.027% | 1.67 | 0.51 | 1.6E-02 | Blue |
| WP_011601990.1 | FRAAL0749 | flagellar motor protein MotB | 19422 | 5.33 | 2 | 14 | Frankia-6 | F201369.dat | 115 | 17 | 2 | 2 | 2 | 1 | 1 | 1 | 0 | 1 | 0.007% | 2 | 2 | 2 | 1 | 3 | 0.017% | 1.67 | 0.51 | 1.7E-02 | Blue |
| WP_041941141.1 | FRAAL6699 | haloacid dehalogenase | 28002 | 5.10 | 5 | 22 | Frankia-8 | F201371.dat | 184 | 24 | 5 | 5 | 5 | 1 | 1 | 2 | 1 | 2 | 0.009% | 2 | 3 | 5 | 3 | 2 | 0.018% | 1.67 | 0.51 | 1.8E-02 | Blue |
| WP_041939398.1 | FRAAL3827 | branched-chain amino acid ABC transporter ATP-binding protein | 23366 | 9.17 | 3 | 14 | Frankia-9 | F201372.dat | 192 | 20 | 3 | 3 | 3 | 1 | 0 | 2 | 1 | 0 | 0.006% | 2 | 1 | 2 | 3 | 2 | 0.014% | 1.67 | 0.51 | 2.2E-02 | Blue |
| WP_011605596.1 | FRAAL4475 | hypothetical protein | 37727 | 5.86 | 3 | 14 | Frankia-9 | F201372.dat | 219 | 14 | 3 | 3 | 3 | 1 | 0 | 2 | 0 | 1 | 0.004% | 2 | 3 | 1 | 3 | 1 | 0.009% | 1.67 | 0.51 | 3.7E-02 | Blue |
| WP_011601765.1 | FRAAL0512 | hypothetical protein | 37317 | 5.89 | 21 | 136 | Frankia-9 | F201372.dat | 981 | 52 | 16 | 16 | 21 | 7 | 9 | 10 | 11 | 13 | 0.048% | 12 | 21 | 14 | 21 | 18 | 0.078% | 1.65 | 0.50 | 4.3E-03 | Blue |
| WP_041940330.1 | FRAAL2085 | potassium transporter TrkA | 23366 | 7.85 | 9 | 51 | Frankia-7 | F201370.dat | 353 | 40 | 7 | 7 | 9 | 4 | 4 | 3 | 4 | 3 | 0.027% | 5 | 9 | 7 | 6 | 6 | 0.048% | 1.65 | 0.50 | 2.0E-03 | Blue |
| WP_011604027.1 | FRAAL2881 | transcriptional regulator | 35519 | 6.09 | 8 | 35 | Frankia-7 | F201370.dat | 242 | 23 | 6 | 6 | 6 | 2 | 1 | 2 | 3 | 4 | 0.012% | 5 | 6 | 5 | 4 | 3 | 0.022% | 1.65 | 0.50 | 7.9E-03 | Blue |
| WP_011602671.1 | FRAAL1481 | hypothetical protein | 41804 | 4.11 | 12 | 80 | Frankia-7 | F201370.dat | 523 | 28 | 11 | 11 | 14 | 7 | 5 | 7 | 6 | 4 | 0.025% | 7 | 14 | 11 | 12 | 7 | 0.041% | 1.65 | 0.50 | 9.7E-03 | Blue |
| WP_011606441.1 | FRAAL5356 | PPOX class F420-dependent enzyme | 14725 | 6.02 | 6 | 64 | Frankia-10 | F201373.dat | 281 | 41 | 6 | 6 | 10 | 4 | 5 | 5 | 4 | 5 | 0.056% | 7 | 8 | 7 | 9 | 10 | 0.094% | 1.64 | 0.50 | 3.4E-04 | Blue |
| WP_011603543.1 | FRAAL2382 | ABC transporter substrate-binding protein | 28934 | 5.73 | 10 | 93 | Frankia-9 | F201372.dat | 543 | 58 | 10 | 10 | 14 | 6 | 5 | 9 | 9 | 5 | 0.042% | 9 | 13 | 13 | 14 | 10 | 0.069% | 1.64 | 0.50 | 2.8E-03 | Blue |
| WP_041939046.1 | FRAAL2079 | hypothetical protein | 10315 | 10.26 | 3 | 56 | Frankia-10 | F201373.dat | 169 | 41 | 3 | 3 | 7 | 2 | 4 | 4 | 5 | 5 | 0.069% | 6 | 6 | 10 | 7 | 7 | 0.118% | 1.64 | 0.49 | 4.1E-03 | Blue |
| WP_041939825.1 | FRAAL6108 | hypothetical protein | 39701 | 9.96 | 6 | 19 | Frankia-8 | F201371.dat | 112 | 10 | 4 | 4 | 4 | 1 | 1 | 2 | 1 | 1 | 0.005% | 2 | 2 | 4 | 3 | 2 | 0.011% | 1.64 | 0.49 | 1.4E-02 | Blue |
| WP_011602194.1 | FRAAL0965 | PhoU family transcriptional regulator | 23841 | 5.00 | 11 | 124 | Frankia-8 | F201371.dat | 592 | 55 | 10 | 10 | 17 | 8 | 9 | 9 | 9 | 11 | 0.069% | 13 | 17 | 17 | 14 | 17 | 0.110% | 1.63 | 0.49 | 1.0E-04 | Blue |
| WP_011603606.1 | FRAAL2449 | F420-dependent oxidoreductase | 33608 | 5.28 | 6 | 32 | Frankia-10 | F201373.dat | 163 | 14 | 4 | 4 | 4 | 2 | 1 | 3 | 3 | 2 | 0.012% | 4 | 3 | 6 | 4 | 4 | 0.021% | 1.63 | 0.49 | 5.9E-03 | Blue |
| WP_041939500.1 | FRAAL4399 | enoyl-CoA hydratase | 26476 | 5.08 | 3 | 11 | Frankia-10 | F201373.dat | 132 | 18 | 3 | 3 | 3 | 1 | 0 | 1 | 0 | 1 | 0.004% | 1 | 1 | 2 | 1 | 3 | 0.010% | 1.63 | 0.49 | 4.5E-02 | Blue |
| WP_009399485.1 | FRAAL4541 | transcriptional regulator | 7804 | 10.97 | 10 | 194 | Frankia-10 | F201373.dat | 494 | 68 | 10 | 10 | 32 | 10 | 14 | 14 | 16 | 19 | 0.334% | 19 | 13 | 25 | 32 | 32 | 0.522% | 1.62 | 0.48 | 2.1E-02 | Blue |
| WP_011602662.1 | FRAAL1472 | bacterioferritin | 17983 | 4.91 | 4 | 24 | Frankia-10 | F201373.dat | 194 | 35 | 4 | 4 | 4 | 1 | 2 | 2 | 2 | 1 | 0.016% | 1 | 2 | 4 | 5 | 4 | 0.030% | 1.62 | 0.48 | 4.1E-02 | Blue |
| WP_011602509.1 | FRAAL1311 | flagellar protein FlgA | 26844 | 9.75 | 8 | 50 | Frankia-7 | F201370.dat | 393 | 43 | 6 | 6 | 7 | 3 | 3 | 5 | 3 | 4 | 0.024% | 4 | 7 | 8 | 6 | 7 | 0.040% | 1.61 | 0.48 | 3.7E-03 | Blue |
| WP_041939756.1 | FRAAL5767 | Rrf2 family transcriptional regulator | 16307 | 7.90 | 8 | 42 | Frankia-9 | F201372.dat | 277 | 44 | 6 | 6 | 7 | 3 | 3 | 3 | 4 | 2 | 0.033% | 5 | 5 | 6 | 7 | 4 | 0.056% | 1.60 | 0.47 | 2.1E-03 | Blue |
| WP_041941153.1 | FRAAL6784 | peptidoglycan-binding protein | 29122 | 7.72 | 10 | 55 | Frankia-10 | F201373.dat | 329 | 39 | 8 | 8 | 8 | 3 | 4 | 5 | 5 | 3 | 0.025% | 6 | 5 | 9 | 7 | 8 | 0.040% | 1.60 | 0.47 | 3.6E-03 | Blue |
| WP_041939621.1 | FRAAL5043 | methyltransferase | 27086 | 5.48 | 4 | 16 | Frankia-10 | F201373.dat | 101 | 10 | 2 | 2 | 3 | 1 | 1 | 1 | 1 | 1 | 0.007% | 1 | 2 | 3 | 2 | 3 | 0.014% | 1.60 | 0.47 | 2.2E-02 | Blue |
| WP_050997472.1 | FRAAL6607 | hypothetical protein | 11756 | 10.12 | 2 | 16 | Frankia-8 | F201371.dat | 100 | 21 | 2 | 2 | 3 | 1 | 1 | 1 | 2 | 0 | 0.015% | 1 | 2 | 3 | 3 | 2 | 0.032% | 1.60 | 0.47 | 2.2E-02 | Blue |
| WP_041939662.1 | FRAAL5238 | aspartate carbamoyltransferase | 33138 | 7.12 | 4 | 16 | Frankia-7 | F201370.dat | 137 | 11 | 3 | 3 | 3 | 2 | 2 | 0 | 1 | 0 | 0.005% | 2 | 3 | 1 | 2 | 3 | 0.011% | 1.60 | 0.47 | 3.7E-02 | Blue |
| WP_041939371.1 | FRAAL3616 | methyltransferase | 33179 | 10.84 | 8 | 47 | Frankia-7 | F201370.dat | 235 | 26 | 7 | 7 | 8 | 5 | 4 | 3 | 3 | 2 | 0.018% | 6 | 8 | 4 | 7 | 5 | 0.030% | 1.59 | 0.46 | 8.8E-03 | Blue |
| WP_009740518.1 | FRAAL1091 | 50S ribosomal protein L14 | 13321 | 10.44 | 9 | 135 | Frankia-10 | F201373.dat | 434 | 56 | 9 | 9 | 20 | 11 | 11 | 10 | 10 | 9 | 0.137% | 15 | 16 | 17 | 16 | 20 | 0.212% | 1.59 | 0.46 | 5.4E-05 | Blue |
| WP_011603645.1 | FRAAL2487 | oxidoreductase | 37358 | 5.35 | 11 | 78 | Frankia-8 | F201371.dat | 530 | 28 | 8 | 8 | 12 | 5 | 5 | 6 | 6 | 7 | 0.028% | 8 | 8 | 12 | 10 | 11 | 0.044% | 1.59 | 0.46 | 9.6E-04 | Blue |
| WP_041939942.1 | FRAAL6659 | hypothetical protein | 14853 | 5.08 | 4 | 34 | Frankia-6 | F201369.dat | 193 | 28 | 3 | 3 | 5 | 2 | 4 | 3 | 2 | 1 | 0.029% | 5 | 5 | 5 | 4 | 3 | 0.050% | 1.59 | 0.46 | 7.5E-03 | Blue |
| WP_011603311.1 | FRAAL2146 | adenine phosphoribosyltransferase | 19413 | 4.89 | 8 | 83 | Frankia-8 | F201371.dat | 403 | 60 | 8 | 8 | 11 | 6 | 7 | 7 | 5 | 6 | 0.057% | 11 | 11 | 11 | 10 | 9 | 0.090% | 1.58 | 0.46 | 3.0E-05 | Blue |
| WP_011607174.1 | FRAAL6123 | succinyl-diaminopimelate desuccinylase | 38509 | 5.38 | 3 | 21 | Frankia-7 | F201370.dat | 126 | 9 | 3 | 3 | 3 | 1 | 1 | 2 | 1 | 2 | 0.006% | 3 | 3 | 3 | 3 | 2 | 0.012% | 1.58 | 0.46 | 8.7E-03 | Blue |
| WP_009739808.1 | FRAAL1926 | 50S ribosomal protein L27 | 9594 | 11.77 | 8 | 83 | Frankia-10 | F201373.dat | 350 | 70 | 8 | 8 | 14 | 5 | 7 | 6 | 7 | 6 | 0.115% | 7 | 7 | 12 | 12 | 14 | 0.183% | 1.58 | 0.46 | 1.1E-02 | Blue |
| WP_011605195.1 | FRAAL4065 | aminoglycoside phosphotransferase | 51503 | 5.42 | 4 | 21 | Frankia-7 | F201370.dat | 193 | 7 | 3 | 3 | 3 | 2 | 0 | 0 | 2 | 3 | 0.005% | 3 | 3 | 3 | 3 | 2 | 0.009% | 1.58 | 0.46 | 3.8E-02 | Blue |
| WP_011607548.1 | FRAAL6507 | Terpene cyclase | 85180 | 5.18 | 21 | 124 | Frankia-9 | F201372.dat | 756 | 25 | 16 | 16 | 17 | 13 | 9 | 10 | 9 | 6 | 0.020% | 15 | 14 | 15 | 17 | 16 | 0.030% | 1.58 | 0.46 | 6.2E-04 | Blue |
| WP_011602802.1 | FRAAL1613 | nitroreductase | 16295 | 5.45 | 5 | 44 | Frankia-6 | F201369.dat | 251 | 47 | 5 | 5 | 6 | 2 | 3 | 4 | 3 | 4 | 0.035% | 6 | 4 | 6 | 5 | 7 | 0.058% | 1.57 | 0.45 | 2.6E-03 | Blue |
| WP_041939096.1 | FRAAL2279 | cell division protein FtsK | 109375 | 5.93 | 10 | 44 | Frankia-10 | F201373.dat | 205 | 10 | 7 | 7 | 7 | 3 | 2 | 3 | 5 | 3 | 0.005% | 4 | 4 | 6 | 7 | 7 | 0.009% | 1.57 | 0.45 | 1.0E-02 | Blue |
| WP_011606896.1 | FRAAL5828 | 3-isopropylmalate dehydratase small subunit | 21425 | 4.59 | 6 | 26 | Frankia-8 | F201371.dat | 237 | 29 | 4 | 4 | 4 | 0 | 2 | 2 | 2 | 3 | 0.015% | 4 | 3 | 4 | 3 | 3 | 0.027% | 1.57 | 0.45 | 1.3E-02 | Blue |
| WP_011607450.1 | FRAAL6405 | transcriptional regulator | 26646 | 6.33 | 5 | 31 | Frankia-8 | F201371.dat | 241 | 29 | 5 | 5 | 5 | 1 | 3 | 2 | 3 | 2 | 0.015% | 4 | 4 | 5 | 4 | 3 | 0.025% | 1.56 | 0.45 | 3.5E-03 | Blue |
| WP_011602999.1 | FRAAL1820 | Serine-threonine protein kinase (afsK-like) | 60365 | 5.41 | 9 | 31 | Frankia-8 | F201371.dat | 266 | 9 | 5 | 5 | 5 | 1 | 2 | 2 | 2 | 4 | 0.007% | 4 | 2 | 5 | 4 | 5 | 0.011% | 1.56 | 0.45 | 2.0E-02 | Blue |
| WP_041938895.1 | FRAAL1319 | pilus assembly protein CpaF | 47593 | 5.56 | 6 | 31 | Frankia-7 | F201370.dat | 214 | 15 | 5 | 5 | 6 | 3 | 1 | 2 | 4 | 1 | 0.008% | 4 | 6 | 2 | 4 | 4 | 0.014% | 1.56 | 0.45 | 3.5E-02 | Blue |
| WP_011606777.1 | FRAAL5703 | LexA repressor | 26901 | 9.20 | 7 | 54 | Frankia-10 | F201373.dat | 371 | 38 | 6 | 6 | 8 | 3 | 3 | 5 | 3 | 6 | 0.027% | 6 | 7 | 9 | 4 | 8 | 0.043% | 1.56 | 0.44 | 1.5E-02 | Blue |
| WP_011603168.1 | FRAAL1996 | polyisoprenoid-binding protein | 19369 | 4.83 | 11 | 100 | Frankia-8 | F201371.dat | 478 | 49 | 10 | 10 | 15 | 7 | 7 | 8 | 7 | 9 | 0.070% | 10 | 12 | 15 | 12 | 13 | 0.108% | 1.56 | 0.44 | 3.6E-04 | Blue |
| WP_009741926.1 | FRAAL6293 | hypothetical protein | 6926 | 4.99 | 3 | 36 | Frankia-8 | F201371.dat | 189 | 59 | 3 | 3 | 4 | 3 | 3 | 3 | 2 | 2 | 0.067% | 4 | 4 | 4 | 6 | 5 | 0.112% | 1.56 | 0.44 | 2.4E-03 | Blue |
| WP_011606966.1 | FRAAL5905 | hypothetical protein | 11517 | 4.85 | 2 | 13 | Frankia-10 | F201373.dat | 109 | 40 | 2 | 2 | 2 | 1 | 1 | 0 | 1 | 1 | 0.012% | 2 | 2 | 2 | 1 | 2 | 0.026% | 1.56 | 0.44 | 3.3E-02 | Blue |
| WP_041939071.1 | FRAAL2183 | SNF family Na+-dependent transporter | 15441 | 11.62 | 2 | 13 | Frankia-10 | F201373.dat | 99 | 16 | 2 | 2 | 2 | 1 | 1 | 0 | 1 | 1 | 0.009% | 1 | 2 | 2 | 2 | 2 | 0.020% | 1.56 | 0.44 | 3.3E-02 | Blue |
| WP_011604933.1 | FRAAL3795 | TetR family transcriptional regulator | 21359 | 9.97 | 3 | 13 | Frankia-8 | F201371.dat | 117 | 16 | 3 | 3 | 3 | 1 | 1 | 1 | 1 | 0 | 0.007% | 1 | 2 | 3 | 2 | 1 | 0.014% | 1.56 | 0.44 | 4.0E-02 | Blue |
| WP_011601953.1 | FRAAL0711 | DNA-binding protein | 15528 | 10.88 | 7 | 69 | Frankia-7 | F201370.dat | 273 | 40 | 6 | 6 | 9 | 7 | 4 | 5 | 5 | 5 | 0.060% | 8 | 9 | 8 | 9 | 9 | 0.093% | 1.55 | 0.44 | 2.1E-04 | Blue |
| WP_050997452.1 | FRAAL5922 | hypothetical protein | 21572 | 6.83 | 3 | 18 | Frankia-8 | F201371.dat | 131 | 21 | 3 | 3 | 3 | 2 | 1 | 1 | 1 | 1 | 0.010% | 2 | 3 | 3 | 2 | 2 | 0.019% | 1.55 | 0.44 | 1.7E-02 | Blue |
| WP_041939636.1 | FRAAL5105 | hydrolase | 14294 | 4.90 | 2 | 18 | Frankia-10 | F201373.dat | 101 | 14 | 2 | 2 | 3 | 1 | 1 | 1 | 2 | 1 | 0.015% | 2 | 2 | 2 | 3 | 3 | 0.028% | 1.55 | 0.44 | 1.7E-02 | Blue |
| WP_011602220.1 | FRAAL0993 | hydrolase | 29949 | 6.38 | 4 | 18 | Frankia-6 | F201369.dat | 135 | 11 | 3 | 3 | 3 | 2 | 1 | 0 | 2 | 1 | 0.007% | 3 | 2 | 2 | 2 | 3 | 0.013% | 1.55 | 0.44 | 2.2E-02 | Blue |
| WP_011603591.1 | FRAAL2433 | NADH dehydrogenase | 27017 | 5.83 | 5 | 18 | Frankia-6 | F201369.dat | 189 | 19 | 3 | 3 | 3 | 2 | 0 | 1 | 2 | 1 | 0.008% | 3 | 2 | 3 | 3 | 1 | 0.015% | 1.55 | 0.44 | 3.0E-02 | Blue |
| WP_041938883.1 | FRAAL1255 | GDP-mannose pyrophosphorylase | 36508 | 4.98 | 5 | 18 | Frankia-10 | F201373.dat | 151 | 23 | 4 | 4 | 4 | 2 | 1 | 1 | 1 | 1 | 0.006% | 2 | 3 | 2 | 1 | 4 | 0.011% | 1.55 | 0.44 | 4.2E-02 | Blue |
| WP_011606975.1 | FRAAL5914 | sugar-binding protein | 25759 | 4.85 | 17 | 140 | Frankia-8 | F201371.dat | 678 | 51 | 12 | 12 | 20 | 9 | 11 | 12 | 12 | 10 | 0.075% | 16 | 18 | 20 | 19 | 13 | 0.112% | 1.54 | 0.43 | 8.0E-04 | Blue |
| WP_011605597.1 | FRAAL4476 | hypothetical protein | 24086 | 6.32 | 4 | 23 | Frankia-7 | F201370.dat | 185 | 29 | 4 | 4 | 4 | 2 | 1 | 2 | 1 | 2 | 0.012% | 3 | 4 | 3 | 3 | 2 | 0.021% | 1.54 | 0.43 | 8.7E-03 | Blue |
| WP_011607005.1 | FRAAL5947 | 50S ribosomal protein L31 | 8699 | 9.46 | 7 | 94 | Frankia-10 | F201373.dat | 373 | 79 | 7 | 7 | 16 | 7 | 6 | 6 | 9 | 8 | 0.148% | 10 | 9 | 12 | 11 | 16 | 0.225% | 1.54 | 0.43 | 5.6E-03 | Blue |
| WP_011607885.1 | FRAAL6850 | 50S ribosomal protein L9 | 15737 | 9.86 | 9 | 132 | Frankia-8 | F201371.dat | 594 | 76 | 9 | 9 | 19 | 11 | 10 | 13 | 8 | 9 | 0.116% | 13 | 15 | 19 | 15 | 19 | 0.173% | 1.54 | 0.43 | 1.8E-03 | Blue |
| WP_041938825.1 | FRAAL0974 | pyrroline-5-carboxylate reductase | 26859 | 5.70 | 8 | 99 | Frankia-8 | F201371.dat | 529 | 45 | 8 | 8 | 14 | 8 | 8 | 7 | 8 | 7 | 0.050% | 12 | 14 | 14 | 11 | 10 | 0.076% | 1.53 | 0.43 | 3.6E-04 | Blue |
| WP_009742848.1 | FRAAL5174 | hypothetical protein | 7398 | 5.05 | 3 | 28 | Frankia-6 | F201369.dat | 120 | 50 | 3 | 3 | 5 | 2 | 2 | 2 | 2 | 2 | 0.048% | 5 | 4 | 2 | 4 | 3 | 0.082% | 1.53 | 0.43 | 1.5E-02 | Blue |
| WP_041939587.1 | FRAAL4898 | LuxR family transcriptional regulator | 31271 | 10.96 | 7 | 33 | Frankia-7 | F201370.dat | 147 | 22 | 5 | 5 | 5 | 1 | 3 | 3 | 2 | 3 | 0.014% | 3 | 5 | 4 | 4 | 5 | 0.023% | 1.53 | 0.42 | 5.5E-03 | Blue |
| WP_011602138.1 | FRAAL0908 | DNA-binding protein | 24974 | 5.79 | 16 | 126 | Frankia-10 | F201373.dat | 569 | 58 | 12 | 12 | 17 | 8 | 8 | 12 | 11 | 10 | 0.070% | 14 | 17 | 14 | 15 | 17 | 0.104% | 1.52 | 0.42 | 3.5E-04 | Blue |
| WP_041939514.1 | FRAAL4478 | oxidoreductase | 34900 | 9.77 | 11 | 58 | Frankia-8 | F201371.dat | 396 | 28 | 8 | 8 | 9 | 4 | 5 | 5 | 4 | 4 | 0.022% | 5 | 11 | 9 | 7 | 4 | 0.035% | 1.52 | 0.42 | 3.4E-02 | Blue |
| WP_011602860.1 | FRAAL1673 | molybdenum-binding protein | 14178 | 5.12 | 8 | 63 | Frankia-7 | F201370.dat | 437 | 51 | 7 | 7 | 9 | 6 | 5 | 6 | 5 | 2 | 0.060% | 5 | 9 | 8 | 9 | 8 | 0.093% | 1.52 | 0.42 | 1.0E-02 | Blue |
| WP_011607458.1 | FRAAL6413 | histidine kinase | 23211 | 4.94 | 7 | 60 | Frankia-7 | F201370.dat | 239 | 32 | 5 | 5 | 7 | 5 | 4 | 5 | 4 | 5 | 0.035% | 7 | 7 | 9 | 7 | 7 | 0.054% | 1.50 | 0.41 | 3.3E-04 | Blue |
| WP_009740532.1 | FRAAL1076 | 30S ribosomal protein S12 | 13803 | 11.46 | 9 | 80 | Frankia-8 | F201371.dat | 300 | 59 | 7 | 7 | 13 | 6 | 6 | 6 | 5 | 8 | 0.080% | 10 | 8 | 13 | 8 | 10 | 0.120% | 1.50 | 0.41 | 4.3E-03 | Blue |
| WP_009741728.1 | FRAAL6574 | anti-sigma B factor antagonist | 12281 | 5.53 | 6 | 95 | Frankia-10 | F201373.dat | 305 | 54 | 6 | 6 | 16 | 7 | 7 | 8 | 8 | 7 | 0.108% | 10 | 9 | 11 | 12 | 16 | 0.159% | 1.50 | 0.41 | 5.0E-03 | Blue |
| WP_011607166.1 | FRAAL6115 | hypothetical protein | 38394 | 8.39 | 8 | 40 | Frankia-6 | F201369.dat | 238 | 23 | 7 | 7 | 7 | 3 | 3 | 4 | 2 | 3 | 0.014% | 7 | 5 | 3 | 5 | 5 | 0.022% | 1.50 | 0.41 | 1.2E-02 | Blue |
| WP_011602612.1 | FRAAL1417 | methylmalonate-semialdehyde dehydrogenase | 51746 | 5.79 | 10 | 50 | Frankia-8 | F201371.dat | 268 | 17 | 7 | 7 | 9 | 2 | 4 | 3 | 4 | 6 | 0.013% | 6 | 5 | 9 | 5 | 6 | 0.020% | 1.50 | 0.41 | 2.1E-02 | Blue |
| WP_050997347.1 | FRAAL2171 | acetyltransferase | 21200 | 5.95 | 3 | 20 | Frankia-8 | F201371.dat | 144 | 18 | 3 | 3 | 3 | 2 | 0 | 2 | 1 | 2 | 0.012% | 2 | 2 | 3 | 3 | 3 | 0.021% | 1.50 | 0.41 | 2.5E-02 | Blue |
| WP_011606808.1 | FRAAL5734 | ribosomal protein S12 methylthiotransferase RimO | 56084 | 4.90 | 6 | 25 | Frankia-8 | F201371.dat | 179 | 15 | 5 | 5 | 5 | 3 | 2 | 2 | 1 | 1 | 0.006% | 3 | 3 | 5 | 3 | 2 | 0.010% | 1.50 | 0.41 | 2.6E-02 | Blue |
| WP_011605665.1 | FRAAL4547 | hypothetical protein | 59965 | 5.26 | 17 | 80 | Frankia-8 | F201371.dat | 599 | 25 | 11 | 11 | 14 | 7 | 6 | 4 | 8 | 6 | 0.018% | 5 | 12 | 14 | 9 | 9 | 0.028% | 1.50 | 0.41 | 3.1E-02 | Blue |
| WP_011606196.1 | FRAAL5087 | glycerol acyltransferase | 33486 | 10.12 | 3 | 15 | Frankia-9 | F201372.dat | 112 | 10 | 3 | 3 | 3 | 1 | 1 | 1 | 1 | 1 | 0.005% | 1 | 2 | 2 | 3 | 2 | 0.010% | 1.50 | 0.41 | 3.3E-02 | Blue |
| WP_011607796.1 | FRAAL6760 | hypothetical protein | 12915 | 9.74 | 2 | 15 | Frankia-3 | F201366.dat | 139 | 18 | 2 | 2 | 2 | 0 | 1 | 2 | 1 | 1 | 0.014% | 2 | 2 | 2 | 2 | 2 | 0.026% | 1.50 | 0.41 | 3.3E-02 | Blue |
| WP_011602563.1 | FRAAL1366 | glyoxalase | 17282 | 5.14 | 5 | 25 | Frankia-8 | F201371.dat | 135 | 31 | 4 | 4 | 5 | 1 | 1 | 3 | 3 | 1 | 0.019% | 2 | 3 | 5 | 3 | 3 | 0.031% | 1.50 | 0.41 | 3.9E-02 | Blue |
| WP_041939323.1 | FRAAL3291 | NADH:riboflavin 5'-phosphate oxidoreductase | 25551 | 11.41 | 4 | 25 | Frankia-10 | F201373.dat | 174 | 21 | 4 | 4 | 5 | 2 | 1 | 1 | 3 | 2 | 0.013% | 2 | 2 | 3 | 4 | 5 | 0.021% | 1.50 | 0.41 | 3.9E-02 | Blue |
| WP_041938621.1 | FRAAL0133 | hypothetical protein | 31189 | 7.81 | 5 | 20 | Frankia-8 | F201371.dat | 168 | 19 | 4 | 4 | 4 | 1 | 2 | 1 | 1 | 2 | 0.008% | 1 | 3 | 4 | 3 | 2 | 0.014% | 1.50 | 0.41 | 4.2E-02 | Blue |
| WP_011606881.1 | FRAAL5811 | DEAD/DEAH box helicase | 78353 | 6.11 | 8 | 40 | Frankia-3 | F201366.dat | 300 | 10 | 6 | 6 | 6 | 5 | 6 | 6 | 4 | 4 | 0.011% | 4 | 2 | 4 | 3 | 2 | 0.006% | -1.50 | -0.41 | 6.7E-03 | Blue |
| WP_041939786.1 | FRAAL5945 | peptide chain release factor 1 | 38447 | 4.93 | 13 | 50 | Frankia-2 | F201365.dat | 205 | 19 | 6 | 6 | 6 | 6 | 6 | 6 | 6 | 7 | 0.029% | 1 | 4 | 6 | 4 | 4 | 0.017% | -1.50 | -0.41 | 1.2E-02 | Blue |
| WP_041939050.1 | FRAAL2097 | acetyl-CoA acetyltransferase | 42248 | 5.40 | 9 | 60 | Frankia-4 | F201367.dat | 397 | 19 | 8 | 8 | 9 | 9 | 6 | 6 | 9 | 7 | 0.031% | 3 | 6 | 8 | 2 | 4 | 0.018% | -1.50 | -0.41 | 3.0E-02 | Blue |
| WP_011602266.1 | FRAAL1039 | NADH-quinone oxidoreductase subunit H | 48722 | 5.74 | 2 | 15 | Frankia-3 | F201366.dat | 74 | 2 | 2 | 2 | 2 | 2 | 2 | 2 | 2 | 2 | 0.007% | 1 | 0 | 2 | 1 | 1 | 0.003% | -1.50 | -0.41 | 3.3E-02 | Blue |
| WP_011603278.1 | FRAAL2113 | deaminase | 28748 | 4.82 | 5 | 20 | Frankia-3 | F201366.dat | 128 | 14 | 3 | 3 | 4 | 1 | 3 | 4 | 3 | 2 | 0.016% | 1 | 2 | 2 | 1 | 1 | 0.008% | -1.50 | -0.41 | 4.2E-02 | Blue |
| WP_011605691.1 | FRAAL4574 | transaldolase | 39932 | 4.81 | 26 | 348 | Frankia-5 | F201368.dat | 1038 | 47 | 23 | 23 | 45 | 42 | 40 | 37 | 46 | 45 | 0.188% | 26 | 28 | 29 | 25 | 30 | 0.116% | -1.50 | -0.41 | 3.1E-05 | Blue |
| WP_011606846.1 | FRAAL5774 | 1-deoxy-D-xylulose 5-phosphate reductoisomerase | 43150 | 5.06 | 14 | 118 | Frankia-2 | F201365.dat | 598 | 34 | 13 | 13 | 16 | 15 | 16 | 14 | 13 | 14 | 0.060% | 7 | 9 | 10 | 7 | 13 | 0.036% | -1.51 | -0.41 | 1.4E-03 | Blue |
| WP_011606315.1 | FRAAL5220 | 3.4-dihydroxy-2-butanone 4-phosphate synthase | 53428 | 5.14 | 12 | 63 | Frankia-5 | F201368.dat | 394 | 21 | 9 | 9 | 9 | 8 | 7 | 7 | 8 | 9 | 0.026% | 6 | 5 | 4 | 5 | 4 | 0.015% | -1.52 | -0.42 | 2.4E-04 | Blue |
| WP_011605800.1 | FRAAL4684 | carbamoyl phosphate synthase small subunit | 40941 | 6.06 | 9 | 38 | Frankia-5 | F201368.dat | 274 | 24 | 6 | 6 | 6 | 3 | 5 | 4 | 6 | 6 | 0.021% | 1 | 3 | 4 | 2 | 4 | 0.012% | -1.53 | -0.42 | 2.1E-02 | Blue |
| WP_011604497.1 | FRAAL3350 | cysteine synthase | 32286 | 5.02 | 15 | 268 | Frankia-5 | F201368.dat | 752 | 43 | 15 | 15 | 35 | 30 | 34 | 30 | 34 | 35 | 0.180% | 20 | 23 | 20 | 21 | 21 | 0.110% | -1.53 | -0.42 | 5.8E-06 | Blue |
| WP_041939603.1 | FRAAL4991 | thiamine biosynthesis protein ThiF | 50661 | 5.66 | 6 | 28 | Frankia-5 | F201368.dat | 220 | 16 | 5 | 5 | 5 | 4 | 3 | 3 | 3 | 5 | 0.013% | 2 | 2 | 2 | 2 | 2 | 0.007% | -1.53 | -0.43 | 7.5E-03 | Blue |
| WP_041939769.1 | FRAAL5846 | 2-isopropylmalate synthase | 64143 | 4.92 | 27 | 168 | Frankia-1 | F201364.dat | 754 | 41 | 21 | 21 | 25 | 25 | 21 | 17 | 22 | 18 | 0.057% | 11 | 12 | 18 | 12 | 12 | 0.034% | -1.54 | -0.43 | 2.1E-03 | Blue |
| WP_011607845.1 | FRAAL6811 | nitrogenase molybdenum-iron protein subunit beta | 57519 | 5.59 | 45 | 1063 | Frankia-2 | F201365.dat | 2433 | 74 | 41 | 41 | 124 | 125 | 124 | 132 | 130 | 136 | 0.401% | 78 | 87 | 87 | 83 | 81 | 0.244% | -1.55 | -0.44 | 9.9E-08 | Blue |
| WP_011601523.1 | FRAAL0263 | hypothetical protein | 71500 | 6.10 | 11 | 59 | Frankia-1 | F201364.dat | 312 | 17 | 8 | 8 | 9 | 9 | 7 | 6 | 9 | 6 | 0.018% | 4 | 4 | 5 | 4 | 5 | 0.010% | -1.56 | -0.44 | 2.0E-03 | Blue |
| WP_011605685.1 | FRAAL4568 | spermidine/putrescine ABC transporter ATP-binding protein | 38270 | 7.04 | 7 | 36 | Frankia-4 | F201367.dat | 235 | 22 | 5 | 5 | 6 | 4 | 4 | 4 | 6 | 5 | 0.021% | 2 | 3 | 3 | 2 | 3 | 0.011% | -1.56 | -0.44 | 2.4E-03 | Blue |
| WP_041939289.1 | FRAAL3139 | hypothetical protein | 26268 | 4.83 | 7 | 54 | Frankia-2 | F201365.dat | 509 | 34 | 7 | 7 | 9 | 6 | 9 | 6 | 6 | 7 | 0.046% | 4 | 3 | 4 | 5 | 4 | 0.026% | -1.56 | -0.44 | 1.5E-03 | Blue |
| WP_011607172.1 | FRAAL6120 | hypothetical protein | 29461 | 6.16 | 8 | 54 | Frankia-3 | F201366.dat | 279 | 24 | 6 | 6 | 9 | 6 | 7 | 9 | 5 | 7 | 0.041% | 5 | 4 | 2 | 6 | 3 | 0.023% | -1.56 | -0.44 | 1.0E-02 | Blue |
| WP_011604505.1 | FRAAL3358 | hopanoid biosynthesis associated radical SAM protein HpnH | 37249 | 6.66 | 9 | 72 | Frankia-4 | F201367.dat | 445 | 36 | 8 | 8 | 11 | 9 | 7 | 13 | 11 | 5 | 0.043% | 5 | 7 | 5 | 5 | 5 | 0.024% | -1.56 | -0.45 | 2.0E-02 | Blue |
| WP_011607479.1 | FRAAL6434 | glycine dehydrogenase subunit 1 | 109257 | 5.74 | 26 | 150 | Frankia-8 | F201371.dat | 1026 | 27 | 19 | 19 | 22 | 22 | 19 | 17 | 12 | 23 | 0.030% | 8 | 7 | 22 | 6 | 14 | 0.018% | -1.58 | -0.46 | 4.0E-02 | Blue |
| WP_011607527.1 | FRAAL6486 | molecular chaperone GroEL | 57071 | 4.90 | 10 | 143 | Frankia-4 | F201367.dat | 4915 | 88 | 69 | 9 | 16 | 19 | 18 | 17 | 16 | 19 | 0.056% | 11 | 11 | 10 | 11 | 11 | 0.032% | -1.59 | -0.47 | 3.1E-06 | Blue |
| WP_011603115.1 | FRAAL1942 | signal peptide | 35798 | 9.30 | 3 | 42 | Frankia-3 | F201366.dat | 222 | 19 | 3 | 3 | 7 | 5 | 5 | 7 | 4 | 6 | 0.027% | 3 | 4 | 2 | 3 | 3 | 0.014% | -1.60 | -0.47 | 2.1E-03 | Blue |
| WP_041939748.1 | FRAAL5686 | transcriptional regulator | 54572 | 9.63 | 5 | 16 | Frankia-1 | F201364.dat | 182 | 11 | 3 | 3 | 3 | 3 | 3 | 1 | 1 | 3 | 0.007% | 1 | 2 | 0 | 1 | 1 | 0.003% | -1.60 | -0.47 | 3.8E-02 | Blue |
| WP_011605680.1 | FRAAL4563 | Fe-S cluster assembly protein SufB | 51814 | 5.03 | 31 | 339 | Frankia-3 | F201366.dat | 1495 | 66 | 26 | 26 | 41 | 45 | 42 | 41 | 43 | 39 | 0.145% | 24 | 30 | 25 | 24 | 26 | 0.084% | -1.60 | -0.47 | 2.3E-06 | Blue |
| WP_011602774.1 | FRAAL1432 | squalene-hopene cyclase | 76535 | 5.42 | 24 | 291 | Frankia-3 | F201366.dat | 1032 | 34 | 21 | 21 | 37 | 40 | 38 | 37 | 34 | 32 | 0.084% | 23 | 26 | 26 | 20 | 15 | 0.048% | -1.62 | -0.48 | 2.4E-04 | Blue |
| WP_011602407.1 | FRAAL1204 | amidohydrolase | 42320 | 5.84 | 8 | 53 | Frankia-1 | F201364.dat | 362 | 28 | 8 | 8 | 8 | 8 | 5 | 7 | 7 | 7 | 0.029% | 4 | 4 | 3 | 3 | 5 | 0.015% | -1.63 | -0.49 | 6.2E-04 | Blue |
| WP_011606193.1 | FRAAL5084 | 6-phosphofructokinase | 36186 | 5.52 | 7 | 32 | Frankia-2 | F201365.dat | 324 | 26 | 6 | 6 | 6 | 3 | 6 | 4 | 3 | 5 | 0.021% | 2 | 2 | 3 | 2 | 2 | 0.010% | -1.63 | -0.49 | 8.8E-03 | Blue |
| WP_011607054.1 | FRAAL6001 | 8-amino-7-oxononanoate synthase | 46901 | 7.88 | 7 | 27 | Frankia-1 | F201364.dat | 223 | 17 | 4 | 4 | 4 | 4 | 2 | 4 | 5 | 3 | 0.014% | 3 | 2 | 2 | 1 | 1 | 0.006% | -1.64 | -0.50 | 1.1E-02 | Blue |
| WP_041938862.1 | FRAAL1155 | serine/threonine protein kinase | 68002 | 9.15 | 5 | 22 | Frankia-5 | F201368.dat | 180 | 8 | 3 | 3 | 3 | 4 | 4 | 2 | 2 | 3 | 0.008% | 2 | 0 | 1 | 2 | 2 | 0.003% | -1.67 | -0.51 | 1.4E-02 | Blue |
| WP_050997052.1 | FRAAL2038 | macrocin-O-methyltransferase | 31330 | 5.10 | 12 | 57 | Frankia-3 | F201366.dat | 321 | 36 | 8 | 8 | 8 | 6 | 7 | 8 | 7 | 9 | 0.042% | 4 | 4 | 4 | 4 | 4 | 0.021% | -1.68 | -0.52 | 2.6E-04 | Blue |
| WP_011607590.1 | FRAAL6550 | ATPase | 37660 | 5.53 | 8 | 25 | Frankia-5 | F201368.dat | 176 | 16 | 4 | 4 | 5 | 2 | 4 | 2 | 4 | 5 | 0.016% | 0 | 1 | 2 | 3 | 2 | 0.007% | -1.69 | -0.53 | 2.6E-02 | Blue |
| WP_041940290.1 | FRAAL1829 | cytochrome-c3 hydrogenase | 58420 | 5.23 | 22 | 203 | Frankia-3 | F201366.dat | 968 | 42 | 18 | 18 | 28 | 24 | 26 | 28 | 27 | 24 | 0.079% | 15 | 18 | 12 | 16 | 13 | 0.043% | -1.70 | -0.53 | 1.8E-05 | Blue |
| WP_011606195.1 | FRAAL5086 | hypothetical protein | 47498 | 6.86 | 3 | 17 | Frankia-5 | F201368.dat | 164 | 9 | 3 | 3 | 3 | 2 | 3 | 2 | 2 | 3 | 0.009% | 0 | 1 | 1 | 1 | 2 | 0.004% | -1.70 | -0.53 | 8.7E-03 | Blue |
| WP_011601571.1 | FRAAL0315 | aldehyde dehydrogenase | 52469 | 4.92 | 2 | 9 | Frankia-1 | F201364.dat | 103 | 6 | 2 | 1 | 1 | 1 | 2 | 1 | 1 | 2 | 0.005% | 0 | 0 | 1 | 0 | 1 | 0.001% | -1.71 | -0.54 | 3.3E-02 | Blue |
| WP_011606489.1 | FRAAL5406 | hypothetical protein | 105693 | 5.33 | 9 | 39 | Frankia-5 | F201368.dat | 432 | 9 | 6 | 6 | 6 | 6 | 6 | 5 | 3 | 6 | 0.009% | 4 | 0 | 5 | 4 | 0 | 0.004% | -1.72 | -0.54 | 3.3E-02 | Blue |
| WP_041939982.1 | FRAAL6799 | 2-oxoglutarate ferredoxin oxidoreductase subunit alpha | 71356 | 5.10 | 29 | 585 | Frankia-1 | F201364.dat | 1921 | 61 | 27 | 26 | 76 | 76 | 70 | 82 | 75 | 70 | 0.187% | 44 | 44 | 45 | 41 | 38 | 0.100% | -1.74 | -0.55 | 7.8E-07 | Blue |
| WP_011606566.1 | FRAAL5481 | malate synthase G | 77831 | 5.08 | 13 | 67 | Frankia-2 | F201365.dat | 377 | 15 | 9 | 9 | 10 | 11 | 10 | 9 | 8 | 6 | 0.020% | 6 | 4 | 5 | 4 | 4 | 0.010% | -1.75 | -0.56 | 1.1E-03 | Blue |
| WP_011605224.1 | FRAAL4095 | FMN-dependent alkanal monooxygenase | 34988 | 6.06 | 5 | 23 | Frankia-2 | F201365.dat | 139 | 18 | 4 | 4 | 4 | 3 | 4 | 2 | 3 | 4 | 0.016% | 0 | 2 | 2 | 1 | 2 | 0.007% | -1.75 | -0.56 | 5.5E-03 | Blue |
| WP_011607550.1 | FRAAL6509 | ABC transporter | 69888 | 6.53 | 3 | 12 | Frankia-3 | F201366.dat | 154 | 6 | 2 | 2 | 2 | 2 | 1 | 2 | 2 | 2 | 0.005% | 0 | 0 | 1 | 1 | 1 | 0.001% | -1.75 | -0.56 | 1.7E-02 | Blue |
| WP_041938718.1 | FRAAL0556 | carboxylesterase | 54150 | 4.97 | 4 | 12 | Frankia-3 | F201366.dat | 113 | 9 | 3 | 3 | 3 | 1 | 1 | 3 | 3 | 1 | 0.006% | 0 | 1 | 2 | 0 | 0 | 0.002% | -1.75 | -0.56 | 4.7E-02 | Blue |
| WP_041941155.1 | FRAAL6800 | cysteine desulfurase | 50066 | 5.73 | 9 | 37 | Frankia-1 | F201364.dat | 184 | 16 | 5 | 5 | 6 | 6 | 4 | 5 | 5 | 5 | 0.018% | 2 | 3 | 2 | 3 | 2 | 0.008% | -1.76 | -0.57 | 2.7E-04 | Blue |
| WP_041940288.1 | FRAAL1827 | tetratricopeptide repeat domain protein | 41371 | 5.02 | 14 | 79 | Frankia-4 | F201367.dat | 550 | 42 | 10 | 10 | 11 | 11 | 8 | 12 | 11 | 10 | 0.045% | 6 | 7 | 3 | 6 | 5 | 0.022% | -1.78 | -0.58 | 4.0E-04 | Blue |
| WP_011607311.1 | FRAAL6261 | tyrosyl-tRNA synthetase | 47130 | 5.79 | 21 | 121 | Frankia-1 | F201364.dat | 673 | 46 | 15 | 15 | 18 | 18 | 13 | 16 | 16 | 16 | 0.060% | 5 | 10 | 8 | 10 | 9 | 0.030% | -1.79 | -0.58 | 1.5E-04 | Blue |
| WP_041939002.1 | FRAAL1830 | hydrogenase | 35082 | 6.16 | 8 | 46 | Frankia-3 | F201366.dat | 331 | 27 | 8 | 8 | 8 | 5 | 3 | 8 | 7 | 8 | 0.032% | 2 | 4 | 4 | 3 | 2 | 0.014% | -1.80 | -0.59 | 8.6E-03 | Blue |
| WP_011603177.1 | FRAAL2005 | coproporphyrinogen III oxidase | 44023 | 5.39 | 4 | 18 | Frankia-3 | F201366.dat | 136 | 18 | 4 | 4 | 5 | 2 | 2 | 5 | 2 | 2 | 0.011% | 0 | 1 | 0 | 2 | 2 | 0.004% | -1.80 | -0.59 | 3.2E-02 | Blue |
| WP_041939956.1 | FRAAL6693 | glutamate dehydrogenase | 44441 | 5.35 | 15 | 109 | Frankia-3 | F201366.dat | 637 | 35 | 12 | 12 | 16 | 18 | 12 | 16 | 14 | 12 | 0.058% | 8 | 10 | 9 | 5 | 5 | 0.028% | -1.83 | -0.61 | 1.0E-03 | Blue |
| WP_011605501.1 | FRAAL4379 | enoyl-CoA hydratase | 46249 | 4.73 | 3 | 7 | Frankia-5 | F201368.dat | 54 | 5 | 2 | 2 | 2 | 1 | 1 | 1 | 1 | 2 | 0.005% | 0 | 0 | 0 | 0 | 1 | 0.001% | -1.83 | -0.61 | 3.3E-02 | Blue |
| WP_011603625.1 | FRAAL2468 | esterase | 35187 | 4.72 | 2 | 7 | Frankia-5 | F201368.dat | 115 | 7 | 2 | 2 | 2 | 1 | 1 | 1 | 1 | 2 | 0.006% | 0 | 0 | 1 | 0 | 0 | 0.001% | -1.83 | -0.61 | 3.3E-02 | Blue |
| WP_011605552.1 | FRAAL4430 | hypothetical protein. partial | 11129 | 9.76 | 2 | 7 | Frankia-3 | F201366.dat | 120 | 17 | 2 | 2 | 2 | 1 | 1 | 2 | 2 | 0 | 0.019% | 0 | 0 | 1 | 0 | 0 | 0.003% | -1.83 | -0.61 | 4.0E-02 | Blue |
| WP_011604974.1 | FRAAL3837 | metalloprotease | 22186 | 4.68 | 7 | 24 | Frankia-3 | F201366.dat | 287 | 43 | 6 | 6 | 7 | 2 | 3 | 7 | 2 | 3 | 0.027% | 0 | 3 | 1 | 2 | 1 | 0.011% | -1.83 | -0.61 | 4.8E-02 | Blue |
| WP_011602531.1 | FRAAL1334 | hypothetical protein | 41231 | 10.15 | 7 | 27 | Frankia-4 | F201367.dat | 405 | 22 | 6 | 6 | 6 | 3 | 3 | 4 | 6 | 3 | 0.016% | 2 | 2 | 1 | 2 | 1 | 0.007% | -1.85 | -0.61 | 5.6E-03 | Blue |
| WP_011604402.1 | FRAAL3253 | pyruvate-flavodoxin oxidoreductase | 124979 | 5.84 | 62 | 943 | Frankia-5 | F201368.dat | 3585 | 57 | 56 | 56 | 127 | 125 | 127 | 123 | 113 | 127 | 0.176% | 66 | 61 | 68 | 69 | 64 | 0.088% | -1.86 | -0.62 | 2.7E-08 | Blue |
| WP_041940787.1 | FRAAL4616 | ABC transporter substrate-binding protein | 39118 | 4.95 | 3 | 13 | Frankia-1 | F201364.dat | 186 | 14 | 3 | 3 | 3 | 3 | 1 | 2 | 2 | 2 | 0.009% | 0 | 2 | 1 | 0 | 0 | 0.003% | -1.88 | -0.63 | 1.4E-02 | Blue |
| WP_041939160.1 | FRAAL2577 | hypothetical protein | 28801 | 5.08 | 3 | 25 | Frankia-4 | F201367.dat | 84 | 18 | 3 | 3 | 5 | 4 | 4 | 2 | 5 | 3 | 0.022% | 0 | 2 | 2 | 3 | 0 | 0.008% | -1.92 | -0.65 | 1.2E-02 | Blue |
| WP_011604942.1 | FRAAL3805 | ATP-binding protein | 87959 | 5.39 | 12 | 63 | Frankia-1 | F201364.dat | 407 | 12 | 9 | 9 | 9 | 9 | 10 | 7 | 7 | 10 | 0.017% | 5 | 3 | 4 | 5 | 3 | 0.008% | -1.92 | -0.65 | 2.4E-04 | Blue |
| WP_011606235.1 | FRAAL5128 | quinolinate synthetase | 42900 | 5.81 | 3 | 14 | Frankia-1 | F201364.dat | 93 | 5 | 3 | 3 | 3 | 3 | 2 | 2 | 2 | 2 | 0.009% | 0 | 0 | 1 | 0 | 2 | 0.002% | -2.00 | -0.69 | 7.5E-03 | Blue |
| WP_011607058.1 | FRAAL6005 | acetyl-CoA carboxylase | 7768 | 3.88 | 2 | 14 | Frankia-4 | F201367.dat | 67 | 50 | 2 | 2 | 3 | 2 | 2 | 2 | 3 | 2 | 0.051% | 0 | 0 | 0 | 2 | 1 | 0.013% | -2.00 | -0.69 | 7.5E-03 | Blue |
| WP_011607134.1 | FRAAL6082 | aldehyde dehydrogenase | 50292 | 5.28 | 12 | 47 | Frankia-2 | F201365.dat | 385 | 35 | 9 | 9 | 10 | 5 | 10 | 8 | 7 | 3 | 0.023% | 2 | 5 | 2 | 2 | 3 | 0.009% | -2.00 | -0.69 | 1.1E-02 | Blue |
| WP_011605945.1 | FRAAL4830 | alkanesulfonate monooxygenase | 40840 | 5.81 | 4 | 11 | Frankia-5 | F201368.dat | 156 | 15 | 3 | 3 | 3 | 2 | 1 | 2 | 1 | 3 | 0.008% | 0 | 0 | 2 | 0 | 0 | 0.002% | -2.00 | -0.69 | 1.7E-02 | Blue |
| WP_011603149.1 | FRAAL1976 | hypothetical protein | 33937 | 4.74 | 2 | 5 | Frankia-4 | F201367.dat | 156 | 8 | 2 | 2 | 2 | 0 | 1 | 1 | 2 | 1 | 0.005% | 0 | 0 | 0 | 0 | 0 | 0.000% | -2.00 | -0.69 | 3.3E-02 | Blue |
| WP_011607830.1 | FRAAL6797 | ferredoxin | 11753 | 4.47 | 2 | 5 | Frankia-2 | F201365.dat | 87 | 24 | 2 | 2 | 2 | 1 | 2 | 0 | 1 | 1 | 0.015% | 0 | 0 | 0 | 0 | 0 | 0.000% | -2.00 | -0.69 | 3.3E-02 | Blue |
| WP_011602036.1 | FRAAL0800 | phosphomethylpyrimidine synthase | 69470 | 5.45 | 5 | 17 | Frankia-4 | F201367.dat | 124 | 7 | 4 | 4 | 4 | 3 | 2 | 2 | 4 | 2 | 0.007% | 0 | 0 | 4 | 0 | 0 | 0.002% | -2.00 | -0.69 | 3.9E-02 | Blue |
| WP_041938600.1 | FRAAL0057 | guanylate cyclase | 116146 | 6.30 | 15 | 54 | Frankia-1 | F201364.dat | 366 | 9 | 8 | 8 | 8 | 8 | 6 | 8 | 8 | 8 | 0.012% | 4 | 2 | 6 | 0 | 4 | 0.005% | -2.05 | -0.72 | 1.9E-03 | Blue |
| WP_011605819.1 | FRAAL4705 | GntR family transcriptional regulator | 47659 | 9.57 | 9 | 21 | Frankia-5 | F201368.dat | 138 | 15 | 5 | 5 | 5 | 4 | 2 | 2 | 3 | 5 | 0.012% | 0 | 2 | 3 | 0 | 0 | 0.004% | -2.10 | -0.74 | 1.7E-02 | Blue |
| WP_011605799.1 | FRAAL4683 | carbamoyl phosphate synthase large subunit | 117502 | 4.89 | 15 | 69 | Frankia-1 | F201364.dat | 554 | 16 | 13 | 13 | 14 | 14 | 7 | 10 | 8 | 10 | 0.015% | 4 | 0 | 10 | 2 | 4 | 0.006% | -2.16 | -0.77 | 1.1E-02 | Blue |
| WP_011603650.1 | FRAAL2491 | squalene-hopene cyclase | 81860 | 5.67 | 7 | 28 | Frankia-5 | F201368.dat | 179 | 7 | 5 | 5 | 5 | 4 | 4 | 3 | 5 | 5 | 0.009% | 1 | 1 | 0 | 2 | 3 | 0.003% | -2.17 | -0.77 | 1.1E-03 | Blue |
| WP_011605797.1 | FRAAL4681 | tryptophan synthase subunit beta | 40623 | 5.33 | 6 | 38 | Frankia-1 | F201364.dat | 438 | 32 | 7 | 6 | 7 | 7 | 4 | 7 | 5 | 5 | 0.025% | 2 | 0 | 4 | 1 | 3 | 0.008% | -2.20 | -0.79 | 2.3E-03 | Blue |
| WP_011603002.1 | FRAAL1823 | hydrogenase formation protein HypD | 40631 | 5.87 | 2 | 6 | Frankia-3 | F201366.dat | 43 | 6 | 2 | 2 | 2 | 2 | 0 | 2 | 1 | 1 | 0.005% | 0 | 0 | 0 | 0 | 0 | 0.000% | -2.20 | -0.79 | 2.2E-02 | Blue |
| WP_011605039.1 | FRAAL3904 | hypothetical protein | 100636 | 5.93 | 10 | 19 | Frankia-8 | F201371.dat | 184 | 5 | 4 | 4 | 4 | 3 | 3 | 3 | 3 | 3 | 0.005% | 0 | 0 | 4 | 0 | 0 | 0.001% | -2.22 | -0.80 | 1.7E-02 | Blue |
| WP_011607568.1 | FRAAL6528 | DNA repair protein RadA | 49269 | 8.83 | 8 | 27 | Frankia-5 | F201368.dat | 215 | 20 | 6 | 6 | 6 | 4 | 4 | 3 | 4 | 6 | 0.015% | 1 | 2 | 0 | 1 | 2 | 0.004% | -2.36 | -0.86 | 6.2E-04 | Blue |
| WP_011602211.1 | FRAAL0984 | porphobilinogen deaminase | 40490 | 5.73 | 7 | 27 | Frankia-4 | F201367.dat | 221 | 21 | 6 | 6 | 6 | 5 | 4 | 2 | 6 | 4 | 0.019% | 0 | 2 | 3 | 1 | 0 | 0.005% | -2.36 | -0.86 | 4.7E-03 | Blue |
| WP_011607007.1 | FRAAL5949 | homoserine kinase | 37029 | 5.33 | 2 | 7 | Frankia-1 | F201364.dat | 56 | 9 | 2 | 2 | 2 | 2 | 2 | 1 | 2 | 0 | 0.007% | 0 | 0 | 0 | 0 | 0 | 0.000% | -2.40 | -0.88 | 1.4E-02 | Blue |
| WP_041939751.1 | FRAAL5704 | ATP-binding protein | 51990 | 5.27 | 6 | 28 | Frankia-1 | F201364.dat | 194 | 16 | 5 | 5 | 5 | 5 | 3 | 4 | 6 | 4 | 0.015% | 1 | 1 | 1 | 2 | 1 | 0.004% | -2.45 | -0.90 | 3.8E-04 | Blue |
| WP_050997156.1 | FRAAL3981 | hypothetical protein | 36941 | 4.38 | 3 | 8 | Frankia-3 | F201366.dat | 108 | 17 | 3 | 3 | 3 | 2 | 0 | 3 | 1 | 2 | 0.008% | 0 | 0 | 0 | 0 | 0 | 0.000% | -2.60 | -0.96 | 1.5E-02 | Blue |
| WP_011606704.1 | FRAAL5625 | poly(3-hydroxybutyrate) depolymerase | 55149 | 8.46 | 3 | 13 | Frankia-5 | F201368.dat | 112 | 5 | 3 | 3 | 3 | 1 | 2 | 3 | 3 | 3 | 0.008% | 0 | 0 | 0 | 1 | 0 | 0.001% | -2.83 | -1.04 | 1.4E-03 | Blue |
| WP_041939968.1 | FRAAL6742 | hypothetical protein | 8180 | 4.27 | 6 | 45 | Frankia-4 | F201367.dat | 379 | 83 | 6 | 6 | 11 | 5 | 5 | 6 | 11 | 9 | 0.157% | 3 | 1 | 2 | 2 | 1 | 0.037% | -2.93 | -1.07 | 1.3E-03 | Blue |
| WP_011603497.1 | FRAAL2333 | precorrin-3B C17-methyltransferase | 58898 | 5.96 | 5 | 14 | Frankia-4 | F201367.dat | 92 | 6 | 3 | 3 | 3 | 3 | 2 | 3 | 3 | 2 | 0.008% | 0 | 0 | 1 | 0 | 0 | 0.001% | -3.00 | -1.10 | 4.5E-04 | Blue |
| WP_011607843.1 | FRAAL6809 | nitrogenase molybdenum-cofactor biosynthesis protein NifN | 60127 | 4.95 | 5 | 17 | Frankia-1 | F201364.dat | 184 | 11 | 5 | 5 | 5 | 5 | 2 | 4 | 2 | 3 | 0.009% | 1 | 0 | 0 | 0 | 0 | 0.001% | -3.50 | -1.25 | 1.0E-03 | Blue |
| WP_011605275.1 | FRAAL4147 | cytochrome C oxidase subunit I | 63598 | 8.61 | 7 | 37 | Frankia-1 | F201364.dat | 376 | 14 | 7 | 4 | 9 | 9 | 9 | 5 | 4 | 5 | 0.018% | 2 | 1 | 2 | 0 | 0 | 0.003% | -3.70 | -1.31 | 8.4E-04 | Blue |
| WP_011607844.1 | FRAAL6810 | nitrogenase iron-molybdenum cofactor biosynthesis protein NifE | 48492 | 5.15 | 10 | 38 | Frankia-4 | F201367.dat | 376 | 23 | 8 | 8 | 8 | 7 | 5 | 6 | 8 | 7 | 0.024% | 0 | 2 | 2 | 0 | 1 | 0.003% | -3.80 | -1.34 | 1.7E-05 | Blue |
| WP_011604986.1 | FRAAL3851 | serine/threonine protein kinase | 79799 | 6.46 | 4 | 15 | Frankia-3 | F201366.dat | 207 | 8 | 4 | 4 | 4 | 3 | 3 | 4 | 2 | 3 | 0.007% | 0 | 0 | 0 | 0 | 0 | 0.000% | -4.00 | -1.39 | 1.0E-04 | Blue |
| WP_041939984.1 | FRAAL6803 | nitrogen fixation protein NifB | 55488 | 5.18 | 21 | 96 | Frankia-1 | F201364.dat | 696 | 51 | 17 | 17 | 19 | 19 | 16 | 21 | 11 | 17 | 0.054% | 4 | 5 | 3 | 0 | 0 | 0.007% | -5.24 | -1.66 | 4.2E-05 | Blue |
| WP_011606542.1 | FRAAL5461 | epoxide hydrolase | 37942 | 5.30 | 7 | 25 | Frankia-4 | F201367.dat | 234 | 27 | 6 | 3 | 7 | 4 | 2 | 6 | 7 | 6 | 0.024% | 0 | 0 | 0 | 0 | 0 | 0.000% | -6.00 | -1.79 | 3.9E-04 | Blue |
| WP_011607848.1 | FRAAL6814 | homocitrate synthase | 43905 | 6.16 | 10 | 35 | Frankia-3 | F201366.dat | 385 | 33 | 10 | 10 | 12 | 7 | 4 | 12 | 5 | 6 | 0.028% | 0 | 0 | 1 | 0 | 0 | 0.001% | -6.50 | -1.87 | 8.7E-04 | Blue |
| WP_011601503.1 | FRAAL0245 | serine/threonine protein kinase | 52593 | 9.56 | 10 | 45 | Frankia-9 | F201372.dat | 222 | 15 | 6 | 6 | 6 | 3 | 2 | 3 | 4 | 5 | 0.012% | 5 | 4 | 7 | 6 | 6 | 0.018% | 1.50 | 0.41 | 7.9E-03 | Orange |
| WP_011606858.1 | FRAAL5786 | hypothetical protein | 11913 | 4.55 | 6 | 30 | Frankia-6 | F201369.dat | 290 | 48 | 5 | 5 | 5 | 2 | 2 | 3 | 1 | 3 | 0.033% | 5 | 2 | 4 | 4 | 4 | 0.054% | 1.50 | 0.41 | 1.6E-02 | Orange |
| WP_050997372.1 | FRAAL3261 | cation diffusion facilitator family transporter | 33789 | 4.76 | 5 | 30 | Frankia-7 | F201370.dat | 181 | 19 | 4 | 4 | 5 | 3 | 2 | 3 | 1 | 2 | 0.012% | 3 | 5 | 4 | 5 | 2 | 0.019% | 1.50 | 0.41 | 2.5E-02 | Orange |
| WP_011606787.1 | FRAAL5713 | arginine ABC transporter ATP-binding protein | 26513 | 5.99 | 11 | 92 | Frankia-9 | F201372.dat | 542 | 42 | 10 | 10 | 14 | 5 | 8 | 6 | 9 | 8 | 0.048% | 7 | 13 | 9 | 14 | 13 | 0.071% | 1.49 | 0.40 | 1.6E-02 | Orange |
| WP_041939920.1 | FRAAL6563 | hypothetical protein | 18950 | 9.98 | 7 | 77 | Frankia-8 | F201371.dat | 285 | 48 | 6 | 6 | 11 | 5 | 7 | 6 | 7 | 5 | 0.056% | 8 | 10 | 11 | 10 | 8 | 0.084% | 1.49 | 0.40 | 9.5E-04 | Orange |
| WP_011605402.1 | FRAAL4276 | NAD-dependent dehydratase | 22191 | 8.26 | 8 | 52 | Frankia-10 | F201373.dat | 365 | 45 | 6 | 6 | 7 | 3 | 3 | 4 | 5 | 5 | 0.032% | 5 | 6 | 7 | 7 | 7 | 0.049% | 1.48 | 0.39 | 2.0E-03 | Orange |
| WP_011607508.1 | FRAAL6463 | hypothetical protein | 26864 | 4.54 | 7 | 47 | Frankia-8 | F201371.dat | 342 | 33 | 7 | 7 | 7 | 4 | 4 | 4 | 3 | 3 | 0.024% | 4 | 9 | 7 | 5 | 4 | 0.036% | 1.48 | 0.39 | 3.2E-02 | Orange |
| WP_041938914.1 | FRAAL1422 | ABC transporter substrate-binding protein | 40640 | 5.82 | 14 | 156 | Frankia-9 | F201372.dat | 812 | 38 | 12 | 12 | 21 | 13 | 11 | 15 | 12 | 11 | 0.054% | 15 | 21 | 21 | 21 | 16 | 0.078% | 1.48 | 0.39 | 1.6E-03 | Orange |
| WP_041940775.1 | FRAAL4545 | hypothetical protein | 30751 | 5.87 | 6 | 37 | Frankia-10 | F201373.dat | 282 | 30 | 6 | 6 | 6 | 2 | 2 | 2 | 4 | 4 | 0.016% | 5 | 4 | 4 | 4 | 6 | 0.025% | 1.47 | 0.39 | 1.1E-02 | Orange |
| WP_050997388.1 | FRAAL3763 | sugar ABC transporter permease | 30860 | 11.59 | 3 | 32 | Frankia-6 | F201369.dat | 160 | 11 | 3 | 3 | 4 | 3 | 2 | 3 | 2 | 2 | 0.014% | 4 | 3 | 4 | 6 | 3 | 0.022% | 1.47 | 0.39 | 1.8E-02 | Orange |
| WP_011605401.1 | FRAAL4275 | methylmalonyl-CoA epimerase | 15001 | 5.69 | 4 | 32 | Frankia-10 | F201373.dat | 229 | 38 | 4 | 4 | 6 | 2 | 2 | 2 | 3 | 3 | 0.029% | 4 | 2 | 5 | 3 | 6 | 0.045% | 1.47 | 0.39 | 3.7E-02 | Orange |
| WP_011601859.1 | FRAAL0610 | recombination protein RecR | 21715 | 5.49 | 7 | 27 | Frankia-10 | F201373.dat | 190 | 30 | 4 | 4 | 4 | 2 | 2 | 2 | 1 | 3 | 0.016% | 3 | 3 | 4 | 3 | 4 | 0.026% | 1.47 | 0.38 | 8.7E-03 | Orange |
| WP_041939635.1 | FRAAL5104 | hypothetical protein | 44915 | 4.56 | 3 | 27 | Frankia-7 | F201370.dat | 209 | 9 | 3 | 3 | 4 | 2 | 2 | 3 | 2 | 1 | 0.008% | 3 | 4 | 3 | 4 | 3 | 0.013% | 1.47 | 0.38 | 8.7E-03 | Orange |
| WP_011601989.1 | FRAAL0748 | hypothetical protein | 26511 | 5.51 | 8 | 64 | Frankia-10 | F201373.dat | 422 | 42 | 8 | 8 | 9 | 5 | 4 | 6 | 4 | 6 | 0.034% | 4 | 9 | 8 | 9 | 9 | 0.050% | 1.47 | 0.38 | 1.5E-02 | Orange |
| WP_011604956.1 | FRAAL3819 | LysR family transcriptional regulator | 26057 | 5.19 | 4 | 27 | Frankia-7 | F201370.dat | 156 | 21 | 4 | 4 | 4 | 0 | 2 | 3 | 3 | 2 | 0.014% | 2 | 4 | 4 | 3 | 4 | 0.022% | 1.47 | 0.38 | 3.6E-02 | Orange |
| WP_011603124.1 | FRAAL1951 | hypothetical protein | 10639 | 6.55 | 7 | 76 | Frankia-10 | F201373.dat | 434 | 89 | 7 | 7 | 11 | 5 | 6 | 7 | 7 | 5 | 0.101% | 7 | 9 | 9 | 10 | 11 | 0.146% | 1.46 | 0.38 | 2.0E-03 | Orange |
| WP_011606683.1 | FRAAL5602 | limonene-1.2-epoxide hydrolase | 15334 | 4.45 | 6 | 66 | Frankia-10 | F201373.dat | 364 | 57 | 6 | 6 | 9 | 4 | 5 | 5 | 8 | 4 | 0.061% | 6 | 6 | 9 | 10 | 9 | 0.088% | 1.45 | 0.37 | 1.8E-02 | Orange |
| WP_041941001.1 | FRAAL5839 | Crp/Fnr family transcriptional regulator | 24962 | 8.25 | 12 | 159 | Frankia-7 | F201370.dat | 790 | 70 | 12 | 12 | 22 | 14 | 13 | 13 | 14 | 10 | 0.091% | 16 | 22 | 16 | 21 | 20 | 0.128% | 1.45 | 0.37 | 1.4E-03 | Orange |
| WP_011606630.1 | FRAAL5546 | methylase | 26736 | 4.71 | 9 | 61 | Frankia-10 | F201373.dat | 504 | 44 | 8 | 8 | 9 | 5 | 5 | 6 | 5 | 3 | 0.032% | 5 | 7 | 9 | 7 | 9 | 0.047% | 1.45 | 0.37 | 9.8E-03 | Orange |
| WP_011603285.1 | FRAAL2120 | hypothetical protein | 34279 | 6.88 | 10 | 83 | Frankia-9 | F201372.dat | 401 | 27 | 9 | 9 | 12 | 8 | 7 | 6 | 6 | 6 | 0.034% | 8 | 12 | 8 | 12 | 10 | 0.049% | 1.45 | 0.37 | 4.2E-03 | Orange |
| WP_011606879.1 | FRAAL5808 | phosphopantetheine adenylyltransferase | 17920 | 6.42 | 5 | 51 | Frankia-8 | F201371.dat | 337 | 49 | 5 | 5 | 6 | 3 | 5 | 5 | 4 | 3 | 0.040% | 4 | 6 | 6 | 7 | 8 | 0.058% | 1.44 | 0.36 | 1.3E-02 | Orange |
| WP_050997220.1 | FRAAL5218 | hypothetical protein | 64041 | 6.04 | 4 | 29 | Frankia-6 | F201369.dat | 228 | 9 | 4 | 4 | 4 | 2 | 3 | 2 | 1 | 3 | 0.006% | 4 | 3 | 4 | 3 | 4 | 0.009% | 1.44 | 0.36 | 1.2E-02 | Orange |
| WP_041938811.1 | FRAAL0925 | membrane protein | 14889 | 9.52 | 3 | 46 | Frankia-7 | F201370.dat | 203 | 28 | 3 | 3 | 6 | 3 | 4 | 5 | 3 | 3 | 0.043% | 4 | 6 | 7 | 5 | 6 | 0.063% | 1.43 | 0.36 | 7.5E-03 | Orange |
| WP_011602827.1 | FRAAL1638 | hypothetical protein | 28565 | 5.09 | 9 | 80 | Frankia-7 | F201370.dat | 483 | 54 | 9 | 9 | 12 | 7 | 5 | 8 | 7 | 5 | 0.040% | 8 | 12 | 9 | 9 | 10 | 0.057% | 1.43 | 0.36 | 3.8E-03 | Orange |
| WP_011607002.1 | FRAAL5943 | hypothetical protein | 22304 | 5.23 | 6 | 58 | Frankia-8 | F201371.dat | 305 | 27 | 6 | 6 | 8 | 6 | 4 | 5 | 5 | 3 | 0.037% | 7 | 6 | 8 | 7 | 7 | 0.053% | 1.43 | 0.36 | 2.1E-03 | Orange |
| WP_041939783.1 | FRAAL5930 | ATP synthase F0F1 subunit epsilon | 8813 | 4.82 | 2 | 24 | Frankia-10 | F201373.dat | 170 | 55 | 2 | 2 | 3 | 2 | 2 | 2 | 2 | 1 | 0.036% | 4 | 2 | 3 | 3 | 3 | 0.057% | 1.43 | 0.36 | 1.7E-02 | Orange |
| WP_011601422.1 | FRAAL0161 | extradiol ring-cleavage dioxygenase | 28519 | 5.30 | 3 | 24 | Frankia-8 | F201371.dat | 178 | 15 | 3 | 3 | 4 | 3 | 2 | 2 | 1 | 1 | 0.011% | 2 | 3 | 4 | 3 | 3 | 0.018% | 1.43 | 0.36 | 2.2E-02 | Orange |
| WP_041939523.1 | FRAAL4508 | urate oxidase | 32617 | 5.54 | 6 | 24 | Frankia-8 | F201371.dat | 202 | 17 | 4 | 4 | 4 | 2 | 2 | 2 | 2 | 1 | 0.010% | 3 | 2 | 4 | 4 | 2 | 0.015% | 1.43 | 0.36 | 3.2E-02 | Orange |
| WP_009399443.1 | FRAAL4590 | sporulation protein | 34799 | 10.57 | 8 | 36 | Frankia-10 | F201373.dat | 202 | 19 | 5 | 5 | 6 | 3 | 3 | 3 | 3 | 2 | 0.014% | 5 | 3 | 4 | 4 | 6 | 0.021% | 1.42 | 0.35 | 1.5E-02 | Orange |
| WP_011601969.1 | FRAAL0728 | ABC transporter | 33597 | 10.24 | 9 | 48 | Frankia-6 | F201369.dat | 413 | 33 | 7 | 7 | 7 | 4 | 4 | 4 | 4 | 3 | 0.020% | 7 | 6 | 5 | 6 | 5 | 0.029% | 1.42 | 0.35 | 2.0E-03 | Orange |
| WP_041940469.1 | FRAAL2861 | haloacid dehalogenase | 28785 | 4.71 | 5 | 19 | Frankia-7 | F201370.dat | 152 | 14 | 3 | 3 | 3 | 1 | 2 | 1 | 2 | 1 | 0.009% | 2 | 3 | 3 | 2 | 2 | 0.014% | 1.42 | 0.35 | 3.3E-02 | Orange |
| WP_009738243.1 | FRAAL3401 | dioxygenase | 19243 | 6.41 | 4 | 19 | Frankia-9 | F201372.dat | 145 | 21 | 3 | 3 | 3 | 1 | 2 | 1 | 1 | 2 | 0.013% | 2 | 3 | 3 | 3 | 1 | 0.021% | 1.42 | 0.35 | 4.5E-02 | Orange |
| WP_011606345.1 | FRAAL5254 | hypothetical protein | 17207 | 10.18 | 8 | 101 | Frankia-8 | F201371.dat | 458 | 37 | 8 | 8 | 13 | 8 | 7 | 10 | 9 | 7 | 0.085% | 10 | 12 | 13 | 14 | 11 | 0.117% | 1.41 | 0.35 | 1.6E-03 | Orange |
| WP_011606853.1 | FRAAL5781 | peptidase | 34426 | 11.20 | 5 | 72 | Frankia-6 | F201369.dat | 232 | 20 | 5 | 5 | 8 | 5 | 5 | 7 | 7 | 5 | 0.030% | 8 | 10 | 6 | 10 | 9 | 0.042% | 1.41 | 0.34 | 7.0E-03 | Orange |
| WP_011607544.1 | FRAAL6503 | hypothetical protein | 16928 | 4.81 | 3 | 31 | Frankia-7 | F201370.dat | 173 | 27 | 3 | 3 | 4 | 2 | 3 | 2 | 2 | 3 | 0.025% | 3 | 4 | 3 | 4 | 5 | 0.038% | 1.41 | 0.34 | 1.2E-02 | Orange |
| WP_009741343.1 | FRAAL2772 | DNA-binding protein | 7457 | 10.48 | 2 | 31 | Frankia-9 | F201372.dat | 140 | 31 | 2 | 2 | 5 | 2 | 3 | 1 | 3 | 3 | 0.057% | 4 | 2 | 4 | 5 | 4 | 0.086% | 1.41 | 0.34 | 2.9E-02 | Orange |
| WP_041938792.1 | FRAAL0865 | methyltransferase type 11 | 21980 | 8.74 | 7 | 31 | Frankia-10 | F201373.dat | 310 | 38 | 6 | 6 | 6 | 2 | 2 | 3 | 2 | 3 | 0.019% | 3 | 3 | 4 | 3 | 6 | 0.029% | 1.41 | 0.34 | 3.5E-02 | Orange |
| WP_011603332.1 | FRAAL2167 | hypothetical protein | 20446 | 9.30 | 5 | 43 | Frankia-10 | F201373.dat | 180 | 24 | 5 | 5 | 6 | 3 | 3 | 2 | 4 | 5 | 0.030% | 5 | 5 | 5 | 5 | 6 | 0.043% | 1.41 | 0.34 | 9.1E-03 | Orange |
| WP_011602328.1 | FRAAL1121 | carbohydrate kinase | 53418 | 5.59 | 10 | 55 | Frankia-9 | F201372.dat | 232 | 18 | 7 | 7 | 7 | 4 | 6 | 5 | 4 | 3 | 0.015% | 6 | 7 | 7 | 7 | 6 | 0.021% | 1.41 | 0.34 | 3.4E-03 | Orange |
| WP_041939670.1 | FRAAL5288 | GlcNAc-PI de-N-acetylase | 32939 | 5.80 | 8 | 67 | Frankia-7 | F201370.dat | 340 | 32 | 7 | 7 | 9 | 4 | 6 | 7 | 6 | 4 | 0.029% | 6 | 9 | 9 | 8 | 8 | 0.041% | 1.41 | 0.34 | 6.3E-03 | Orange |
| WP_011606763.1 | FRAAL5690 | hypothetical protein | 12296 | 9.21 | 15 | 379 | Frankia-10 | F201373.dat | 887 | 81 | 14 | 14 | 60 | 24 | 28 | 29 | 37 | 39 | 0.456% | 33 | 31 | 42 | 56 | 60 | 0.608% | 1.40 | 0.34 | 4.1E-02 | Orange |
| WP_041938704.1 | FRAAL0496 | polyketide cyclase | 15603 | 9.85 | 9 | 98 | Frankia-8 | F201371.dat | 548 | 80 | 9 | 9 | 14 | 6 | 7 | 8 | 10 | 9 | 0.091% | 10 | 10 | 14 | 11 | 13 | 0.125% | 1.40 | 0.34 | 5.1E-03 | Orange |
| WP_011605174.1 | FRAAL4043 | polyketide cyclase | 17829 | 10.17 | 6 | 26 | Frankia-9 | F201372.dat | 140 | 40 | 5 | 5 | 5 | 2 | 2 | 1 | 3 | 2 | 0.020% | 3 | 2 | 3 | 5 | 3 | 0.030% | 1.40 | 0.34 | 3.8E-02 | Orange |
| WP_011601735.1 | FRAAL0482 | alpha/beta hydrolase | 29965 | 6.24 | 14 | 141 | Frankia-7 | F201370.dat | 564 | 49 | 11 | 11 | 19 | 10 | 11 | 13 | 11 | 13 | 0.069% | 14 | 19 | 17 | 18 | 15 | 0.093% | 1.40 | 0.33 | 9.7E-04 | Orange |
| WP_011605001.1 | FRAAL3866 | ABC transporter substrate-binding protein | 58863 | 8.73 | 9 | 57 | Frankia-7 | F201370.dat | 454 | 20 | 9 | 9 | 9 | 5 | 4 | 5 | 4 | 5 | 0.014% | 7 | 9 | 6 | 7 | 5 | 0.019% | 1.39 | 0.33 | 9.0E-03 | Orange |
| WP_011607307.1 | FRAAL6257 | serine O-acetyltransferase | 24743 | 9.91 | 9 | 155 | Frankia-8 | F201371.dat | 519 | 57 | 9 | 9 | 20 | 13 | 11 | 13 | 14 | 13 | 0.092% | 17 | 18 | 20 | 19 | 17 | 0.124% | 1.39 | 0.33 | 5.1E-05 | Orange |
| WP_011603598.1 | FRAAL2441 | pyridoxamine 5-phosphate oxidase | 16967 | 6.75 | 6 | 45 | Frankia-8 | F201371.dat | 344 | 40 | 5 | 5 | 6 | 3 | 4 | 3 | 4 | 4 | 0.038% | 6 | 5 | 6 | 5 | 5 | 0.054% | 1.39 | 0.33 | 2.5E-03 | Orange |
| WP_011603480.1 | FRAAL2316 | chemical-damaging agent resistance protein C | 20536 | 4.62 | 11 | 100 | Frankia-7 | F201370.dat | 706 | 60 | 11 | 11 | 14 | 4 | 10 | 8 | 10 | 9 | 0.071% | 10 | 14 | 12 | 12 | 11 | 0.097% | 1.39 | 0.33 | 1.2E-02 | Orange |
| WP_011603489.1 | FRAAL2325 | 3-(3-hydroxyphenyl)propionate hydroxylase | 59477 | 6.29 | 20 | 143 | Frankia-6 | F201369.dat | 768 | 41 | 16 | 16 | 21 | 13 | 14 | 10 | 11 | 11 | 0.035% | 21 | 17 | 17 | 15 | 14 | 0.048% | 1.39 | 0.33 | 3.7E-03 | Orange |
| WP_011602322.1 | FRAAL1115 | 30S ribosomal protein S9 | 14993 | 10.60 | 14 | 241 | Frankia-8 | F201371.dat | 624 | 79 | 13 | 13 | 34 | 19 | 18 | 19 | 22 | 22 | 0.238% | 21 | 23 | 34 | 33 | 30 | 0.317% | 1.39 | 0.33 | 9.0E-03 | Orange |
| WP_041939006.1 | FRAAL1850 | two-component system response regulator | 26546 | 5.16 | 11 | 107 | Frankia-8 | F201371.dat | 483 | 40 | 9 | 9 | 14 | 9 | 10 | 7 | 9 | 9 | 0.059% | 12 | 12 | 14 | 12 | 13 | 0.080% | 1.39 | 0.33 | 1.6E-04 | Orange |
| WP_011607656.1 | FRAAL6619 | ABC transporter ATP-binding protein | 36449 | 8.11 | 10 | 64 | Frankia-6 | F201369.dat | 417 | 38 | 9 | 9 | 10 | 7 | 4 | 5 | 5 | 5 | 0.025% | 10 | 10 | 6 | 7 | 5 | 0.035% | 1.39 | 0.33 | 3.4E-02 | Orange |
| WP_011605775.1 | FRAAL4659 | dimethylmenaquinone methyltransferase | 22688 | 5.73 | 9 | 157 | Frankia-9 | F201372.dat | 615 | 51 | 9 | 9 | 20 | 10 | 13 | 11 | 15 | 16 | 0.102% | 17 | 15 | 22 | 20 | 18 | 0.137% | 1.39 | 0.33 | 5.8E-03 | Orange |
| WP_011606223.1 | FRAAL5115 | hypothetical protein | 22069 | 4.69 | 8 | 52 | Frankia-10 | F201373.dat | 434 | 44 | 7 | 7 | 7 | 2 | 6 | 3 | 5 | 5 | 0.034% | 6 | 5 | 6 | 7 | 7 | 0.047% | 1.38 | 0.33 | 2.1E-02 | Orange |
| WP_011602373.1 | FRAAL1171 | phosphoesterase | 22168 | 5.55 | 3 | 21 | Frankia-9 | F201372.dat | 111 | 22 | 3 | 3 | 3 | 1 | 2 | 1 | 2 | 2 | 0.013% | 3 | 3 | 2 | 3 | 2 | 0.020% | 1.38 | 0.33 | 3.3E-02 | Orange |
| WP_011601669.1 | FRAAL0413 | hypothetical protein | 14695 | 4.82 | 13 | 140 | Frankia-9 | F201372.dat | 639 | 98 | 11 | 11 | 16 | 11 | 11 | 12 | 13 | 11 | 0.141% | 12 | 19 | 17 | 16 | 18 | 0.188% | 1.38 | 0.32 | 2.7E-03 | Orange |
| WP_050997268.1 | FRAAL6318 | hypothetical protein | 26649 | 5.44 | 6 | 66 | Frankia-10 | F201373.dat | 278 | 26 | 6 | 6 | 9 | 5 | 6 | 5 | 5 | 6 | 0.036% | 7 | 6 | 8 | 9 | 9 | 0.049% | 1.38 | 0.32 | 3.6E-03 | Orange |
| WP_011605771.1 | FRAAL4655 | hypothetical protein | 44057 | 6.07 | 14 | 104 | Frankia-7 | F201370.dat | 543 | 28 | 11 | 11 | 14 | 10 | 8 | 7 | 7 | 11 | 0.035% | 10 | 14 | 15 | 10 | 12 | 0.047% | 1.38 | 0.32 | 1.2E-02 | Orange |
| WP_011604604.1 | FRAAL3460 | 3-ketoacyl-ACP reductase | 25729 | 5.94 | 12 | 85 | Frankia-9 | F201372.dat | 457 | 39 | 10 | 10 | 13 | 6 | 6 | 6 | 8 | 9 | 0.049% | 7 | 11 | 10 | 13 | 9 | 0.065% | 1.38 | 0.32 | 1.7E-02 | Orange |
| WP_011605875.1 | FRAAL4760 | transcriptional regulator | 52515 | 6.42 | 7 | 28 | Frankia-6 | F201369.dat | 269 | 12 | 4 | 4 | 4 | 1 | 2 | 3 | 2 | 3 | 0.007% | 4 | 3 | 3 | 4 | 3 | 0.011% | 1.38 | 0.32 | 2.2E-02 | Orange |
| WP_041939032.1 | FRAAL5087 | glycerol acyltransferase | 26455 | 9.65 | 6 | 28 | Frankia-8 | F201371.dat | 93 | 14 | 3 | 3 | 3 | 2 | 3 | 1 | 2 | 3 | 0.015% | 3 | 3 | 3 | 4 | 4 | 0.022% | 1.38 | 0.32 | 2.2E-02 | Orange |
| WP_041938785.1 | FRAAL0835 | universal stress protein | 16793 | 6.34 | 4 | 28 | Frankia-8 | F201371.dat | 223 | 36 | 4 | 4 | 4 | 2 | 1 | 2 | 3 | 3 | 0.023% | 2 | 3 | 4 | 4 | 4 | 0.034% | 1.38 | 0.32 | 3.0E-02 | Orange |
| WP_041939508.1 | FRAAL4442 | peroxiredoxin | 16146 | 5.56 | 11 | 149 | Frankia-10 | F201373.dat | 563 | 72 | 10 | 10 | 19 | 10 | 13 | 12 | 13 | 14 | 0.137% | 15 | 17 | 18 | 18 | 19 | 0.181% | 1.37 | 0.32 | 4.0E-04 | Orange |
| WP_041939945.1 | FRAAL6664 | phosphoribosylaminoimidazole carboxylase | 17426 | 4.96 | 5 | 92 | Frankia-9 | F201372.dat | 353 | 42 | 5 | 5 | 10 | 6 | 9 | 8 | 6 | 9 | 0.078% | 11 | 11 | 10 | 10 | 12 | 0.104% | 1.37 | 0.32 | 1.6E-03 | Orange |
| WP_011607575.1 | FRAAL6535 | phosphate ABC transporter substrate-binding protein | 35983 | 5.02 | 14 | 118 | Frankia-7 | F201370.dat | 752 | 56 | 13 | 13 | 15 | 11 | 8 | 10 | 9 | 11 | 0.049% | 13 | 15 | 14 | 13 | 14 | 0.065% | 1.37 | 0.32 | 2.1E-04 | Orange |
| WP_011601396.1 | FRAAL0132 | valine dehydrogenase (ValDH) | 37482 | 5.51 | 8 | 54 | Frankia-8 | F201371.dat | 293 | 28 | 7 | 7 | 7 | 5 | 4 | 4 | 5 | 4 | 0.021% | 7 | 6 | 7 | 6 | 6 | 0.029% | 1.37 | 0.32 | 1.4E-03 | Orange |
| WP_011603421.1 | FRAAL2254 | glycerol acyltransferase | 29309 | 9.70 | 5 | 35 | Frankia-8 | F201371.dat | 198 | 22 | 5 | 5 | 5 | 3 | 2 | 3 | 3 | 3 | 0.017% | 5 | 3 | 5 | 3 | 5 | 0.024% | 1.37 | 0.31 | 2.3E-02 | Orange |
| WP_011604927.1 | FRAAL3789 | thioesterase | 20629 | 5.13 | 3 | 42 | Frankia-3 | F201366.dat | 216 | 27 | 3 | 3 | 4 | 4 | 3 | 4 | 3 | 3 | 0.029% | 5 | 5 | 5 | 5 | 5 | 0.041% | 1.36 | 0.31 | 4.6E-03 | Orange |
| WP_009742300.1 | FRAAL5827 | transcriptional regulator | 9840 | 8.16 | 7 | 127 | Frankia-9 | F201372.dat | 472 | 80 | 7 | 7 | 16 | 9 | 11 | 10 | 11 | 12 | 0.192% | 13 | 13 | 14 | 16 | 18 | 0.253% | 1.36 | 0.31 | 2.5E-03 | Orange |
| WP_041938893.1 | FRAAL1314 | XRE family transcriptional regulator | 18773 | 6.86 | 4 | 23 | Frankia-10 | F201373.dat | 152 | 25 | 3 | 3 | 3 | 2 | 2 | 2 | 1 | 2 | 0.017% | 2 | 2 | 3 | 4 | 3 | 0.025% | 1.36 | 0.31 | 4.0E-02 | Orange |
| WP_050997314.1 | FRAAL0740 | hypothetical protein | 20177 | 4.54 | 4 | 23 | Frankia-10 | F201373.dat | 158 | 26 | 3 | 3 | 4 | 2 | 2 | 1 | 3 | 1 | 0.016% | 2 | 3 | 3 | 2 | 4 | 0.023% | 1.36 | 0.31 | 4.8E-02 | Orange |
| WP_050997059.1 | FRAAL2290 | LuxR family transcriptional regulator | 24272 | 5.26 | 5 | 30 | Frankia-9 | F201372.dat | 204 | 19 | 4 | 4 | 4 | 2 | 2 | 3 | 2 | 3 | 0.018% | 4 | 2 | 4 | 4 | 4 | 0.025% | 1.35 | 0.30 | 2.5E-02 | Orange |
| WP_011606320.1 | FRAAL5225 | methionyl-tRNA formyltransferase | 34376 | 5.55 | 8 | 77 | Frankia-8 | F201371.dat | 350 | 31 | 7 | 7 | 10 | 7 | 6 | 5 | 7 | 7 | 0.033% | 8 | 9 | 10 | 8 | 10 | 0.044% | 1.35 | 0.30 | 1.3E-03 | Orange |
| WP_011602490.1 | FRAAL1291 | PPOX class F420-dependent enzyme | 17811 | 5.74 | 4 | 37 | Frankia-9 | F201372.dat | 81 | 17 | 2 | 2 | 5 | 3 | 2 | 3 | 4 | 3 | 0.030% | 4 | 4 | 4 | 5 | 5 | 0.042% | 1.35 | 0.30 | 8.7E-03 | Orange |
| WP_011604701.1 | FRAAL3560 | short-chain dehydrogenase | 26386 | 5.10 | 6 | 37 | Frankia-8 | F201371.dat | 283 | 35 | 5 | 5 | 5 | 4 | 3 | 3 | 3 | 2 | 0.020% | 3 | 5 | 5 | 5 | 4 | 0.028% | 1.35 | 0.30 | 1.4E-02 | Orange |
| WP_011607358.1 | FRAAL6310 | NUDIX hydrolase | 18203 | 4.83 | 4 | 37 | Frankia-7 | F201370.dat | 243 | 25 | 4 | 4 | 6 | 4 | 2 | 4 | 2 | 3 | 0.029% | 3 | 6 | 5 | 4 | 4 | 0.041% | 1.35 | 0.30 | 3.6E-02 | Orange |
| WP_041939266.1 | FRAAL3015 | oxidoreductase | 37553 | 5.11 | 5 | 51 | Frankia-8 | F201371.dat | 179 | 17 | 5 | 5 | 7 | 3 | 4 | 4 | 5 | 5 | 0.020% | 5 | 5 | 7 | 7 | 6 | 0.027% | 1.35 | 0.30 | 7.5E-03 | Orange |
| WP_011601578.1 | FRAAL0322 | short-chain dehydrogenase | 28255 | 5.55 | 8 | 65 | Frankia-10 | F201373.dat | 347 | 36 | 7 | 7 | 9 | 5 | 5 | 6 | 5 | 6 | 0.034% | 5 | 6 | 9 | 9 | 9 | 0.045% | 1.34 | 0.30 | 2.3E-02 | Orange |
| WP_041940124.1 | FRAAL0762 | short-chain dehydrogenase | 26328 | 5.37 | 6 | 53 | Frankia-3 | F201366.dat | 362 | 24 | 5 | 5 | 5 | 5 | 5 | 5 | 4 | 3 | 0.030% | 7 | 7 | 7 | 5 | 5 | 0.040% | 1.33 | 0.29 | 1.1E-02 | Orange |
| WP_011606556.1 | FRAAL5471 | malonic semialdehyde reductase | 22615 | 4.92 | 14 | 130 | Frankia-8 | F201371.dat | 611 | 74 | 10 | 10 | 17 | 10 | 14 | 8 | 14 | 9 | 0.087% | 14 | 14 | 17 | 14 | 16 | 0.112% | 1.33 | 0.29 | 1.1E-02 | Orange |
| WP_009739579.1 | FRAAL2187 | transcriptional regulator MraZ | 16058 | 5.25 | 4 | 32 | Frankia-10 | F201373.dat | 153 | 33 | 4 | 4 | 4 | 2 | 2 | 3 | 3 | 3 | 0.029% | 4 | 4 | 3 | 4 | 4 | 0.040% | 1.33 | 0.29 | 1.7E-02 | Orange |
| WP_041939104.1 | FRAAL2315 | protein-disulfide isomerase | 22281 | 5.21 | 7 | 39 | Frankia-9 | F201372.dat | 296 | 31 | 5 | 5 | 5 | 4 | 2 | 4 | 3 | 3 | 0.026% | 4 | 5 | 3 | 5 | 6 | 0.035% | 1.33 | 0.29 | 2.9E-02 | Orange |
| WP_011604103.1 | FRAAL2957 | hypothetical protein | 31315 | 4.85 | 10 | 67 | Frankia-10 | F201373.dat | 540 | 61 | 10 | 6 | 11 | 5 | 5 | 5 | 8 | 5 | 0.032% | 6 | 7 | 7 | 8 | 11 | 0.042% | 1.33 | 0.29 | 3.5E-02 | Orange |
| WP_011604900.1 | FRAAL3762 | ABC transporter ATP-binding protein | 43431 | 6.27 | 22 | 409 | Frankia-7 | F201370.dat | 1455 | 65 | 21 | 21 | 52 | 35 | 35 | 36 | 37 | 32 | 0.144% | 41 | 52 | 48 | 51 | 42 | 0.181% | 1.33 | 0.28 | 6.1E-04 | Orange |
| WP_009740537.1 | FRAAL1069 | 50S ribosomal protein L7/L12 | 13371 | 4.57 | 9 | 104 | Frankia-6 | F201369.dat | 503 | 60 | 9 | 9 | 16 | 8 | 11 | 10 | 9 | 6 | 0.117% | 16 | 11 | 11 | 11 | 11 | 0.151% | 1.33 | 0.28 | 2.1E-02 | Orange |
| WP_041941131.1 | FRAAL6600 | zinc-binding alcohol dehydrogenase | 32242 | 5.41 | 12 | 85 | Frankia-9 | F201372.dat | 350 | 35 | 9 | 9 | 11 | 6 | 7 | 7 | 9 | 7 | 0.040% | 10 | 10 | 8 | 11 | 10 | 0.051% | 1.32 | 0.28 | 2.8E-03 | Orange |
| WP_041939632.1 | FRAAL5090 | hypothetical protein | 44542 | 5.56 | 11 | 85 | Frankia-8 | F201371.dat | 377 | 22 | 8 | 8 | 12 | 7 | 6 | 10 | 7 | 6 | 0.029% | 8 | 10 | 12 | 9 | 10 | 0.037% | 1.32 | 0.28 | 1.5E-02 | Orange |
| WP_011606689.1 | FRAAL5608 | alkyl sulfatase | 16685 | 5.25 | 9 | 85 | Frankia-8 | F201371.dat | 470 | 69 | 9 | 9 | 12 | 7 | 9 | 6 | 7 | 7 | 0.077% | 7 | 9 | 12 | 9 | 12 | 0.099% | 1.32 | 0.28 | 2.2E-02 | Orange |
| WP_041939901.1 | FRAAL6468 | hypothetical protein | 32605 | 11.55 | 3 | 34 | Frankia-7 | F201370.dat | 175 | 15 | 3 | 3 | 4 | 3 | 3 | 2 | 3 | 3 | 0.015% | 5 | 4 | 3 | 3 | 5 | 0.021% | 1.32 | 0.27 | 3.2E-02 | Orange |
| WP_041938807.1 | FRAAL0905 | ammonium transporter | 44253 | 6.14 | 2 | 115 | Frankia-4 | F201367.dat | 222 | 11 | 2 | 2 | 9 | 11 | 7 | 13 | 9 | 9 | 0.040% | 12 | 13 | 15 | 14 | 12 | 0.050% | 1.31 | 0.27 | 1.0E-02 | Orange |
| WP_011607592.1 | FRAAL6552 | translation initiation inhibitor | 15907 | 5.01 | 3 | 27 | Frankia-9 | F201372.dat | 166 | 36 | 3 | 3 | 4 | 2 | 2 | 3 | 2 | 2 | 0.025% | 2 | 4 | 3 | 4 | 3 | 0.034% | 1.31 | 0.27 | 4.0E-02 | Orange |
| WP_011606917.1 | FRAAL5850 | acetolactate synthase | 19045 | 9.58 | 12 | 87 | Frankia-8 | F201371.dat | 608 | 74 | 11 | 11 | 13 | 9 | 9 | 6 | 8 | 5 | 0.069% | 8 | 8 | 13 | 10 | 11 | 0.088% | 1.31 | 0.27 | 3.5E-02 | Orange |
| WP_011602302.1 | FRAAL1086 | 50S ribosomal protein L22 | 15309 | 10.83 | 8 | 117 | Frankia-10 | F201373.dat | 361 | 43 | 7 | 7 | 17 | 10 | 10 | 10 | 10 | 10 | 0.117% | 11 | 14 | 10 | 15 | 17 | 0.147% | 1.31 | 0.27 | 1.7E-02 | Orange |
| WP_041939734.1 | FRAAL5606 | NUDIX hydrolase | 32436 | 5.92 | 14 | 117 | Frankia-8 | F201371.dat | 723 | 48 | 12 | 12 | 16 | 7 | 10 | 13 | 10 | 10 | 0.055% | 9 | 12 | 16 | 15 | 15 | 0.070% | 1.31 | 0.27 | 3.3E-02 | Orange |
| WP_041938798.1 | FRAAL0883 | 3-oxoacyl-ACP reductase | 25782 | 5.27 | 11 | 147 | Frankia-7 | F201370.dat | 574 | 51 | 9 | 9 | 18 | 14 | 12 | 12 | 12 | 13 | 0.087% | 14 | 18 | 18 | 18 | 16 | 0.110% | 1.31 | 0.27 | 7.7E-04 | Orange |
| WP_011607545.1 | FRAAL6504 | isopentenyl-diphosphate delta isomerase | 21076 | 4.91 | 5 | 50 | Frankia-8 | F201371.dat | 337 | 34 | 5 | 5 | 8 | 4 | 3 | 5 | 4 | 5 | 0.036% | 6 | 4 | 8 | 6 | 5 | 0.046% | 1.31 | 0.27 | 3.4E-02 | Orange |
| WP_009737569.1 | FRAAL4338 | bacterioferritin | 18412 | 4.68 | 7 | 73 | Frankia-9 | F201372.dat | 325 | 36 | 7 | 7 | 10 | 6 | 6 | 6 | 6 | 7 | 0.060% | 7 | 9 | 8 | 10 | 8 | 0.077% | 1.31 | 0.27 | 3.4E-03 | Orange |
| WP_011602310.1 | FRAAL1096 | 50S ribosomal protein L6 | 19249 | 10.17 | 18 | 255 | Frankia-9 | F201372.dat | 914 | 84 | 16 | 16 | 33 | 22 | 24 | 22 | 24 | 18 | 0.204% | 23 | 29 | 29 | 33 | 31 | 0.254% | 1.30 | 0.27 | 4.0E-03 | Orange |
| WP_011602185.1 | FRAAL0956 | DNA-binding protein | 32453 | 6.66 | 14 | 119 | Frankia-8 | F201371.dat | 599 | 55 | 11 | 11 | 15 | 10 | 10 | 10 | 12 | 9 | 0.056% | 12 | 13 | 15 | 15 | 13 | 0.071% | 1.30 | 0.27 | 1.2E-03 | Orange |
| WP_011605661.1 | FRAAL4543 | cation diffusion facilitator transporter | 33386 | 5.21 | 6 | 59 | Frankia-9 | F201372.dat | 341 | 20 | 6 | 6 | 7 | 4 | 4 | 6 | 6 | 5 | 0.027% | 7 | 8 | 6 | 7 | 6 | 0.034% | 1.30 | 0.26 | 7.5E-03 | Orange |
| WP_011606710.1 | FRAAL5631 | branched-chain amino acid ABC transporter substrate-binding protein | 43116 | 9.08 | 20 | 190 | Frankia-9 | F201372.dat | 943 | 56 | 18 | 18 | 26 | 17 | 17 | 15 | 19 | 14 | 0.068% | 25 | 22 | 17 | 26 | 18 | 0.084% | 1.30 | 0.26 | 1.6E-02 | Orange |
| WP_041939971.1 | FRAAL6753 | cell division protein FtsI | 50598 | 5.76 | 13 | 75 | Frankia-7 | F201370.dat | 444 | 30 | 8 | 8 | 10 | 4 | 6 | 7 | 8 | 7 | 0.023% | 8 | 10 | 8 | 9 | 8 | 0.029% | 1.30 | 0.26 | 1.2E-02 | Orange |
| WP_011605347.1 | FRAAL4220 | heme ABC transporter ATP-binding protein | 57991 | 5.53 | 9 | 52 | Frankia-9 | F201372.dat | 324 | 19 | 7 | 7 | 7 | 6 | 4 | 4 | 4 | 4 | 0.014% | 7 | 5 | 5 | 7 | 6 | 0.017% | 1.30 | 0.26 | 1.4E-02 | Orange |
| WP_011602280.1 | FRAAL1053 | protease | 30891 | 9.78 | 7 | 68 | Frankia-8 | F201371.dat | 295 | 34 | 6 | 6 | 9 | 7 | 7 | 6 | 4 | 5 | 0.034% | 7 | 9 | 9 | 7 | 7 | 0.043% | 1.29 | 0.26 | 1.5E-02 | Orange |
| WP_011603508.1 | FRAAL2344 | carbamoyl-phosphate synthase subunit L | 89961 | 4.98 | 8 | 29 | Frankia-10 | F201373.dat | 181 | 5 | 4 | 4 | 4 | 2 | 3 | 2 | 2 | 3 | 0.005% | 3 | 2 | 4 | 4 | 4 | 0.006% | 1.29 | 0.26 | 4.5E-02 | Orange |
| WP_011606083.1 | FRAAL4969 | indole-3-glycerol phosphate synthase | 28011 | 5.34 | 13 | 109 | Frankia-10 | F201373.dat | 456 | 37 | 10 | 10 | 15 | 9 | 8 | 10 | 10 | 10 | 0.060% | 10 | 13 | 11 | 13 | 15 | 0.075% | 1.29 | 0.25 | 7.0E-03 | Orange |
| WP_011606224.1 | FRAAL5116 | rubrerythrin | 15846 | 4.85 | 11 | 125 | Frankia-9 | F201372.dat | 529 | 52 | 9 | 9 | 15 | 10 | 12 | 11 | 13 | 8 | 0.122% | 13 | 10 | 17 | 15 | 16 | 0.151% | 1.29 | 0.25 | 2.7E-02 | Orange |
| WP_009742365.1 | FRAAL5754 | 30S ribosomal protein S15 | 10466 | 10.87 | 12 | 191 | Frankia-8 | F201371.dat | 519 | 66 | 11 | 11 | 25 | 16 | 16 | 17 | 19 | 15 | 0.283% | 21 | 14 | 25 | 22 | 26 | 0.348% | 1.28 | 0.25 | 2.7E-02 | Orange |
| WP_011604153.1 | FRAAL3003 | hypothetical protein | 31297 | 5.45 | 12 | 136 | Frankia-10 | F201373.dat | 591 | 60 | 11 | 7 | 17 | 10 | 14 | 11 | 13 | 11 | 0.067% | 15 | 15 | 15 | 15 | 17 | 0.083% | 1.28 | 0.25 | 1.3E-03 | Orange |
| WP_041938849.1 | FRAAL1095 | 30S ribosomal protein S8 | 14627 | 9.74 | 8 | 154 | Frankia-9 | F201372.dat | 421 | 54 | 8 | 8 | 19 | 13 | 12 | 14 | 14 | 14 | 0.163% | 14 | 16 | 17 | 19 | 21 | 0.200% | 1.28 | 0.25 | 6.9E-03 | Orange |
| WP_011605622.1 | FRAAL4503 | bacteriocin | 28596 | 4.96 | 16 | 188 | Frankia-10 | F201373.dat | 753 | 44 | 14 | 14 | 21 | 16 | 16 | 16 | 17 | 17 | 0.102% | 21 | 23 | 17 | 24 | 21 | 0.125% | 1.28 | 0.24 | 2.4E-03 | Orange |
| WP_011606443.1 | FRAAL5358 | glutamine amidotransferase | 25573 | 4.67 | 7 | 56 | Frankia-8 | F201371.dat | 269 | 32 | 6 | 6 | 7 | 4 | 4 | 6 | 6 | 4 | 0.033% | 6 | 6 | 7 | 6 | 7 | 0.042% | 1.28 | 0.24 | 1.3E-02 | Orange |
| WP_041938922.1 | FRAAL1451 | nitrate ABC transporter ATPase | 30876 | 6.33 | 10 | 81 | Frankia-8 | F201371.dat | 279 | 33 | 8 | 8 | 10 | 6 | 7 | 7 | 7 | 8 | 0.040% | 8 | 11 | 10 | 7 | 10 | 0.050% | 1.28 | 0.24 | 1.3E-02 | Orange |
| WP_011604880.1 | FRAAL3741 | hypothetical protein | 18863 | 10.85 | 6 | 74 | Frankia-10 | F201373.dat | 363 | 50 | 6 | 6 | 9 | 5 | 9 | 7 | 6 | 5 | 0.061% | 7 | 8 | 9 | 9 | 9 | 0.075% | 1.27 | 0.24 | 2.3E-02 | Orange |
| WP_011606820.1 | FRAAL5746 | UDP-glucose 4-epimerase | 38402 | 5.44 | 13 | 83 | Frankia-6 | F201369.dat | 403 | 39 | 11 | 11 | 11 | 8 | 6 | 9 | 5 | 8 | 0.033% | 11 | 11 | 10 | 7 | 8 | 0.041% | 1.27 | 0.24 | 4.0E-02 | Orange |
| WP_041939027.1 | FRAAL1958 | short-chain dehydrogenase | 27146 | 5.53 | 13 | 119 | Frankia-6 | F201369.dat | 535 | 56 | 11 | 11 | 12 | 10 | 13 | 9 | 10 | 10 | 0.068% | 12 | 15 | 14 | 11 | 15 | 0.083% | 1.26 | 0.23 | 1.1E-02 | Orange |
| WP_011605735.1 | FRAAL4619 | transcriptional regulator | 22164 | 5.20 | 16 | 205 | Frankia-10 | F201373.dat | 1002 | 78 | 16 | 16 | 29 | 17 | 15 | 19 | 20 | 19 | 0.145% | 23 | 18 | 25 | 20 | 29 | 0.175% | 1.26 | 0.23 | 2.3E-02 | Orange |
| WP_011601627.1 | FRAAL0370 | DNA-binding protein | 33718 | 7.74 | 6 | 33 | Frankia-7 | F201370.dat | 224 | 21 | 5 | 5 | 5 | 2 | 3 | 4 | 2 | 3 | 0.015% | 3 | 5 | 4 | 3 | 4 | 0.019% | 1.26 | 0.23 | 4.8E-02 | Orange |
| WP_050997069.1 | FRAAL2452 | alpha/beta hydrolase | 33085 | 5.67 | 11 | 128 | Frankia-7 | F201370.dat | 503 | 41 | 11 | 11 | 16 | 11 | 12 | 11 | 12 | 10 | 0.060% | 15 | 16 | 11 | 17 | 13 | 0.073% | 1.26 | 0.23 | 1.1E-02 | Orange |
| WP_041939064.1 | FRAAL2152 | peptidylprolyl isomerase | 28517 | 9.30 | 6 | 87 | Frankia-8 | F201371.dat | 296 | 29 | 5 | 5 | 12 | 7 | 6 | 7 | 10 | 8 | 0.048% | 8 | 10 | 12 | 9 | 10 | 0.058% | 1.26 | 0.23 | 2.4E-02 | Orange |
| WP_011607125.1 | FRAAL6073 | 2-hydroxy-3-oxopropionate reductase | 31736 | 4.73 | 12 | 96 | Frankia-10 | F201373.dat | 743 | 57 | 10 | 10 | 13 | 7 | 8 | 9 | 7 | 11 | 0.047% | 9 | 10 | 10 | 12 | 13 | 0.057% | 1.26 | 0.23 | 2.6E-02 | Orange |
| WP_011606956.1 | FRAAL5895 | tellurium resistance protein terA | 23560 | 6.73 | 11 | 186 | Frankia-6 | F201369.dat | 758 | 69 | 11 | 11 | 19 | 16 | 13 | 19 | 17 | 17 | 0.124% | 19 | 19 | 25 | 20 | 21 | 0.149% | 1.25 | 0.23 | 9.0E-03 | Orange |
| WP_011607766.1 | FRAAL6729 | amino acid ABC transporter substrate-binding protein | 32189 | 4.99 | 16 | 161 | Frankia-7 | F201370.dat | 905 | 56 | 13 | 13 | 21 | 11 | 14 | 16 | 16 | 14 | 0.079% | 18 | 21 | 17 | 17 | 17 | 0.094% | 1.25 | 0.22 | 6.6E-03 | Orange |
| WP_050997454.1 | FRAAL6040 | antitermination regulator | 17696 | 11.40 | 4 | 35 | Frankia-8 | F201371.dat | 193 | 21 | 3 | 3 | 4 | 4 | 3 | 3 | 3 | 2 | 0.030% | 4 | 3 | 4 | 4 | 5 | 0.038% | 1.25 | 0.22 | 3.3E-02 | Orange |
| WP_011603586.1 | FRAAL2428 | nitroreductase | 19206 | 5.82 | 9 | 107 | Frankia-8 | F201371.dat | 366 | 63 | 9 | 9 | 15 | 9 | 10 | 6 | 11 | 11 | 0.087% | 11 | 10 | 15 | 11 | 13 | 0.105% | 1.25 | 0.22 | 3.9E-02 | Orange |
| WP_041939998.1 | FRAAL6853 | 30S ribosomal protein S6 | 11352 | 6.59 | 8 | 118 | Frankia-2 | F201365.dat | 399 | 78 | 8 | 8 | 13 | 7 | 13 | 10 | 10 | 12 | 0.163% | 11 | 13 | 14 | 13 | 15 | 0.196% | 1.25 | 0.22 | 2.6E-02 | Orange |
| WP_011607657.1 | FRAAL6620 | transcriptional regulator | 25623 | 5.13 | 11 | 129 | Frankia-7 | F201370.dat | 569 | 47 | 10 | 10 | 14 | 12 | 9 | 12 | 11 | 13 | 0.079% | 15 | 14 | 12 | 16 | 15 | 0.095% | 1.24 | 0.22 | 7.0E-03 | Orange |
| WP_011602281.1 | FRAAL1054 | hypothetical protein | 18049 | 7.88 | 13 | 102 | Frankia-10 | F201373.dat | 576 | 60 | 10 | 10 | 14 | 8 | 7 | 8 | 11 | 11 | 0.089% | 10 | 9 | 13 | 11 | 14 | 0.106% | 1.24 | 0.22 | 4.5E-02 | Orange |
| WP_009740511.1 | FRAAL1098 | 30S ribosomal protein S5 | 20717 | 10.44 | 12 | 196 | Frankia-5 | F201368.dat | 431 | 56 | 10 | 10 | 22 | 16 | 16 | 17 | 16 | 22 | 0.150% | 23 | 21 | 24 | 19 | 22 | 0.177% | 1.24 | 0.21 | 8.1E-03 | Orange |
| WP_011602481.1 | FRAAL1281 | twin-arginine translocation pathway signal protein | 103429 | 9.44 | 11 | 66 | Frankia-8 | F201371.dat | 364 | 15 | 9 | 9 | 9 | 6 | 7 | 7 | 4 | 5 | 0.010% | 6 | 8 | 9 | 7 | 7 | 0.012% | 1.24 | 0.21 | 3.6E-02 | Orange |
| WP_011601851.1 | FRAAL0601 | metal-binding protein | 56371 | 9.76 | 31 | 390 | Frankia-7 | F201370.dat | 1423 | 52 | 27 | 27 | 44 | 38 | 35 | 30 | 34 | 37 | 0.110% | 40 | 44 | 47 | 43 | 42 | 0.129% | 1.23 | 0.21 | 8.4E-04 | Orange |
| WP_041939926.1 | FRAAL6583 | two-component system response regulator | 27244 | 6.80 | 23 | 363 | Frankia-9 | F201372.dat | 1099 | 84 | 21 | 21 | 45 | 31 | 31 | 33 | 32 | 35 | 0.212% | 38 | 38 | 38 | 45 | 42 | 0.248% | 1.23 | 0.21 | 6.5E-04 | Orange |
| WP_041939546.1 | FRAAL4633 | 3-oxoacyl-ACP reductase | 27087 | 5.83 | 14 | 260 | Frankia-9 | F201372.dat | 849 | 58 | 13 | 13 | 28 | 22 | 21 | 23 | 26 | 24 | 0.153% | 25 | 32 | 29 | 28 | 30 | 0.179% | 1.23 | 0.21 | 2.3E-03 | Orange |
| WP_011607126.1 | FRAAL6074 | stress protein | 50974 | 8.39 | 16 | 146 | Frankia-8 | F201371.dat | 674 | 28 | 13 | 13 | 19 | 13 | 13 | 14 | 12 | 13 | 0.046% | 12 | 18 | 19 | 15 | 17 | 0.054% | 1.23 | 0.21 | 1.9E-02 | Orange |
| WP_041938921.1 | FRAAL1450 | ABC transporter substrate-binding protein | 38213 | 4.97 | 37 | 478 | Frankia-7 | F201370.dat | 1614 | 67 | 26 | 26 | 58 | 42 | 40 | 44 | 38 | 50 | 0.200% | 43 | 58 | 60 | 54 | 49 | 0.233% | 1.23 | 0.21 | 1.4E-02 | Orange |
| WP_011607638.1 | FRAAL6601 | nitroreductase | 23849 | 6.08 | 11 | 108 | Frankia-9 | F201372.dat | 475 | 56 | 9 | 9 | 13 | 7 | 12 | 8 | 11 | 10 | 0.072% | 12 | 11 | 12 | 13 | 12 | 0.085% | 1.23 | 0.20 | 2.0E-02 | Orange |
| WP_011606861.1 | FRAAL5789 | 50S ribosomal protein L19 | 13500 | 10.89 | 11 | 208 | Frankia-10 | F201373.dat | 461 | 67 | 11 | 11 | 30 | 18 | 19 | 18 | 18 | 20 | 0.246% | 21 | 20 | 20 | 24 | 30 | 0.287% | 1.22 | 0.20 | 2.6E-02 | Orange |
| WP_011603090.1 | FRAAL1915 | nucleoside diphosphate kinase | 14496 | 5.39 | 12 | 228 | Frankia-10 | F201373.dat | 526 | 62 | 11 | 11 | 26 | 20 | 21 | 17 | 22 | 22 | 0.251% | 25 | 25 | 26 | 24 | 26 | 0.293% | 1.22 | 0.20 | 6.8E-04 | Orange |
| WP_011604937.1 | FRAAL3799 | enoyl-CoA hydratase | 26192 | 5.15 | 19 | 268 | Frankia-10 | F201373.dat | 942 | 80 | 16 | 16 | 31 | 27 | 23 | 20 | 23 | 27 | 0.163% | 23 | 30 | 32 | 32 | 31 | 0.190% | 1.22 | 0.20 | 1.6E-02 | Orange |
| WP_011606864.1 | FRAAL5792 | 30S ribosomal protein S16 | 15866 | 9.90 | 16 | 328 | Frankia-10 | F201373.dat | 707 | 82 | 15 | 15 | 44 | 27 | 27 | 28 | 32 | 33 | 0.331% | 27 | 31 | 42 | 37 | 44 | 0.384% | 1.22 | 0.20 | 4.3E-02 | Orange |
| WP_009740503.1 | FRAAL1108 | 30S ribosomal protein S11 | 14330 | 11.42 | 9 | 170 | Frankia-10 | F201373.dat | 571 | 77 | 8 | 8 | 23 | 14 | 13 | 19 | 15 | 15 | 0.189% | 18 | 16 | 20 | 17 | 23 | 0.221% | 1.22 | 0.20 | 2.8E-02 | Orange |
| WP_011601666.1 | FRAAL0410 | heavy metal transporter | 65533 | 5.52 | 18 | 121 | Frankia-6 | F201369.dat | 525 | 27 | 12 | 12 | 14 | 13 | 10 | 11 | 11 | 9 | 0.029% | 14 | 13 | 16 | 12 | 12 | 0.034% | 1.22 | 0.20 | 1.6E-02 | Orange |
| WP_009740526.1 | FRAAL1083 | 50S ribosomal protein L23 | 10939 | 10.47 | 3 | 61 | Frankia-9 | F201372.dat | 205 | 42 | 3 | 3 | 7 | 4 | 6 | 6 | 6 | 5 | 0.088% | 6 | 6 | 7 | 7 | 8 | 0.105% | 1.22 | 0.20 | 1.7E-02 | Orange |
| WP_041938956.1 | FRAAL1640 | acyl-CoA synthetase | 55313 | 5.44 | 8 | 61 | Frankia-7 | F201370.dat | 428 | 18 | 7 | 7 | 7 | 5 | 6 | 4 | 7 | 5 | 0.017% | 6 | 7 | 7 | 7 | 7 | 0.021% | 1.22 | 0.20 | 2.5E-02 | Orange |
| WP_007514815.1 | FRAAL1080 | 30S ribosomal protein S10 | 11639 | 9.37 | 15 | 258 | Frankia-10 | F201373.dat | 609 | 84 | 13 | 13 | 33 | 19 | 26 | 24 | 23 | 24 | 0.356% | 25 | 27 | 27 | 30 | 33 | 0.411% | 1.21 | 0.19 | 1.1E-02 | Orange |
| WP_050997302.1 | FRAAL0148 | hypothetical protein | 41004 | 8.86 | 13 | 156 | Frankia-6 | F201369.dat | 789 | 45 | 12 | 12 | 17 | 16 | 12 | 14 | 14 | 14 | 0.061% | 17 | 18 | 16 | 19 | 16 | 0.071% | 1.21 | 0.19 | 2.9E-03 | Orange |
| WP_011606884.1 | FRAAL5814 | D-alanine--D-alanine ligase | 38500 | 5.22 | 11 | 74 | Frankia-6 | F201369.dat | 338 | 25 | 8 | 8 | 8 | 5 | 7 | 6 | 7 | 8 | 0.031% | 8 | 8 | 7 | 9 | 9 | 0.036% | 1.21 | 0.19 | 1.8E-02 | Orange |
| WP_011604557.1 | FRAAL3411 | carnitine dehydratase | 51654 | 5.11 | 14 | 74 | Frankia-8 | F201371.dat | 466 | 22 | 8 | 8 | 10 | 7 | 6 | 7 | 6 | 7 | 0.023% | 8 | 8 | 10 | 9 | 6 | 0.027% | 1.21 | 0.19 | 3.1E-02 | Orange |
| WP_041940884.1 | FRAAL5216 | translation initiation factor IF-3 | 17107 | 9.90 | 15 | 191 | Frankia-8 | F201371.dat | 572 | 69 | 13 | 13 | 25 | 16 | 15 | 16 | 19 | 20 | 0.179% | 18 | 17 | 25 | 21 | 24 | 0.207% | 1.21 | 0.19 | 3.7E-02 | Orange |
| WP_011602808.1 | FRAAL1619 | PPOX class F420-dependent enzyme | 14790 | 6.27 | 9 | 149 | Frankia-10 | F201373.dat | 444 | 60 | 8 | 8 | 19 | 13 | 13 | 15 | 13 | 13 | 0.162% | 12 | 16 | 15 | 20 | 19 | 0.187% | 1.21 | 0.19 | 3.9E-02 | Orange |
| WP_011603359.1 | FRAAL2194 | UDP-N-acetylmuramoylalanine--D-glutamate ligase | 46548 | 6.85 | 7 | 54 | Frankia-8 | F201371.dat | 369 | 18 | 7 | 7 | 7 | 5 | 4 | 5 | 5 | 5 | 0.018% | 6 | 6 | 7 | 7 | 4 | 0.022% | 1.21 | 0.19 | 4.9E-02 | Orange |
| WP_006541558.1 | FRAAL1105 | translation initiation factor IF-1 | 8537 | 9.77 | 7 | 129 | Frankia-10 | F201373.dat | 345 | 78 | 7 | 7 | 18 | 11 | 13 | 13 | 11 | 10 | 0.242% | 12 | 13 | 13 | 15 | 18 | 0.280% | 1.21 | 0.19 | 3.3E-02 | Orange |
| WP_011603376.1 | FRAAL2211 | hypothetical protein | 24094 | 5.14 | 12 | 226 | Frankia-9 | F201372.dat | 491 | 51 | 12 | 12 | 24 | 17 | 23 | 19 | 22 | 21 | 0.151% | 24 | 24 | 26 | 24 | 26 | 0.173% | 1.21 | 0.19 | 2.9E-03 | Orange |
| WP_041939516.1 | FRAAL4482 | hypothetical protein | 20375 | 4.73 | 7 | 78 | Frankia-9 | F201372.dat | 501 | 48 | 6 | 6 | 10 | 8 | 6 | 7 | 7 | 7 | 0.061% | 9 | 7 | 9 | 10 | 8 | 0.071% | 1.20 | 0.18 | 1.5E-02 | Orange |
| WP_011605343.1 | FRAAL4216 | bifunctional protein FolD | 29300 | 5.28 | 15 | 148 | Frankia-7 | F201370.dat | 478 | 44 | 12 | 12 | 17 | 11 | 10 | 14 | 15 | 17 | 0.082% | 14 | 17 | 17 | 17 | 16 | 0.093% | 1.19 | 0.18 | 4.2E-02 | Orange |
| WP_035923438.1 | FRAAL6556 | Crp/Fnr family transcriptional regulator | 25140 | 6.35 | 16 | 330 | Frankia-7 | F201370.dat | 838 | 69 | 15 | 15 | 43 | 31 | 28 | 34 | 30 | 27 | 0.213% | 33 | 43 | 35 | 36 | 33 | 0.241% | 1.19 | 0.18 | 1.3E-02 | Orange |
| WP_011606318.1 | FRAAL5223 | ribulose-phosphate 3-epimerase | 26162 | 4.72 | 6 | 58 | Frankia-10 | F201373.dat | 275 | 29 | 6 | 6 | 7 | 6 | 5 | 5 | 4 | 6 | 0.035% | 5 | 7 | 6 | 7 | 7 | 0.041% | 1.19 | 0.18 | 3.0E-02 | Orange |
| WP_011605381.1 | FRAAL4255 | PPOX class F420-dependent enzyme | 14537 | 5.63 | 10 | 93 | Frankia-7 | F201370.dat | 429 | 77 | 7 | 7 | 11 | 7 | 9 | 7 | 9 | 10 | 0.103% | 11 | 11 | 9 | 10 | 10 | 0.118% | 1.19 | 0.18 | 1.7E-02 | Orange |
| WP_011605778.1 | FRAAL4662 | hypothetical protein | 27916 | 5.54 | 15 | 237 | Frankia-10 | F201373.dat | 883 | 60 | 13 | 13 | 29 | 21 | 23 | 23 | 20 | 21 | 0.138% | 24 | 25 | 27 | 24 | 29 | 0.156% | 1.19 | 0.17 | 3.1E-03 | Orange |
| WP_050997134.1 | FRAAL3470 | phosphoketolase | 89482 | 5.88 | 36 | 274 | Frankia-8 | F201371.dat | 1239 | 40 | 26 | 26 | 30 | 25 | 25 | 27 | 20 | 28 | 0.050% | 29 | 26 | 30 | 33 | 31 | 0.056% | 1.18 | 0.17 | 1.4E-02 | Orange |
| WP_011604921.1 | FRAAL3783 | transcriptional regulator | 22955 | 5.99 | 12 | 88 | Frankia-8 | F201371.dat | 475 | 43 | 8 | 8 | 11 | 7 | 8 | 10 | 8 | 7 | 0.062% | 10 | 9 | 11 | 9 | 9 | 0.070% | 1.18 | 0.16 | 2.3E-02 | Orange |
| WP_041939092.1 | FRAAL2263 | glycoside hydrolase | 37923 | 9.67 | 9 | 212 | Frankia-9 | F201372.dat | 624 | 39 | 9 | 9 | 26 | 20 | 20 | 20 | 17 | 20 | 0.091% | 26 | 25 | 17 | 26 | 21 | 0.102% | 1.18 | 0.16 | 4.4E-02 | Orange |
| WP_011602672.1 | FRAAL1482 | amino acid ABC transporter substrate-binding protein | 47539 | 6.88 | 27 | 626 | Frankia-8 | F201371.dat | 1759 | 81 | 26 | 26 | 63 | 62 | 53 | 57 | 59 | 57 | 0.216% | 67 | 76 | 63 | 63 | 69 | 0.239% | 1.17 | 0.16 | 3.7E-03 | Orange |
| WP_011602307.1 | FRAAL1093 | 50S ribosomal protein L5 | 21752 | 9.97 | 23 | 400 | Frankia-7 | F201370.dat | 958 | 80 | 19 | 19 | 44 | 35 | 35 | 38 | 36 | 40 | 0.302% | 34 | 44 | 46 | 46 | 46 | 0.334% | 1.17 | 0.16 | 1.8E-02 | Orange |
| WP_041938715.1 | FRAAL0541 | short-chain dehydrogenase | 26289 | 5.29 | 9 | 81 | Frankia-8 | F201371.dat | 369 | 38 | 8 | 8 | 10 | 8 | 8 | 7 | 6 | 8 | 0.050% | 9 | 8 | 10 | 9 | 8 | 0.056% | 1.17 | 0.15 | 1.7E-02 | Orange |
| WP_041938813.1 | FRAAL0936 | transcriptional regulator | 26725 | 6.23 | 9 | 96 | Frankia-7 | F201370.dat | 498 | 48 | 8 | 8 | 12 | 9 | 9 | 8 | 9 | 9 | 0.059% | 10 | 12 | 9 | 11 | 10 | 0.066% | 1.16 | 0.15 | 1.5E-02 | Orange |
| WP_011605479.1 | FRAAL4357 | hypothetical protein | 42090 | 6.00 | 20 | 184 | Frankia-10 | F201373.dat | 897 | 49 | 15 | 15 | 21 | 18 | 17 | 18 | 14 | 18 | 0.072% | 19 | 23 | 18 | 18 | 21 | 0.079% | 1.16 | 0.14 | 2.7E-02 | Orange |
| WP_009740540.1 | FRAAL1065 | 50S ribosomal protein L11 | 15174 | 9.68 | 11 | 147 | Frankia-10 | F201373.dat | 578 | 75 | 10 | 10 | 18 | 11 | 14 | 15 | 14 | 14 | 0.160% | 15 | 15 | 16 | 15 | 18 | 0.175% | 1.15 | 0.14 | 2.0E-02 | Orange |
| WP_011607582.1 | FRAAL6542 | uridylate kinase | 28168 | 6.13 | 15 | 119 | Frankia-7 | F201370.dat | 520 | 55 | 12 | 12 | 13 | 10 | 10 | 12 | 11 | 12 | 0.070% | 11 | 13 | 15 | 12 | 13 | 0.077% | 1.15 | 0.14 | 2.7E-02 | Orange |
| WP_041940172.1 | FRAAL1100 | 50S ribosomal protein L15 | 15072 | 10.84 | 7 | 76 | Frankia-9 | F201372.dat | 351 | 59 | 7 | 7 | 10 | 7 | 8 | 6 | 7 | 7 | 0.083% | 8 | 8 | 8 | 10 | 7 | 0.092% | 1.15 | 0.14 | 3.8E-02 | Orange |
| WP_011604020.1 | FRAAL2874 | proteasome subunit alpha | 26287 | 5.99 | 16 | 351 | Frankia-2 | F201365.dat | 941 | 62 | 15 | 15 | 29 | 29 | 29 | 39 | 33 | 33 | 0.221% | 35 | 39 | 37 | 40 | 37 | 0.241% | 1.15 | 0.14 | 2.0E-02 | Orange |
| WP_041939907.1 | FRAAL6506 | Crp/Fnr family transcriptional regulator | 51018 | 5.23 | 28 | 634 | Frankia-9 | F201372.dat | 1730 | 72 | 27 | 27 | 72 | 56 | 62 | 58 | 57 | 62 | 0.206% | 69 | 66 | 64 | 72 | 68 | 0.224% | 1.15 | 0.14 | 7.3E-04 | Orange |
| WP_011606788.1 | FRAAL5714 | ABC transporter substrate-binding protein | 34746 | 9.37 | 36 | 723 | Frankia-10 | F201373.dat | 2097 | 65 | 35 | 35 | 77 | 66 | 67 | 72 | 69 | 63 | 0.346% | 72 | 87 | 72 | 78 | 77 | 0.374% | 1.14 | 0.13 | 7.0E-03 | Orange |
| WP_041939041.1 | FRAAL2025 | DNA primase | 68930 | 6.83 | 14 | 82 | Frankia-8 | F201371.dat | 387 | 16 | 9 | 9 | 9 | 8 | 7 | 9 | 7 | 7 | 0.020% | 9 | 9 | 9 | 10 | 7 | 0.021% | 1.14 | 0.13 | 4.7E-02 | Orange |
| WP_011602189.1 | FRAAL0960 | ABC transporter substrate-binding protein | 46341 | 8.87 | 17 | 236 | Frankia-10 | F201373.dat | 969 | 50 | 16 | 16 | 27 | 22 | 20 | 22 | 23 | 23 | 0.085% | 26 | 28 | 20 | 25 | 27 | 0.092% | 1.14 | 0.13 | 3.2E-02 | Orange |
| WP_041938754.1 | FRAAL0725 | ABC transporter substrate-binding protein | 39941 | 5.66 | 35 | 1032 | Frankia-10 | F201373.dat | 2399 | 90 | 31 | 31 | 112 | 96 | 88 | 97 | 97 | 105 | 0.432% | 106 | 115 | 101 | 115 | 112 | 0.463% | 1.14 | 0.13 | 4.5E-03 | Orange |
| WP_011605894.1 | FRAAL4779 | 3-hydroxy-2-methylbutyryl-CoA dehydrogenase | 25846 | 5.13 | 12 | 184 | Frankia-10 | F201373.dat | 997 | 82 | 12 | 12 | 23 | 16 | 16 | 18 | 16 | 20 | 0.119% | 17 | 19 | 20 | 19 | 23 | 0.128% | 1.13 | 0.12 | 4.7E-02 | Orange |
| WP_041939757.1 | FRAAL5770 | sulfonate ABC transporter substrate-binding protein | 37196 | 9.13 | 20 | 233 | Frankia-7 | F201370.dat | 892 | 58 | 15 | 15 | 28 | 21 | 23 | 21 | 21 | 23 | 0.105% | 23 | 28 | 25 | 24 | 24 | 0.112% | 1.13 | 0.12 | 8.1E-03 | Orange |
| WP_011606156.1 | FRAAL5045 | hypothetical protein | 16959 | 4.78 | 13 | 241 | Frankia-2 | F201365.dat | 772 | 76 | 12 | 12 | 23 | 21 | 23 | 23 | 24 | 22 | 0.238% | 21 | 27 | 28 | 28 | 24 | 0.254% | 1.13 | 0.12 | 3.7E-02 | Orange |
| WP_011602965.1 | FRAAL1783 | glutathione peroxidase | 19545 | 4.49 | 12 | 249 | Frankia-9 | F201372.dat | 799 | 73 | 12 | 12 | 29 | 24 | 26 | 22 | 24 | 21 | 0.214% | 28 | 24 | 24 | 29 | 27 | 0.227% | 1.12 | 0.12 | 2.8E-02 | Orange |
| WP_041939054.1 | FRAAL2103 | chlorite dismutase | 26420 | 5.54 | 14 | 111 | Frankia-3 | F201366.dat | 418 | 44 | 11 | 11 | 11 | 9 | 11 | 11 | 10 | 11 | 0.070% | 11 | 13 | 12 | 12 | 11 | 0.075% | 1.12 | 0.12 | 1.7E-02 | Orange |
| WP_011602298.1 | FRAAL1077 | 30S ribosomal protein S7 | 17188 | 10.48 | 17 | 369 | Frankia-10 | F201373.dat | 962 | 74 | 16 | 16 | 42 | 33 | 39 | 34 | 35 | 33 | 0.361% | 35 | 39 | 37 | 42 | 42 | 0.382% | 1.12 | 0.11 | 2.3E-02 | Orange |
| WP_041939238.1 | FRAAL2873 | proteasome subunit beta | 27596 | 5.05 | 21 | 390 | Frankia-5 | F201368.dat | 1282 | 70 | 18 | 18 | 38 | 33 | 36 | 39 | 38 | 38 | 0.238% | 40 | 40 | 39 | 42 | 45 | 0.251% | 1.12 | 0.11 | 9.7E-03 | Orange |
| WP_041939357.1 | FRAAL3551 | 3-ketoacyl-ACP reductase | 28978 | 4.71 | 17 | 182 | Frankia-5 | F201368.dat | 708 | 49 | 13 | 13 | 17 | 18 | 16 | 18 | 18 | 17 | 0.107% | 18 | 20 | 19 | 19 | 19 | 0.110% | 1.09 | 0.08 | 7.5E-03 | Orange |
| WP_011601560.1 | FRAAL0302 | peroxidase | 24010 | 4.99 | 21 | 476 | Frankia-5 | F201368.dat | 971 | 70 | 18 | 18 | 48 | 42 | 45 | 44 | 49 | 48 | 0.339% | 45 | 53 | 52 | 49 | 49 | 0.348% | 1.09 | 0.08 | 3.4E-02 | Orange |
| WP_011606744.1 | FRAAL5668 | ACP S-malonyltransferase | 34266 | 5.32 | 14 | 213 | Frankia-10 | F201373.dat | 908 | 57 | 13 | 13 | 22 | 21 | 21 | 19 | 21 | 21 | 0.107% | 21 | 22 | 24 | 21 | 22 | 0.108% | 1.06 | 0.06 | 3.6E-02 | Orange |
| WP_011607676.1 | FRAAL6639 | molecular chaperone DnaK | 65021 | 4.76 | 61 | 1298 | Frankia-8 | F201371.dat | 3051 | 73 | 50 | 50 | 134 | 141 | 125 | 132 | 135 | 133 | 0.366% | 121 | 121 | 134 | 126 | 130 | 0.327% | -1.05 | -0.05 | 4.9E-02 | Orange |
| WP_050996951.1 | FRAAL0094 | peptidase S1 | 56356 | 5.15 | 14 | 272 | Frankia-7 | F201370.dat | 970 | 43 | 14 | 14 | 25 | 29 | 28 | 28 | 29 | 27 | 0.089% | 26 | 25 | 25 | 28 | 27 | 0.078% | -1.07 | -0.07 | 1.0E-02 | Orange |
| WP_009742347.1 | FRAAL5772 | 4-hydroxy-3-methylbut-2-en-1-yl diphosphate synthase (flavodoxin) | 40108 | 5.51 | 28 | 482 | Frankia-4 | F201367.dat | 1477 | 72 | 24 | 24 | 56 | 49 | 52 | 46 | 56 | 48 | 0.223% | 45 | 48 | 46 | 48 | 44 | 0.194% | -1.08 | -0.08 | 3.5E-02 | Orange |
| WP_041939851.1 | FRAAL6233 | enolase | 44718 | 4.66 | 35 | 791 | Frankia-5 | F201368.dat | 2174 | 65 | 33 | 33 | 86 | 79 | 84 | 82 | 81 | 86 | 0.329% | 73 | 81 | 70 | 74 | 81 | 0.285% | -1.09 | -0.08 | 1.6E-02 | Orange |
| WP_011606938.1 | FRAAL5877 | electron transfer flavoprotein subunit alpha | 32579 | 5.13 | 16 | 432 | Frankia-5 | F201368.dat | 1024 | 67 | 14 | 14 | 50 | 42 | 42 | 44 | 48 | 50 | 0.248% | 37 | 42 | 44 | 42 | 41 | 0.213% | -1.09 | -0.09 | 4.0E-02 | Orange |
| WP_011606793.1 | FRAAL5719 | protein RecA | 36543 | 5.23 | 20 | 164 | Frankia-10 | F201373.dat | 706 | 52 | 13 | 13 | 17 | 18 | 16 | 17 | 17 | 18 | 0.084% | 17 | 15 | 13 | 16 | 17 | 0.072% | -1.10 | -0.09 | 4.6E-02 | Orange |
| WP_011605704.1 | FRAAL4588 | glyceraldehyde-3-phosphate dehydrogenase | 35562 | 5.67 | 29 | 877 | Frankia-7 | F201370.dat | 1884 | 85 | 27 | 27 | 91 | 88 | 87 | 96 | 97 | 93 | 0.463% | 75 | 91 | 92 | 78 | 80 | 0.394% | -1.11 | -0.10 | 2.8E-02 | Orange |
| WP_011607738.1 | FRAAL6701 | molecular chaperone GroEL | 56802 | 4.88 | 83 | 2701 | Frankia-3 | F201366.dat | 5475 | 97 | 76 | 17 | 283 | 287 | 270 | 283 | 282 | 298 | 0.892% | 238 | 283 | 275 | 237 | 248 | 0.759% | -1.11 | -0.10 | 1.5E-02 | Orange |
| WP_041941002.1 | FRAAL5842 | branched-chain amino acid aminotransferase | 33755 | 5.00 | 20 | 376 | Frankia-8 | F201371.dat | 1085 | 69 | 19 | 19 | 39 | 39 | 37 | 40 | 41 | 41 | 0.209% | 31 | 35 | 39 | 34 | 39 | 0.178% | -1.11 | -0.10 | 2.4E-02 | Orange |
| WP_011607705.1 | FRAAL6667 | phosphoribosylaminoimidazole-succinocarboxamide synthase | 33193 | 4.72 | 28 | 395 | Frankia-4 | F201367.dat | 1280 | 76 | 23 | 23 | 48 | 41 | 40 | 39 | 48 | 40 | 0.224% | 36 | 36 | 43 | 34 | 38 | 0.190% | -1.11 | -0.10 | 4.9E-02 | Orange |
| WP_041938882.1 | FRAAL1253 | glycosyl transferase family 1 | 40424 | 5.96 | 16 | 237 | Frankia-5 | F201368.dat | 796 | 56 | 15 | 15 | 29 | 24 | 25 | 23 | 24 | 29 | 0.110% | 22 | 24 | 22 | 21 | 23 | 0.093% | -1.11 | -0.11 | 2.8E-02 | Orange |
| WP_011606811.1 | FRAAL5737 | RNase J family beta-CASP ribonuclease | 60639 | 6.03 | 32 | 347 | Frankia-7 | F201370.dat | 1498 | 55 | 26 | 26 | 36 | 38 | 37 | 36 | 34 | 38 | 0.108% | 35 | 36 | 31 | 33 | 29 | 0.091% | -1.11 | -0.11 | 1.7E-02 | Orange |
| WP_011606867.1 | FRAAL5795 | signal recognition particle-docking protein FtsY | 43433 | 5.07 | 22 | 309 | Frankia-4 | F201367.dat | 1081 | 45 | 19 | 19 | 34 | 31 | 34 | 32 | 34 | 32 | 0.134% | 28 | 27 | 28 | 31 | 32 | 0.113% | -1.11 | -0.11 | 8.8E-03 | Orange |
| WP_011607093.1 | FRAAL6041 | peptidase M16 | 44955 | 5.01 | 20 | 269 | Frankia-8 | F201371.dat | 972 | 51 | 18 | 18 | 29 | 32 | 26 | 28 | 27 | 29 | 0.113% | 22 | 26 | 29 | 25 | 25 | 0.095% | -1.11 | -0.11 | 4.2E-02 | Orange |
| WP_011606915.1 | FRAAL5848 | 3-phosphoglycerate dehydrogenase | 54569 | 4.85 | 28 | 544 | Frankia-4 | F201367.dat | 1723 | 66 | 25 | 25 | 58 | 61 | 52 | 61 | 58 | 55 | 0.188% | 49 | 57 | 51 | 51 | 49 | 0.159% | -1.11 | -0.11 | 1.5E-02 | Orange |
| WP_041938845.1 | FRAAL1051 | 2-oxoglutarate ferredoxin oxidoreductase subunit alpha | 67096 | 5.88 | 34 | 620 | Frankia-1 | F201364.dat | 2043 | 58 | 30 | 29 | 70 | 70 | 64 | 72 | 58 | 64 | 0.174% | 57 | 61 | 62 | 56 | 56 | 0.147% | -1.12 | -0.11 | 1.6E-02 | Orange |
| WP_011607716.1 | FRAAL6680 | ATP-dependent Clp protease ATP-binding protein | 92095 | 5.70 | 75 | 1223 | Frankia-1 | F201364.dat | 3528 | 70 | 64 | 61 | 137 | 137 | 132 | 135 | 127 | 118 | 0.251% | 118 | 102 | 126 | 114 | 114 | 0.210% | -1.13 | -0.12 | 9.8E-03 | Orange |
| WP_011606916.1 | FRAAL5849 | ketol-acid reductoisomerase | 35460 | 5.09 | 29 | 843 | Frankia-5 | F201368.dat | 2059 | 80 | 29 | 29 | 96 | 85 | 89 | 84 | 94 | 96 | 0.451% | 71 | 73 | 86 | 79 | 86 | 0.375% | -1.13 | -0.12 | 1.4E-02 | Orange |
| WP_041938992.1 | FRAAL1781 | 30S ribosomal protein S1 | 54771 | 4.72 | 44 | 885 | Frankia-5 | F201368.dat | 2185 | 69 | 42 | 42 | 96 | 98 | 88 | 89 | 100 | 96 | 0.307% | 88 | 79 | 85 | 79 | 83 | 0.255% | -1.14 | -0.13 | 2.5E-03 | Orange |
| WP_011606296.1 | FRAAL5202 | argininosuccinate synthase | 42999 | 4.80 | 32 | 376 | Frankia-1 | F201364.dat | 1527 | 71 | 26 | 26 | 40 | 40 | 41 | 37 | 40 | 43 | 0.167% | 32 | 32 | 38 | 32 | 41 | 0.137% | -1.14 | -0.13 | 2.0E-02 | Orange |
| WP_011607698.1 | FRAAL6660 | adenylosuccinate synthetase | 46136 | 6.04 | 21 | 192 | Frankia-8 | F201371.dat | 824 | 41 | 14 | 14 | 20 | 19 | 20 | 22 | 21 | 21 | 0.080% | 16 | 20 | 20 | 18 | 15 | 0.065% | -1.15 | -0.14 | 2.0E-02 | Orange |
| WP_041939978.1 | FRAAL6781 | S-adenosyl-L-homocysteine hydrolase | 51596 | 5.06 | 34 | 582 | Frankia-5 | F201368.dat | 1593 | 53 | 30 | 30 | 65 | 65 | 61 | 58 | 63 | 65 | 0.216% | 54 | 47 | 58 | 53 | 58 | 0.176% | -1.15 | -0.14 | 4.2E-03 | Orange |
| WP_041939531.1 | FRAAL4537 | pyruvate kinase | 49633 | 5.54 | 33 | 809 | Frankia-3 | F201366.dat | 2026 | 85 | 32 | 32 | 87 | 88 | 91 | 87 | 87 | 81 | 0.312% | 75 | 76 | 79 | 71 | 74 | 0.254% | -1.16 | -0.14 | 2.4E-04 | Orange |
| WP_011602623.1 | FRAAL1428 | squalene synthase | 34852 | 6.02 | 24 | 307 | Frankia-7 | F201370.dat | 1246 | 73 | 20 | 20 | 36 | 32 | 32 | 34 | 34 | 33 | 0.169% | 27 | 36 | 25 | 28 | 26 | 0.137% | -1.16 | -0.15 | 2.6E-02 | Orange |
| WP_011607254.1 | FRAAL6204 | phosphoenolpyruvate carboxykinase [GTP] | 66539 | 5.07 | 54 | 1025 | Frankia-1 | F201364.dat | 2697 | 69 | 47 | 47 | 126 | 126 | 100 | 104 | 110 | 111 | 0.296% | 84 | 97 | 116 | 84 | 93 | 0.240% | -1.16 | -0.15 | 3.5E-02 | Orange |
| WP_041939534.1 | FRAAL4560 | Fe-S cluster assembly ATPase SufC | 28269 | 5.61 | 23 | 487 | Frankia-5 | F201368.dat | 1313 | 83 | 21 | 21 | 53 | 52 | 50 | 51 | 56 | 53 | 0.331% | 39 | 49 | 45 | 45 | 47 | 0.268% | -1.16 | -0.15 | 2.7E-03 | Orange |
| WP_011605703.1 | FRAAL4587 | phosphoglycerate kinase | 40767 | 5.16 | 27 | 483 | Frankia-4 | F201367.dat | 1403 | 69 | 25 | 25 | 54 | 49 | 52 | 49 | 54 | 56 | 0.228% | 41 | 39 | 48 | 42 | 53 | 0.184% | -1.16 | -0.15 | 1.8E-02 | Orange |
| WP_041940781.1 | FRAAL4586 | triosephosphate isomerase | 27119 | 5.23 | 13 | 241 | Frankia-2 | F201365.dat | 824 | 60 | 11 | 11 | 27 | 24 | 27 | 23 | 28 | 28 | 0.171% | 19 | 21 | 23 | 23 | 25 | 0.138% | -1.16 | -0.15 | 1.6E-02 | Orange |
| WP_011606827.1 | FRAAL5753 | polyribonucleotide nucleotidyltransferase | 77175 | 5.09 | 62 | 1243 | Frankia-10 | F201373.dat | 2795 | 60 | 51 | 51 | 121 | 140 | 137 | 126 | 129 | 137 | 0.309% | 116 | 112 | 114 | 111 | 121 | 0.250% | -1.16 | -0.15 | 1.8E-04 | Orange |
| WP_011605117.1 | FRAAL3986 | heat-shock protein Hsp90 | 73015 | 4.83 | 43 | 553 | Frankia-1 | F201364.dat | 1750 | 54 | 36 | 36 | 66 | 66 | 60 | 51 | 57 | 64 | 0.146% | 52 | 45 | 59 | 46 | 53 | 0.118% | -1.17 | -0.15 | 2.4E-02 | Orange |
| WP_011602301.1 | FRAAL1082 | 50S ribosomal protein L4 | 24666 | 10.38 | 10 | 209 | Frankia-5 | F201368.dat | 573 | 36 | 10 | 10 | 26 | 21 | 22 | 21 | 23 | 26 | 0.163% | 17 | 19 | 20 | 19 | 21 | 0.131% | -1.17 | -0.16 | 8.8E-03 | Orange |
| WP_009739818.1 | FRAAL1916 | rod shape-determining protein Mbl | 36693 | 6.21 | 18 | 153 | Frankia-3 | F201366.dat | 627 | 51 | 13 | 13 | 16 | 16 | 17 | 16 | 16 | 18 | 0.081% | 14 | 16 | 13 | 13 | 14 | 0.064% | -1.17 | -0.16 | 2.5E-03 | Orange |
| WP_041939664.1 | FRAAL5252 | alanine--tRNA ligase | 94597 | 5.17 | 43 | 440 | Frankia-3 | F201366.dat | 1650 | 42 | 30 | 30 | 51 | 53 | 46 | 51 | 44 | 44 | 0.090% | 39 | 38 | 51 | 34 | 40 | 0.072% | -1.17 | -0.16 | 3.3E-02 | Orange |
| WP_041939653.1 | FRAAL5168 | threonyl-tRNA synthetase | 73493 | 5.46 | 26 | 227 | Frankia-5 | F201368.dat | 903 | 40 | 19 | 19 | 24 | 27 | 23 | 24 | 25 | 24 | 0.060% | 21 | 17 | 26 | 19 | 21 | 0.048% | -1.17 | -0.16 | 2.5E-02 | Orange |
| WP_011602353.1 | FRAAL1150 | GMP synthase | 60125 | 5.47 | 30 | 286 | Frankia-8 | F201371.dat | 1304 | 52 | 25 | 25 | 30 | 36 | 28 | 32 | 32 | 27 | 0.092% | 27 | 23 | 30 | 23 | 28 | 0.073% | -1.18 | -0.16 | 2.7E-02 | Orange |
| WP_011606993.1 | FRAAL5933 | ATP synthase subunit alpha | 59505 | 5.12 | 54 | 1338 | Frankia-5 | F201368.dat | 2968 | 71 | 51 | 51 | 143 | 145 | 146 | 149 | 141 | 143 | 0.434% | 120 | 128 | 120 | 119 | 127 | 0.347% | -1.18 | -0.16 | 7.2E-06 | Orange |
| WP_041940872.1 | FRAAL5078 | oxidoreductase | 57319 | 5.62 | 16 | 147 | Frankia-1 | F201364.dat | 658 | 31 | 14 | 14 | 19 | 19 | 14 | 15 | 18 | 14 | 0.050% | 12 | 12 | 16 | 12 | 15 | 0.039% | -1.18 | -0.17 | 4.7E-02 | Orange |
| WP_050996965.1 | FRAAL0248 | helicase | 123677 | 5.69 | 43 | 267 | Frankia-1 | F201364.dat | 1442 | 35 | 28 | 28 | 31 | 31 | 28 | 28 | 26 | 32 | 0.042% | 23 | 20 | 28 | 22 | 29 | 0.033% | -1.18 | -0.17 | 2.8E-02 | Orange |
| WP_011602340.1 | FRAAL1134 | molecular chaperone GroEL | 57494 | 4.86 | 41 | 780 | Frankia-5 | F201368.dat | 3160 | 70 | 41 | 36 | 90 | 86 | 77 | 83 | 87 | 90 | 0.263% | 63 | 76 | 74 | 68 | 76 | 0.209% | -1.18 | -0.17 | 2.3E-03 | Orange |
| WP_011601375.1 | FRAAL0110 | phosphoserine aminotransferase | 39651 | 5.05 | 13 | 121 | Frankia-5 | F201368.dat | 601 | 47 | 12 | 12 | 14 | 14 | 13 | 13 | 12 | 14 | 0.059% | 11 | 11 | 12 | 10 | 11 | 0.047% | -1.18 | -0.17 | 1.1E-03 | Orange |
| WP_011603726.1 | FRAAL2569 | hypothetical protein | 31457 | 5.16 | 15 | 132 | Frankia-5 | F201368.dat | 603 | 43 | 11 | 11 | 15 | 14 | 15 | 12 | 16 | 15 | 0.082% | 11 | 12 | 15 | 11 | 11 | 0.064% | -1.18 | -0.17 | 2.4E-02 | Orange |
| WP_011606952.1 | FRAAL5891 | beta-mannanase | 66297 | 6.52 | 22 | 473 | Frankia-4 | F201367.dat | 1316 | 36 | 19 | 19 | 54 | 52 | 51 | 48 | 54 | 52 | 0.138% | 56 | 41 | 33 | 46 | 40 | 0.110% | -1.19 | -0.17 | 3.5E-02 | Orange |
| WP_041939936.1 | FRAAL6640 | co-chaperone GrpE | 27734 | 4.52 | 18 | 163 | Frankia-5 | F201368.dat | 756 | 77 | 14 | 14 | 21 | 15 | 17 | 17 | 19 | 21 | 0.115% | 11 | 16 | 17 | 17 | 13 | 0.090% | -1.19 | -0.17 | 4.7E-02 | Orange |
| WP_041941010.1 | FRAAL5903 | thioredoxin | 31834 | 4.65 | 15 | 174 | Frankia-5 | F201368.dat | 644 | 43 | 13 | 13 | 19 | 20 | 17 | 19 | 20 | 19 | 0.106% | 12 | 14 | 18 | 17 | 18 | 0.084% | -1.19 | -0.17 | 2.1E-02 | Orange |
| WP_011602412.1 | FRAAL1210 | acetyl-/propionyl-CoA carboxylase subunit alpha | 61427 | 5.02 | 35 | 659 | Frankia-5 | F201368.dat | 2104 | 67 | 32 | 32 | 75 | 73 | 73 | 64 | 74 | 75 | 0.209% | 54 | 56 | 67 | 58 | 65 | 0.164% | -1.19 | -0.18 | 3.2E-03 | Orange |
| WP_041938858.1 | FRAAL1139 | inosine-5-monophosphate dehydrogenase | 56722 | 5.87 | 27 | 203 | Frankia-1 | F201364.dat | 992 | 49 | 20 | 20 | 28 | 28 | 21 | 21 | 21 | 20 | 0.070% | 16 | 18 | 19 | 17 | 22 | 0.055% | -1.20 | -0.18 | 3.3E-02 | Orange |
| WP_041939638.1 | FRAAL5110 | (2Fe-2S)-binding protein | 46695 | 5.78 | 24 | 427 | Frankia-3 | F201366.dat | 1437 | 56 | 23 | 23 | 50 | 48 | 46 | 50 | 48 | 41 | 0.178% | 39 | 44 | 39 | 39 | 33 | 0.140% | -1.20 | -0.18 | 5.0E-03 | Orange |
| WP_041939862.1 | FRAAL6282 | bifunctional N-acetylglucosamine-1-phosphate uridyltransferase/glucosamine-1-phosphate acetyltransferase | 55650 | 5.73 | 17 | 135 | Frankia-6 | F201369.dat | 672 | 30 | 12 | 12 | 13 | 16 | 15 | 15 | 13 | 15 | 0.047% | 13 | 11 | 12 | 12 | 13 | 0.037% | -1.20 | -0.18 | 1.5E-03 | Orange |
| WP_011606731.1 | FRAAL5654 | succinate dehydrogenase | 71200 | 5.97 | 54 | 1261 | Frankia-3 | F201366.dat | 2845 | 71 | 50 | 50 | 139 | 141 | 138 | 139 | 132 | 138 | 0.345% | 109 | 109 | 127 | 111 | 117 | 0.271% | -1.20 | -0.18 | 1.4E-04 | Orange |
| WP_011607692.1 | FRAAL6655 | fructose-bisphosphate aldolase | 36877 | 5.28 | 24 | 573 | Frankia-4 | F201367.dat | 1338 | 86 | 23 | 23 | 63 | 57 | 60 | 63 | 63 | 70 | 0.303% | 47 | 55 | 53 | 51 | 54 | 0.237% | -1.20 | -0.18 | 1.7E-03 | Orange |
| WP_041939983.1 | FRAAL6802 | heme biosynthesis protein HemY | 21169 | 4.66 | 12 | 175 | Frankia-9 | F201372.dat | 779 | 87 | 11 | 11 | 19 | 21 | 18 | 20 | 18 | 19 | 0.162% | 16 | 16 | 12 | 19 | 16 | 0.126% | -1.20 | -0.18 | 1.3E-02 | Orange |
| WP_041938846.1 | FRAAL1062 | aspartate aminotransferase | 42402 | 5.34 | 21 | 356 | Frankia-2 | F201365.dat | 1180 | 68 | 19 | 19 | 39 | 39 | 39 | 40 | 40 | 37 | 0.164% | 29 | 33 | 35 | 31 | 33 | 0.128% | -1.20 | -0.19 | 1.9E-04 | Orange |
| WP_011607623.1 | FRAAL6586 | hypothetical protein | 40197 | 5.87 | 23 | 180 | Frankia-5 | F201368.dat | 726 | 45 | 15 | 15 | 22 | 17 | 22 | 20 | 18 | 22 | 0.088% | 17 | 14 | 15 | 18 | 17 | 0.068% | -1.21 | -0.19 | 1.1E-02 | Orange |
| WP_011605806.1 | FRAAL4690 | hypothetical protein | 67743 | 5.81 | 32 | 315 | Frankia-1 | F201364.dat | 1233 | 44 | 23 | 23 | 38 | 38 | 31 | 35 | 37 | 32 | 0.091% | 22 | 29 | 34 | 25 | 32 | 0.071% | -1.21 | -0.19 | 2.2E-02 | Orange |
| WP_011603996.1 | FRAAL2849 | hypothetical protein | 55584 | 5.85 | 39 | 713 | Frankia-5 | F201368.dat | 2224 | 73 | 37 | 37 | 78 | 83 | 78 | 76 | 76 | 78 | 0.251% | 66 | 59 | 66 | 67 | 64 | 0.195% | -1.21 | -0.19 | 4.7E-05 | Orange |
| WP_011602360.1 | FRAAL1157 | succinyl-CoA synthetase subunit alpha | 29742 | 5.40 | 16 | 550 | Frankia-4 | F201367.dat | 1192 | 76 | 16 | 16 | 60 | 58 | 60 | 58 | 60 | 66 | 0.362% | 47 | 47 | 51 | 47 | 56 | 0.281% | -1.21 | -0.19 | 7.9E-04 | Orange |
| WP_041939741.1 | FRAAL5628 | translocation protein TolB | 41400 | 8.87 | 12 | 83 | Frankia-3 | F201366.dat | 413 | 30 | 10 | 10 | 10 | 9 | 9 | 10 | 8 | 10 | 0.040% | 9 | 8 | 6 | 9 | 5 | 0.030% | -1.21 | -0.19 | 3.9E-02 | Orange |
| WP_011601745.1 | FRAAL0492 | RNA-binding protein | 93282 | 6.16 | 45 | 370 | Frankia-8 | F201371.dat | 1493 | 41 | 32 | 32 | 40 | 41 | 40 | 41 | 42 | 40 | 0.078% | 29 | 30 | 40 | 31 | 36 | 0.060% | -1.22 | -0.20 | 3.5E-03 | Orange |
| WP_011602299.1 | FRAAL1078 | elongation factor G | 76727 | 4.96 | 49 | 997 | Frankia-10 | F201373.dat | 2503 | 76 | 45 | 45 | 104 | 117 | 113 | 107 | 113 | 99 | 0.255% | 92 | 77 | 96 | 79 | 104 | 0.197% | -1.22 | -0.20 | 5.0E-03 | Orange |
| WP_011605803.1 | FRAAL4687 | hypothetical protein | 44555 | 5.03 | 19 | 377 | Frankia-5 | F201368.dat | 1157 | 52 | 18 | 18 | 44 | 42 | 39 | 37 | 46 | 44 | 0.167% | 26 | 29 | 40 | 34 | 40 | 0.128% | -1.22 | -0.20 | 2.2E-02 | Orange |
| WP_011607768.1 | FRAAL6731 | hypothetical protein | 44193 | 4.49 | 10 | 79 | Frankia-3 | F201366.dat | 401 | 30 | 8 | 8 | 10 | 8 | 8 | 10 | 10 | 8 | 0.036% | 6 | 6 | 9 | 6 | 8 | 0.027% | -1.23 | -0.20 | 2.7E-02 | Orange |
| WP_009742053.1 | FRAAL6126 | ferredoxin | 11541 | 3.74 | 8 | 177 | Frankia-1 | F201364.dat | 595 | 99 | 7 | 7 | 19 | 19 | 20 | 19 | 20 | 20 | 0.303% | 19 | 18 | 13 | 16 | 13 | 0.231% | -1.23 | -0.20 | 9.1E-03 | Orange |
| WP_011604323.1 | FRAAL3174 | endoglucanase | 65460 | 5.41 | 17 | 157 | Frankia-2 | F201365.dat | 854 | 23 | 13 | 13 | 17 | 19 | 17 | 19 | 17 | 15 | 0.047% | 13 | 19 | 14 | 15 | 9 | 0.036% | -1.23 | -0.20 | 4.6E-02 | Orange |
| WP_011606629.1 | FRAAL5545 | twin-arginine translocation pathway signal protein | 42156 | 7.21 | 9 | 137 | Frankia-2 | F201365.dat | 696 | 40 | 9 | 9 | 17 | 15 | 17 | 13 | 15 | 16 | 0.064% | 12 | 16 | 10 | 11 | 12 | 0.049% | -1.23 | -0.20 | 1.9E-02 | Orange |
| WP_041940203.1 | FRAAL1283 | S-adenosyl-L-homocysteine hydrolase | 44878 | 5.33 | 4 | 39 | Frankia-3 | F201366.dat | 211 | 13 | 4 | 4 | 5 | 5 | 4 | 5 | 3 | 5 | 0.017% | 3 | 3 | 4 | 3 | 4 | 0.013% | -1.23 | -0.20 | 4.5E-02 | Orange |
| WP_041939451.1 | FRAAL4107 | peptidase M13 | 72941 | 4.95 | 26 | 224 | Frankia-1 | F201364.dat | 825 | 33 | 18 | 18 | 27 | 27 | 24 | 24 | 26 | 23 | 0.061% | 15 | 20 | 23 | 18 | 24 | 0.046% | -1.23 | -0.21 | 1.4E-02 | Orange |
| WP_011602985.1 | FRAAL1805 | carnitine O-acetyltransferase | 66409 | 5.10 | 24 | 184 | Frankia-4 | F201367.dat | 1072 | 42 | 18 | 18 | 20 | 24 | 18 | 21 | 20 | 19 | 0.055% | 14 | 17 | 20 | 13 | 18 | 0.042% | -1.23 | -0.21 | 2.1E-02 | Orange |
| WP_041939658.1 | FRAAL5210 | phenylalanine--tRNA ligase subunit beta | 87677 | 5.09 | 32 | 298 | Frankia-3 | F201366.dat | 1348 | 38 | 27 | 27 | 39 | 36 | 29 | 39 | 33 | 28 | 0.067% | 23 | 27 | 34 | 23 | 26 | 0.051% | -1.23 | -0.21 | 2.9E-02 | Orange |
| WP_011606116.1 | FRAAL5004 | histidinol dehydrogenase | 44136 | 5.17 | 11 | 104 | Frankia-4 | F201367.dat | 480 | 37 | 10 | 10 | 12 | 11 | 11 | 12 | 12 | 12 | 0.047% | 9 | 7 | 12 | 8 | 10 | 0.035% | -1.24 | -0.21 | 1.6E-02 | Orange |
| WP_050997465.1 | FRAAL6242 | peptidase M28 | 47152 | 5.22 | 12 | 75 | Frankia-1 | F201364.dat | 368 | 28 | 10 | 10 | 11 | 11 | 7 | 8 | 9 | 7 | 0.032% | 5 | 7 | 8 | 6 | 7 | 0.024% | -1.24 | -0.21 | 4.1E-02 | Orange |
| WP_011606261.1 | FRAAL5157 | lipoyl synthase | 36341 | 6.34 | 7 | 37 | Frankia-2 | F201365.dat | 191 | 20 | 5 | 5 | 5 | 3 | 5 | 5 | 4 | 4 | 0.021% | 3 | 3 | 4 | 3 | 3 | 0.015% | -1.24 | -0.21 | 4.0E-02 | Orange |
| WP_011603241.1 | FRAAL2075 | valyl-tRNA synthetase | 98131 | 5.47 | 35 | 308 | Frankia-2 | F201365.dat | 1194 | 36 | 27 | 27 | 35 | 38 | 35 | 34 | 31 | 33 | 0.062% | 24 | 30 | 35 | 22 | 26 | 0.047% | -1.24 | -0.21 | 1.5E-02 | Orange |
| WP_011604445.1 | FRAAL3297 | isocitrate dehydrogenase | 78781 | 4.94 | 60 | 1204 | Frankia-1 | F201364.dat | 3356 | 81 | 54 | 54 | 147 | 147 | 125 | 138 | 128 | 129 | 0.302% | 101 | 107 | 125 | 92 | 112 | 0.230% | -1.24 | -0.21 | 2.6E-03 | Orange |
| WP_041939813.1 | FRAAL6042 | peptidase M16 | 46673 | 4.83 | 14 | 129 | Frankia-2 | F201365.dat | 513 | 31 | 12 | 12 | 15 | 12 | 15 | 13 | 14 | 18 | 0.055% | 10 | 10 | 14 | 11 | 12 | 0.041% | -1.24 | -0.22 | 2.3E-02 | Orange |
| WP_011602626.1 | FRAAL1431 | dimethylallyltransferase | 35343 | 4.77 | 18 | 284 | Frankia-5 | F201368.dat | 848 | 43 | 16 | 16 | 36 | 30 | 32 | 25 | 35 | 36 | 0.160% | 19 | 28 | 26 | 24 | 29 | 0.120% | -1.24 | -0.22 | 2.1E-02 | Orange |
| WP_041938938.1 | FRAAL1544 | LuxR family transcriptional regulator | 31571 | 10.99 | 18 | 201 | Frankia-3 | F201366.dat | 664 | 61 | 15 | 15 | 27 | 23 | 22 | 27 | 20 | 20 | 0.127% | 16 | 21 | 17 | 18 | 17 | 0.095% | -1.24 | -0.22 | 8.9E-03 | Orange |
| WP_011603087.1 | FRAAL1912 | ATP-dependent Clp protease ATP-binding subunit ClpX | 46977 | 5.17 | 11 | 109 | Frankia-1 | F201364.dat | 640 | 31 | 11 | 11 | 14 | 14 | 10 | 13 | 12 | 12 | 0.046% | 9 | 10 | 10 | 10 | 9 | 0.034% | -1.25 | -0.22 | 4.0E-03 | Orange |
| WP_041939913.1 | FRAAL6527 | hypothetical protein | 24649 | 10.20 | 4 | 109 | Frankia-3 | F201366.dat | 345 | 40 | 4 | 4 | 13 | 11 | 13 | 13 | 11 | 13 | 0.088% | 8 | 9 | 12 | 10 | 9 | 0.066% | -1.25 | -0.22 | 7.2E-03 | Orange |
| WP_011603254.1 | FRAAL2088 | 1-deoxy-D-xylulose-5-phosphate synthase | 69522 | 5.30 | 41 | 516 | Frankia-3 | F201366.dat | 1970 | 64 | 35 | 35 | 63 | 56 | 60 | 63 | 50 | 58 | 0.147% | 49 | 43 | 46 | 48 | 43 | 0.111% | -1.25 | -0.22 | 8.5E-04 | Orange |
| WP_011603193.1 | FRAAL2021 | transketolase | 65690 | 5.11 | 41 | 930 | Frankia-4 | F201367.dat | 2515 | 76 | 35 | 35 | 110 | 104 | 104 | 106 | 110 | 93 | 0.281% | 77 | 82 | 92 | 76 | 86 | 0.212% | -1.25 | -0.22 | 4.7E-04 | Orange |
| WP_011602409.1 | FRAAL1206 | pyridine nucleotide-disulfide oxidoreductase | 51246 | 5.67 | 13 | 71 | Frankia-4 | F201367.dat | 390 | 27 | 9 | 9 | 10 | 8 | 8 | 8 | 10 | 6 | 0.028% | 6 | 4 | 7 | 6 | 8 | 0.020% | -1.25 | -0.22 | 4.3E-02 | Orange |
| WP_041938599.1 | FRAAL0052 | glucose-6-phosphate isomerase | 60041 | 5.33 | 24 | 375 | Frankia-3 | F201366.dat | 1337 | 48 | 21 | 21 | 42 | 47 | 39 | 42 | 41 | 40 | 0.124% | 28 | 32 | 38 | 33 | 35 | 0.093% | -1.25 | -0.22 | 2.0E-03 | Orange |
| WP_011603191.1 | FRAAL2019 | glycyl-tRNA synthetase subunit alpha | 112788 | 5.01 | 44 | 339 | Frankia-1 | F201364.dat | 1547 | 36 | 32 | 32 | 46 | 46 | 34 | 37 | 35 | 37 | 0.060% | 24 | 24 | 35 | 30 | 37 | 0.045% | -1.25 | -0.22 | 2.7E-02 | Orange |
| WP_011606693.1 | FRAAL5612 | malate dehydrogenase | 34520 | 5.20 | 28 | 574 | Frankia-2 | F201365.dat | 1566 | 74 | 27 | 27 | 69 | 57 | 69 | 57 | 68 | 69 | 0.331% | 45 | 49 | 56 | 47 | 57 | 0.248% | -1.25 | -0.23 | 3.9E-03 | Orange |
| WP_011603035.1 | FRAAL1858 | aminopeptidase N | 94232 | 4.78 | 52 | 538 | Frankia-1 | F201364.dat | 2422 | 59 | 39 | 39 | 65 | 65 | 63 | 57 | 56 | 59 | 0.114% | 43 | 41 | 58 | 43 | 53 | 0.085% | -1.26 | -0.23 | 5.5E-03 | Orange |
| WP_041939844.1 | FRAAL6200 | haloacid dehalogenase | 30684 | 5.21 | 8 | 96 | Frankia-7 | F201370.dat | 260 | 27 | 6 | 6 | 10 | 10 | 11 | 11 | 12 | 10 | 0.063% | 6 | 10 | 10 | 8 | 8 | 0.046% | -1.26 | -0.23 | 1.0E-02 | Orange |
| WP_011606298.1 | FRAAL5204 | ornithine carbamoyltransferase | 35892 | 5.20 | 12 | 87 | Frankia-5 | F201368.dat | 535 | 49 | 9 | 9 | 11 | 11 | 9 | 8 | 10 | 11 | 0.049% | 7 | 9 | 10 | 5 | 7 | 0.036% | -1.26 | -0.23 | 3.5E-02 | Orange |
| WP_041938863.1 | FRAAL1156 | succinyl-CoA synthetase subunit beta | 40161 | 4.76 | 36 | 1030 | Frankia-5 | F201368.dat | 2026 | 75 | 33 | 33 | 120 | 107 | 115 | 110 | 122 | 120 | 0.510% | 86 | 93 | 99 | 86 | 92 | 0.382% | -1.26 | -0.23 | 1.2E-04 | Orange |
| WP_041939077.1 | FRAAL2207 | isoleucyl-tRNA synthetase | 116864 | 5.35 | 24 | 189 | Frankia-1 | F201364.dat | 925 | 20 | 19 | 19 | 25 | 25 | 20 | 22 | 19 | 20 | 0.032% | 15 | 14 | 18 | 15 | 21 | 0.024% | -1.26 | -0.23 | 1.3E-02 | Orange |
| WP_011602217.1 | FRAAL0990 | methionyl-tRNA synthetase | 55368 | 5.20 | 22 | 300 | Frankia-1 | F201364.dat | 1303 | 57 | 21 | 21 | 37 | 37 | 33 | 35 | 32 | 31 | 0.108% | 24 | 23 | 32 | 26 | 27 | 0.080% | -1.26 | -0.23 | 2.7E-03 | Orange |
| WP_050997261.1 | FRAAL6210 | leucyl-tRNA synthetase | 115084 | 5.27 | 35 | 334 | Frankia-1 | F201364.dat | 1496 | 31 | 28 | 28 | 44 | 44 | 36 | 36 | 37 | 34 | 0.058% | 29 | 26 | 38 | 25 | 29 | 0.043% | -1.26 | -0.23 | 1.2E-02 | Orange |
| WP_011607577.1 | FRAAL6537 | proline--tRNA ligase | 50933 | 5.27 | 23 | 221 | Frankia-2 | F201365.dat | 1038 | 65 | 21 | 21 | 24 | 24 | 24 | 25 | 28 | 23 | 0.087% | 19 | 20 | 19 | 21 | 18 | 0.064% | -1.26 | -0.23 | 3.2E-04 | Orange |
| WP_011606065.1 | FRAAL4951 | methylmalonyl-CoA mutase | 65100 | 4.93 | 24 | 262 | Frankia-3 | F201366.dat | 1206 | 45 | 21 | 21 | 28 | 28 | 32 | 28 | 30 | 29 | 0.081% | 18 | 22 | 29 | 23 | 23 | 0.059% | -1.27 | -0.24 | 5.1E-03 | Orange |
| WP_011602795.1 | FRAAL1606 | aminopeptidase | 94151 | 5.13 | 19 | 160 | Frankia-1 | F201364.dat | 768 | 24 | 16 | 16 | 21 | 21 | 19 | 16 | 18 | 16 | 0.034% | 14 | 9 | 20 | 14 | 13 | 0.025% | -1.27 | -0.24 | 4.0E-02 | Orange |
| WP_041938823.1 | FRAAL0963 | glycine cleavage system protein T | 39762 | 5.90 | 13 | 90 | Frankia-3 | F201366.dat | 568 | 40 | 11 | 11 | 12 | 9 | 11 | 12 | 11 | 8 | 0.046% | 8 | 7 | 8 | 8 | 8 | 0.033% | -1.27 | -0.24 | 8.8E-03 | Orange |
| WP_011607915.1 | FRAAL6880 | single-stranded DNA-binding protein | 19827 | 4.60 | 10 | 65 | Frankia-5 | F201368.dat | 273 | 50 | 7 | 7 | 10 | 6 | 8 | 6 | 7 | 10 | 0.067% | 4 | 5 | 7 | 6 | 6 | 0.048% | -1.27 | -0.24 | 4.1E-02 | Orange |
| WP_011606192.1 | FRAAL5083 | phospho-2-dehydro-3-deoxyheptonate aldolase | 50035 | 5.48 | 9 | 70 | Frankia-3 | F201366.dat | 301 | 19 | 8 | 8 | 8 | 8 | 6 | 8 | 8 | 10 | 0.029% | 6 | 7 | 5 | 6 | 6 | 0.020% | -1.29 | -0.25 | 1.2E-02 | Orange |
| WP_011607008.1 | FRAAL5950 | threonine synthase | 41672 | 5.84 | 16 | 109 | Frankia-3 | F201366.dat | 762 | 49 | 12 | 12 | 14 | 14 | 9 | 14 | 13 | 12 | 0.053% | 9 | 11 | 12 | 7 | 8 | 0.038% | -1.29 | -0.25 | 2.6E-02 | Orange |
| WP_011607011.1 | FRAAL5953 | arginine--tRNA ligase | 58243 | 5.39 | 22 | 199 | Frankia-5 | F201368.dat | 1140 | 46 | 17 | 17 | 26 | 24 | 23 | 19 | 21 | 26 | 0.069% | 14 | 18 | 21 | 14 | 19 | 0.050% | -1.30 | -0.26 | 9.5E-03 | Orange |
| WP_011606818.1 | FRAAL5744 | histidyl-tRNA synthetase | 48184 | 5.21 | 23 | 181 | Frankia-4 | F201367.dat | 1046 | 59 | 20 | 20 | 24 | 19 | 19 | 20 | 24 | 21 | 0.076% | 13 | 15 | 20 | 14 | 16 | 0.055% | -1.30 | -0.26 | 5.6E-03 | Orange |
| WP_041938917.1 | FRAAL1430 | phytoene dehydrogenase | 60221 | 7.19 | 21 | 165 | Frankia-3 | F201366.dat | 918 | 44 | 16 | 16 | 21 | 19 | 18 | 21 | 21 | 15 | 0.056% | 14 | 14 | 14 | 14 | 15 | 0.040% | -1.30 | -0.26 | 2.1E-03 | Orange |
| WP_011607084.1 | FRAAL6032 | hydrolase | 49234 | 4.71 | 15 | 172 | Frankia-4 | F201367.dat | 796 | 41 | 14 | 14 | 23 | 17 | 17 | 21 | 23 | 20 | 0.071% | 10 | 11 | 21 | 15 | 17 | 0.051% | -1.30 | -0.27 | 3.6E-02 | Orange |
| WP_011602405.1 | FRAAL1202 | aspartyl-tRNA synthetase | 66701 | 5.60 | 31 | 278 | Frankia-4 | F201367.dat | 1156 | 44 | 24 | 24 | 36 | 32 | 29 | 34 | 36 | 27 | 0.085% | 23 | 22 | 31 | 21 | 23 | 0.061% | -1.30 | -0.27 | 6.9E-03 | Orange |
| WP_011601861.1 | FRAAL0612 | DNA polymerase III subunit gamma/tau | 99864 | 6.10 | 19 | 96 | Frankia-1 | F201364.dat | 475 | 19 | 11 | 11 | 11 | 11 | 11 | 10 | 12 | 11 | 0.020% | 9 | 8 | 6 | 8 | 10 | 0.014% | -1.30 | -0.27 | 2.7E-03 | Orange |
| WP_041939928.1 | FRAAL6590 | ATP phosphoribosyltransferase | 30229 | 5.17 | 19 | 149 | Frankia-2 | F201365.dat | 730 | 63 | 16 | 16 | 17 | 17 | 17 | 16 | 17 | 18 | 0.100% | 10 | 11 | 14 | 13 | 16 | 0.071% | -1.30 | -0.27 | 2.8E-03 | Orange |
| WP_041939522.1 | FRAAL4507 | hypothetical protein | 47588 | 9.69 | 11 | 96 | Frankia-5 | F201368.dat | 477 | 38 | 10 | 10 | 13 | 10 | 8 | 12 | 12 | 13 | 0.041% | 9 | 9 | 9 | 8 | 6 | 0.029% | -1.30 | -0.27 | 1.5E-02 | Orange |
| WP_050997321.1 | FRAAL0998 | hypothetical protein | 24163 | 6.80 | 6 | 43 | Frankia-8 | F201371.dat | 212 | 33 | 5 | 5 | 5 | 4 | 6 | 5 | 6 | 4 | 0.037% | 2 | 3 | 5 | 4 | 4 | 0.025% | -1.30 | -0.27 | 3.6E-02 | Orange |
| WP_041939400.1 | FRAAL3855 | (4Fe-4S)-binding protein | 11752 | 4.41 | 7 | 103 | Frankia-4 | F201367.dat | 455 | 95 | 7 | 7 | 13 | 12 | 11 | 15 | 13 | 8 | 0.179% | 8 | 13 | 7 | 9 | 7 | 0.126% | -1.31 | -0.27 | 4.9E-02 | Orange |
| WP_011606921.1 | FRAAL5853 | aspartyl/glutamyl-tRNA amidotransferase subunit B | 53948 | 5.13 | 34 | 320 | Frankia-4 | F201367.dat | 1450 | 56 | 29 | 29 | 44 | 36 | 36 | 31 | 44 | 35 | 0.120% | 28 | 24 | 29 | 26 | 31 | 0.086% | -1.31 | -0.27 | 3.4E-03 | Orange |
| WP_011606161.1 | FRAAL5051 | pyruvate dehydrogenase E1 | 102529 | 5.42 | 57 | 1079 | Frankia-5 | F201368.dat | 2767 | 55 | 53 | 53 | 119 | 130 | 125 | 124 | 115 | 119 | 0.213% | 92 | 85 | 103 | 88 | 98 | 0.153% | -1.31 | -0.27 | 5.4E-05 | Orange |
| WP_011605532.1 | FRAAL4409 | cyclopropane-fatty-acyl-phospholipid synthase | 50622 | 8.46 | 11 | 64 | Frankia-5 | F201368.dat | 388 | 25 | 8 | 8 | 9 | 9 | 7 | 6 | 6 | 9 | 0.026% | 7 | 4 | 6 | 5 | 5 | 0.018% | -1.31 | -0.27 | 2.3E-02 | Orange |
| WP_011602743.1 | FRAAL1555 | TenA family transcriptional regulator | 25450 | 4.93 | 5 | 27 | Frankia-7 | F201370.dat | 151 | 16 | 3 | 3 | 3 | 3 | 3 | 3 | 3 | 4 | 0.022% | 1 | 3 | 2 | 2 | 3 | 0.015% | -1.31 | -0.27 | 4.0E-02 | Orange |
| WP_011602324.1 | FRAAL1117 | glutamine amidotransferase | 70401 | 5.61 | 21 | 159 | Frankia-3 | F201366.dat | 915 | 37 | 18 | 18 | 20 | 21 | 18 | 20 | 16 | 16 | 0.046% | 13 | 15 | 16 | 14 | 10 | 0.033% | -1.32 | -0.27 | 6.6E-03 | Orange |
| WP_050997010.1 | FRAAL1025 | dehypoxanthine futalosine cyclase | 43891 | 5.97 | 10 | 78 | Frankia-1 | F201364.dat | 368 | 30 | 8 | 8 | 10 | 10 | 8 | 10 | 9 | 8 | 0.037% | 4 | 8 | 7 | 8 | 6 | 0.025% | -1.32 | -0.27 | 1.2E-02 | Orange |
| WP_011607303.1 | FRAAL6253 | NDP-hexose 3-C-methyltransferase | 46478 | 4.85 | 14 | 148 | Frankia-2 | F201365.dat | 765 | 50 | 14 | 14 | 18 | 20 | 18 | 16 | 15 | 16 | 0.065% | 12 | 12 | 11 | 14 | 14 | 0.046% | -1.32 | -0.28 | 1.8E-03 | Orange |
| WP_011604963.1 | FRAAL3826 | calcium-binding protein | 45803 | 4.89 | 18 | 148 | Frankia-1 | F201364.dat | 704 | 41 | 15 | 15 | 21 | 21 | 13 | 13 | 19 | 19 | 0.066% | 12 | 12 | 13 | 12 | 14 | 0.046% | -1.32 | -0.28 | 1.7E-02 | Orange |
| WP_011607293.1 | FRAAL6244 | hypothetical protein | 34874 | 5.38 | 10 | 76 | Frankia-5 | F201368.dat | 311 | 37 | 8 | 8 | 11 | 9 | 9 | 6 | 9 | 11 | 0.045% | 7 | 6 | 8 | 6 | 5 | 0.031% | -1.32 | -0.28 | 1.8E-02 | Orange |
| WP_041939599.1 | FRAAL4950 | methylmalonyl-CoA mutase | 80109 | 5.18 | 31 | 244 | Frankia-1 | F201364.dat | 1139 | 43 | 22 | 22 | 34 | 34 | 25 | 29 | 27 | 25 | 0.062% | 18 | 17 | 26 | 20 | 23 | 0.044% | -1.33 | -0.29 | 7.8E-03 | Orange |
| WP_011602289.1 | FRAAL1064 | transcription termination/antitermination factor NusG | 31458 | 4.25 | 18 | 258 | Frankia-4 | F201367.dat | 775 | 43 | 17 | 17 | 32 | 29 | 29 | 27 | 32 | 31 | 0.168% | 20 | 23 | 25 | 20 | 22 | 0.118% | -1.33 | -0.29 | 1.8E-04 | Orange |
| WP_011602756.1 | FRAAL1569 | FAD-dependent oxidoreductase | 63438 | 6.89 | 19 | 144 | Frankia-3 | F201366.dat | 747 | 37 | 15 | 15 | 19 | 19 | 14 | 19 | 17 | 14 | 0.047% | 13 | 12 | 15 | 9 | 12 | 0.032% | -1.33 | -0.29 | 9.0E-03 | Orange |
| WP_011603155.1 | FRAAL1982 | acyl-CoA dehydrogenase | 41548 | 5.79 | 10 | 81 | Frankia-4 | F201367.dat | 359 | 35 | 9 | 9 | 11 | 7 | 10 | 8 | 11 | 11 | 0.040% | 5 | 7 | 9 | 7 | 6 | 0.028% | -1.33 | -0.29 | 1.9E-02 | Orange |
| WP_041938731.1 | FRAAL0598 | cytochrome D oxidase subunit I | 81220 | 9.60 | 4 | 25 | Frankia-3 | F201366.dat | 154 | 5 | 3 | 3 | 4 | 3 | 3 | 4 | 2 | 3 | 0.007% | 2 | 2 | 2 | 2 | 2 | 0.004% | -1.33 | -0.29 | 3.3E-02 | Orange |
| WP_041938906.1 | FRAAL1401 | peroxidase | 44072 | 6.10 | 16 | 102 | Frankia-5 | F201368.dat | 594 | 38 | 13 | 13 | 16 | 13 | 8 | 11 | 11 | 16 | 0.048% | 7 | 11 | 8 | 10 | 7 | 0.033% | -1.33 | -0.29 | 3.6E-02 | Orange |
| WP_011607001.1 | FRAAL5942 | serine hydroxymethyltransferase | 44079 | 5.79 | 9 | 53 | Frankia-5 | F201368.dat | 362 | 22 | 7 | 7 | 8 | 6 | 4 | 5 | 8 | 8 | 0.025% | 4 | 6 | 4 | 4 | 4 | 0.017% | -1.33 | -0.29 | 3.9E-02 | Orange |
| WP_050997057.1 | FRAAL2191 | UDP-N-acetylmuramoylalanyl-D-glutamate--2.6-diaminopimelate ligase | 54209 | 5.72 | 6 | 39 | Frankia-4 | F201367.dat | 296 | 14 | 5 | 5 | 6 | 5 | 5 | 4 | 6 | 3 | 0.015% | 2 | 5 | 3 | 3 | 3 | 0.010% | -1.33 | -0.29 | 4.2E-02 | Orange |
| WP_050997107.1 | FRAAL2825 | hypothetical protein | 48577 | 4.77 | 14 | 60 | Frankia-2 | F201365.dat | 397 | 27 | 8 | 8 | 10 | 7 | 10 | 7 | 6 | 5 | 0.026% | 3 | 6 | 6 | 4 | 6 | 0.017% | -1.33 | -0.29 | 4.6E-02 | Orange |
| WP_041939794.1 | FRAAL5981 | alpha-ketoglutarate decarboxylase | 137394 | 5.86 | 72 | 1251 | Frankia-3 | F201366.dat | 3932 | 59 | 65 | 65 | 152 | 149 | 147 | 152 | 121 | 147 | 0.186% | 113 | 82 | 126 | 107 | 107 | 0.131% | -1.34 | -0.29 | 2.0E-03 | Orange |
| WP_050997023.1 | FRAAL1433 | 4-hydroxy-3-methylbut-2-enyl diphosphate reductase | 66377 | 5.48 | 17 | 142 | Frankia-1 | F201364.dat | 814 | 33 | 16 | 16 | 21 | 21 | 14 | 19 | 16 | 12 | 0.044% | 12 | 13 | 14 | 9 | 12 | 0.030% | -1.34 | -0.29 | 2.2E-02 | Orange |
| WP_041940589.1 | FRAAL3607 | dihydrolipoyl dehydrogenase | 49442 | 5.32 | 33 | 577 | Frankia-3 | F201366.dat | 2178 | 81 | 32 | 32 | 74 | 70 | 61 | 74 | 69 | 57 | 0.239% | 41 | 52 | 55 | 48 | 50 | 0.168% | -1.34 | -0.29 | 1.2E-03 | Orange |
| WP_050997043.1 | FRAAL1791 | lysyl-tRNA synthetase | 65096 | 5.39 | 24 | 217 | Frankia-4 | F201367.dat | 1031 | 55 | 22 | 22 | 33 | 25 | 24 | 24 | 33 | 19 | 0.069% | 14 | 18 | 23 | 18 | 19 | 0.048% | -1.34 | -0.29 | 1.9E-02 | Orange |
| WP_011603084.1 | FRAAL1909 | trigger factor | 53221 | 4.31 | 28 | 337 | Frankia-4 | F201367.dat | 1409 | 52 | 25 | 25 | 42 | 44 | 37 | 36 | 42 | 35 | 0.130% | 30 | 25 | 30 | 28 | 30 | 0.090% | -1.34 | -0.30 | 5.0E-04 | Orange |
| WP_011605409.1 | FRAAL4283 | hypothetical protein | 43378 | 6.46 | 13 | 126 | Frankia-5 | F201368.dat | 456 | 35 | 10 | 10 | 16 | 14 | 12 | 16 | 15 | 16 | 0.060% | 9 | 12 | 11 | 9 | 12 | 0.041% | -1.34 | -0.30 | 2.1E-03 | Orange |
| WP_011603018.1 | FRAAL1839 | oxidoreductase | 32116 | 5.52 | 9 | 51 | Frankia-1 | F201364.dat | 374 | 37 | 7 | 7 | 7 | 7 | 6 | 4 | 7 | 6 | 0.033% | 4 | 4 | 6 | 5 | 2 | 0.022% | -1.35 | -0.30 | 3.5E-02 | Orange |
| WP_011606255.1 | FRAAL5151 | dihydrolipoamide dehydrogenase | 47814 | 5.37 | 30 | 565 | Frankia-5 | F201368.dat | 1838 | 61 | 28 | 28 | 63 | 68 | 64 | 67 | 63 | 63 | 0.243% | 42 | 54 | 54 | 47 | 43 | 0.169% | -1.35 | -0.30 | 1.5E-04 | Orange |
| WP_011604791.1 | FRAAL3652 | 3-oxoacyl-ACP synthase | 39594 | 5.61 | 12 | 98 | Frankia-1 | F201364.dat | 500 | 37 | 10 | 10 | 13 | 13 | 12 | 10 | 11 | 11 | 0.051% | 7 | 7 | 10 | 8 | 9 | 0.035% | -1.35 | -0.30 | 1.6E-03 | Orange |
| WP_011606918.1 | FRAAL5851 | acetolactate synthase | 63197 | 5.37 | 12 | 138 | Frankia-3 | F201366.dat | 657 | 26 | 12 | 12 | 16 | 18 | 13 | 16 | 17 | 16 | 0.045% | 9 | 9 | 15 | 13 | 12 | 0.031% | -1.35 | -0.30 | 7.7E-03 | Orange |
| WP_011602268.1 | FRAAL1041 | NADH dehydrogenase subunit J | 35138 | 4.90 | 5 | 37 | Frankia-2 | F201365.dat | 176 | 26 | 4 | 4 | 6 | 4 | 6 | 4 | 4 | 4 | 0.022% | 3 | 2 | 4 | 4 | 2 | 0.014% | -1.35 | -0.30 | 2.4E-02 | Orange |
| WP_041939835.1 | FRAAL6152 | aspartate ammonia-lyase | 48998 | 5.26 | 31 | 599 | Frankia-5 | F201368.dat | 1571 | 58 | 28 | 28 | 70 | 70 | 63 | 67 | 75 | 70 | 0.251% | 45 | 55 | 55 | 48 | 51 | 0.175% | -1.35 | -0.30 | 9.0E-05 | Orange |
| WP_011602754.1 | FRAAL1567 | 6-phosphogluconate dehydrogenase | 36176 | 5.00 | 21 | 331 | Frankia-4 | F201367.dat | 1022 | 64 | 18 | 18 | 42 | 40 | 36 | 36 | 42 | 37 | 0.188% | 22 | 30 | 32 | 24 | 32 | 0.130% | -1.35 | -0.30 | 1.5E-03 | Orange |
| WP_009741523.1 | FRAAL6813 | nitrogenase iron protein | 31335 | 4.90 | 34 | 1505 | Frankia-5 | F201368.dat | 2214 | 72 | 32 | 32 | 191 | 165 | 172 | 162 | 176 | 191 | 0.986% | 121 | 137 | 121 | 128 | 132 | 0.687% | -1.35 | -0.30 | 3.2E-05 | Orange |
| WP_011607258.1 | FRAAL6208 | hypothetical protein | 71351 | 5.14 | 18 | 143 | Frankia-4 | F201367.dat | 717 | 26 | 13 | 12 | 18 | 17 | 15 | 17 | 18 | 16 | 0.042% | 10 | 10 | 18 | 12 | 10 | 0.028% | -1.35 | -0.30 | 1.1E-02 | Orange |
| WP_011603274.1 | FRAAL2109 | sulfate adenylyltransferase | 45838 | 5.67 | 14 | 82 | Frankia-4 | F201367.dat | 474 | 36 | 10 | 10 | 11 | 8 | 10 | 8 | 11 | 11 | 0.037% | 6 | 8 | 9 | 5 | 6 | 0.025% | -1.36 | -0.31 | 1.2E-02 | Orange |
| WP_011602811.1 | FRAAL1622 | NADH dehydrogenase | 52891 | 7.90 | 34 | 658 | Frankia-1 | F201364.dat | 2156 | 78 | 32 | 32 | 85 | 85 | 76 | 77 | 73 | 69 | 0.256% | 50 | 58 | 55 | 61 | 54 | 0.177% | -1.36 | -0.31 | 1.2E-04 | Orange |
| WP_011607680.1 | FRAAL6643 | chaperone protein ClpB | 94751 | 5.16 | 34 | 224 | Frankia-1 | F201364.dat | 1119 | 31 | 27 | 24 | 28 | 28 | 23 | 23 | 28 | 28 | 0.049% | 16 | 19 | 21 | 18 | 20 | 0.033% | -1.36 | -0.31 | 6.7E-04 | Orange |
| WP_041940999.1 | FRAAL5831 | serine/threonine protein kinase | 71645 | 6.00 | 11 | 87 | Frankia-5 | F201368.dat | 279 | 17 | 9 | 9 | 10 | 11 | 8 | 12 | 10 | 10 | 0.025% | 7 | 5 | 7 | 9 | 8 | 0.017% | -1.37 | -0.31 | 6.3E-03 | Orange |
| WP_041939492.1 | FRAAL4376 | diphosphate--fructose-6-phosphate 1-phosphotransferase | 48328 | 5.98 | 23 | 310 | Frankia-2 | F201365.dat | 1245 | 65 | 20 | 20 | 35 | 37 | 35 | 39 | 35 | 34 | 0.133% | 26 | 25 | 29 | 25 | 25 | 0.091% | -1.37 | -0.32 | 1.5E-05 | Orange |
| WP_041938976.1 | FRAAL1714 | glycosyl hydrolase | 41572 | 5.26 | 8 | 47 | Frankia-1 | F201364.dat | 224 | 22 | 6 | 6 | 6 | 6 | 5 | 6 | 6 | 5 | 0.024% | 5 | 5 | 2 | 4 | 3 | 0.015% | -1.38 | -0.32 | 1.4E-02 | Orange |
| WP_011607010.1 | FRAAL5952 | diaminopimelate decarboxylase | 49710 | 5.14 | 14 | 59 | Frankia-1 | F201364.dat | 525 | 29 | 9 | 9 | 9 | 9 | 7 | 6 | 8 | 5 | 0.025% | 7 | 4 | 6 | 2 | 5 | 0.016% | -1.38 | -0.32 | 4.2E-02 | Orange |
| WP_011607832.1 | FRAAL6798 | 2-oxoacid ferredoxin oxidoreductase subunit beta | 37297 | 7.18 | 25 | 571 | Frankia-5 | F201368.dat | 1480 | 76 | 24 | 24 | 69 | 65 | 64 | 64 | 70 | 69 | 0.318% | 47 | 51 | 49 | 47 | 45 | 0.216% | -1.38 | -0.32 | 1.7E-06 | Orange |
| WP_011604752.1 | FRAAL3613 | diacylglycerol O-acyltransferase | 52224 | 9.76 | 23 | 221 | Frankia-1 | F201364.dat | 944 | 51 | 19 | 19 | 25 | 25 | 23 | 30 | 24 | 27 | 0.088% | 22 | 21 | 17 | 19 | 13 | 0.059% | -1.38 | -0.32 | 3.2E-03 | Orange |
| WP_011603231.1 | FRAAL2064 | aconitate hydratase | 98720 | 4.80 | 53 | 1374 | Frankia-2 | F201365.dat | 3082 | 56 | 51 | 51 | 164 | 170 | 164 | 154 | 157 | 153 | 0.288% | 113 | 104 | 128 | 109 | 122 | 0.196% | -1.38 | -0.32 | 1.8E-05 | Orange |
| WP_011604611.1 | FRAAL3467 | branched-chain amino acid ABC transporter substrate-binding protein | 41803 | 4.92 | 4 | 21 | Frankia-5 | F201368.dat | 148 | 9 | 3 | 3 | 4 | 3 | 2 | 2 | 2 | 4 | 0.011% | 1 | 2 | 2 | 2 | 1 | 0.006% | -1.38 | -0.33 | 4.5E-02 | Orange |
| WP_041940149.1 | FRAAL0914 | 3-amino-5-hydroxybenzoic acid synthase | 42018 | 5.48 | 17 | 119 | Frankia-4 | F201367.dat | 548 | 46 | 13 | 13 | 16 | 17 | 11 | 12 | 16 | 14 | 0.059% | 8 | 10 | 9 | 9 | 13 | 0.039% | -1.39 | -0.33 | 9.3E-03 | Orange |
| WP_041938984.1 | FRAAL1749 | dehydrogenase | 56045 | 6.03 | 14 | 76 | Frankia-1 | F201364.dat | 495 | 22 | 10 | 10 | 11 | 11 | 8 | 7 | 8 | 11 | 0.029% | 7 | 4 | 8 | 5 | 7 | 0.019% | -1.39 | -0.33 | 1.8E-02 | Orange |
| WP_011603316.1 | FRAAL2151 | hypothetical protein | 63246 | 6.92 | 6 | 33 | Frankia-1 | F201364.dat | 213 | 12 | 5 | 5 | 5 | 5 | 4 | 3 | 4 | 4 | 0.011% | 3 | 3 | 1 | 2 | 4 | 0.007% | -1.39 | -0.33 | 2.5E-02 | Orange |
| WP_011603100.1 | FRAAL1927 | GTPase Obg | 55547 | 5.34 | 10 | 62 | Frankia-5 | F201368.dat | 376 | 25 | 9 | 9 | 10 | 8 | 7 | 6 | 6 | 10 | 0.024% | 5 | 5 | 3 | 6 | 6 | 0.015% | -1.40 | -0.34 | 1.6E-02 | Orange |
| WP_041938884.1 | FRAAL1257 | DNA polymerase III subunit beta | 41360 | 5.11 | 5 | 26 | Frankia-1 | F201364.dat | 308 | 21 | 6 | 5 | 5 | 5 | 3 | 2 | 3 | 3 | 0.014% | 2 | 2 | 2 | 2 | 2 | 0.008% | -1.40 | -0.34 | 3.8E-02 | Orange |
| WP_041940472.1 | FRAAL2870 | Pup deamidase/depupylase | 55544 | 5.19 | 31 | 235 | Frankia-1 | F201364.dat | 1184 | 58 | 23 | 23 | 33 | 33 | 26 | 24 | 30 | 25 | 0.089% | 19 | 18 | 25 | 18 | 17 | 0.059% | -1.40 | -0.34 | 3.0E-03 | Orange |
| WP_011606048.1 | FRAAL4934 | acetyl-CoA acetyltransferase | 41435 | 5.09 | 13 | 115 | Frankia-3 | F201366.dat | 649 | 47 | 11 | 11 | 16 | 12 | 13 | 16 | 14 | 13 | 0.059% | 8 | 9 | 13 | 6 | 11 | 0.038% | -1.40 | -0.34 | 8.1E-03 | Orange |
| WP_011603448.1 | FRAAL2283 | alpha.alpha-trehalose-phosphate synthase | 55655 | 5.00 | 12 | 132 | Frankia-5 | F201368.dat | 468 | 22 | 11 | 11 | 16 | 15 | 16 | 15 | 16 | 16 | 0.050% | 8 | 12 | 12 | 9 | 13 | 0.033% | -1.41 | -0.34 | 7.9E-04 | Orange |
| WP_011602314.1 | FRAAL1101 | preprotein translocase subunit SecY | 46895 | 9.97 | 4 | 31 | Frankia-1 | F201364.dat | 169 | 8 | 4 | 4 | 4 | 4 | 3 | 5 | 4 | 3 | 0.014% | 4 | 2 | 3 | 2 | 1 | 0.009% | -1.41 | -0.34 | 2.9E-02 | Orange |
| WP_011607245.1 | FRAAL6195 | hypothetical protein | 51818 | 5.37 | 13 | 89 | Frankia-4 | F201367.dat | 497 | 30 | 10 | 10 | 11 | 11 | 10 | 9 | 11 | 12 | 0.037% | 8 | 8 | 6 | 7 | 7 | 0.023% | -1.41 | -0.35 | 3.3E-04 | Orange |
| WP_011602471.1 | FRAAL1271 | phosphomannomutase | 47599 | 5.16 | 14 | 118 | Frankia-1 | F201364.dat | 632 | 26 | 11 | 11 | 18 | 18 | 14 | 12 | 12 | 14 | 0.052% | 9 | 9 | 11 | 9 | 10 | 0.034% | -1.42 | -0.35 | 2.7E-03 | Orange |
| WP_011605125.1 | FRAAL3995 | hydrolase | 34113 | 5.19 | 3 | 19 | Frankia-5 | F201368.dat | 125 | 12 | 3 | 3 | 3 | 1 | 3 | 2 | 3 | 3 | 0.013% | 2 | 1 | 2 | 1 | 1 | 0.007% | -1.42 | -0.35 | 4.5E-02 | Orange |
| WP_041940758.1 | FRAAL4421 | F420-dependent oxidoreductase | 30893 | 6.07 | 7 | 53 | Frankia-1 | F201364.dat | 324 | 27 | 5 | 5 | 6 | 6 | 5 | 8 | 9 | 4 | 0.037% | 4 | 5 | 4 | 5 | 3 | 0.023% | -1.42 | -0.35 | 2.9E-02 | Orange |
| WP_011606600.1 | FRAAL5516 | ATPase | 47780 | 5.29 | 31 | 601 | Frankia-2 | F201365.dat | 1902 | 80 | 28 | 28 | 72 | 72 | 72 | 71 | 69 | 70 | 0.264% | 53 | 56 | 44 | 47 | 47 | 0.174% | -1.42 | -0.35 | 6.8E-06 | Orange |
| WP_041941053.1 | FRAAL6153 | pyridine nucleotide-disulfide oxidoreductase | 50848 | 5.92 | 36 | 638 | Frankia-1 | F201364.dat | 1905 | 73 | 32 | 32 | 77 | 77 | 74 | 80 | 71 | 74 | 0.264% | 54 | 57 | 53 | 52 | 46 | 0.174% | -1.43 | -0.36 | 5.6E-06 | Orange |
| WP_011604636.1 | FRAAL3493 | diterpenoid dioxygenase | 41301 | 5.28 | 5 | 24 | Frankia-5 | F201368.dat | 159 | 16 | 4 | 4 | 4 | 3 | 2 | 3 | 3 | 4 | 0.013% | 2 | 2 | 3 | 1 | 1 | 0.007% | -1.43 | -0.36 | 2.2E-02 | Orange |
| WP_011603273.1 | FRAAL2108 | sulfate adenylyltransferase subunit 2 | 34496 | 5.35 | 4 | 24 | Frankia-5 | F201368.dat | 113 | 13 | 3 | 3 | 4 | 2 | 2 | 3 | 4 | 4 | 0.016% | 1 | 2 | 3 | 1 | 2 | 0.009% | -1.43 | -0.36 | 3.7E-02 | Orange |
| WP_011603462.1 | FRAAL2298 | bifunctional protein FolD | 27937 | 5.52 | 9 | 105 | Frankia-3 | F201366.dat | 358 | 30 | 7 | 7 | 13 | 12 | 11 | 13 | 15 | 12 | 0.080% | 7 | 10 | 9 | 7 | 9 | 0.051% | -1.45 | -0.37 | 8.4E-04 | Orange |
| WP_011607814.1 | FRAAL6779 | RND transporter | 93844 | 6.90 | 13 | 110 | Frankia-2 | F201365.dat | 643 | 19 | 11 | 11 | 15 | 12 | 15 | 15 | 12 | 12 | 0.025% | 12 | 10 | 10 | 7 | 5 | 0.016% | -1.45 | -0.37 | 7.9E-03 | Orange |
| WP_011604375.1 | FRAAL3226 | branched-chain alpha-keto acid dehydrogenase subunit E2 | 47405 | 6.46 | 14 | 86 | Frankia-3 | F201366.dat | 554 | 34 | 11 | 11 | 13 | 10 | 9 | 13 | 11 | 9 | 0.039% | 10 | 6 | 6 | 7 | 5 | 0.024% | -1.46 | -0.38 | 6.7E-03 | Orange |
| WP_011606340.1 | FRAAL5249 | shikimate dehydrogenase | 28889 | 5.62 | 3 | 27 | Frankia-5 | F201368.dat | 153 | 16 | 3 | 3 | 3 | 3 | 3 | 3 | 5 | 3 | 0.021% | 3 | 1 | 2 | 2 | 2 | 0.012% | -1.47 | -0.38 | 1.4E-02 | Orange |
| WP_011601346.1 | FRAAL0078 | aminotransferase AlaT | 43853 | 5.20 | 14 | 69 | Frankia-3 | F201366.dat | 383 | 32 | 9 | 9 | 10 | 8 | 6 | 10 | 8 | 10 | 0.034% | 5 | 5 | 7 | 6 | 4 | 0.021% | -1.47 | -0.38 | 5.3E-03 | Orange |
| WP_011607838.1 | FRAAL6804 | protein nifZ | 20984 | 5.22 | 4 | 52 | Frankia-5 | F201368.dat | 227 | 29 | 4 | 4 | 6 | 6 | 6 | 7 | 7 | 6 | 0.054% | 4 | 3 | 4 | 5 | 4 | 0.032% | -1.48 | -0.39 | 4.5E-04 | Orange |
| WP_050997228.1 | FRAAL5337 | (2Fe-2S)-binding protein | 40496 | 5.34 | 11 | 67 | Frankia-1 | F201364.dat | 311 | 29 | 8 | 8 | 10 | 10 | 7 | 8 | 8 | 8 | 0.036% | 4 | 7 | 5 | 5 | 5 | 0.022% | -1.48 | -0.39 | 1.3E-03 | Orange |
| WP_041938891.1 | FRAAL1303 | 30S ribosomal protein S30 | 28140 | 5.77 | 8 | 87 | Frankia-5 | F201368.dat | 462 | 38 | 8 | 8 | 12 | 9 | 10 | 10 | 12 | 12 | 0.067% | 6 | 5 | 8 | 7 | 8 | 0.041% | -1.49 | -0.40 | 9.5E-04 | Orange |
| WP_011606757.1 | FRAAL5683 | DNA-binding protein | 64943 | 8.87 | 15 | 92 | Frankia-4 | F201367.dat | 433 | 21 | 11 | 11 | 14 | 9 | 12 | 11 | 14 | 10 | 0.031% | 8 | 4 | 9 | 6 | 9 | 0.019% | -1.49 | -0.40 | 7.5E-03 | Orange |
| WP_011602680.1 | FRAAL1491 | XRE family transcriptional regulator | 54549 | 7.03 | 18 | 142 | Frankia-3 | F201366.dat | 556 | 34 | 14 | 14 | 20 | 17 | 15 | 20 | 17 | 17 | 0.056% | 13 | 11 | 11 | 12 | 9 | 0.035% | -1.49 | -0.40 | 2.1E-04 | Orange |
| WP_011607846.1 | FRAAL6812 | nitrogenase molybdenum-iron protein alpha chain | 54026 | 5.84 | 50 | 1168 | Frankia-3 | F201366.dat | 2892 | 91 | 46 | 46 | 150 | 140 | 144 | 150 | 132 | 135 | 0.463% | 89 | 107 | 88 | 93 | 90 | 0.291% | -1.50 | -0.40 | 4.7E-06 | Orange |
| WP_011603281.1 | FRAAL2116 | malto-oligosyltrehalose trehalohydrolase | 64144 | 5.37 | 20 | 175 | Frankia-3 | F201366.dat | 791 | 39 | 16 | 16 | 22 | 22 | 19 | 22 | 21 | 22 | 0.059% | 11 | 14 | 16 | 14 | 14 | 0.036% | -1.50 | -0.41 | 3.5E-05 | Orange |
| WP_011601337.1 | FRAAL0069 | dehydrogenase | 54315 | 6.68 | 5 | 7 | Frankia-7 | F201370.dat | 80 | 9 | 3 | 3 | 3 | 0 | 0 | 0 | 0 | 0 | 0.000% | 1 | 3 | 3 | 0 | 0 | 0.004% | 2.40 | 0.88 | 5.0E-02 | Green |
| WP_041938818.1 | FRAAL0948 | fatty acid-binding protein | 20584 | 5.21 | 2 | 6 | Frankia-8 | F201371.dat | 90 | 14 | 2 | 2 | 3 | 0 | 0 | 0 | 0 | 0 | 0.000% | 0 | 2 | 3 | 1 | 0 | 0.010% | 2.20 | 0.79 | 5.6E-02 | Green |
| WP_011603704.1 | FRAAL2547 | non-ribosomal peptide synthetase | 179619 | 4.91 | 5 | 6 | Frankia-8 | F201371.dat | 167 | 4 | 4 | 1 | 4 | 0 | 0 | 0 | 0 | 0 | 0.000% | 0 | 0 | 4 | 0 | 2 | 0.001% | 2.20 | 0.79 | 1.0E-01 | Green |
| WP_011607517.1 | FRAAL6476 | hypothetical protein | 22773 | 7.94 | 4 | 6 | Frankia-8 | F201371.dat | 138 | 16 | 4 | 4 | 4 | 0 | 0 | 0 | 0 | 0 | 0.000% | 0 | 0 | 4 | 0 | 2 | 0.009% | 2.20 | 0.79 | 1.0E-01 | Green |
| WP_011606240.1 | FRAAL5133 | hypothetical protein | 17797 | 11.09 | 5 | 9 | Frankia-8 | F201371.dat | 78 | 20 | 3 | 3 | 3 | 1 | 0 | 0 | 0 | 0 | 0.002% | 0 | 0 | 3 | 3 | 2 | 0.015% | 2.17 | 0.77 | 5.0E-02 | Green |
| WP_011602991.1 | FRAAL1811 | ATP/GTP-binding protein | 19448 | 4.53 | 3 | 5 | Frankia-7 | F201370.dat | 105 | 13 | 2 | 2 | 2 | 0 | 0 | 0 | 0 | 0 | 0.000% | 2 | 2 | 1 | 0 | 0 | 0.009% | 2.00 | 0.69 | 5.5E-02 | Green |
| WP_011601890.1 | FRAAL0642 | hypothetical protein | 14469 | 6.31 | 2 | 5 | Frankia-10 | F201373.dat | 135 | 24 | 2 | 2 | 2 | 0 | 0 | 0 | 0 | 0 | 0.000% | 0 | 0 | 2 | 1 | 2 | 0.012% | 2.00 | 0.69 | 5.5E-02 | Green |
| WP_011604988.1 | FRAAL3853 | TetR family transcriptional regulator | 21030 | 8.34 | 2 | 5 | Frankia-10 | F201373.dat | 115 | 11 | 2 | 2 | 2 | 0 | 0 | 0 | 0 | 0 | 0.000% | 0 | 0 | 2 | 1 | 2 | 0.008% | 2.00 | 0.69 | 5.5E-02 | Green |
| WP_041939495.1 | FRAAL4385 | short-chain dehydrogenase | 25112 | 5.48 | 3 | 14 | Frankia-8 | F201371.dat | 127 | 11 | 3 | 3 | 4 | 0 | 0 | 0 | 0 | 3 | 0.004% | 0 | 2 | 4 | 2 | 3 | 0.015% | 2.00 | 0.69 | 5.6E-02 | Green |
| WP_011607359.1 | FRAAL6311 | MerR family transcriptional regulator | 14444 | 5.44 | 4 | 8 | Frankia-10 | F201373.dat | 153 | 28 | 3 | 3 | 3 | 0 | 0 | 0 | 1 | 0 | 0.002% | 0 | 0 | 2 | 2 | 3 | 0.016% | 2.00 | 0.69 | 5.9E-02 | Green |
| WP_011602338.1 | FRAAL1131 | MarR family transcriptional regulator | 21723 | 5.06 | 2 | 5 | Frankia-8 | F201371.dat | 56 | 10 | 2 | 2 | 3 | 0 | 0 | 0 | 0 | 0 | 0.000% | 0 | 1 | 3 | 1 | 0 | 0.008% | 2.00 | 0.69 | 7.8E-02 | Green |
| WP_050997418.1 | FRAAL4713 | enoyl-CoA hydratase | 30311 | 5.62 | 3 | 5 | Frankia-8 | F201371.dat | 177 | 16 | 3 | 3 | 3 | 0 | 0 | 0 | 0 | 0 | 0.000% | 0 | 2 | 3 | 0 | 0 | 0.006% | 2.00 | 0.69 | 9.9E-02 | Green |
| WP_011604852.1 | FRAAL3713 | phosphohydrolase | 22275 | 5.96 | 3 | 5 | Frankia-8 | F201371.dat | 114 | 20 | 3 | 3 | 3 | 0 | 0 | 0 | 0 | 0 | 0.000% | 0 | 0 | 3 | 0 | 2 | 0.008% | 2.00 | 0.69 | 9.9E-02 | Green |
| WP_011607378.1 | FRAAL6330 | helicase | 233812 | 5.66 | 17 | 42 | Frankia-8 | F201371.dat | 541 | 8 | 13 | 13 | 14 | 5 | 2 | 3 | 1 | 2 | 0.002% | 2 | 0 | 14 | 8 | 5 | 0.004% | 1.89 | 0.64 | 1.2E-01 | Green |
| WP_011606282.1 | FRAAL5180 | aldo/keto reductase | 33942 | 5.39 | 11 | 44 | Frankia-8 | F201371.dat | 450 | 36 | 10 | 10 | 12 | 2 | 4 | 4 | 1 | 3 | 0.015% | 2 | 5 | 12 | 5 | 6 | 0.030% | 1.84 | 0.61 | 5.2E-02 | Green |
| WP_041939477.1 | FRAAL4277 | hypothetical protein | 16907 | 9.44 | 3 | 7 | Frankia-7 | F201370.dat | 119 | 26 | 2 | 2 | 2 | 0 | 0 | 0 | 0 | 1 | 0.002% | 0 | 2 | 2 | 2 | 0 | 0.012% | 1.83 | 0.61 | 6.5E-02 | Green |
| WP_011607480.1 | FRAAL6435 | GlcNAc-PI de-N-acetylase | 26702 | 5.29 | 3 | 7 | Frankia-8 | F201371.dat | 108 | 15 | 3 | 3 | 3 | 0 | 0 | 1 | 0 | 0 | 0.001% | 0 | 1 | 3 | 1 | 1 | 0.008% | 1.83 | 0.61 | 6.5E-02 | Green |
| WP_011601987.1 | FRAAL0746 | hypothetical protein | 39396 | 9.24 | 5 | 7 | Frankia-7 | F201370.dat | 81 | 9 | 3 | 3 | 3 | 1 | 0 | 0 | 0 | 0 | 0.001% | 0 | 3 | 2 | 0 | 1 | 0.005% | 1.83 | 0.61 | 8.7E-02 | Green |
| WP_041939888.1 | FRAAL6394 | molybdenum cofactor biosynthesis protein | 18252 | 5.14 | 3 | 7 | Frankia-8 | F201371.dat | 130 | 35 | 3 | 3 | 3 | 0 | 0 | 0 | 1 | 0 | 0.002% | 0 | 0 | 3 | 1 | 2 | 0.011% | 1.83 | 0.61 | 8.7E-02 | Green |
| WP_050997055.1 | FRAAL2130 | HIT family hydrolase | 21242 | 5.19 | 2 | 4 | Frankia-9 | F201372.dat | 46 | 5 | 1 | 1 | 1 | 0 | 0 | 0 | 0 | 0 | 0.000% | 1 | 0 | 1 | 1 | 1 | 0.006% | 1.80 | 0.59 | 6.3E-02 | Green |
| WP_041939821.1 | FRAAL6067 | recombinase RecB | 31152 | 8.61 | 2 | 4 | Frankia-8 | F201371.dat | 40 | 4 | 1 | 1 | 1 | 0 | 0 | 0 | 0 | 0 | 0.000% | 1 | 0 | 1 | 1 | 1 | 0.004% | 1.80 | 0.59 | 6.3E-02 | Green |
| WP_011606006.1 | FRAAL4892 | type 12 methyltransferase | 24580 | 5.33 | 2 | 4 | Frankia-8 | F201371.dat | 141 | 10 | 2 | 2 | 2 | 0 | 0 | 0 | 0 | 0 | 0.000% | 0 | 1 | 2 | 1 | 0 | 0.005% | 1.80 | 0.59 | 7.4E-02 | Green |
| WP_041939672.1 | FRAAL5297 | hypothetical protein | 32127 | 9.78 | 2 | 4 | Frankia-8 | F201371.dat | 118 | 8 | 2 | 2 | 2 | 0 | 0 | 0 | 0 | 0 | 0.000% | 0 | 0 | 2 | 1 | 1 | 0.004% | 1.80 | 0.59 | 7.4E-02 | Green |
| WP_011607873.1 | FRAAL6838 | transporter | 5045 | 11.62 | 2 | 4 | Frankia-9 | F201372.dat | 98 | 58 | 2 | 2 | 2 | 0 | 0 | 0 | 0 | 0 | 0.000% | 0 | 0 | 1 | 2 | 1 | 0.027% | 1.80 | 0.59 | 7.4E-02 | Green |
| WP_041939262.1 | FRAAL2995 | branched-chain amino acid ABC transporter substrate-binding protein | 42827 | 5.03 | 2 | 4 | Frankia-8 | F201371.dat | 86 | 5 | 2 | 1 | 1 | 0 | 0 | 0 | 0 | 0 | 0.000% | 0 | 0 | 1 | 1 | 2 | 0.003% | 1.80 | 0.59 | 7.4E-02 | Green |
| WP_041939063.1 | FRAAL2150 | XRE family transcriptional regulator | 54651 | 9.37 | 2 | 4 | Frankia-7 | F201370.dat | 82 | 3 | 2 | 2 | 2 | 0 | 0 | 0 | 0 | 0 | 0.000% | 0 | 2 | 0 | 1 | 1 | 0.002% | 1.80 | 0.59 | 7.4E-02 | Green |
| WP_011603751.1 | FRAAL2597 | regulatory protein MarR | 22783 | 6.98 | 2 | 4 | Frankia-8 | F201371.dat | 80 | 12 | 2 | 2 | 2 | 0 | 0 | 0 | 0 | 0 | 0.000% | 0 | 1 | 2 | 1 | 0 | 0.006% | 1.80 | 0.59 | 7.4E-02 | Green |
| WP_041940903.1 | FRAAL5299 | two-component system response regulator | 26671 | 8.14 | 2 | 4 | Frankia-8 | F201371.dat | 61 | 7 | 2 | 2 | 2 | 0 | 0 | 0 | 0 | 0 | 0.000% | 0 | 0 | 2 | 1 | 1 | 0.005% | 1.80 | 0.59 | 7.4E-02 | Green |
| WP_041939149.1 | FRAAL2542 | peptide synthetase | 56656 | 5.51 | 2 | 4 | Frankia-7 | F201370.dat | 58 | 7 | 2 | 2 | 2 | 0 | 0 | 0 | 0 | 0 | 0.000% | 0 | 2 | 1 | 0 | 1 | 0.002% | 1.80 | 0.59 | 7.4E-02 | Green |
| WP_041940718.1 | FRAAL4223 | hypothetical protein | 25389 | 5.24 | 3 | 4 | Frankia-8 | F201371.dat | 68 | 11 | 2 | 2 | 2 | 0 | 0 | 0 | 0 | 0 | 0.000% | 0 | 0 | 2 | 0 | 2 | 0.005% | 1.80 | 0.59 | 1.1E-01 | Green |
| WP_011602720.1 | FRAAL1530 | phosphoglycerate mutase | 21559 | 5.87 | 3 | 4 | Frankia-10 | F201373.dat | 110 | 19 | 3 | 3 | 3 | 0 | 0 | 0 | 0 | 0 | 0.000% | 0 | 0 | 0 | 1 | 3 | 0.006% | 1.80 | 0.59 | 1.3E-01 | Green |
| WP_011602503.1 | FRAAL1305 | acetyltransferase | 20504 | 5.18 | 5 | 12 | Frankia-10 | F201373.dat | 227 | 25 | 4 | 4 | 4 | 0 | 0 | 1 | 1 | 1 | 0.005% | 0 | 2 | 2 | 1 | 4 | 0.015% | 1.75 | 0.56 | 7.2E-02 | Green |
| WP_041939013.1 | FRAAL1891 | FAD-dependent oxidoreductase | 42337 | 4.97 | 4 | 12 | Frankia-8 | F201371.dat | 179 | 16 | 4 | 4 | 4 | 2 | 0 | 0 | 1 | 0 | 0.003% | 0 | 1 | 4 | 1 | 3 | 0.007% | 1.75 | 0.56 | 9.5E-02 | Green |
| WP_011607365.1 | FRAAL6317 | ATP/GTP-binding protein | 96689 | 5.56 | 8 | 20 | Frankia-8 | F201371.dat | 218 | 9 | 7 | 7 | 7 | 2 | 1 | 1 | 1 | 1 | 0.002% | 1 | 1 | 7 | 2 | 3 | 0.005% | 1.73 | 0.55 | 1.0E-01 | Green |
| WP_011603178.1 | FRAAL2006 | HrcA family transcriptional regulator | 36821 | 5.40 | 3 | 9 | Frankia-7 | F201370.dat | 121 | 12 | 3 | 3 | 3 | 0 | 0 | 0 | 1 | 1 | 0.002% | 0 | 3 | 1 | 2 | 1 | 0.006% | 1.71 | 0.54 | 6.9E-02 | Green |
| WP_011603610.1 | FRAAL2453 | TetR family transcriptional regulator | 21607 | 6.15 | 4 | 9 | Frankia-7 | F201370.dat | 120 | 20 | 3 | 3 | 3 | 0 | 0 | 2 | 0 | 0 | 0.003% | 0 | 3 | 2 | 2 | 0 | 0.011% | 1.71 | 0.54 | 1.0E-01 | Green |
| WP_035921480.1 | FRAAL6400 | hypothetical protein | 9212 | 10.34 | 4 | 17 | Frankia-10 | F201373.dat | 148 | 37 | 3 | 3 | 4 | 0 | 2 | 0 | 0 | 3 | 0.019% | 2 | 2 | 3 | 1 | 4 | 0.044% | 1.70 | 0.53 | 6.2E-02 | Green |
| WP_041940992.1 | FRAAL5775 | plasmid partitioning protein | 44236 | 8.58 | 5 | 17 | Frankia-7 | F201370.dat | 129 | 8 | 3 | 3 | 3 | 0 | 3 | 0 | 2 | 0 | 0.004% | 0 | 3 | 3 | 3 | 3 | 0.009% | 1.70 | 0.53 | 7.3E-02 | Green |
| WP_011604201.1 | FRAAL3052 | short-chain dehydrogenase | 27658 | 5.56 | 9 | 41 | Frankia-10 | F201373.dat | 444 | 49 | 8 | 8 | 9 | 2 | 6 | 0 | 2 | 4 | 0.018% | 3 | 4 | 6 | 5 | 9 | 0.033% | 1.68 | 0.52 | 5.5E-02 | Green |
| WP_041940778.1 | FRAAL4567 | XRE family transcriptional regulator | 20493 | 11.52 | 2 | 6 | Frankia-10 | F201373.dat | 100 | 20 | 2 | 2 | 2 | 0 | 1 | 0 | 0 | 0 | 0.002% | 1 | 0 | 1 | 1 | 2 | 0.008% | 1.67 | 0.51 | 6.3E-02 | Green |
| WP_050997022.1 | FRAAL1390 | C4-dicarboxylate transporter | 52733 | 6.14 | 2 | 6 | Frankia-7 | F201370.dat | 62 | 4 | 2 | 2 | 2 | 1 | 0 | 0 | 0 | 0 | 0.001% | 1 | 2 | 1 | 0 | 1 | 0.003% | 1.67 | 0.51 | 6.3E-02 | Green |
| WP_011604456.1 | FRAAL3309 | hypothetical protein | 52375 | 6.13 | 4 | 6 | Frankia-9 | F201372.dat | 93 | 5 | 2 | 2 | 2 | 1 | 0 | 0 | 0 | 0 | 0.001% | 0 | 2 | 1 | 2 | 0 | 0.003% | 1.67 | 0.51 | 9.4E-02 | Green |
| WP_041938697.1 | FRAAL0457 | CRISPR-associated protein Cas7 | 27683 | 11.13 | 2 | 6 | Frankia-10 | F201373.dat | 107 | 10 | 2 | 2 | 2 | 0 | 0 | 0 | 0 | 1 | 0.001% | 0 | 1 | 2 | 0 | 2 | 0.006% | 1.67 | 0.51 | 9.4E-02 | Green |
| WP_011604061.1 | FRAAL2915 | dehydrogenase | 27100 | 5.96 | 3 | 6 | Frankia-8 | F201371.dat | 201 | 15 | 3 | 3 | 3 | 0 | 0 | 1 | 0 | 0 | 0.001% | 0 | 0 | 3 | 0 | 2 | 0.006% | 1.67 | 0.51 | 1.5E-01 | Green |
| WP_011606599.1 | FRAAL5515 | hypothetical protein | 41882 | 5.10 | 3 | 6 | Frankia-7 | F201370.dat | 99 | 9 | 3 | 3 | 3 | 1 | 0 | 0 | 0 | 0 | 0.001% | 2 | 3 | 0 | 0 | 0 | 0.004% | 1.67 | 0.51 | 1.5E-01 | Green |
| WP_041940083.1 | FRAAL0508 | epimerase | 34801 | 4.99 | 6 | 19 | Frankia-8 | F201371.dat | 298 | 32 | 6 | 6 | 6 | 1 | 2 | 1 | 1 | 1 | 0.006% | 2 | 2 | 6 | 1 | 2 | 0.013% | 1.64 | 0.49 | 8.6E-02 | Green |
| WP_041939919.1 | FRAAL6558 | endonuclease III | 26472 | 9.39 | 3 | 11 | Frankia-10 | F201373.dat | 123 | 17 | 3 | 3 | 3 | 0 | 1 | 0 | 0 | 2 | 0.004% | 1 | 1 | 2 | 1 | 3 | 0.010% | 1.63 | 0.49 | 5.8E-02 | Green |
| WP_041940616.1 | FRAAL3709 | F420-dependent oxidoreductase | 30702 | 5.20 | 5 | 24 | Frankia-8 | F201371.dat | 184 | 20 | 5 | 5 | 5 | 1 | 2 | 1 | 2 | 2 | 0.009% | 0 | 4 | 5 | 2 | 5 | 0.018% | 1.62 | 0.48 | 7.8E-02 | Green |
| WP_011603073.1 | FRAAL1896 | hypothetical protein | 95329 | 9.05 | 6 | 24 | Frankia-8 | F201371.dat | 237 | 10 | 6 | 6 | 7 | 2 | 1 | 3 | 2 | 0 | 0.003% | 2 | 1 | 7 | 3 | 3 | 0.006% | 1.62 | 0.48 | 9.9E-02 | Green |
| WP_009739504.1 | FRAAL2273 | hypothetical protein | 17629 | 4.46 | 5 | 29 | Frankia-8 | F201371.dat | 185 | 29 | 4 | 4 | 7 | 2 | 1 | 3 | 3 | 1 | 0.020% | 3 | 2 | 7 | 4 | 3 | 0.036% | 1.60 | 0.47 | 5.0E-02 | Green |
| WP_011601857.1 | FRAAL0608 | hypothetical protein | 15868 | 11.00 | 2 | 3 | Frankia-8 | F201371.dat | 60 | 11 | 1 | 1 | 1 | 0 | 0 | 0 | 0 | 0 | 0.000% | 1 | 1 | 1 | 0 | 0 | 0.006% | 1.60 | 0.47 | 1.2E-01 | Green |
| WP_041940925.1 | FRAAL5379 | ABC transporter | 25043 | 9.02 | 2 | 3 | Frankia-9 | F201372.dat | 54 | 10 | 1 | 1 | 1 | 0 | 0 | 0 | 0 | 0 | 0.000% | 0 | 1 | 1 | 1 | 0 | 0.004% | 1.60 | 0.47 | 1.2E-01 | Green |
| WP_011604705.1 | FRAAL3564 | UDP pyrophosphate synthase | 28935 | 6.51 | 2 | 3 | Frankia-9 | F201372.dat | 39 | 6 | 1 | 1 | 1 | 0 | 0 | 0 | 0 | 0 | 0.000% | 1 | 0 | 1 | 1 | 0 | 0.003% | 1.60 | 0.47 | 1.2E-01 | Green |
| WP_011604125.1 | FRAAL2979 | multidrug ABC transporter ATP-binding protein | 34504 | 5.94 | 3 | 3 | Frankia-7 | F201370.dat | 172 | 12 | 2 | 2 | 2 | 0 | 0 | 0 | 0 | 0 | 0.000% | 0 | 2 | 0 | 1 | 0 | 0.003% | 1.60 | 0.47 | 1.4E-01 | Green |
| WP_041939151.1 | FRAAL2545 | non-ribosomal peptide synthetase | 277990 | 5.13 | 3 | 3 | Frankia-8 | F201371.dat | 142 | 2 | 4 | 1 | 1 | 0 | 0 | 0 | 0 | 0 | 0.000% | 0 | 0 | 1 | 2 | 0 | 0.000% | 1.60 | 0.47 | 1.4E-01 | Green |
| WP_041941146.1 | FRAAL6723 | anti-sigma factor | 19696 | 4.89 | 2 | 3 | Frankia-8 | F201371.dat | 105 | 14 | 2 | 2 | 2 | 0 | 0 | 0 | 0 | 0 | 0.000% | 0 | 1 | 2 | 0 | 0 | 0.005% | 1.60 | 0.47 | 1.4E-01 | Green |
| WP_011605628.1 | FRAAL4509 | hypothetical protein | 28907 | 6.20 | 2 | 3 | Frankia-8 | F201371.dat | 91 | 13 | 2 | 2 | 2 | 0 | 0 | 0 | 0 | 0 | 0.000% | 1 | 0 | 2 | 0 | 0 | 0.003% | 1.60 | 0.47 | 1.4E-01 | Green |
| WP_041939116.1 | FRAAL2387 | hypothetical protein | 8132 | 5.11 | 2 | 3 | Frankia-9 | F201372.dat | 90 | 38 | 2 | 2 | 2 | 0 | 0 | 0 | 0 | 0 | 0.000% | 1 | 0 | 0 | 2 | 0 | 0.012% | 1.60 | 0.47 | 1.4E-01 | Green |
| WP_011607257.1 | FRAAL6207 | hypothetical protein | 21882 | 5.83 | 2 | 3 | Frankia-8 | F201371.dat | 90 | 9 | 2 | 2 | 2 | 0 | 0 | 0 | 0 | 0 | 0.000% | 0 | 0 | 2 | 1 | 0 | 0.005% | 1.60 | 0.47 | 1.4E-01 | Green |
| WP_011601807.1 | FRAAL0554 | MarR family transcriptional regulator | 17235 | 11.02 | 2 | 3 | Frankia-8 | F201371.dat | 89 | 15 | 2 | 2 | 2 | 0 | 0 | 0 | 0 | 0 | 0.000% | 0 | 0 | 2 | 1 | 0 | 0.006% | 1.60 | 0.47 | 1.4E-01 | Green |
| WP_011602649.1 | FRAAL1459 | carbon-monoxide dehydrogenase | 83916 | 5.48 | 2 | 3 | Frankia-8 | F201371.dat | 77 | 5 | 2 | 2 | 2 | 0 | 0 | 0 | 0 | 0 | 0.000% | 0 | 0 | 2 | 0 | 1 | 0.001% | 1.60 | 0.47 | 1.4E-01 | Green |
| WP_041939964.1 | FRAAL6721 | NADP transhydrogenase subunit alpha | 34409 | 7.90 | 2 | 3 | Frankia-8 | F201371.dat | 71 | 7 | 2 | 2 | 2 | 0 | 0 | 0 | 0 | 0 | 0.000% | 0 | 0 | 2 | 0 | 1 | 0.003% | 1.60 | 0.47 | 1.4E-01 | Green |
| WP_050997323.1 | FRAAL1244 | serine/threonine protein kinase | 87621 | 6.04 | 2 | 3 | Frankia-9 | F201372.dat | 70 | 2 | 2 | 2 | 2 | 0 | 0 | 0 | 0 | 0 | 0.000% | 0 | 0 | 1 | 2 | 0 | 0.001% | 1.60 | 0.47 | 1.4E-01 | Green |
| WP_011604834.1 | FRAAL3695 | deaminase | 22901 | 4.73 | 2 | 3 | Frankia-9 | F201372.dat | 63 | 21 | 2 | 2 | 2 | 0 | 0 | 0 | 0 | 0 | 0.000% | 0 | 1 | 0 | 2 | 0 | 0.004% | 1.60 | 0.47 | 1.4E-01 | Green |
| WP_041938875.1 | FRAAL1201 | hypothetical protein | 14592 | 11.58 | 3 | 3 | Frankia-8 | F201371.dat | 131 | 26 | 3 | 3 | 3 | 0 | 0 | 0 | 0 | 0 | 0.000% | 0 | 0 | 3 | 0 | 0 | 0.007% | 1.60 | 0.47 | 2.0E-01 | Green |
| WP_011605384.1 | FRAAL4258 | luciferase | 45720 | 5.89 | 3 | 3 | Frankia-8 | F201371.dat | 79 | 8 | 3 | 3 | 3 | 0 | 0 | 0 | 0 | 0 | 0.000% | 0 | 0 | 3 | 0 | 0 | 0.002% | 1.60 | 0.47 | 2.0E-01 | Green |
| WP_041940225.1 | FRAAL1434 | aromatic compound degradation protein PaaI | 19431 | 5.36 | 2 | 3 | Frankia-10 | F201373.dat | 66 | 21 | 2 | 2 | 3 | 0 | 0 | 0 | 0 | 0 | 0.000% | 0 | 0 | 0 | 0 | 3 | 0.005% | 1.60 | 0.47 | 2.0E-01 | Green |
| WP_011604237.1 | FRAAL3088 | transcriptional regulator | 22942 | 7.03 | 2 | 8 | Frankia-7 | F201370.dat | 100 | 22 | 2 | 2 | 2 | 1 | 0 | 1 | 0 | 0 | 0.003% | 1 | 2 | 1 | 1 | 1 | 0.009% | 1.57 | 0.45 | 6.3E-02 | Green |
| WP_011605196.1 | FRAAL4066 | hypothetical protein | 34578 | 5.47 | 2 | 8 | Frankia-8 | F201371.dat | 63 | 13 | 2 | 2 | 2 | 1 | 0 | 1 | 0 | 0 | 0.002% | 1 | 1 | 2 | 1 | 1 | 0.006% | 1.57 | 0.45 | 6.3E-02 | Green |
| WP_011604029.1 | FRAAL2883 | primosome assembly protein PriA | 9295 | 8.23 | 2 | 8 | Frankia-9 | F201372.dat | 64 | 16 | 2 | 2 | 2 | 0 | 0 | 1 | 1 | 0 | 0.008% | 0 | 1 | 1 | 2 | 2 | 0.022% | 1.57 | 0.45 | 7.4E-02 | Green |
| WP_011607865.1 | FRAAL6830 | chromosome partitioning protein | 38658 | 6.26 | 6 | 8 | Frankia-7 | F201370.dat | 83 | 6 | 2 | 2 | 2 | 0 | 0 | 0 | 2 | 0 | 0.002% | 2 | 2 | 1 | 1 | 0 | 0.005% | 1.57 | 0.45 | 9.1E-02 | Green |
| WP_011606396.1 | FRAAL5306 | 3-oxoacyl-ACP reductase | 25446 | 4.97 | 4 | 8 | Frankia-8 | F201371.dat | 70 | 13 | 3 | 3 | 4 | 2 | 0 | 0 | 0 | 0 | 0.003% | 0 | 0 | 4 | 1 | 1 | 0.008% | 1.57 | 0.45 | 1.8E-01 | Green |
| WP_011606610.1 | FRAAL5526 | aldo/keto reductase | 38199 | 5.54 | 16 | 123 | Frankia-8 | F201371.dat | 935 | 64 | 16 | 16 | 28 | 10 | 6 | 11 | 6 | 14 | 0.044% | 11 | 13 | 28 | 11 | 13 | 0.067% | 1.56 | 0.44 | 7.2E-02 | Green |
| WP_011601962.1 | FRAAL0720 | L-asparaginase | 34911 | 6.37 | 9 | 36 | Frankia-8 | F201371.dat | 256 | 24 | 7 | 7 | 8 | 3 | 3 | 1 | 3 | 3 | 0.013% | 4 | 2 | 8 | 3 | 6 | 0.022% | 1.56 | 0.44 | 6.0E-02 | Green |
| WP_041939093.1 | FRAAL2265 | mannose-1-phosphate guanyltransferase | 88457 | 5.02 | 3 | 13 | Frankia-10 | F201373.dat | 82 | 4 | 3 | 3 | 3 | 1 | 0 | 1 | 0 | 2 | 0.002% | 2 | 2 | 0 | 2 | 3 | 0.003% | 1.56 | 0.44 | 7.2E-02 | Green |
| WP_011603677.1 | FRAAL2521 | hypothetical protein | 25106 | 5.88 | 3 | 13 | Frankia-8 | F201371.dat | 142 | 22 | 3 | 3 | 3 | 0 | 0 | 1 | 3 | 0 | 0.006% | 1 | 2 | 3 | 1 | 2 | 0.012% | 1.56 | 0.44 | 9.3E-02 | Green |
| WP_041939911.1 | FRAAL6522 | PPOX class F420-dependent enzyme | 14624 | 4.90 | 6 | 23 | Frankia-10 | F201373.dat | 250 | 50 | 6 | 6 | 6 | 1 | 1 | 0 | 3 | 3 | 0.020% | 1 | 2 | 3 | 3 | 6 | 0.035% | 1.54 | 0.43 | 1.1E-01 | Green |
| WP_011603492.1 | FRAAL2328 | aldo/keto reductase | 34709 | 5.18 | 8 | 23 | Frankia-8 | F201371.dat | 344 | 37 | 9 | 8 | 9 | 1 | 2 | 0 | 2 | 3 | 0.008% | 1 | 1 | 9 | 2 | 2 | 0.015% | 1.54 | 0.43 | 2.0E-01 | Green |
| WP_011602397.1 | FRAAL1194 | pyrimidine-nucleoside phosphorylase | 45971 | 5.96 | 5 | 15 | Frankia-8 | F201371.dat | 142 | 11 | 3 | 3 | 3 | 1 | 1 | 1 | 1 | 1 | 0.004% | 1 | 3 | 3 | 1 | 2 | 0.007% | 1.50 | 0.41 | 5.5E-02 | Green |
| WP_041938638.1 | FRAAL0222 | hypothetical protein | 51764 | 5.05 | 6 | 20 | Frankia-10 | F201373.dat | 193 | 11 | 4 | 4 | 4 | 3 | 1 | 0 | 1 | 2 | 0.005% | 1 | 2 | 3 | 3 | 4 | 0.008% | 1.50 | 0.41 | 6.7E-02 | Green |
| WP_011606324.1 | FRAAL5231 | guanylate kinase | 19602 | 5.98 | 4 | 15 | Frankia-9 | F201372.dat | 129 | 27 | 3 | 3 | 3 | 2 | 0 | 2 | 0 | 1 | 0.009% | 1 | 2 | 3 | 3 | 1 | 0.017% | 1.50 | 0.41 | 7.6E-02 | Green |
| WP_011606205.1 | FRAAL5097 | metallophosphoesterase | 28498 | 5.52 | 2 | 5 | Frankia-7 | F201370.dat | 64 | 4 | 1 | 1 | 1 | 0 | 0 | 0 | 1 | 0 | 0.001% | 0 | 1 | 1 | 1 | 1 | 0.005% | 1.50 | 0.41 | 1.2E-01 | Green |
| WP_041939643.1 | FRAAL5124 | aminotransferase V | 39309 | 6.05 | 2 | 5 | Frankia-9 | F201372.dat | 56 | 3 | 1 | 1 | 1 | 0 | 0 | 1 | 0 | 0 | 0.001% | 0 | 1 | 1 | 1 | 1 | 0.003% | 1.50 | 0.41 | 1.2E-01 | Green |
| WP_011601378.1 | FRAAL0113 | LuxR family transcriptional regulator | 26836 | 6.60 | 4 | 5 | Frankia-9 | F201372.dat | 58 | 16 | 2 | 2 | 2 | 0 | 0 | 0 | 0 | 1 | 0.001% | 0 | 0 | 1 | 2 | 1 | 0.005% | 1.50 | 0.41 | 1.3E-01 | Green |
| WP_011605857.1 | FRAAL4742 | F420-dependent oxidoreductase | 29668 | 4.81 | 3 | 5 | Frankia-10 | F201373.dat | 97 | 10 | 2 | 2 | 2 | 0 | 1 | 0 | 0 | 0 | 0.001% | 0 | 1 | 1 | 0 | 2 | 0.005% | 1.50 | 0.41 | 1.3E-01 | Green |
| WP_009738322.1 | FRAAL3494 | MaoC family dehydratase | 17141 | 5.34 | 3 | 5 | Frankia-10 | F201373.dat | 70 | 20 | 2 | 2 | 2 | 0 | 1 | 0 | 0 | 0 | 0.002% | 0 | 0 | 1 | 1 | 2 | 0.008% | 1.50 | 0.41 | 1.3E-01 | Green |
| WP_041938629.1 | FRAAL0163 | manganese ABC transporter | 33117 | 5.21 | 2 | 5 | Frankia-7 | F201370.dat | 119 | 7 | 2 | 2 | 2 | 1 | 0 | 0 | 0 | 0 | 0.001% | 0 | 2 | 1 | 0 | 1 | 0.004% | 1.50 | 0.41 | 1.3E-01 | Green |
| WP_041939446.1 | FRAAL4097 | TetR family transcriptional regulator | 21347 | 5.51 | 2 | 5 | Frankia-10 | F201373.dat | 48 | 18 | 2 | 2 | 2 | 1 | 0 | 0 | 0 | 0 | 0.002% | 0 | 0 | 1 | 1 | 2 | 0.006% | 1.50 | 0.41 | 1.3E-01 | Green |
| WP_011602780.1 | FRAAL1590 | glyoxalase | 16136 | 4.30 | 3 | 5 | Frankia-10 | F201373.dat | 64 | 21 | 2 | 2 | 2 | 0 | 0 | 0 | 1 | 0 | 0.002% | 0 | 2 | 0 | 0 | 2 | 0.008% | 1.50 | 0.41 | 1.7E-01 | Green |
| WP_011604932.1 | FRAAL3794 | short-chain dehydrogenase | 31344 | 5.59 | 3 | 5 | Frankia-9 | F201372.dat | 53 | 9 | 2 | 2 | 2 | 0 | 0 | 0 | 1 | 0 | 0.001% | 0 | 0 | 2 | 2 | 0 | 0.004% | 1.50 | 0.41 | 1.7E-01 | Green |
| WP_011602507.1 | FRAAL1309 | hypothetical protein | 20882 | 6.22 | 2 | 5 | Frankia-10 | F201373.dat | 93 | 20 | 2 | 2 | 2 | 0 | 0 | 0 | 0 | 1 | 0.002% | 0 | 0 | 0 | 2 | 2 | 0.006% | 1.50 | 0.41 | 1.7E-01 | Green |
| WP_011607894.1 | FRAAL6859 | PadR family transcriptional regulator | 29929 | 8.75 | 2 | 5 | Frankia-8 | F201371.dat | 67 | 10 | 2 | 2 | 2 | 0 | 0 | 0 | 1 | 0 | 0.001% | 0 | 0 | 2 | 2 | 0 | 0.005% | 1.50 | 0.41 | 1.7E-01 | Green |
| WP_041939965.1 | FRAAL6724 | phosphoribosylformylglycinamidine synthase | 24621 | 4.86 | 5 | 15 | Frankia-10 | F201373.dat | 234 | 28 | 4 | 4 | 4 | 1 | 0 | 0 | 2 | 2 | 0.007% | 2 | 0 | 4 | 0 | 4 | 0.014% | 1.50 | 0.41 | 1.7E-01 | Green |
| WP_011601916.1 | FRAAL0668 | thymidylate kinase | 22441 | 4.96 | 4 | 5 | Frankia-10 | F201373.dat | 114 | 18 | 3 | 3 | 3 | 0 | 0 | 1 | 0 | 0 | 0.002% | 1 | 0 | 0 | 0 | 3 | 0.006% | 1.50 | 0.41 | 2.0E-01 | Green |
| WP_011607709.1 | FRAAL6672 | L-aspartate oxidase | 58269 | 5.85 | 6 | 25 | Frankia-1 | F201364.dat | 156 | 11 | 4 | 4 | 4 | 4 | 2 | 4 | 2 | 4 | 0.010% | 0 | 2 | 3 | 1 | 3 | 0.005% | -1.50 | -0.41 | 5.2E-02 | Green |
| WP_041940962.1 | FRAAL5603 | protease | 21007 | 9.09 | 2 | 5 | Frankia-2 | F201365.dat | 85 | 9 | 1 | 1 | 1 | 0 | 1 | 1 | 1 | 1 | 0.007% | 0 | 0 | 1 | 0 | 0 | 0.002% | -1.50 | -0.41 | 1.2E-01 | Green |
| WP_011604591.1 | FRAAL3447 | beta-ketoacyl synthase | 222422 | 6.04 | 22 | 80 | Frankia-3 | F201366.dat | 608 | 12 | 16 | 16 | 17 | 12 | 11 | 17 | 4 | 5 | 0.008% | 12 | 2 | 6 | 7 | 4 | 0.005% | -1.50 | -0.41 | 1.3E-01 | Green |
| WP_041939793.1 | FRAAL5976 | ATPase AAA | 128352 | 5.51 | 3 | 5 | Frankia-6 | F201369.dat | 75 | 1 | 2 | 2 | 1 | 0 | 1 | 0 | 1 | 2 | 0.001% | 1 | 0 | 0 | 0 | 0 | 0.000% | -1.50 | -0.41 | 1.3E-01 | Green |
| WP_011605132.1 | FRAAL4002 | purine-nucleoside phosphorylase | 26636 | 6.09 | 2 | 5 | Frankia-3 | F201366.dat | 123 | 10 | 2 | 2 | 2 | 0 | 1 | 2 | 0 | 1 | 0.005% | 0 | 0 | 0 | 0 | 1 | 0.001% | -1.50 | -0.41 | 1.3E-01 | Green |
| WP_041939921.1 | FRAAL6572 | hypothetical protein | 30771 | 11.32 | 4 | 15 | Frankia-8 | F201371.dat | 123 | 19 | 4 | 4 | 4 | 2 | 2 | 2 | 3 | 1 | 0.012% | 0 | 0 | 4 | 0 | 1 | 0.005% | -1.50 | -0.41 | 1.3E-01 | Green |
| WP_011607191.1 | FRAAL6141 | DNA recombination protein RmuC | 54330 | 5.73 | 3 | 13 | Frankia-1 | F201364.dat | 111 | 7 | 3 | 3 | 3 | 3 | 2 | 0 | 2 | 2 | 0.006% | 2 | 1 | 0 | 1 | 0 | 0.002% | -1.56 | -0.44 | 7.2E-02 | Green |
| WP_041940122.1 | FRAAL0739 | FMN-linked alkanal monooxygenase | 38000 | 6.12 | 2 | 8 | Frankia-3 | F201366.dat | 64 | 8 | 2 | 2 | 2 | 1 | 0 | 2 | 1 | 2 | 0.006% | 1 | 0 | 1 | 0 | 0 | 0.002% | -1.57 | -0.45 | 7.4E-02 | Green |
| WP_011605616.1 | FRAAL4495 | GntR family transcriptional regulator | 32375 | 6.80 | 2 | 3 | Frankia-4 | F201367.dat | 63 | 6 | 1 | 1 | 1 | 0 | 1 | 1 | 1 | 0 | 0.003% | 0 | 0 | 0 | 0 | 0 | 0.000% | -1.60 | -0.47 | 1.2E-01 | Green |
| WP_050997478.1 | FRAAL6847 | DNA helicase | 92718 | 8.28 | 3 | 3 | Frankia-1 | F201364.dat | 50 | 2 | 2 | 2 | 2 | 2 | 0 | 1 | 0 | 0 | 0.001% | 0 | 0 | 0 | 0 | 0 | 0.000% | -1.60 | -0.47 | 1.4E-01 | Green |
| WP_041939900.1 | FRAAL6461 | hypothetical protein | 34933 | 5.48 | 2 | 3 | Frankia-3 | F201366.dat | 87 | 15 | 2 | 2 | 2 | 1 | 0 | 2 | 0 | 0 | 0.003% | 0 | 0 | 0 | 0 | 0 | 0.000% | -1.60 | -0.47 | 1.4E-01 | Green |
| WP_041939778.1 | FRAAL5894 | hypothetical protein | 96143 | 4.66 | 2 | 3 | Frankia-1 | F201364.dat | 57 | 3 | 2 | 2 | 2 | 2 | 0 | 1 | 0 | 0 | 0.001% | 0 | 0 | 0 | 0 | 0 | 0.000% | -1.60 | -0.47 | 1.4E-01 | Green |
| WP_011607059.1 | FRAAL6006 | hypothetical protein | 33837 | 4.94 | 2 | 3 | Frankia-1 | F201364.dat | 46 | 15 | 2 | 2 | 2 | 2 | 0 | 0 | 1 | 0 | 0.003% | 0 | 0 | 0 | 0 | 0 | 0.000% | -1.60 | -0.47 | 1.4E-01 | Green |
| WP_011601849.1 | FRAAL0599 | cytochrome BD ubiquinol oxidase subunit II | 34210 | 6.58 | 3 | 3 | Frankia-3 | F201366.dat | 61 | 10 | 3 | 3 | 3 | 0 | 0 | 3 | 0 | 0 | 0.003% | 0 | 0 | 0 | 0 | 0 | 0.000% | -1.60 | -0.47 | 2.0E-01 | Green |
| WP_011604866.1 | FRAAL3727 | protoporphyrin IX magnesium chelatase | 85549 | 8.38 | 2 | 3 | Frankia-5 | F201368.dat | 48 | 4 | 2 | 2 | 3 | 0 | 0 | 0 | 0 | 3 | 0.001% | 0 | 0 | 0 | 0 | 0 | 0.000% | -1.60 | -0.47 | 2.0E-01 | Green |
| WP_041938949.1 | FRAAL1589 | helicase | 95277 | 5.37 | 4 | 11 | Frankia-3 | F201366.dat | 177 | 4 | 3 | 3 | 3 | 2 | 1 | 3 | 2 | 0 | 0.003% | 1 | 1 | 1 | 0 | 0 | 0.001% | -1.63 | -0.49 | 6.9E-02 | Green |
| WP_011605057.1 | FRAAL3922 | RND transporter | 87330 | 10.81 | 3 | 6 | Frankia-3 | F201366.dat | 101 | 3 | 2 | 2 | 2 | 1 | 1 | 2 | 1 | 0 | 0.002% | 0 | 0 | 1 | 0 | 0 | 0.000% | -1.67 | -0.51 | 6.3E-02 | Green |
| WP_011605472.1 | FRAAL4350 | hypothetical protein | 36674 | 6.74 | 2 | 6 | Frankia-4 | F201367.dat | 147 | 11 | 2 | 2 | 2 | 1 | 0 | 1 | 2 | 1 | 0.005% | 0 | 0 | 0 | 0 | 1 | 0.001% | -1.67 | -0.51 | 6.3E-02 | Green |
| WP_011606592.1 | FRAAL5508 | hypothetical protein | 31491 | 5.51 | 2 | 6 | Frankia-2 | F201365.dat | 54 | 8 | 2 | 2 | 2 | 0 | 2 | 0 | 1 | 2 | 0.006% | 0 | 0 | 0 | 0 | 1 | 0.001% | -1.67 | -0.51 | 9.4E-02 | Green |
| WP_011605434.1 | FRAAL4308 | copper resistance protein CopD | 82004 | 11.08 | 4 | 14 | Frankia-8 | F201371.dat | 229 | 7 | 4 | 4 | 3 | 3 | 0 | 4 | 2 | 1 | 0.004% | 1 | 0 | 3 | 0 | 0 | 0.002% | -1.67 | -0.51 | 1.1E-01 | Green |
| WP_011601965.1 | FRAAL0723 | carnitine dehydratase | 48804 | 6.23 | 7 | 20 | Frankia-2 | F201365.dat | 188 | 12 | 5 | 5 | 5 | 3 | 5 | 3 | 0 | 3 | 0.010% | 0 | 0 | 3 | 2 | 1 | 0.004% | -1.73 | -0.55 | 7.2E-02 | Green |
| WP_011602230.1 | FRAAL1003 | cytochrome C biogenesis protein ResB | 74671 | 9.74 | 6 | 23 | Frankia-8 | F201371.dat | 167 | 9 | 5 | 5 | 5 | 4 | 3 | 5 | 0 | 4 | 0.008% | 0 | 0 | 5 | 1 | 1 | 0.003% | -1.75 | -0.56 | 9.6E-02 | Green |
| WP_011607289.1 | FRAAL6239 | hypothetical protein | 79913 | 8.62 | 2 | 4 | Frankia-5 | F201368.dat | 74 | 2 | 1 | 1 | 1 | 1 | 0 | 1 | 1 | 1 | 0.002% | 0 | 0 | 0 | 0 | 0 | 0.000% | -1.80 | -0.59 | 6.3E-02 | Green |
| WP_041938842.1 | FRAAL1046 | geranylgeranyl pyrophosphate synthase | 35818 | 4.74 | 2 | 4 | Frankia-5 | F201368.dat | 83 | 13 | 2 | 2 | 2 | 1 | 0 | 0 | 1 | 2 | 0.004% | 0 | 0 | 0 | 0 | 0 | 0.000% | -1.80 | -0.59 | 7.4E-02 | Green |
| WP_041938750.1 | FRAAL0713 | phospholipid-binding protein | 39644 | 4.73 | 2 | 4 | Frankia-4 | F201367.dat | 75 | 7 | 2 | 2 | 2 | 1 | 0 | 0 | 2 | 1 | 0.004% | 0 | 0 | 0 | 0 | 0 | 0.000% | -1.80 | -0.59 | 7.4E-02 | Green |
| WP_011606070.1 | FRAAL4956 | endoglucanase | 41625 | 9.20 | 2 | 4 | Frankia-5 | F201368.dat | 57 | 5 | 2 | 2 | 2 | 0 | 0 | 0 | 2 | 2 | 0.003% | 0 | 0 | 0 | 0 | 0 | 0.000% | -1.80 | -0.59 | 1.1E-01 | Green |
| WP_011606701.1 | FRAAL5622 | ATP-dependent RNA helicase HrpA | 165470 | 8.48 | 4 | 4 | Frankia-1 | F201364.dat | 148 | 3 | 4 | 4 | 4 | 4 | 0 | 0 | 0 | 0 | 0.001% | 0 | 0 | 0 | 0 | 0 | 0.000% | -1.80 | -0.59 | 1.9E-01 | Green |
| WP_011602368.1 | FRAAL1166 | forkhead-associated protein | 23253 | 10.33 | 3 | 7 | Frankia-4 | F201367.dat | 89 | 21 | 3 | 3 | 3 | 0 | 0 | 1 | 3 | 2 | 0.009% | 0 | 0 | 1 | 0 | 0 | 0.001% | -1.83 | -0.61 | 8.7E-02 | Green |
| WP_011605683.1 | FRAAL4566 | glycerol kinase | 55332 | 5.13 | 4 | 7 | Frankia-4 | F201367.dat | 91 | 9 | 3 | 3 | 4 | 0 | 0 | 2 | 4 | 0 | 0.004% | 0 | 0 | 1 | 0 | 0 | 0.001% | -1.83 | -0.61 | 1.4E-01 | Green |
| WP_041939628.1 | FRAAL5071 | hypothetical protein | 36066 | 10.92 | 4 | 5 | Frankia-5 | F201368.dat | 113 | 10 | 3 | 3 | 3 | 0 | 0 | 0 | 2 | 3 | 0.005% | 0 | 0 | 0 | 0 | 0 | 0.000% | -2.00 | -0.69 | 9.9E-02 | Green |
| WP_041938997.1 | FRAAL1813 | dynein regulation protein LC7 | 15523 | 5.16 | 2 | 10 | Frankia-10 | F201373.dat | 109 | 23 | 2 | 2 | 2 | 1 | 1 | 1 | 0 | 0 | 0.007% | 2 | 1 | 1 | 1 | 2 | 0.015% | 1.50 | 0.41 | 6.3E-02 | Red |
| WP_011601743.1 | FRAAL0490 | 30S ribosomal protein S18 | 9698 | 11.79 | 2 | 10 | Frankia-10 | F201373.dat | 75 | 26 | 2 | 2 | 2 | 1 | 1 | 0 | 1 | 0 | 0.011% | 1 | 1 | 2 | 1 | 2 | 0.024% | 1.50 | 0.41 | 6.3E-02 | Red |
| WP_011607820.1 | FRAAL6786 | adenosine deaminase | 35473 | 4.84 | 2 | 10 | Frankia-8 | F201371.dat | 133 | 8 | 2 | 2 | 2 | 0 | 0 | 1 | 1 | 1 | 0.003% | 2 | 2 | 2 | 0 | 1 | 0.007% | 1.50 | 0.41 | 8.1E-02 | Red |
| WP_011605021.1 | FRAAL3886 | transcriptional regulator | 27676 | 8.98 | 2 | 10 | Frankia-10 | F201373.dat | 121 | 10 | 2 | 2 | 2 | 1 | 0 | 0 | 1 | 1 | 0.004% | 1 | 0 | 2 | 2 | 2 | 0.009% | 1.50 | 0.41 | 8.1E-02 | Red |
| WP_050997283.1 | FRAAL6439 | ATPase | 36339 | 5.94 | 4 | 10 | Frankia-7 | F201370.dat | 138 | 12 | 3 | 3 | 3 | 1 | 1 | 1 | 0 | 0 | 0.003% | 2 | 3 | 1 | 1 | 0 | 0.006% | 1.50 | 0.41 | 1.1E-01 | Red |
| WP_011606486.1 | FRAAL5403 | F420-dependent oxidoreductase | 33577 | 6.10 | 5 | 10 | Frankia-8 | F201371.dat | 151 | 15 | 4 | 4 | 4 | 0 | 2 | 1 | 0 | 0 | 0.003% | 0 | 0 | 4 | 0 | 3 | 0.007% | 1.50 | 0.41 | 2.1E-01 | Red |
| WP_011606301.1 | FRAAL5207 | N-acetylglutamate synthase | 39165 | 4.51 | 9 | 57 | Frankia-10 | F201373.dat | 462 | 35 | 8 | 8 | 11 | 5 | 4 | 3 | 6 | 4 | 0.020% | 6 | 4 | 9 | 5 | 11 | 0.030% | 1.48 | 0.39 | 5.0E-02 | Red |
| WP_011603052.1 | FRAAL1875 | 2.5-diketo-D-gluconic acid reductase | 30140 | 6.41 | 5 | 32 | Frankia-10 | F201373.dat | 290 | 25 | 5 | 5 | 7 | 3 | 2 | 3 | 2 | 2 | 0.014% | 2 | 3 | 3 | 5 | 7 | 0.022% | 1.47 | 0.39 | 6.6E-02 | Red |
| WP_041938675.1 | FRAAL0382 | CoA-transferase | 43515 | 5.57 | 12 | 32 | Frankia-8 | F201371.dat | 220 | 18 | 5 | 5 | 5 | 4 | 0 | 3 | 1 | 4 | 0.010% | 4 | 4 | 5 | 2 | 5 | 0.015% | 1.47 | 0.39 | 7.1E-02 | Red |
| WP_041939848.1 | FRAAL6213 | transcription elongation factor GreA | 17333 | 4.87 | 5 | 27 | Frankia-9 | F201372.dat | 202 | 33 | 4 | 4 | 4 | 2 | 1 | 2 | 4 | 1 | 0.021% | 1 | 4 | 4 | 4 | 4 | 0.033% | 1.47 | 0.38 | 6.2E-02 | Red |
| WP_041938705.1 | FRAAL0509 | glycosyl hydrolase | 46479 | 7.09 | 6 | 27 | Frankia-6 | F201369.dat | 161 | 9 | 5 | 5 | 5 | 3 | 1 | 4 | 1 | 1 | 0.008% | 5 | 4 | 4 | 3 | 1 | 0.012% | 1.47 | 0.38 | 8.5E-02 | Red |
| WP_011604321.1 | FRAAL3172 | hypothetical protein | 30505 | 5.20 | 5 | 39 | Frankia-8 | F201371.dat | 394 | 39 | 7 | 3 | 5 | 0 | 4 | 2 | 7 | 2 | 0.018% | 4 | 6 | 5 | 5 | 4 | 0.026% | 1.45 | 0.37 | 9.2E-02 | Red |
| WP_041938975.1 | FRAAL1712 | monooxygenase | 11048 | 4.95 | 3 | 12 | Frankia-9 | F201372.dat | 151 | 37 | 2 | 2 | 2 | 1 | 1 | 1 | 1 | 0 | 0.013% | 1 | 2 | 1 | 2 | 2 | 0.024% | 1.44 | 0.37 | 6.3E-02 | Red |
| WP_041939039.1 | FRAAL2001 | 30S ribosomal protein S20 | 9836 | 11.44 | 14 | 122 | Frankia-10 | F201373.dat | 406 | 64 | 11 | 11 | 22 | 5 | 7 | 10 | 13 | 14 | 0.178% | 10 | 8 | 17 | 16 | 22 | 0.250% | 1.44 | 0.37 | 7.7E-02 | Red |
| WP_050997050.1 | FRAAL1965 | hypothetical protein | 74806 | 6.22 | 2 | 12 | Frankia-1 | F201364.dat | 101 | 4 | 2 | 2 | 2 | 2 | 0 | 2 | 0 | 0 | 0.002% | 2 | 2 | 1 | 2 | 1 | 0.004% | 1.44 | 0.37 | 1.1E-01 | Red |
| WP_011601494.1 | FRAAL0235 | TetR family transcriptional regulator | 23326 | 5.56 | 4 | 12 | Frankia-9 | F201372.dat | 132 | 15 | 3 | 3 | 3 | 2 | 0 | 2 | 0 | 0 | 0.006% | 1 | 2 | 2 | 3 | 0 | 0.012% | 1.44 | 0.37 | 1.5E-01 | Red |
| WP_011607681.1 | FRAAL6644 | PPOX class F420-dependent enzyme. Rv3369 family | 15905 | 6.58 | 3 | 12 | Frankia-8 | F201371.dat | 117 | 22 | 3 | 3 | 3 | 0 | 1 | 2 | 0 | 1 | 0.009% | 1 | 0 | 3 | 1 | 3 | 0.017% | 1.44 | 0.37 | 1.5E-01 | Red |
| WP_011602955.1 | FRAAL1772 | hypothetical protein | 169306 | 7.72 | 5 | 12 | Frankia-1 | F201364.dat | 112 | 1 | 3 | 3 | 3 | 3 | 0 | 1 | 0 | 0 | 0.001% | 3 | 0 | 2 | 2 | 1 | 0.002% | 1.44 | 0.37 | 1.7E-01 | Red |
| WP_011604920.1 | FRAAL3782 | DEAD/DEAH box helicase | 94548 | 5.22 | 8 | 29 | Frankia-8 | F201371.dat | 265 | 11 | 6 | 6 | 6 | 3 | 3 | 2 | 0 | 3 | 0.004% | 3 | 2 | 6 | 4 | 3 | 0.006% | 1.44 | 0.36 | 7.8E-02 | Red |
| WP_009742431.1 | FRAAL5684 | hypothetical protein | 7329 | 6.89 | 4 | 29 | Frankia-6 | F201369.dat | 119 | 73 | 4 | 4 | 5 | 2 | 3 | 3 | 3 | 0 | 0.054% | 5 | 5 | 0 | 5 | 3 | 0.083% | 1.44 | 0.36 | 1.3E-01 | Red |
| WP_011602261.1 | FRAAL1034 | NADH dehydrogenase subunit C | 26650 | 5.04 | 8 | 46 | Frankia-8 | F201371.dat | 253 | 50 | 7 | 7 | 7 | 4 | 4 | 2 | 3 | 5 | 0.024% | 3 | 3 | 7 | 7 | 8 | 0.035% | 1.43 | 0.36 | 6.6E-02 | Red |
| WP_011602178.1 | FRAAL0949 | hypothetical protein | 17716 | 4.76 | 2 | 7 | Frankia-9 | F201372.dat | 98 | 17 | 2 | 2 | 2 | 0 | 0 | 1 | 1 | 0 | 0.004% | 1 | 0 | 1 | 2 | 1 | 0.010% | 1.43 | 0.36 | 1.2E-01 | Red |
| WP_050996963.1 | FRAAL0230 | hypothetical protein | 51726 | 5.51 | 2 | 7 | Frankia-8 | F201371.dat | 85 | 3 | 2 | 2 | 2 | 1 | 0 | 0 | 1 | 0 | 0.001% | 1 | 0 | 2 | 1 | 1 | 0.003% | 1.43 | 0.36 | 1.2E-01 | Red |
| WP_011603474.1 | FRAAL2310 | hypothetical protein | 12826 | 10.16 | 2 | 7 | Frankia-9 | F201372.dat | 72 | 12 | 1 | 1 | 1 | 0 | 1 | 0 | 0 | 1 | 0.006% | 1 | 1 | 1 | 1 | 1 | 0.013% | 1.43 | 0.36 | 1.2E-01 | Red |
| WP_041939890.1 | FRAAL6411 | serine/threonine protein kinase | 112288 | 5.33 | 2 | 7 | Frankia-3 | F201366.dat | 77 | 2 | 2 | 2 | 2 | 0 | 0 | 2 | 0 | 0 | 0.001% | 1 | 1 | 1 | 1 | 1 | 0.001% | 1.43 | 0.36 | 1.4E-01 | Red |
| WP_041939805.1 | FRAAL6016 | GTPase | 34422 | 8.72 | 2 | 7 | Frankia-8 | F201371.dat | 135 | 8 | 2 | 2 | 2 | 0 | 0 | 1 | 1 | 0 | 0.002% | 0 | 2 | 2 | 1 | 0 | 0.005% | 1.43 | 0.36 | 1.6E-01 | Red |
| WP_011602147.1 | FRAAL0917 | kanosamine kinase | 35944 | 5.33 | 2 | 7 | Frankia-10 | F201373.dat | 108 | 4 | 2 | 2 | 2 | 0 | 1 | 0 | 1 | 0 | 0.002% | 0 | 0 | 2 | 1 | 2 | 0.005% | 1.43 | 0.36 | 1.6E-01 | Red |
| WP_011603613.1 | FRAAL2456 | hypothetical protein | 14578 | 7.93 | 2 | 7 | Frankia-10 | F201373.dat | 57 | 15 | 2 | 2 | 2 | 0 | 0 | 1 | 1 | 0 | 0.005% | 0 | 1 | 0 | 2 | 2 | 0.012% | 1.43 | 0.36 | 1.6E-01 | Red |
| WP_011606020.1 | FRAAL4906 | hypothetical protein | 27770 | 6.30 | 4 | 7 | Frankia-10 | F201373.dat | 161 | 10 | 2 | 2 | 2 | 0 | 0 | 0 | 0 | 2 | 0.003% | 1 | 0 | 0 | 2 | 2 | 0.006% | 1.43 | 0.36 | 1.7E-01 | Red |
| WP_011605651.1 | FRAAL4532 | LuxR family transcriptional regulator | 24383 | 4.87 | 3 | 7 | Frankia-10 | F201373.dat | 62 | 17 | 2 | 2 | 3 | 0 | 1 | 0 | 0 | 1 | 0.003% | 1 | 1 | 0 | 0 | 3 | 0.007% | 1.43 | 0.36 | 1.9E-01 | Red |
| WP_041939481.1 | FRAAL4302 | hypothetical protein | 27454 | 7.20 | 3 | 7 | Frankia-8 | F201371.dat | 142 | 14 | 3 | 3 | 3 | 0 | 0 | 2 | 0 | 0 | 0.003% | 0 | 1 | 3 | 1 | 0 | 0.006% | 1.43 | 0.36 | 2.0E-01 | Red |
| WP_050996966.1 | FRAAL0260 | hypothetical protein | 45249 | 5.33 | 3 | 7 | Frankia-8 | F201371.dat | 111 | 8 | 3 | 3 | 3 | 1 | 0 | 0 | 0 | 1 | 0.002% | 0 | 0 | 3 | 0 | 2 | 0.004% | 1.43 | 0.36 | 2.1E-01 | Red |
| WP_011601500.1 | FRAAL0242 | hypothetical protein | 40164 | 5.64 | 3 | 7 | Frankia-10 | F201373.dat | 77 | 13 | 3 | 3 | 3 | 0 | 0 | 0 | 2 | 0 | 0.002% | 0 | 0 | 2 | 0 | 3 | 0.004% | 1.43 | 0.36 | 2.2E-01 | Red |
| WP_011602394.1 | FRAAL1191 | serine/threonine protein kinase | 68550 | 6.30 | 4 | 7 | Frankia-8 | F201371.dat | 121 | 10 | 4 | 4 | 4 | 2 | 0 | 0 | 0 | 0 | 0.001% | 0 | 0 | 4 | 0 | 1 | 0.002% | 1.43 | 0.36 | 2.6E-01 | Red |
| WP_003956441.1 | FRAAL1106 | 50S ribosomal protein L36 [Actinobacteria] | 4386 | 10.69 | 2 | 19 | Frankia-9 | F201372.dat | 75 | 43 | 2 | 2 | 4 | 2 | 2 | 2 | 0 | 1 | 0.057% | 3 | 1 | 2 | 4 | 2 | 0.092% | 1.42 | 0.35 | 8.1E-02 | Red |
| WP_041940389.1 | FRAAL2457 | oxidoreductase | 30023 | 5.10 | 6 | 31 | Frankia-8 | F201371.dat | 298 | 25 | 5 | 5 | 7 | 4 | 2 | 2 | 3 | 1 | 0.014% | 2 | 3 | 7 | 3 | 4 | 0.021% | 1.41 | 0.34 | 1.0E-01 | Red |
| WP_011604826.1 | FRAAL3687 | hypothetical protein | 53916 | 5.13 | 6 | 31 | Frankia-9 | F201372.dat | 267 | 12 | 5 | 5 | 6 | 2 | 1 | 4 | 3 | 2 | 0.008% | 1 | 3 | 5 | 6 | 4 | 0.012% | 1.41 | 0.34 | 1.0E-01 | Red |
| WP_041938912.1 | FRAAL1415 | acyltransferase | 31307 | 4.90 | 12 | 103 | Frankia-8 | F201371.dat | 446 | 41 | 10 | 10 | 18 | 5 | 10 | 7 | 6 | 14 | 0.048% | 8 | 10 | 18 | 10 | 15 | 0.066% | 1.40 | 0.34 | 8.1E-02 | Red |
| WP_041939801.1 | FRAAL6004 | hypothetical protein | 7045 | 8.95 | 2 | 14 | Frankia-9 | F201372.dat | 96 | 22 | 2 | 2 | 2 | 1 | 1 | 2 | 1 | 0 | 0.025% | 2 | 1 | 2 | 2 | 2 | 0.043% | 1.40 | 0.34 | 6.3E-02 | Red |
| WP_009740545.1 | FRAAL1059 | 50S ribosomal protein L33 | 6464 | 9.96 | 5 | 50 | Frankia-9 | F201372.dat | 138 | 48 | 4 | 4 | 8 | 4 | 4 | 5 | 4 | 3 | 0.110% | 3 | 4 | 6 | 8 | 9 | 0.156% | 1.40 | 0.34 | 6.5E-02 | Red |
| WP_041939385.1 | FRAAL3734 | GTP cyclohydrolase | 11126 | 5.60 | 4 | 14 | Frankia-9 | F201372.dat | 126 | 39 | 3 | 3 | 3 | 1 | 1 | 0 | 2 | 1 | 0.016% | 1 | 1 | 2 | 3 | 2 | 0.027% | 1.40 | 0.34 | 7.4E-02 | Red |
| WP_011606204.1 | FRAAL5096 | cyclase | 15876 | 5.02 | 3 | 14 | Frankia-7 | F201370.dat | 173 | 17 | 2 | 2 | 3 | 1 | 0 | 2 | 1 | 1 | 0.011% | 1 | 3 | 2 | 1 | 2 | 0.019% | 1.40 | 0.34 | 7.4E-02 | Red |
| WP_011604612.1 | FRAAL3468 | carnitine dehydratase | 38477 | 5.31 | 3 | 14 | Frankia-8 | F201371.dat | 143 | 14 | 3 | 3 | 3 | 0 | 1 | 1 | 2 | 1 | 0.005% | 1 | 1 | 3 | 2 | 2 | 0.008% | 1.40 | 0.34 | 7.4E-02 | Red |
| WP_011602335.1 | FRAAL1128 | tRNA threonylcarbamoyladenosine biosynthesis protein TsaB | 26556 | 5.20 | 5 | 14 | Frankia-8 | F201371.dat | 137 | 19 | 3 | 3 | 3 | 2 | 2 | 0 | 1 | 0 | 0.007% | 1 | 3 | 3 | 2 | 0 | 0.011% | 1.40 | 0.34 | 1.5E-01 | Red |
| WP_011605237.1 | FRAAL4108 | hypothetical protein | 10105 | 6.05 | 2 | 2 | Frankia-9 | F201372.dat | 202 | 29 | 2 | 1 | 1 | 0 | 0 | 0 | 0 | 0 | 0.000% | 1 | 0 | 0 | 1 | 0 | 0.007% | 1.40 | 0.34 | 2.1E-01 | Red |
| WP_041940551.1 | FRAAL3357 | septum formation inhibitor Maf | 20697 | 5.28 | 2 | 2 | Frankia-8 | F201371.dat | 89 | 11 | 1 | 1 | 1 | 0 | 0 | 0 | 0 | 0 | 0.000% | 0 | 0 | 1 | 1 | 0 | 0.003% | 1.40 | 0.34 | 2.1E-01 | Red |
| WP_041939713.1 | FRAAL5478 | IclR family transcriptional regulator | 25830 | 6.43 | 2 | 2 | Frankia-8 | F201371.dat | 47 | 3 | 1 | 1 | 1 | 0 | 0 | 0 | 0 | 0 | 0.000% | 0 | 0 | 1 | 1 | 0 | 0.003% | 1.40 | 0.34 | 2.1E-01 | Red |
| WP_011602738.1 | FRAAL1550 | beta-ketoacyl synthase | 187400 | 6.14 | 2 | 2 | Frankia-9 | F201372.dat | 47 | 1 | 2 | 2 | 1 | 0 | 0 | 0 | 0 | 0 | 0.000% | 0 | 0 | 1 | 1 | 0 | 0.000% | 1.40 | 0.34 | 2.1E-01 | Red |
| WP_050997458.1 | FRAAL6081 | 4-aminobutyrate aminotransferase | 47407 | 6.22 | 2 | 2 | Frankia-10 | F201373.dat | 44 | 3 | 1 | 1 | 1 | 0 | 0 | 0 | 0 | 0 | 0.000% | 0 | 0 | 1 | 0 | 1 | 0.001% | 1.40 | 0.34 | 2.1E-01 | Red |
| WP_011603110.1 | FRAAL1937 | ribosome silencing factor RsfS | 15704 | 5.05 | 2 | 2 | Frankia-8 | F201371.dat | 40 | 9 | 1 | 1 | 1 | 0 | 0 | 0 | 0 | 0 | 0.000% | 0 | 0 | 1 | 0 | 1 | 0.004% | 1.40 | 0.34 | 2.1E-01 | Red |
| WP_011606189.1 | FRAAL5080 | thiazole synthase | 26972 | 5.77 | 2 | 2 | Frankia-7 | F201370.dat | 40 | 4 | 1 | 1 | 1 | 0 | 0 | 0 | 0 | 0 | 0.000% | 0 | 1 | 1 | 0 | 0 | 0.002% | 1.40 | 0.34 | 2.1E-01 | Red |
| WP_011607540.1 | FRAAL6499 | beta-lactamase | 51047 | 6.94 | 2 | 2 | Frankia-8 | F201371.dat | 38 | 2 | 1 | 1 | 1 | 0 | 0 | 0 | 0 | 0 | 0.000% | 0 | 0 | 1 | 1 | 0 | 0.001% | 1.40 | 0.34 | 2.1E-01 | Red |
| WP_011604380.1 | FRAAL3231 | undecaprenyl-diphosphatase 1 | 29220 | 6.91 | 2 | 2 | Frankia-7 | F201370.dat | 38 | 4 | 1 | 1 | 1 | 0 | 0 | 0 | 0 | 0 | 0.000% | 0 | 1 | 1 | 0 | 0 | 0.002% | 1.40 | 0.34 | 2.1E-01 | Red |
| WP_041941120.1 | FRAAL6544 | metallophosphoesterase | 32260 | 10.28 | 2 | 2 | Frankia-6 | F201369.dat | 34 | 6 | 1 | 1 | 1 | 0 | 0 | 0 | 0 | 0 | 0.000% | 1 | 0 | 0 | 0 | 1 | 0.002% | 1.40 | 0.34 | 2.1E-01 | Red |
| WP_011603366.1 | FRAAL2201 | laccase | 23595 | 6.21 | 2 | 2 | Frankia-10 | F201373.dat | 32 | 4 | 1 | 1 | 1 | 0 | 0 | 0 | 0 | 0 | 0.000% | 0 | 0 | 0 | 1 | 1 | 0.003% | 1.40 | 0.34 | 2.1E-01 | Red |
| WP_041939517.1 | FRAAL4483 | sporulation protein | 28312 | 5.44 | 3 | 14 | Frankia-3 | F201366.dat | 141 | 26 | 3 | 3 | 3 | 2 | 0 | 3 | 0 | 0 | 0.006% | 0 | 2 | 4 | 3 | 0 | 0.011% | 1.40 | 0.34 | 2.3E-01 | Red |
| WP_041940726.1 | FRAAL4260 | glutamyl-tRNA amidotransferase | 38497 | 7.06 | 2 | 2 | Frankia-10 | F201373.dat | 105 | 6 | 2 | 2 | 2 | 0 | 0 | 0 | 0 | 0 | 0.000% | 0 | 0 | 0 | 0 | 2 | 0.002% | 1.40 | 0.34 | 2.3E-01 | Red |
| WP_041939329.1 | FRAAL3338 | 3-ketoacyl-ACP reductase | 28514 | 5.23 | 2 | 2 | Frankia-8 | F201371.dat | 99 | 16 | 3 | 2 | 2 | 0 | 0 | 0 | 0 | 0 | 0.000% | 0 | 0 | 2 | 0 | 0 | 0.002% | 1.40 | 0.34 | 2.3E-01 | Red |
| WP_050997129.1 | FRAAL3410 | alpha-dehydro-beta-deoxy-D-glucarate aldolase | 29156 | 5.26 | 2 | 2 | Frankia-8 | F201371.dat | 92 | 9 | 2 | 2 | 2 | 0 | 0 | 0 | 0 | 0 | 0.000% | 0 | 0 | 2 | 0 | 0 | 0.002% | 1.40 | 0.34 | 2.3E-01 | Red |
| WP_041940313.1 | FRAAL1968 | acyltransferase | 28739 | 6.50 | 2 | 2 | Frankia-8 | F201371.dat | 70 | 8 | 2 | 2 | 2 | 0 | 0 | 0 | 0 | 0 | 0.000% | 0 | 0 | 2 | 0 | 0 | 0.002% | 1.40 | 0.34 | 2.3E-01 | Red |
| WP_011606188.1 | FRAAL5079 | thiamine biosynthesis protein ThiH | 42763 | 6.99 | 2 | 2 | Frankia-8 | F201371.dat | 69 | 8 | 2 | 2 | 2 | 0 | 0 | 0 | 0 | 0 | 0.000% | 0 | 0 | 2 | 0 | 0 | 0.002% | 1.40 | 0.34 | 2.3E-01 | Red |
| WP_011605567.1 | FRAAL4446 | peptide ABC transporter substrate-binding protein | 58421 | 7.01 | 2 | 2 | Frankia-8 | F201371.dat | 67 | 4 | 2 | 2 | 2 | 0 | 0 | 0 | 0 | 0 | 0.000% | 0 | 0 | 2 | 0 | 0 | 0.001% | 1.40 | 0.34 | 2.3E-01 | Red |
| WP_011607735.1 | FRAAL6698 | alpha.alpha-trehalose-phosphate synthase | 54118 | 6.05 | 2 | 2 | Frankia-7 | F201370.dat | 62 | 5 | 2 | 2 | 2 | 0 | 0 | 0 | 0 | 0 | 0.000% | 0 | 2 | 0 | 0 | 0 | 0.001% | 1.40 | 0.34 | 2.3E-01 | Red |
| WP_041940062.1 | FRAAL0372 | methyltransferase | 28563 | 5.52 | 2 | 2 | Frankia-8 | F201371.dat | 58 | 7 | 2 | 2 | 2 | 0 | 0 | 0 | 0 | 0 | 0.000% | 0 | 0 | 2 | 0 | 0 | 0.002% | 1.40 | 0.34 | 2.3E-01 | Red |
| WP_011605588.1 | FRAAL4465 | hypothetical protein | 15934 | 5.17 | 2 | 2 | Frankia-8 | F201371.dat | 49 | 22 | 2 | 2 | 2 | 0 | 0 | 0 | 0 | 0 | 0.000% | 0 | 0 | 2 | 0 | 0 | 0.004% | 1.40 | 0.34 | 2.3E-01 | Red |
| WP_009739962.1 | FRAAL1782 | hypothetical protein | 6803 | 5.51 | 2 | 2 | Frankia-9 | F201372.dat | 48 | 50 | 2 | 2 | 2 | 0 | 0 | 0 | 0 | 0 | 0.000% | 0 | 0 | 0 | 2 | 0 | 0.010% | 1.40 | 0.34 | 2.3E-01 | Red |
| WP_041941133.1 | FRAAL6613 | transcriptional regulator | 17572 | 5.72 | 2 | 2 | Frankia-8 | F201371.dat | 47 | 11 | 2 | 2 | 2 | 0 | 0 | 0 | 0 | 0 | 0.000% | 0 | 0 | 2 | 0 | 0 | 0.004% | 1.40 | 0.34 | 2.3E-01 | Red |
| WP_041940515.1 | FRAAL3117 | citrate lyase subunit beta | 27499 | 5.40 | 2 | 2 | Frankia-8 | F201371.dat | 45 | 8 | 2 | 2 | 2 | 0 | 0 | 0 | 0 | 0 | 0.000% | 0 | 0 | 2 | 0 | 0 | 0.002% | 1.40 | 0.34 | 2.3E-01 | Red |
| WP_011603307.1 | FRAAL2142 | preprotein translocase subunit YajC | 17092 | 4.47 | 2 | 2 | Frankia-8 | F201371.dat | 44 | 6 | 2 | 2 | 2 | 0 | 0 | 0 | 0 | 0 | 0.000% | 0 | 0 | 2 | 0 | 0 | 0.004% | 1.40 | 0.34 | 2.3E-01 | Red |
| WP_041940412.1 | FRAAL2564 | nonribosomal peptide synthase | 114293 | 5.27 | 2 | 2 | Frankia-8 | F201371.dat | 44 | 2 | 2 | 2 | 2 | 0 | 0 | 0 | 0 | 0 | 0.000% | 0 | 0 | 2 | 0 | 0 | 0.001% | 1.40 | 0.34 | 2.3E-01 | Red |
| WP_011606802.1 | FRAAL5728 | phage-shock protein | 32342 | 6.13 | 9 | 57 | Frankia-7 | F201370.dat | 348 | 31 | 7 | 7 | 8 | 4 | 4 | 3 | 7 | 5 | 0.025% | 3 | 8 | 8 | 8 | 7 | 0.035% | 1.39 | 0.33 | 5.0E-02 | Red |
| WP_011607572.1 | FRAAL6532 | phosphate ABC transporter ATP-binding protein | 28156 | 8.38 | 5 | 21 | Frankia-10 | F201373.dat | 142 | 18 | 4 | 4 | 4 | 2 | 3 | 2 | 1 | 0 | 0.010% | 3 | 1 | 3 | 2 | 4 | 0.016% | 1.38 | 0.33 | 1.0E-01 | Red |
| WP_011605427.1 | FRAAL4301 | DNA mismatch repair protein MutT | 17212 | 4.98 | 6 | 21 | Frankia-10 | F201373.dat | 247 | 48 | 5 | 5 | 5 | 1 | 1 | 2 | 2 | 2 | 0.017% | 1 | 1 | 4 | 2 | 5 | 0.025% | 1.38 | 0.33 | 1.4E-01 | Red |
| WP_011603302.1 | FRAAL2137 | pyridoxal 5'-phosphate synthase subunit PdxT | 21053 | 5.23 | 8 | 40 | Frankia-8 | F201371.dat | 344 | 44 | 7 | 7 | 8 | 2 | 3 | 4 | 3 | 4 | 0.027% | 3 | 3 | 8 | 4 | 6 | 0.038% | 1.38 | 0.32 | 8.1E-02 | Red |
| WP_011603313.1 | FRAAL2148 | (p)ppGpp synthetase | 81239 | 6.15 | 6 | 28 | Frankia-8 | F201371.dat | 149 | 12 | 5 | 5 | 5 | 2 | 2 | 3 | 3 | 1 | 0.005% | 4 | 2 | 5 | 2 | 4 | 0.007% | 1.38 | 0.32 | 6.4E-02 | Red |
| WP_011604167.1 | FRAAL3017 | PPOX class F420-dependent enzyme | 14633 | 9.50 | 2 | 9 | Frankia-8 | F201371.dat | 126 | 24 | 3 | 1 | 1 | 1 | 0 | 1 | 0 | 1 | 0.007% | 1 | 1 | 1 | 2 | 1 | 0.014% | 1.38 | 0.32 | 1.2E-01 | Red |
| WP_041940390.1 | FRAAL2461 | aminoglycoside adenylyltransferase | 18615 | 5.27 | 2 | 9 | Frankia-8 | F201371.dat | 87 | 16 | 2 | 2 | 2 | 1 | 0 | 1 | 1 | 0 | 0.006% | 1 | 1 | 2 | 1 | 1 | 0.011% | 1.38 | 0.32 | 1.2E-01 | Red |
| WP_011607342.1 | FRAAL6294 | hypothetical protein | 19864 | 6.09 | 2 | 9 | Frankia-10 | F201373.dat | 72 | 12 | 2 | 2 | 2 | 1 | 0 | 1 | 1 | 0 | 0.005% | 1 | 1 | 1 | 1 | 2 | 0.010% | 1.38 | 0.32 | 1.2E-01 | Red |
| WP_011607748.1 | FRAAL6711 | GCN5 family acetyltransferase | 30882 | 6.91 | 2 | 9 | Frankia-7 | F201370.dat | 93 | 8 | 2 | 2 | 2 | 1 | 0 | 1 | 0 | 1 | 0.003% | 2 | 2 | 1 | 0 | 1 | 0.007% | 1.38 | 0.32 | 1.3E-01 | Red |
| WP_011607745.1 | FRAAL6708 | 2-amino-4-hydroxy-6-hydroxymethyldihydropteridine pyrophosphokinase | 21878 | 4.98 | 2 | 9 | Frankia-8 | F201371.dat | 97 | 14 | 2 | 2 | 2 | 0 | 0 | 0 | 2 | 1 | 0.005% | 0 | 0 | 2 | 2 | 2 | 0.009% | 1.38 | 0.32 | 1.9E-01 | Red |
| WP_050996957.1 | FRAAL0177 | GntR family transcriptional regulator | 60609 | 6.49 | 4 | 9 | Frankia-8 | F201371.dat | 172 | 10 | 3 | 3 | 3 | 1 | 0 | 2 | 0 | 0 | 0.002% | 0 | 2 | 3 | 0 | 1 | 0.003% | 1.38 | 0.32 | 2.1E-01 | Red |
| WP_011606046.1 | FRAAL4932 | S-adenosylmethionine synthase | 43050 | 5.16 | 5 | 9 | Frankia-8 | F201371.dat | 369 | 16 | 7 | 2 | 2 | 3 | 0 | 0 | 0 | 0 | 0.002% | 0 | 0 | 2 | 2 | 2 | 0.005% | 1.38 | 0.32 | 2.3E-01 | Red |
| WP_009741495.1 | FRAAL6851 | 30S ribosomal protein S18 | 8899 | 10.26 | 8 | 73 | Frankia-8 | F201371.dat | 292 | 59 | 7 | 7 | 14 | 6 | 7 | 7 | 5 | 5 | 0.120% | 7 | 7 | 14 | 6 | 9 | 0.163% | 1.37 | 0.32 | 6.1E-02 | Red |
| WP_041939937.1 | FRAAL6642 | MerR family transcriptional regulator | 20599 | 10.59 | 3 | 16 | Frankia-9 | F201372.dat | 153 | 15 | 3 | 3 | 3 | 1 | 2 | 1 | 1 | 1 | 0.010% | 2 | 1 | 2 | 3 | 2 | 0.016% | 1.36 | 0.31 | 6.3E-02 | Red |
| WP_011602739.1 | FRAAL1551 | acyl carrier protein | 12434 | 4.14 | 3 | 16 | Frankia-6 | F201369.dat | 112 | 23 | 2 | 2 | 2 | 2 | 1 | 1 | 1 | 1 | 0.017% | 2 | 1 | 2 | 3 | 2 | 0.027% | 1.36 | 0.31 | 6.3E-02 | Red |
| WP_011605047.1 | FRAAL3912 | NUDIX hydrolase | 25922 | 5.22 | 2 | 16 | Frankia-8 | F201371.dat | 88 | 11 | 2 | 2 | 2 | 1 | 1 | 2 | 1 | 1 | 0.008% | 2 | 1 | 2 | 2 | 3 | 0.013% | 1.36 | 0.31 | 6.3E-02 | Red |
| WP_050997389.1 | FRAAL3764 | ABC transporter permease | 31875 | 9.94 | 2 | 16 | Frankia-9 | F201372.dat | 79 | 8 | 2 | 2 | 3 | 1 | 1 | 1 | 1 | 2 | 0.007% | 2 | 2 | 1 | 3 | 2 | 0.011% | 1.36 | 0.31 | 6.3E-02 | Red |
| WP_011602372.1 | FRAAL1170 | penicillin-binding protein | 29641 | 5.35 | 4 | 16 | Frankia-7 | F201370.dat | 145 | 10 | 3 | 3 | 3 | 2 | 1 | 0 | 1 | 2 | 0.007% | 2 | 3 | 2 | 1 | 2 | 0.011% | 1.36 | 0.31 | 7.4E-02 | Red |
| WP_041940299.1 | FRAAL1889 | glutamate synthase | 21953 | 7.89 | 4 | 16 | Frankia-7 | F201370.dat | 103 | 18 | 3 | 3 | 3 | 1 | 0 | 2 | 1 | 2 | 0.010% | 2 | 3 | 2 | 2 | 1 | 0.015% | 1.36 | 0.31 | 7.4E-02 | Red |
| WP_041938824.1 | FRAAL0970 | ubiquinone biosynthesis protein UbiE | 28519 | 8.88 | 2 | 16 | Frankia-8 | F201371.dat | 76 | 10 | 3 | 3 | 2 | 2 | 1 | 0 | 2 | 1 | 0.008% | 2 | 1 | 2 | 3 | 2 | 0.012% | 1.36 | 0.31 | 7.4E-02 | Red |
| WP_050996974.1 | FRAAL0373 | hypothetical protein | 23686 | 5.88 | 3 | 16 | Frankia-10 | F201373.dat | 197 | 20 | 3 | 3 | 4 | 1 | 0 | 1 | 2 | 2 | 0.009% | 1 | 1 | 2 | 2 | 4 | 0.014% | 1.36 | 0.31 | 1.3E-01 | Red |
| WP_011607083.1 | FRAAL6031 | molybdopterin synthase | 19427 | 5.15 | 3 | 16 | Frankia-3 | F201366.dat | 196 | 33 | 3 | 3 | 3 | 2 | 0 | 3 | 1 | 0 | 0.011% | 2 | 2 | 2 | 1 | 3 | 0.017% | 1.36 | 0.31 | 1.3E-01 | Red |
| WP_041939542.1 | FRAAL4607 | excinuclease ABC subunit B | 80288 | 5.26 | 8 | 16 | Frankia-8 | F201371.dat | 154 | 6 | 4 | 4 | 4 | 1 | 3 | 2 | 0 | 0 | 0.003% | 3 | 1 | 4 | 2 | 0 | 0.004% | 1.36 | 0.31 | 2.0E-01 | Red |
| WP_011602121.1 | FRAAL0890 | peptidoglycan-binding protein LysM | 171079 | 4.95 | 8 | 30 | Frankia-8 | F201371.dat | 192 | 4 | 5 | 5 | 5 | 3 | 3 | 1 | 1 | 4 | 0.003% | 4 | 1 | 5 | 3 | 5 | 0.004% | 1.35 | 0.30 | 1.2E-01 | Red |
| WP_041940170.1 | FRAAL1092 | 50S ribosomal protein L24 | 11555 | 10.72 | 7 | 91 | Frankia-10 | F201373.dat | 351 | 70 | 7 | 7 | 15 | 7 | 6 | 7 | 9 | 9 | 0.117% | 7 | 8 | 9 | 14 | 15 | 0.154% | 1.35 | 0.30 | 6.1E-02 | Red |
| WP_041939771.1 | FRAAL5852 | penicillin-binding protein | 61497 | 9.58 | 17 | 114 | Frankia-9 | F201372.dat | 709 | 40 | 15 | 15 | 18 | 9 | 15 | 11 | 6 | 7 | 0.028% | 12 | 15 | 10 | 18 | 11 | 0.036% | 1.34 | 0.29 | 6.8E-02 | Red |
| WP_041940635.1 | FRAAL3808 | amino acid transporter | 69180 | 6.10 | 2 | 18 | Frankia-6 | F201369.dat | 96 | 4 | 2 | 2 | 3 | 2 | 1 | 1 | 2 | 1 | 0.004% | 3 | 2 | 2 | 2 | 2 | 0.005% | 1.33 | 0.29 | 6.3E-02 | Red |
| WP_011605998.1 | FRAAL4884 | FMNH2-utilizing oxygenase | 49730 | 5.33 | 10 | 46 | Frankia-8 | F201371.dat | 354 | 21 | 7 | 7 | 7 | 4 | 2 | 6 | 3 | 4 | 0.014% | 4 | 7 | 7 | 4 | 5 | 0.018% | 1.33 | 0.29 | 6.5E-02 | Red |
| WP_011605821.1 | FRAAL4707 | SAM-dependent methyltransferase | 30673 | 5.12 | 3 | 18 | Frankia-10 | F201373.dat | 178 | 12 | 3 | 3 | 3 | 1 | 1 | 1 | 2 | 2 | 0.008% | 2 | 3 | 1 | 2 | 3 | 0.012% | 1.33 | 0.29 | 7.4E-02 | Red |
| WP_011601710.1 | FRAAL0454 | CRISPR-associated protein Cse2 | 17724 | 11.28 | 2 | 18 | Frankia-8 | F201371.dat | 89 | 18 | 2 | 2 | 3 | 2 | 1 | 2 | 1 | 1 | 0.014% | 1 | 2 | 3 | 3 | 2 | 0.021% | 1.33 | 0.29 | 7.4E-02 | Red |
| WP_011601504.1 | FRAAL0246 | helicase | 108714 | 9.14 | 19 | 81 | Frankia-8 | F201371.dat | 629 | 17 | 14 | 14 | 15 | 8 | 7 | 9 | 5 | 5 | 0.011% | 7 | 9 | 15 | 7 | 9 | 0.015% | 1.33 | 0.29 | 7.9E-02 | Red |
| WP_041938840.1 | FRAAL1033 | NADH-quinone oxidoreductase subunit B | 29712 | 6.96 | 12 | 109 | Frankia-8 | F201371.dat | 538 | 47 | 11 | 11 | 19 | 7 | 10 | 9 | 10 | 10 | 0.055% | 5 | 13 | 19 | 13 | 13 | 0.071% | 1.33 | 0.29 | 8.9E-02 | Red |
| WP_011603799.1 | FRAAL2646 | hypothetical protein | 61444 | 7.23 | 11 | 46 | Frankia-8 | F201371.dat | 378 | 20 | 9 | 9 | 9 | 4 | 3 | 2 | 5 | 5 | 0.011% | 3 | 4 | 9 | 6 | 5 | 0.015% | 1.33 | 0.29 | 1.1E-01 | Red |
| WP_011602334.1 | FRAAL1127 | tRNA threonylcarbamoyladenosine biosynthesis protein TsaE | 17210 | 4.90 | 3 | 11 | Frankia-8 | F201371.dat | 119 | 19 | 2 | 2 | 2 | 1 | 1 | 0 | 1 | 1 | 0.008% | 1 | 1 | 2 | 2 | 1 | 0.014% | 1.33 | 0.29 | 1.2E-01 | Red |
| WP_011607351.1 | FRAAL6303 | acyl-CoA thioesterase | 31695 | 5.91 | 2 | 11 | Frankia-10 | F201373.dat | 70 | 9 | 2 | 2 | 2 | 1 | 0 | 1 | 1 | 1 | 0.005% | 1 | 2 | 1 | 1 | 2 | 0.007% | 1.33 | 0.29 | 1.2E-01 | Red |
| WP_011603247.1 | FRAAL2081 | hypothetical protein | 24939 | 4.95 | 3 | 18 | Frankia-7 | F201370.dat | 137 | 15 | 3 | 3 | 3 | 0 | 1 | 2 | 3 | 1 | 0.010% | 1 | 3 | 3 | 2 | 2 | 0.015% | 1.33 | 0.29 | 1.2E-01 | Red |
| WP_050997348.1 | FRAAL2202 | PLP-binding domain-containing protein | 30608 | 6.14 | 4 | 18 | Frankia-4 | F201367.dat | 119 | 9 | 3 | 3 | 3 | 2 | 2 | 0 | 3 | 0 | 0.008% | 1 | 2 | 2 | 3 | 3 | 0.012% | 1.33 | 0.29 | 1.5E-01 | Red |
| WP_011604499.1 | FRAAL3352 | phosphatidylinositol diacylglycerol-lyase | 40112 | 4.55 | 3 | 4 | Frankia-5 | F201368.dat | 64 | 4 | 1 | 1 | 1 | 0 | 0 | 0 | 0 | 1 | 0.001% | 1 | 0 | 0 | 1 | 1 | 0.003% | 1.33 | 0.29 | 2.1E-01 | Red |
| WP_011601678.1 | FRAAL0422 | 3-beta hydroxysteroid dehydrogenase | 31910 | 4.97 | 2 | 4 | Frankia-5 | F201368.dat | 65 | 6 | 1 | 1 | 1 | 0 | 0 | 0 | 0 | 1 | 0.001% | 0 | 1 | 0 | 1 | 1 | 0.003% | 1.33 | 0.29 | 2.1E-01 | Red |
| WP_011607511.1 | FRAAL6466 | macrolide ABC transporter ATP-binding protein | 28696 | 5.20 | 2 | 4 | Frankia-8 | F201371.dat | 58 | 5 | 1 | 1 | 1 | 0 | 0 | 0 | 0 | 1 | 0.001% | 0 | 1 | 1 | 1 | 0 | 0.004% | 1.33 | 0.29 | 2.1E-01 | Red |
| WP_050997363.1 | FRAAL2880 | transcriptional regulator | 41423 | 7.72 | 2 | 4 | Frankia-8 | F201371.dat | 55 | 5 | 1 | 1 | 1 | 0 | 0 | 1 | 0 | 0 | 0.001% | 1 | 0 | 1 | 0 | 1 | 0.002% | 1.33 | 0.29 | 2.1E-01 | Red |
| WP_011604643.1 | FRAAL3502 | hypothetical protein | 39455 | 5.80 | 4 | 4 | Frankia-8 | F201371.dat | 41 | 6 | 2 | 2 | 2 | 0 | 0 | 0 | 0 | 1 | 0.001% | 0 | 0 | 2 | 0 | 1 | 0.003% | 1.33 | 0.29 | 2.3E-01 | Red |
| WP_050997339.1 | FRAAL1885 | hypothetical protein | 29397 | 10.35 | 3 | 4 | Frankia-6 | F201369.dat | 56 | 6 | 2 | 2 | 2 | 0 | 0 | 0 | 0 | 1 | 0.001% | 2 | 0 | 1 | 0 | 0 | 0.003% | 1.33 | 0.29 | 2.3E-01 | Red |
| WP_011605555.1 | FRAAL4433 | hypothetical protein | 21336 | 4.88 | 2 | 4 | Frankia-7 | F201370.dat | 123 | 12 | 2 | 2 | 2 | 0 | 0 | 0 | 1 | 0 | 0.002% | 0 | 2 | 1 | 0 | 0 | 0.005% | 1.33 | 0.29 | 2.3E-01 | Red |
| WP_011602498.1 | FRAAL1299 | hypothetical protein | 48702 | 10.18 | 2 | 4 | Frankia-7 | F201370.dat | 103 | 6 | 2 | 2 | 2 | 0 | 0 | 0 | 1 | 0 | 0.001% | 0 | 2 | 0 | 1 | 0 | 0.002% | 1.33 | 0.29 | 2.3E-01 | Red |
| WP_041938758.1 | FRAAL0734 | FAD-linked oxidoreductase | 49172 | 9.04 | 2 | 4 | Frankia-8 | F201371.dat | 103 | 6 | 2 | 2 | 2 | 0 | 0 | 1 | 0 | 0 | 0.001% | 1 | 0 | 2 | 0 | 0 | 0.002% | 1.33 | 0.29 | 2.3E-01 | Red |
| WP_041940052.1 | FRAAL0321 | short-chain dehydrogenase | 26698 | 5.33 | 2 | 4 | Frankia-8 | F201371.dat | 102 | 7 | 2 | 2 | 2 | 0 | 0 | 1 | 0 | 0 | 0.001% | 0 | 0 | 2 | 0 | 1 | 0.004% | 1.33 | 0.29 | 2.3E-01 | Red |
| WP_050997338.1 | FRAAL1860 | permease | 40576 | 9.93 | 2 | 4 | Frankia-2 | F201365.dat | 71 | 8 | 1 | 1 | 1 | 0 | 1 | 0 | 0 | 0 | 0.001% | 1 | 2 | 0 | 0 | 0 | 0.002% | 1.33 | 0.29 | 2.3E-01 | Red |
| WP_041939709.1 | FRAAL5460 | TetR family transcriptional regulator | 20633 | 8.97 | 2 | 4 | Frankia-10 | F201373.dat | 68 | 16 | 2 | 2 | 2 | 0 | 0 | 1 | 0 | 0 | 0.002% | 0 | 0 | 1 | 0 | 2 | 0.005% | 1.33 | 0.29 | 2.3E-01 | Red |
| WP_011601983.1 | FRAAL0742 | patatin | 29382 | 7.14 | 2 | 4 | Frankia-7 | F201370.dat | 55 | 10 | 2 | 2 | 2 | 0 | 0 | 0 | 0 | 1 | 0.001% | 0 | 2 | 0 | 0 | 1 | 0.003% | 1.33 | 0.29 | 2.3E-01 | Red |
| WP_011606709.1 | FRAAL5630 | FMN reductase | 19358 | 5.32 | 2 | 4 | Frankia-9 | F201372.dat | 54 | 19 | 2 | 2 | 2 | 1 | 0 | 0 | 0 | 0 | 0.002% | 0 | 0 | 1 | 2 | 0 | 0.005% | 1.33 | 0.29 | 2.3E-01 | Red |
| WP_011605483.1 | FRAAL4361 | phosphodiesterase | 65969 | 5.75 | 5 | 11 | Frankia-8 | F201371.dat | 184 | 13 | 4 | 4 | 4 | 0 | 0 | 3 | 0 | 1 | 0.002% | 0 | 0 | 4 | 2 | 1 | 0.004% | 1.33 | 0.29 | 2.7E-01 | Red |
| WP_011604563.1 | FRAAL3417 | acyl dehydratase | 22766 | 6.29 | 5 | 11 | Frankia-8 | F201371.dat | 122 | 24 | 3 | 3 | 4 | 0 | 0 | 2 | 0 | 2 | 0.006% | 0 | 3 | 4 | 0 | 0 | 0.010% | 1.33 | 0.29 | 2.8E-01 | Red |
| WP_011606716.1 | FRAAL5637 | amidohydrolase | 38430 | 5.30 | 3 | 4 | Frankia-8 | F201371.dat | 90 | 13 | 3 | 3 | 3 | 0 | 0 | 0 | 0 | 1 | 0.001% | 0 | 0 | 3 | 0 | 0 | 0.003% | 1.33 | 0.29 | 2.9E-01 | Red |
| WP_050997084.1 | FRAAL2534 | hypothetical protein | 102947 | 5.11 | 3 | 4 | Frankia-8 | F201371.dat | 71 | 4 | 3 | 3 | 3 | 0 | 0 | 1 | 0 | 0 | 0.000% | 0 | 0 | 3 | 0 | 0 | 0.001% | 1.33 | 0.29 | 2.9E-01 | Red |
| WP_050996989.1 | FRAAL0617 | methyltransferase | 29880 | 5.05 | 7 | 48 | Frankia-10 | F201373.dat | 505 | 47 | 10 | 5 | 7 | 2 | 5 | 3 | 5 | 5 | 0.024% | 4 | 7 | 4 | 6 | 7 | 0.032% | 1.32 | 0.28 | 6.1E-02 | Red |
| WP_011603310.1 | FRAAL2145 | preprotein translocase subunit SecF | 43526 | 9.89 | 9 | 48 | Frankia-8 | F201371.dat | 407 | 24 | 8 | 8 | 9 | 4 | 4 | 4 | 4 | 4 | 0.016% | 5 | 5 | 9 | 4 | 5 | 0.022% | 1.32 | 0.28 | 6.2E-02 | Red |
| WP_011605942.1 | FRAAL4828 | stress protein | 19105 | 4.99 | 6 | 34 | Frankia-10 | F201373.dat | 212 | 47 | 5 | 5 | 5 | 2 | 3 | 4 | 3 | 2 | 0.026% | 2 | 4 | 5 | 4 | 5 | 0.035% | 1.32 | 0.27 | 5.4E-02 | Red |
| WP_041939923.1 | FRAAL6577 | potassium transporter | 78218 | 5.59 | 6 | 34 | Frankia-9 | F201372.dat | 171 | 6 | 4 | 4 | 5 | 3 | 1 | 3 | 3 | 4 | 0.006% | 3 | 5 | 5 | 5 | 2 | 0.009% | 1.32 | 0.27 | 8.6E-02 | Red |
| WP_011603140.1 | FRAAL1967 | MBL fold metallo-hydrolase | 25793 | 5.00 | 4 | 20 | Frankia-8 | F201371.dat | 159 | 21 | 3 | 3 | 3 | 2 | 2 | 2 | 1 | 1 | 0.011% | 2 | 2 | 3 | 2 | 3 | 0.016% | 1.31 | 0.27 | 6.3E-02 | Red |
| WP_011607075.1 | FRAAL6023 | FAD-dependent oxidoreductase | 51265 | 9.60 | 3 | 20 | Frankia-8 | F201371.dat | 94 | 5 | 3 | 3 | 3 | 1 | 2 | 1 | 2 | 2 | 0.006% | 2 | 3 | 3 | 3 | 1 | 0.008% | 1.31 | 0.27 | 8.1E-02 | Red |
| WP_041938698.1 | FRAAL0458 | subtype I-E CRISPR-associated endonuclease Cas1 | 36386 | 5.54 | 2 | 20 | Frankia-8 | F201371.dat | 74 | 7 | 2 | 2 | 3 | 1 | 1 | 2 | 2 | 2 | 0.008% | 2 | 1 | 3 | 3 | 3 | 0.011% | 1.31 | 0.27 | 8.1E-02 | Red |
| WP_011602674.1 | FRAAL1484 | ABC transporter | 29648 | 6.11 | 5 | 36 | Frankia-8 | F201371.dat | 294 | 32 | 5 | 5 | 6 | 3 | 4 | 2 | 2 | 4 | 0.018% | 3 | 4 | 6 | 4 | 4 | 0.024% | 1.30 | 0.26 | 5.4E-02 | Red |
| WP_041938953.1 | FRAAL1618 | hypothetical protein | 17167 | 9.98 | 5 | 36 | Frankia-8 | F201371.dat | 144 | 41 | 5 | 5 | 5 | 2 | 2 | 4 | 2 | 5 | 0.031% | 5 | 4 | 5 | 4 | 3 | 0.041% | 1.30 | 0.26 | 7.1E-02 | Red |
| WP_050997246.1 | FRAAL5715 | amino acid ABC transporter permease | 22861 | 9.60 | 4 | 13 | Frankia-9 | F201372.dat | 123 | 12 | 2 | 2 | 2 | 1 | 1 | 1 | 1 | 1 | 0.008% | 2 | 1 | 2 | 2 | 1 | 0.012% | 1.30 | 0.26 | 1.2E-01 | Red |
| WP_041939905.1 | FRAAL6470 | hypothetical protein | 38845 | 4.78 | 3 | 13 | Frankia-8 | F201371.dat | 158 | 9 | 2 | 2 | 2 | 2 | 1 | 1 | 1 | 0 | 0.005% | 1 | 3 | 2 | 1 | 1 | 0.007% | 1.30 | 0.26 | 1.4E-01 | Red |
| WP_041939972.1 | FRAAL6756 | para-aminobenzoate synthase | 23058 | 5.10 | 3 | 13 | Frankia-10 | F201373.dat | 124 | 15 | 2 | 2 | 2 | 1 | 1 | 1 | 1 | 1 | 0.008% | 2 | 2 | 0 | 2 | 2 | 0.012% | 1.30 | 0.26 | 1.4E-01 | Red |
| WP_011604940.1 | FRAAL3803 | sulfotransferase | 34937 | 7.86 | 3 | 13 | Frankia-10 | F201373.dat | 89 | 8 | 2 | 2 | 2 | 0 | 1 | 2 | 0 | 2 | 0.005% | 2 | 1 | 1 | 2 | 2 | 0.008% | 1.30 | 0.26 | 1.6E-01 | Red |
| WP_011602101.1 | FRAAL0868 | TetR family transcriptional regulator | 24373 | 5.19 | 3 | 13 | Frankia-9 | F201372.dat | 112 | 11 | 2 | 2 | 2 | 1 | 2 | 0 | 1 | 1 | 0.007% | 1 | 0 | 2 | 2 | 3 | 0.011% | 1.30 | 0.26 | 1.8E-01 | Red |
| WP_011603593.1 | FRAAL2435 | FMN-dependent NADH-azoreductase | 23439 | 6.49 | 5 | 13 | Frankia-8 | F201371.dat | 89 | 25 | 3 | 3 | 3 | 2 | 0 | 1 | 0 | 2 | 0.008% | 1 | 1 | 3 | 0 | 3 | 0.011% | 1.30 | 0.26 | 2.2E-01 | Red |
| WP_041940067.1 | FRAAL0401 | nitroreductase | 22508 | 7.82 | 8 | 91 | Frankia-8 | F201371.dat | 369 | 31 | 7 | 7 | 13 | 6 | 8 | 12 | 6 | 7 | 0.062% | 9 | 8 | 13 | 13 | 9 | 0.078% | 1.30 | 0.26 | 6.6E-02 | Red |
| WP_011606756.1 | FRAAL5682 | polyphosphate glucokinase | 29722 | 5.98 | 7 | 61 | Frankia-8 | F201371.dat | 283 | 17 | 5 | 5 | 10 | 5 | 6 | 6 | 4 | 5 | 0.031% | 5 | 8 | 10 | 7 | 5 | 0.040% | 1.29 | 0.25 | 5.8E-02 | Red |
| WP_011607772.1 | FRAAL6735 | 1.4-dihydroxy-6-naphthoate synthase | 29938 | 4.87 | 4 | 22 | Frankia-5 | F201368.dat | 100 | 11 | 3 | 3 | 3 | 1 | 2 | 2 | 1 | 3 | 0.011% | 1 | 3 | 3 | 3 | 3 | 0.015% | 1.29 | 0.25 | 9.1E-02 | Red |
| WP_011607354.1 | FRAAL6306 | hypothetical protein | 83959 | 10.48 | 2 | 22 | Frankia-4 | F201367.dat | 89 | 4 | 2 | 2 | 3 | 0 | 2 | 2 | 3 | 2 | 0.004% | 3 | 2 | 2 | 3 | 3 | 0.005% | 1.29 | 0.25 | 1.1E-01 | Red |
| WP_041938799.1 | FRAAL0884 | acyl-CoA hydratase | 29582 | 5.33 | 9 | 38 | Frankia-8 | F201371.dat | 337 | 32 | 7 | 7 | 8 | 1 | 4 | 5 | 2 | 4 | 0.019% | 4 | 2 | 8 | 3 | 5 | 0.025% | 1.29 | 0.25 | 1.9E-01 | Red |
| WP_041939560.1 | FRAAL4719 | mannose-6-phosphate isomerase | 17353 | 5.74 | 2 | 6 | Frankia-7 | F201370.dat | 83 | 13 | 1 | 1 | 1 | 0 | 1 | 1 | 0 | 0 | 0.004% | 0 | 1 | 1 | 1 | 1 | 0.008% | 1.29 | 0.25 | 2.1E-01 | Red |
| WP_041940258.1 | FRAAL1637 | alcohol dehydrogenase | 35219 | 5.85 | 3 | 6 | Frankia-8 | F201371.dat | 89 | 10 | 2 | 2 | 2 | 1 | 0 | 0 | 0 | 1 | 0.002% | 0 | 1 | 2 | 1 | 0 | 0.004% | 1.29 | 0.25 | 2.2E-01 | Red |
| WP_011604001.1 | FRAAL2855 | hypothetical protein | 18890 | 6.09 | 2 | 6 | Frankia-10 | F201373.dat | 81 | 12 | 2 | 2 | 2 | 0 | 1 | 0 | 0 | 1 | 0.004% | 0 | 1 | 0 | 1 | 2 | 0.007% | 1.29 | 0.25 | 2.2E-01 | Red |
| WP_041940214.1 | FRAAL1354 | transcriptional regulator | 39316 | 9.32 | 4 | 6 | Frankia-7 | F201370.dat | 123 | 12 | 3 | 3 | 3 | 1 | 0 | 0 | 0 | 1 | 0.002% | 0 | 3 | 0 | 0 | 1 | 0.003% | 1.29 | 0.25 | 2.8E-01 | Red |
| WP_011606005.1 | FRAAL4891 | oxidoreductase | 31221 | 4.99 | 3 | 6 | Frankia-8 | F201371.dat | 161 | 13 | 3 | 3 | 3 | 0 | 0 | 1 | 1 | 0 | 0.002% | 0 | 0 | 3 | 0 | 1 | 0.004% | 1.29 | 0.25 | 2.8E-01 | Red |
| WP_011607275.1 | FRAAL6225 | PPOX class F420-dependent enzyme | 15068 | 5.65 | 6 | 63 | Frankia-8 | F201371.dat | 316 | 46 | 5 | 5 | 9 | 5 | 3 | 6 | 7 | 6 | 0.064% | 6 | 4 | 9 | 7 | 10 | 0.080% | 1.28 | 0.25 | 9.6E-02 | Red |
| WP_011602451.1 | FRAAL1250 | ubiquinone biosynthesis protein | 25738 | 9.29 | 6 | 47 | Frankia-10 | F201373.dat | 232 | 22 | 5 | 5 | 7 | 3 | 5 | 4 | 3 | 5 | 0.028% | 3 | 6 | 6 | 5 | 7 | 0.035% | 1.28 | 0.25 | 6.2E-02 | Red |
| WP_041938808.1 | FRAAL0912 | 3-dehydroquinate dehydratase | 15842 | 5.28 | 4 | 31 | Frankia-8 | F201371.dat | 255 | 49 | 4 | 4 | 5 | 3 | 3 | 2 | 3 | 2 | 0.029% | 3 | 2 | 5 | 4 | 4 | 0.038% | 1.28 | 0.25 | 6.9E-02 | Red |
| WP_011606184.1 | FRAAL5075 | thiamine-phosphate pyrophosphorylase | 23279 | 6.43 | 5 | 31 | Frankia-10 | F201373.dat | 276 | 32 | 5 | 5 | 6 | 1 | 3 | 3 | 3 | 3 | 0.020% | 2 | 3 | 4 | 3 | 6 | 0.026% | 1.28 | 0.25 | 1.2E-01 | Red |
| WP_011601760.1 | FRAAL0507 | aminotransferase DegT | 39551 | 5.61 | 8 | 31 | Frankia-8 | F201371.dat | 238 | 25 | 6 | 6 | 6 | 1 | 4 | 3 | 2 | 3 | 0.012% | 4 | 3 | 6 | 4 | 1 | 0.015% | 1.28 | 0.25 | 1.6E-01 | Red |
| WP_011603181.1 | FRAAL2009 | phosphate starvation protein PhoH | 37855 | 6.32 | 7 | 56 | Frankia-5 | F201368.dat | 321 | 27 | 5 | 5 | 7 | 5 | 4 | 3 | 5 | 7 | 0.023% | 8 | 5 | 7 | 7 | 5 | 0.028% | 1.28 | 0.24 | 5.6E-02 | Red |
| WP_011607326.1 | FRAAL6277 | glycerophosphoryl diester phosphodiesterase | 69286 | 5.81 | 9 | 56 | Frankia-3 | F201366.dat | 444 | 14 | 7 | 7 | 8 | 4 | 3 | 8 | 5 | 4 | 0.012% | 6 | 8 | 7 | 5 | 6 | 0.016% | 1.28 | 0.24 | 7.4E-02 | Red |
| WP_011601712.1 | FRAAL0456 | CRISPR-associated protein Cas5 | 29833 | 5.87 | 5 | 15 | Frankia-10 | F201373.dat | 119 | 11 | 3 | 3 | 3 | 2 | 0 | 1 | 1 | 2 | 0.007% | 0 | 2 | 2 | 2 | 3 | 0.010% | 1.27 | 0.24 | 1.8E-01 | Red |
| WP_011605346.1 | FRAAL4219 | Scramblase | 32680 | 6.66 | 10 | 58 | Frankia-3 | F201366.dat | 398 | 43 | 8 | 8 | 8 | 4 | 5 | 8 | 5 | 3 | 0.027% | 7 | 6 | 6 | 7 | 7 | 0.034% | 1.27 | 0.24 | 5.7E-02 | Red |
| WP_009741388.1 | FRAAL0085 | transcription regulator of the Arc/MetJ class | 12343 | 6.39 | 3 | 24 | Frankia-10 | F201373.dat | 139 | 40 | 3 | 3 | 4 | 2 | 2 | 2 | 2 | 2 | 0.029% | 2 | 2 | 3 | 3 | 4 | 0.038% | 1.27 | 0.24 | 7.4E-02 | Red |
| WP_011607461.1 | FRAAL6416 | anti-sigma factor | 30144 | 6.93 | 3 | 24 | Frankia-9 | F201372.dat | 114 | 20 | 3 | 3 | 4 | 2 | 2 | 1 | 2 | 3 | 0.012% | 2 | 2 | 3 | 4 | 3 | 0.016% | 1.27 | 0.24 | 7.4E-02 | Red |
| WP_050997384.1 | FRAAL3562 | transcriptional regulator | 21154 | 7.77 | 5 | 33 | Frankia-7 | F201370.dat | 218 | 34 | 5 | 5 | 6 | 2 | 3 | 3 | 3 | 3 | 0.024% | 1 | 6 | 5 | 4 | 3 | 0.030% | 1.26 | 0.23 | 1.5E-01 | Red |
| WP_041939532.1 | FRAAL4552 | glycosyl transferase family 1 | 41845 | 5.34 | 12 | 69 | Frankia-7 | F201370.dat | 415 | 33 | 10 | 10 | 10 | 7 | 5 | 5 | 6 | 7 | 0.026% | 5 | 10 | 9 | 7 | 8 | 0.031% | 1.26 | 0.23 | 5.0E-02 | Red |
| WP_011601619.1 | FRAAL0362 | serine phosphatase | 38612 | 6.00 | 7 | 44 | Frankia-8 | F201371.dat | 298 | 18 | 6 | 6 | 6 | 4 | 5 | 5 | 3 | 2 | 0.018% | 5 | 6 | 6 | 4 | 4 | 0.022% | 1.25 | 0.22 | 7.1E-02 | Red |
| WP_011607246.1 | FRAAL6196 | hypothetical protein | 25408 | 5.72 | 4 | 26 | Frankia-7 | F201370.dat | 161 | 25 | 4 | 4 | 4 | 2 | 1 | 2 | 3 | 3 | 0.015% | 2 | 4 | 3 | 3 | 3 | 0.020% | 1.25 | 0.22 | 7.4E-02 | Red |
| WP_011604025.1 | FRAAL2879 | peptidylprolyl isomerase | 20881 | 9.17 | 3 | 35 | Frankia-10 | F201373.dat | 221 | 32 | 3 | 3 | 4 | 3 | 4 | 2 | 4 | 2 | 0.026% | 3 | 5 | 3 | 5 | 4 | 0.032% | 1.25 | 0.22 | 7.6E-02 | Red |
| WP_041939242.1 | FRAAL2891 | aminoglycoside phosphotransferase | 38575 | 4.93 | 4 | 26 | Frankia-8 | F201371.dat | 164 | 10 | 3 | 3 | 3 | 2 | 1 | 2 | 2 | 4 | 0.010% | 2 | 3 | 3 | 4 | 3 | 0.013% | 1.25 | 0.22 | 1.1E-01 | Red |
| WP_050997212.1 | FRAAL4955 | hypothetical protein | 46465 | 8.63 | 4 | 17 | Frankia-6 | F201369.dat | 76 | 7 | 3 | 3 | 3 | 1 | 2 | 1 | 2 | 1 | 0.005% | 3 | 2 | 2 | 2 | 1 | 0.007% | 1.25 | 0.22 | 1.2E-01 | Red |
| WP_011607109.1 | FRAAL6057 | hypothetical protein | 24317 | 5.07 | 3 | 17 | Frankia-8 | F201371.dat | 145 | 17 | 3 | 3 | 3 | 2 | 1 | 2 | 1 | 1 | 0.010% | 2 | 2 | 3 | 1 | 2 | 0.014% | 1.25 | 0.22 | 1.2E-01 | Red |
| WP_011605756.1 | FRAAL4640 | L-alanine:N-amidino-3-keto-scyllo-inosamine aminotransferase | 44873 | 6.02 | 14 | 80 | Frankia-8 | F201371.dat | 724 | 43 | 12 | 12 | 14 | 5 | 7 | 8 | 7 | 8 | 0.028% | 6 | 10 | 14 | 9 | 6 | 0.034% | 1.25 | 0.22 | 1.2E-01 | Red |
| WP_011603252.1 | FRAAL2086 | DNA-binding protein | 72830 | 10.01 | 4 | 17 | Frankia-1 | F201364.dat | 125 | 6 | 3 | 3 | 3 | 3 | 1 | 1 | 1 | 1 | 0.003% | 2 | 2 | 2 | 2 | 2 | 0.005% | 1.25 | 0.22 | 1.4E-01 | Red |
| WP_011601520.1 | FRAAL0261 | type IV restriction endonuclease | 44502 | 7.77 | 2 | 17 | Frankia-10 | F201373.dat | 157 | 10 | 2 | 2 | 2 | 2 | 1 | 2 | 0 | 2 | 0.006% | 2 | 2 | 2 | 2 | 2 | 0.008% | 1.25 | 0.22 | 1.4E-01 | Red |
| WP_011603144.1 | FRAAL1971 | apolipoprotein N- acyltransferase | 14981 | 10.87 | 2 | 17 | Frankia-5 | F201368.dat | 93 | 22 | 2 | 2 | 2 | 0 | 2 | 1 | 2 | 2 | 0.017% | 2 | 2 | 2 | 2 | 2 | 0.022% | 1.25 | 0.22 | 1.4E-01 | Red |
| WP_009741775.1 | FRAAL6525 | CarD family transcriptional regulator | 17735 | 5.49 | 9 | 53 | Frankia-9 | F201372.dat | 337 | 51 | 7 | 7 | 9 | 3 | 4 | 3 | 8 | 5 | 0.046% | 4 | 4 | 6 | 9 | 7 | 0.057% | 1.25 | 0.22 | 1.6E-01 | Red |
| WP_011603562.1 | FRAAL2402 | cold-shock protein | 14840 | 4.72 | 3 | 17 | Frankia-10 | F201373.dat | 179 | 42 | 3 | 3 | 4 | 2 | 1 | 2 | 1 | 1 | 0.017% | 2 | 1 | 2 | 1 | 4 | 0.023% | 1.25 | 0.22 | 1.9E-01 | Red |
| WP_041939387.1 | FRAAL3739 | methionine aminopeptidase | 26532 | 4.92 | 5 | 26 | Frankia-5 | F201368.dat | 203 | 20 | 4 | 4 | 4 | 2 | 1 | 1 | 3 | 4 | 0.015% | 2 | 2 | 4 | 2 | 5 | 0.019% | 1.25 | 0.22 | 1.9E-01 | Red |
| WP_011606246.1 | FRAAL5141 | nicotinate-nucleotide--dimethylbenzimidazole phosphoribosyltransferase | 38590 | 5.11 | 9 | 44 | Frankia-10 | F201373.dat | 301 | 29 | 7 | 7 | 7 | 3 | 3 | 5 | 5 | 3 | 0.018% | 0 | 6 | 6 | 6 | 7 | 0.022% | 1.25 | 0.22 | 2.0E-01 | Red |
| WP_011604048.1 | FRAAL2902 | haloacid dehalogenase | 24985 | 6.51 | 2 | 8 | Frankia-8 | F201371.dat | 81 | 9 | 2 | 2 | 2 | 0 | 1 | 0 | 1 | 1 | 0.004% | 0 | 1 | 2 | 1 | 1 | 0.007% | 1.25 | 0.22 | 2.1E-01 | Red |
| WP_011602510.1 | FRAAL1312 | chromosome partitioning protein | 45496 | 5.53 | 2 | 8 | Frankia-2 | F201365.dat | 71 | 2 | 1 | 1 | 1 | 1 | 1 | 1 | 0 | 0 | 0.002% | 2 | 1 | 0 | 1 | 1 | 0.004% | 1.25 | 0.22 | 2.1E-01 | Red |
| WP_041938901.1 | FRAAL1382 | hypothetical protein | 34453 | 5.41 | 4 | 17 | Frankia-10 | F201373.dat | 123 | 21 | 4 | 4 | 4 | 2 | 2 | 0 | 3 | 0 | 0.007% | 1 | 1 | 3 | 1 | 4 | 0.010% | 1.25 | 0.22 | 2.6E-01 | Red |
| WP_041939051.1 | FRAAL2098 | 3'-5' exonuclease | 54451 | 6.24 | 2 | 8 | Frankia-10 | F201373.dat | 85 | 4 | 2 | 2 | 2 | 0 | 0 | 2 | 0 | 1 | 0.002% | 1 | 0 | 2 | 0 | 2 | 0.003% | 1.25 | 0.22 | 2.6E-01 | Red |
| WP_041939807.1 | FRAAL3248 | hypothetical protein | 7528 | 6.09 | 2 | 8 | Frankia-9 | F201372.dat | 73 | 26 | 2 | 2 | 2 | 0 | 2 | 0 | 1 | 0 | 0.014% | 0 | 0 | 1 | 2 | 2 | 0.022% | 1.25 | 0.22 | 2.6E-01 | Red |
| WP_011605841.1 | FRAAL4726 | aldo/keto reductase | 35628 | 5.51 | 3 | 8 | Frankia-10 | F201373.dat | 200 | 18 | 5 | 3 | 3 | 1 | 1 | 0 | 0 | 1 | 0.003% | 1 | 1 | 0 | 0 | 3 | 0.005% | 1.25 | 0.22 | 2.7E-01 | Red |
| WP_011602555.1 | FRAAL1358 | hypothetical protein | 38228 | 8.21 | 5 | 17 | Frankia-8 | F201371.dat | 94 | 14 | 4 | 4 | 4 | 2 | 2 | 0 | 3 | 0 | 0.007% | 0 | 0 | 4 | 3 | 3 | 0.009% | 1.25 | 0.22 | 2.9E-01 | Red |
| WP_050997432.1 | FRAAL5206 | acetylglutamate kinase | 31951 | 4.92 | 12 | 91 | Frankia-8 | F201371.dat | 686 | 56 | 11 | 11 | 14 | 10 | 6 | 11 | 7 | 6 | 0.045% | 9 | 9 | 14 | 10 | 9 | 0.054% | 1.24 | 0.22 | 8.1E-02 | Red |
| WP_009740504.1 | FRAAL1107 | 30S ribosomal protein S13 | 14578 | 11.02 | 16 | 185 | Frankia-10 | F201373.dat | 630 | 77 | 14 | 14 | 27 | 15 | 16 | 15 | 17 | 19 | 0.201% | 14 | 18 | 22 | 22 | 27 | 0.238% | 1.24 | 0.22 | 5.3E-02 | Red |
| WP_041939779.1 | FRAAL5899 | alpha-1.4-glucan--maltose-1-phosphate maltosyltransferase | 75435 | 5.35 | 19 | 111 | Frankia-8 | F201371.dat | 868 | 30 | 16 | 16 | 20 | 12 | 7 | 9 | 11 | 10 | 0.023% | 9 | 10 | 20 | 11 | 12 | 0.028% | 1.24 | 0.22 | 1.3E-01 | Red |
| WP_011602227.1 | FRAAL1000 | 3-oxoacyl-ACP reductase | 25643 | 5.06 | 7 | 46 | Frankia-9 | F201372.dat | 331 | 37 | 6 | 6 | 6 | 5 | 3 | 3 | 4 | 5 | 0.028% | 5 | 3 | 5 | 6 | 7 | 0.034% | 1.24 | 0.22 | 8.6E-02 | Red |
| WP_041938929.1 | FRAAL1502 | purine NTP phosphatase | 21838 | 5.09 | 6 | 28 | Frankia-10 | F201373.dat | 199 | 29 | 4 | 4 | 4 | 2 | 2 | 1 | 3 | 4 | 0.020% | 2 | 2 | 3 | 5 | 4 | 0.025% | 1.24 | 0.21 | 1.7E-01 | Red |
| WP_011607104.1 | FRAAL6052 | DNA repair protein | 39459 | 5.69 | 14 | 57 | Frankia-8 | F201371.dat | 510 | 39 | 10 | 10 | 11 | 4 | 3 | 6 | 7 | 5 | 0.023% | 5 | 5 | 11 | 4 | 7 | 0.027% | 1.23 | 0.21 | 1.8E-01 | Red |
| WP_011606944.1 | FRAAL5883 | glycogen-debranching protein | 81606 | 6.27 | 11 | 48 | Frankia-8 | F201371.dat | 304 | 15 | 8 | 8 | 8 | 5 | 4 | 5 | 4 | 3 | 0.009% | 3 | 6 | 8 | 6 | 4 | 0.011% | 1.23 | 0.21 | 1.2E-01 | Red |
| WP_011605306.1 | FRAAL4178 | hypothetical protein | 46222 | 6.43 | 6 | 19 | Frankia-1 | F201364.dat | 165 | 11 | 3 | 3 | 3 | 3 | 0 | 2 | 2 | 1 | 0.006% | 2 | 3 | 2 | 2 | 2 | 0.008% | 1.23 | 0.21 | 1.8E-01 | Red |
| WP_041940527.1 | FRAAL3251 | NAD synthetase | 73380 | 5.80 | 12 | 68 | Frankia-6 | F201369.dat | 519 | 16 | 7 | 7 | 9 | 6 | 7 | 5 | 5 | 7 | 0.015% | 9 | 9 | 5 | 7 | 8 | 0.017% | 1.23 | 0.21 | 5.2E-02 | Red |
| WP_011602448.1 | FRAAL1247 | acetyltransferase | 22348 | 9.61 | 9 | 88 | Frankia-7 | F201370.dat | 332 | 44 | 8 | 8 | 12 | 6 | 7 | 11 | 8 | 7 | 0.062% | 10 | 12 | 8 | 9 | 10 | 0.074% | 1.23 | 0.20 | 5.1E-02 | Red |
| WP_041938837.1 | FRAAL1014 | XRE family transcriptional regulator | 40855 | 6.68 | 6 | 39 | Frankia-8 | F201371.dat | 302 | 21 | 5 | 5 | 5 | 4 | 4 | 4 | 3 | 2 | 0.015% | 5 | 5 | 5 | 3 | 4 | 0.018% | 1.23 | 0.20 | 5.8E-02 | Red |
| WP_041939044.1 | FRAAL2076 | MFS transporter | 48739 | 10.44 | 4 | 39 | Frankia-6 | F201369.dat | 201 | 9 | 4 | 4 | 5 | 3 | 3 | 5 | 4 | 2 | 0.012% | 5 | 3 | 5 | 4 | 5 | 0.015% | 1.23 | 0.20 | 8.1E-02 | Red |
| WP_011605172.1 | FRAAL4041 | (2Fe-2S)-binding protein | 32195 | 9.70 | 6 | 30 | Frankia-7 | F201370.dat | 216 | 21 | 4 | 4 | 4 | 2 | 3 | 2 | 3 | 3 | 0.014% | 4 | 4 | 4 | 2 | 3 | 0.018% | 1.22 | 0.20 | 8.1E-02 | Red |
| WP_011604011.1 | FRAAL2865 | exonuclease RecB | 35597 | 6.02 | 5 | 30 | Frankia-9 | F201372.dat | 235 | 20 | 5 | 5 | 5 | 3 | 2 | 3 | 2 | 3 | 0.013% | 2 | 3 | 4 | 5 | 3 | 0.016% | 1.22 | 0.20 | 1.1E-01 | Red |
| WP_011606929.1 | FRAAL5861 | tRNA-specific 2-thiouridylase MnmA | 37251 | 6.08 | 9 | 30 | Frankia-8 | F201371.dat | 150 | 16 | 5 | 5 | 5 | 2 | 2 | 4 | 3 | 2 | 0.012% | 1 | 3 | 5 | 4 | 4 | 0.015% | 1.22 | 0.20 | 1.7E-01 | Red |
| WP_011607786.1 | FRAAL6750 | hypothetical protein | 17976 | 10.83 | 2 | 10 | Frankia-7 | F201370.dat | 118 | 19 | 2 | 2 | 2 | 1 | 0 | 1 | 1 | 1 | 0.008% | 1 | 2 | 1 | 1 | 1 | 0.011% | 1.22 | 0.20 | 2.1E-01 | Red |
| WP_041939910.1 | FRAAL6518 | serine/threonine protein phosphatase | 38615 | 4.49 | 2 | 10 | Frankia-6 | F201369.dat | 111 | 5 | 2 | 2 | 2 | 1 | 1 | 0 | 1 | 1 | 0.004% | 2 | 1 | 1 | 1 | 1 | 0.005% | 1.22 | 0.20 | 2.1E-01 | Red |
| WP_041939788.1 | FRAAL5954 | histidine kinase | 14062 | 4.41 | 2 | 10 | Frankia-9 | F201372.dat | 84 | 14 | 2 | 2 | 2 | 1 | 0 | 1 | 1 | 1 | 0.010% | 1 | 1 | 1 | 2 | 1 | 0.014% | 1.22 | 0.20 | 2.1E-01 | Red |
| WP_041939511.1 | FRAAL4461 | daunorubicin resistance protein DrrA family ABC transporter ATP-binding protein | 36831 | 4.74 | 2 | 10 | Frankia-8 | F201371.dat | 76 | 8 | 2 | 2 | 2 | 1 | 0 | 2 | 1 | 0 | 0.004% | 1 | 1 | 2 | 2 | 0 | 0.005% | 1.22 | 0.20 | 2.4E-01 | Red |
| WP_041940712.1 | FRAAL4168 | iron ABC transporter | 30586 | 5.00 | 6 | 30 | Frankia-8 | F201371.dat | 203 | 24 | 6 | 6 | 6 | 3 | 2 | 2 | 3 | 3 | 0.015% | 0 | 2 | 6 | 5 | 4 | 0.019% | 1.22 | 0.20 | 2.5E-01 | Red |
| WP_041938841.1 | FRAAL1036 | NADH dehydrogenase | 26112 | 4.59 | 2 | 10 | Frankia-8 | F201371.dat | 125 | 20 | 2 | 2 | 3 | 1 | 1 | 1 | 1 | 0 | 0.005% | 0 | 1 | 3 | 0 | 2 | 0.008% | 1.22 | 0.20 | 2.8E-01 | Red |
| WP_050997375.1 | FRAAL3284 | esterase | 34975 | 4.47 | 2 | 10 | Frankia-3 | F201366.dat | 103 | 7 | 2 | 2 | 3 | 1 | 0 | 3 | 0 | 0 | 0.004% | 1 | 2 | 1 | 2 | 0 | 0.006% | 1.22 | 0.20 | 2.9E-01 | Red |
| WP_041938909.1 | FRAAL1411 | initiation factor 2B subunit alpha | 34137 | 5.22 | 2 | 10 | Frankia-8 | F201371.dat | 96 | 5 | 2 | 2 | 2 | 0 | 0 | 2 | 2 | 0 | 0.004% | 2 | 0 | 2 | 0 | 2 | 0.006% | 1.22 | 0.20 | 2.9E-01 | Red |
| WP_011606080.1 | FRAAL4966 | prolipoprotein diacylglyceryl transferase | 39857 | 6.39 | 3 | 10 | Frankia-10 | F201373.dat | 59 | 6 | 2 | 2 | 3 | 2 | 0 | 2 | 0 | 0 | 0.004% | 1 | 0 | 2 | 0 | 3 | 0.005% | 1.22 | 0.20 | 3.1E-01 | Red |
| WP_041940554.1 | FRAAL3372 | hypothetical protein | 19744 | 5.67 | 4 | 10 | Frankia-10 | F201373.dat | 146 | 37 | 4 | 4 | 4 | 0 | 0 | 1 | 1 | 2 | 0.007% | 0 | 0 | 2 | 0 | 4 | 0.010% | 1.22 | 0.20 | 3.3E-01 | Red |
| WP_011603060.1 | FRAAL1883 | exodeoxyribonuclease III | 32518 | 5.30 | 9 | 61 | Frankia-10 | F201373.dat | 407 | 30 | 8 | 8 | 10 | 5 | 5 | 7 | 6 | 4 | 0.030% | 3 | 7 | 8 | 6 | 10 | 0.035% | 1.22 | 0.20 | 1.5E-01 | Red |
| WP_041940596.1 | FRAAL3642 | 3-ketoacyl-ACP reductase | 29594 | 4.78 | 17 | 225 | Frankia-8 | F201371.dat | 898 | 68 | 16 | 16 | 32 | 20 | 20 | 21 | 18 | 22 | 0.122% | 17 | 24 | 32 | 25 | 26 | 0.141% | 1.22 | 0.20 | 5.1E-02 | Red |
| WP_041938692.1 | FRAAL0440 | hypothetical protein | 73917 | 5.98 | 16 | 72 | Frankia-10 | F201373.dat | 470 | 24 | 12 | 12 | 12 | 7 | 8 | 3 | 4 | 10 | 0.015% | 7 | 8 | 7 | 6 | 12 | 0.018% | 1.22 | 0.20 | 1.8E-01 | Red |
| WP_011602771.1 | FRAAL1584 | hypothetical protein | 48652 | 8.81 | 9 | 52 | Frankia-7 | F201370.dat | 336 | 28 | 7 | 7 | 7 | 6 | 3 | 6 | 4 | 4 | 0.017% | 5 | 7 | 6 | 5 | 6 | 0.020% | 1.21 | 0.19 | 6.4E-02 | Red |
| WP_011601940.1 | FRAAL0697 | carboxymethylenebutenolidase | 26356 | 4.71 | 7 | 52 | Frankia-8 | F201371.dat | 487 | 54 | 7 | 7 | 8 | 4 | 4 | 5 | 6 | 4 | 0.031% | 4 | 5 | 8 | 7 | 5 | 0.037% | 1.21 | 0.19 | 9.5E-02 | Red |
| WP_011603160.1 | FRAAL1987 | hypothetical protein | 19844 | 6.63 | 3 | 21 | Frankia-6 | F201369.dat | 118 | 20 | 3 | 3 | 3 | 2 | 2 | 2 | 3 | 0 | 0.016% | 3 | 1 | 3 | 2 | 3 | 0.020% | 1.21 | 0.19 | 1.9E-01 | Red |
| WP_011606260.1 | FRAAL5156 | lipoyltransferase | 26207 | 5.86 | 5 | 21 | Frankia-5 | F201368.dat | 130 | 27 | 4 | 4 | 4 | 2 | 0 | 0 | 3 | 4 | 0.012% | 0 | 4 | 4 | 0 | 4 | 0.015% | 1.21 | 0.19 | 3.2E-01 | Red |
| WP_011602737.1 | FRAAL1549 | beta-ketoacyl synthase | 284562 | 5.03 | 8 | 21 | Frankia-8 | F201371.dat | 218 | 3 | 6 | 6 | 6 | 6 | 3 | 0 | 0 | 0 | 0.001% | 4 | 0 | 6 | 2 | 0 | 0.001% | 1.21 | 0.19 | 3.6E-01 | Red |
| WP_011605165.1 | FRAAL4034 | short-chain dehydrogenase | 27205 | 5.24 | 9 | 63 | Frankia-8 | F201371.dat | 453 | 44 | 7 | 7 | 10 | 5 | 5 | 5 | 7 | 6 | 0.037% | 5 | 7 | 10 | 4 | 9 | 0.043% | 1.21 | 0.19 | 1.4E-01 | Red |
| WP_050997016.1 | FRAAL1276 | sugar dehydratase | 17627 | 4.39 | 4 | 32 | Frankia-9 | F201372.dat | 225 | 25 | 3 | 3 | 4 | 1 | 3 | 3 | 3 | 4 | 0.028% | 4 | 3 | 3 | 4 | 4 | 0.034% | 1.21 | 0.19 | 1.1E-01 | Red |
| WP_011603152.1 | FRAAL1979 | phosphoglycerate dehydrogenase | 32282 | 6.25 | 8 | 32 | Frankia-5 | F201368.dat | 166 | 20 | 4 | 4 | 4 | 3 | 3 | 3 | 1 | 4 | 0.015% | 3 | 5 | 3 | 4 | 3 | 0.019% | 1.21 | 0.19 | 1.2E-01 | Red |
| WP_011607061.1 | FRAAL6008 | UDP-glucose 4-epimerase | 32919 | 6.45 | 6 | 32 | Frankia-8 | F201371.dat | 198 | 23 | 5 | 5 | 5 | 3 | 1 | 2 | 4 | 4 | 0.015% | 3 | 3 | 5 | 3 | 4 | 0.018% | 1.21 | 0.19 | 1.5E-01 | Red |
| WP_041939873.1 | FRAAL6332 | helicase | 103454 | 5.41 | 7 | 32 | Frankia-10 | F201373.dat | 256 | 6 | 6 | 6 | 6 | 4 | 4 | 2 | 1 | 3 | 0.005% | 2 | 3 | 4 | 3 | 6 | 0.006% | 1.21 | 0.19 | 2.0E-01 | Red |
| WP_011601753.1 | FRAAL0500 | hypothetical protein | 19624 | 6.84 | 5 | 32 | Frankia-8 | F201371.dat | 243 | 31 | 4 | 4 | 6 | 4 | 3 | 3 | 2 | 2 | 0.025% | 2 | 5 | 6 | 3 | 2 | 0.031% | 1.21 | 0.19 | 2.0E-01 | Red |
| WP_011604498.1 | FRAAL3351 | serine acetyltransferase | 25768 | 7.18 | 4 | 32 | Frankia-9 | F201372.dat | 191 | 16 | 4 | 4 | 5 | 2 | 3 | 4 | 3 | 2 | 0.019% | 0 | 5 | 4 | 5 | 4 | 0.024% | 1.21 | 0.19 | 2.2E-01 | Red |
| WP_041939927.1 | FRAAL6584 | inorganic pyrophosphatase | 20541 | 4.85 | 15 | 202 | Frankia-8 | F201371.dat | 701 | 75 | 13 | 13 | 32 | 17 | 18 | 18 | 19 | 19 | 0.158% | 18 | 18 | 32 | 20 | 23 | 0.182% | 1.21 | 0.19 | 8.4E-02 | Red |
| WP_041939922.1 | FRAAL6575 | anti-sigma factor | 18524 | 4.52 | 6 | 43 | Frankia-8 | F201371.dat | 278 | 22 | 5 | 5 | 7 | 4 | 4 | 4 | 5 | 2 | 0.037% | 5 | 4 | 7 | 3 | 5 | 0.044% | 1.21 | 0.19 | 1.3E-01 | Red |
| WP_011603999.1 | FRAAL2853 | exodeoxyribonuclease V | 81320 | 4.85 | 11 | 54 | Frankia-9 | F201372.dat | 286 | 12 | 7 | 7 | 8 | 4 | 7 | 5 | 5 | 3 | 0.011% | 6 | 4 | 5 | 8 | 7 | 0.012% | 1.21 | 0.19 | 1.3E-01 | Red |
| WP_011605708.1 | FRAAL4592 | glmZ(sRNA)-inactivating NTPase | 31366 | 6.46 | 12 | 76 | Frankia-9 | F201372.dat | 296 | 29 | 8 | 8 | 10 | 7 | 8 | 8 | 6 | 5 | 0.039% | 5 | 10 | 10 | 10 | 7 | 0.045% | 1.21 | 0.19 | 1.1E-01 | Red |
| WP_041940887.1 | FRAAL5228 | phosphopantothenoylcysteine decarboxylase | 44860 | 6.53 | 18 | 109 | Frankia-8 | F201371.dat | 598 | 36 | 14 | 14 | 14 | 11 | 7 | 11 | 10 | 10 | 0.039% | 8 | 15 | 14 | 12 | 11 | 0.045% | 1.20 | 0.19 | 8.1E-02 | Red |
| WP_041939020.1 | FRAAL1925 | 50S ribosomal protein L21 | 11024 | 9.93 | 3 | 56 | Frankia-7 | F201370.dat | 181 | 20 | 3 | 3 | 5 | 4 | 5 | 4 | 6 | 6 | 0.081% | 7 | 5 | 5 | 6 | 8 | 0.095% | 1.20 | 0.18 | 7.1E-02 | Red |
| WP_050997248.1 | FRAAL5763 | ribosome maturation factor RimP | 26248 | 5.04 | 6 | 56 | Frankia-7 | F201370.dat | 406 | 35 | 6 | 6 | 7 | 5 | 4 | 6 | 7 | 3 | 0.034% | 5 | 7 | 7 | 5 | 7 | 0.040% | 1.20 | 0.18 | 1.0E-01 | Red |
| WP_011606860.1 | FRAAL5788 | signal peptidase | 43428 | 9.63 | 9 | 56 | Frankia-8 | F201371.dat | 376 | 25 | 7 | 7 | 8 | 4 | 5 | 6 | 5 | 5 | 0.021% | 4 | 8 | 8 | 6 | 5 | 0.024% | 1.20 | 0.18 | 1.0E-01 | Red |
| WP_011601887.1 | FRAAL0638 | cobalamin biosynthesis protein CobB | 27293 | 5.61 | 4 | 34 | Frankia-1 | F201364.dat | 184 | 23 | 4 | 4 | 4 | 4 | 4 | 2 | 3 | 2 | 0.020% | 3 | 4 | 4 | 3 | 5 | 0.023% | 1.20 | 0.18 | 1.0E-01 | Red |
| WP_011607510.1 | FRAAL6465 | hypothetical protein | 43866 | 9.96 | 3 | 23 | Frankia-9 | F201372.dat | 295 | 14 | 3 | 3 | 3 | 2 | 2 | 2 | 3 | 1 | 0.008% | 3 | 3 | 2 | 3 | 2 | 0.010% | 1.20 | 0.18 | 1.2E-01 | Red |
| WP_011606283.1 | FRAAL5181 | DNA repair protein RecN | 60853 | 4.87 | 10 | 34 | Frankia-10 | F201373.dat | 266 | 9 | 4 | 4 | 4 | 3 | 3 | 4 | 1 | 4 | 0.009% | 4 | 4 | 3 | 4 | 4 | 0.011% | 1.20 | 0.18 | 1.2E-01 | Red |
| WP_041938889.1 | FRAAL1295 | cell entry protein | 15053 | 5.81 | 4 | 23 | Frankia-8 | F201371.dat | 179 | 34 | 3 | 3 | 3 | 1 | 2 | 2 | 3 | 2 | 0.024% | 3 | 1 | 3 | 3 | 3 | 0.029% | 1.20 | 0.18 | 1.4E-01 | Red |
| WP_011602390.1 | FRAAL1187 | succinate dehydrogenase | 28484 | 5.75 | 3 | 12 | Frankia-9 | F201372.dat | 61 | 7 | 2 | 2 | 2 | 0 | 2 | 1 | 1 | 1 | 0.006% | 2 | 1 | 1 | 2 | 1 | 0.008% | 1.20 | 0.18 | 2.1E-01 | Red |
| WP_011606372.1 | FRAAL5281 | hypothetical protein | 47693 | 5.15 | 3 | 12 | Frankia-5 | F201368.dat | 54 | 6 | 2 | 2 | 2 | 1 | 1 | 0 | 1 | 2 | 0.004% | 1 | 1 | 1 | 2 | 2 | 0.005% | 1.20 | 0.18 | 2.1E-01 | Red |
| WP_041940467.1 | FRAAL2848 | membrane protein | 66754 | 10.08 | 10 | 45 | Frankia-2 | F201365.dat | 272 | 11 | 6 | 6 | 7 | 3 | 7 | 5 | 5 | 0 | 0.011% | 5 | 5 | 6 | 5 | 4 | 0.013% | 1.20 | 0.18 | 2.2E-01 | Red |
| WP_011602716.1 | FRAAL1526 | electron transporter SenC | 20772 | 4.94 | 2 | 12 | Frankia-7 | F201370.dat | 96 | 17 | 2 | 2 | 2 | 1 | 1 | 1 | 1 | 1 | 0.009% | 0 | 2 | 1 | 2 | 2 | 0.011% | 1.20 | 0.18 | 2.3E-01 | Red |
| WP_011601554.1 | FRAAL0296 | aminocarboxymuconate-semialdehyde decarboxylase | 43021 | 5.35 | 4 | 12 | Frankia-8 | F201371.dat | 128 | 13 | 3 | 3 | 3 | 1 | 1 | 1 | 0 | 2 | 0.004% | 0 | 1 | 3 | 1 | 2 | 0.005% | 1.20 | 0.18 | 2.6E-01 | Red |
| WP_009742309.1 | FRAAL5817 | AsnC family transcriptional regulator | 8307 | 4.85 | 3 | 12 | Frankia-9 | F201372.dat | 141 | 56 | 3 | 3 | 3 | 1 | 1 | 1 | 1 | 1 | 0.021% | 2 | 0 | 1 | 3 | 1 | 0.028% | 1.20 | 0.18 | 2.6E-01 | Red |
| WP_041939595.1 | FRAAL4941 | methyltransferase | 29512 | 4.83 | 3 | 12 | Frankia-8 | F201371.dat | 139 | 14 | 3 | 3 | 3 | 1 | 1 | 1 | 1 | 1 | 0.006% | 1 | 0 | 3 | 1 | 2 | 0.008% | 1.20 | 0.18 | 2.6E-01 | Red |
| WP_011603289.1 | FRAAL2124 | hypothetical protein | 28725 | 6.11 | 3 | 12 | Frankia-4 | F201367.dat | 115 | 10 | 2 | 2 | 3 | 0 | 0 | 0 | 3 | 2 | 0.006% | 1 | 1 | 2 | 1 | 2 | 0.008% | 1.20 | 0.18 | 3.0E-01 | Red |
| WP_041938915.1 | FRAAL1427 | short-chain dehydrogenase | 28391 | 10.56 | 11 | 91 | Frankia-8 | F201371.dat | 366 | 41 | 9 | 9 | 13 | 9 | 8 | 10 | 6 | 8 | 0.052% | 7 | 10 | 13 | 10 | 10 | 0.059% | 1.20 | 0.18 | 7.9E-02 | Red |
| WP_011602529.1 | FRAAL1332 | aminoglycoside phosphotransferase | 127096 | 6.05 | 14 | 58 | Frankia-8 | F201371.dat | 378 | 8 | 8 | 8 | 9 | 7 | 5 | 4 | 6 | 4 | 0.007% | 6 | 6 | 9 | 5 | 6 | 0.008% | 1.19 | 0.18 | 1.1E-01 | Red |
| WP_011605171.1 | FRAAL4040 | oxidoreductase | 22987 | 4.82 | 5 | 47 | Frankia-8 | F201371.dat | 289 | 32 | 5 | 5 | 9 | 3 | 3 | 5 | 4 | 6 | 0.033% | 3 | 5 | 9 | 4 | 5 | 0.038% | 1.19 | 0.18 | 2.1E-01 | Red |
| WP_011601330.1 | FRAAL0061 | nitroreductase | 22067 | 6.74 | 4 | 36 | Frankia-7 | F201370.dat | 207 | 32 | 4 | 4 | 5 | 2 | 4 | 3 | 3 | 4 | 0.026% | 3 | 5 | 4 | 4 | 4 | 0.031% | 1.19 | 0.17 | 7.4E-02 | Red |
| WP_011606281.1 | FRAAL5179 | CTP synthase | 66247 | 5.75 | 15 | 82 | Frankia-8 | F201371.dat | 361 | 18 | 10 | 10 | 11 | 8 | 7 | 6 | 7 | 9 | 0.020% | 8 | 6 | 11 | 10 | 10 | 0.023% | 1.19 | 0.17 | 7.9E-02 | Red |
| WP_041939503.1 | FRAAL4427 | 3-ketoacyl-ACP reductase | 28301 | 5.23 | 11 | 82 | Frankia-4 | F201367.dat | 373 | 43 | 8 | 8 | 11 | 7 | 8 | 6 | 11 | 5 | 0.047% | 8 | 8 | 12 | 10 | 7 | 0.054% | 1.19 | 0.17 | 1.4E-01 | Red |
| WP_011606122.1 | FRAAL5011 | short-chain dehydrogenase | 30174 | 4.99 | 11 | 82 | Frankia-8 | F201371.dat | 512 | 46 | 9 | 9 | 13 | 8 | 4 | 9 | 9 | 7 | 0.044% | 5 | 9 | 13 | 8 | 10 | 0.050% | 1.19 | 0.17 | 1.7E-01 | Red |
| WP_011601652.1 | FRAAL0396 | enoyl-CoA hydratase | 27568 | 5.74 | 6 | 36 | Frankia-8 | F201371.dat | 305 | 33 | 6 | 6 | 6 | 4 | 4 | 4 | 1 | 3 | 0.021% | 3 | 3 | 6 | 4 | 4 | 0.024% | 1.19 | 0.17 | 1.7E-01 | Red |
| WP_011606927.1 | FRAAL5859 | DNA ligase | 81377 | 5.17 | 14 | 71 | Frankia-1 | F201364.dat | 397 | 15 | 10 | 10 | 10 | 10 | 4 | 6 | 6 | 6 | 0.014% | 5 | 7 | 12 | 7 | 8 | 0.016% | 1.19 | 0.17 | 1.9E-01 | Red |
| WP_011607041.1 | FRAAL5986 | arginine ABC transporter substrate-binding protein | 24924 | 4.85 | 11 | 141 | Frankia-8 | F201371.dat | 770 | 60 | 10 | 10 | 19 | 12 | 11 | 12 | 16 | 13 | 0.092% | 11 | 16 | 19 | 16 | 15 | 0.104% | 1.19 | 0.17 | 6.6E-02 | Red |
| WP_041938828.1 | FRAAL0979 | hydrolase | 31612 | 6.67 | 14 | 95 | Frankia-3 | F201366.dat | 606 | 53 | 11 | 11 | 12 | 6 | 8 | 12 | 7 | 10 | 0.049% | 9 | 10 | 12 | 9 | 12 | 0.055% | 1.19 | 0.17 | 9.8E-02 | Red |
| WP_041938732.1 | FRAAL0602 | aldo/keto reductase | 35386 | 5.17 | 9 | 60 | Frankia-8 | F201371.dat | 255 | 27 | 6 | 6 | 8 | 6 | 7 | 4 | 5 | 5 | 0.027% | 7 | 8 | 8 | 4 | 6 | 0.031% | 1.19 | 0.17 | 1.1E-01 | Red |
| WP_050997315.1 | FRAAL0777 | FMN reductase | 17382 | 5.06 | 3 | 25 | Frankia-5 | F201368.dat | 145 | 14 | 3 | 3 | 3 | 2 | 2 | 2 | 2 | 3 | 0.023% | 3 | 2 | 2 | 3 | 4 | 0.027% | 1.19 | 0.17 | 1.3E-01 | Red |
| WP_041939221.1 | FRAAL2794 | N-acetylmuramoyl-L-alanine amidase | 15461 | 8.20 | 8 | 202 | Frankia-10 | F201373.dat | 542 | 81 | 8 | 8 | 24 | 11 | 19 | 22 | 20 | 20 | 0.212% | 16 | 21 | 23 | 26 | 24 | 0.240% | 1.19 | 0.17 | 9.9E-02 | Red |
| WP_011603725.1 | FRAAL2568 | alpha/beta hydrolase | 29022 | 8.67 | 6 | 49 | Frankia-10 | F201373.dat | 207 | 17 | 5 | 5 | 5 | 3 | 6 | 6 | 3 | 4 | 0.027% | 5 | 5 | 6 | 6 | 5 | 0.031% | 1.19 | 0.17 | 1.1E-01 | Red |
| WP_011603239.1 | FRAAL2073 | lyase | 15004 | 4.50 | 3 | 14 | Frankia-7 | F201370.dat | 114 | 18 | 2 | 2 | 2 | 1 | 1 | 1 | 1 | 2 | 0.014% | 1 | 2 | 1 | 2 | 2 | 0.018% | 1.18 | 0.17 | 2.1E-01 | Red |
| WP_050997355.1 | FRAAL2447 | metal-binding protein | 53854 | 10.33 | 3 | 14 | Frankia-8 | F201371.dat | 108 | 6 | 2 | 2 | 2 | 1 | 1 | 2 | 1 | 1 | 0.004% | 2 | 1 | 2 | 1 | 2 | 0.005% | 1.18 | 0.17 | 2.1E-01 | Red |
| WP_041939137.1 | FRAAL2476 | hypothetical protein | 19778 | 5.24 | 3 | 14 | Frankia-7 | F201370.dat | 107 | 12 | 2 | 2 | 2 | 1 | 1 | 1 | 1 | 2 | 0.011% | 2 | 2 | 1 | 1 | 2 | 0.014% | 1.18 | 0.17 | 2.1E-01 | Red |
| WP_041940167.1 | FRAAL1060 | hypothetical protein | 16231 | 5.54 | 3 | 14 | Frankia-5 | F201368.dat | 128 | 31 | 3 | 3 | 3 | 1 | 1 | 0 | 1 | 3 | 0.013% | 2 | 2 | 1 | 1 | 2 | 0.017% | 1.18 | 0.17 | 2.6E-01 | Red |
| WP_011604156.1 | FRAAL3006 | metallophosphatase | 30176 | 4.76 | 5 | 14 | Frankia-8 | F201371.dat | 147 | 18 | 3 | 3 | 3 | 1 | 1 | 2 | 1 | 1 | 0.007% | 0 | 1 | 3 | 2 | 2 | 0.009% | 1.18 | 0.17 | 2.6E-01 | Red |
| WP_011602045.1 | FRAAL0809 | P-loop ATPase | 42406 | 7.03 | 4 | 14 | Frankia-8 | F201371.dat | 102 | 9 | 3 | 3 | 3 | 0 | 2 | 2 | 0 | 2 | 0.005% | 2 | 0 | 3 | 1 | 2 | 0.006% | 1.18 | 0.17 | 2.9E-01 | Red |
| WP_041939340.1 | FRAAL3433 | TetR family transcriptional regulator | 49857 | 5.72 | 3 | 14 | Frankia-7 | F201370.dat | 129 | 10 | 3 | 3 | 3 | 1 | 1 | 1 | 3 | 0 | 0.004% | 0 | 3 | 1 | 2 | 2 | 0.005% | 1.18 | 0.17 | 2.9E-01 | Red |
| WP_041938902.1 | FRAAL1383 | hypothetical protein | 34366 | 5.12 | 4 | 14 | Frankia-10 | F201373.dat | 116 | 15 | 4 | 4 | 4 | 1 | 0 | 1 | 2 | 2 | 0.006% | 0 | 1 | 2 | 1 | 4 | 0.008% | 1.18 | 0.17 | 3.1E-01 | Red |
| WP_011607707.1 | FRAAL6670 | Pantothenate synthetase 4 | 31330 | 5.65 | 3 | 14 | Frankia-8 | F201371.dat | 134 | 13 | 2 | 2 | 4 | 1 | 2 | 1 | 2 | 0 | 0.007% | 0 | 1 | 4 | 1 | 2 | 0.009% | 1.18 | 0.17 | 3.1E-01 | Red |
| WP_041939767.1 | FRAAL5826 | polyphosphate kinase | 81999 | 5.50 | 23 | 197 | Frankia-10 | F201373.dat | 767 | 29 | 18 | 18 | 22 | 22 | 18 | 17 | 17 | 16 | 0.039% | 20 | 17 | 27 | 21 | 22 | 0.044% | 1.18 | 0.16 | 5.9E-02 | Red |
| WP_011604580.1 | FRAAL3435 | luciferase | 31689 | 5.23 | 10 | 51 | Frankia-8 | F201371.dat | 428 | 41 | 7 | 7 | 7 | 4 | 5 | 5 | 4 | 5 | 0.026% | 4 | 6 | 7 | 5 | 6 | 0.030% | 1.18 | 0.16 | 6.9E-02 | Red |
| WP_011606428.1 | FRAAL5341 | oxidoreductase | 56912 | 6.23 | 12 | 101 | Frankia-10 | F201373.dat | 438 | 24 | 9 | 9 | 13 | 9 | 9 | 8 | 9 | 11 | 0.029% | 8 | 11 | 13 | 10 | 13 | 0.033% | 1.18 | 0.16 | 6.5E-02 | Red |
| WP_011607755.1 | FRAAL6718 | geranylgeranyl reductase | 43537 | 9.48 | 7 | 27 | Frankia-8 | F201371.dat | 235 | 11 | 4 | 4 | 4 | 3 | 3 | 3 | 1 | 2 | 0.010% | 3 | 2 | 4 | 3 | 3 | 0.012% | 1.18 | 0.16 | 1.4E-01 | Red |
| WP_011606081.1 | FRAAL4967 | tryptophan synthase subunit alpha | 27561 | 4.60 | 7 | 27 | Frankia-1 | F201364.dat | 250 | 29 | 5 | 5 | 5 | 5 | 1 | 2 | 2 | 2 | 0.016% | 3 | 3 | 3 | 3 | 3 | 0.018% | 1.18 | 0.16 | 2.2E-01 | Red |
| WP_011605766.1 | FRAAL4650 | anthranilate synthase | 48568 | 5.59 | 7 | 27 | Frankia-8 | F201371.dat | 244 | 13 | 5 | 5 | 5 | 2 | 3 | 2 | 2 | 3 | 0.009% | 1 | 3 | 5 | 5 | 1 | 0.010% | 1.18 | 0.16 | 2.7E-01 | Red |
| WP_011607046.1 | FRAAL5991 | anti-sigma factor | 18126 | 4.32 | 9 | 90 | Frankia-7 | F201370.dat | 443 | 56 | 8 | 8 | 11 | 7 | 8 | 9 | 8 | 9 | 0.081% | 8 | 11 | 12 | 8 | 10 | 0.091% | 1.17 | 0.16 | 5.4E-02 | Red |
| WP_011605392.1 | FRAAL4266 | luciferase | 40205 | 5.61 | 11 | 40 | Frankia-8 | F201371.dat | 233 | 29 | 7 | 7 | 7 | 3 | 1 | 4 | 4 | 6 | 0.016% | 2 | 5 | 7 | 3 | 5 | 0.018% | 1.17 | 0.16 | 2.6E-01 | Red |
| WP_011602165.1 | FRAAL0935 | mycothiol acetyltransferase | 35388 | 5.59 | 9 | 66 | Frankia-3 | F201366.dat | 276 | 30 | 8 | 8 | 8 | 7 | 3 | 8 | 6 | 6 | 0.030% | 5 | 8 | 8 | 8 | 7 | 0.034% | 1.17 | 0.16 | 1.4E-01 | Red |
| WP_011605598.1 | FRAAL4477 | hypothetical protein | 48341 | 5.72 | 10 | 68 | Frankia-10 | F201373.dat | 454 | 25 | 8 | 8 | 9 | 7 | 7 | 5 | 7 | 5 | 0.023% | 7 | 6 | 7 | 8 | 9 | 0.026% | 1.17 | 0.15 | 6.4E-02 | Red |
| WP_011607911.1 | FRAAL6876 | chromosome partitioning protein ParB | 35876 | 6.64 | 12 | 68 | Frankia-4 | F201367.dat | 473 | 33 | 8 | 8 | 8 | 5 | 6 | 8 | 8 | 4 | 0.031% | 8 | 8 | 7 | 8 | 6 | 0.035% | 1.17 | 0.15 | 1.1E-01 | Red |
| WP_011606831.1 | FRAAL5758 | ribosome-binding factor A | 22058 | 4.56 | 8 | 42 | Frankia-5 | F201368.dat | 172 | 18 | 4 | 4 | 5 | 3 | 3 | 3 | 5 | 5 | 0.031% | 5 | 6 | 3 | 5 | 4 | 0.035% | 1.17 | 0.15 | 1.5E-01 | Red |
| WP_041939224.1 | FRAAL2799 | peptide deformylase | 20659 | 4.99 | 9 | 55 | Frankia-7 | F201370.dat | 273 | 49 | 7 | 7 | 7 | 2 | 5 | 7 | 5 | 6 | 0.043% | 4 | 7 | 7 | 6 | 6 | 0.049% | 1.17 | 0.15 | 1.7E-01 | Red |
| WP_011604908.1 | FRAAL3770 | dolichol-phosphate mannosyltransferase | 30522 | 6.76 | 7 | 29 | Frankia-8 | F201371.dat | 168 | 20 | 4 | 4 | 4 | 3 | 4 | 2 | 1 | 3 | 0.015% | 3 | 2 | 4 | 4 | 3 | 0.018% | 1.17 | 0.15 | 1.9E-01 | Red |
| WP_011601304.1 | FRAAL0036 | membrane protein | 54291 | 8.31 | 9 | 55 | Frankia-8 | F201371.dat | 300 | 17 | 7 | 7 | 10 | 6 | 4 | 5 | 6 | 4 | 0.016% | 4 | 6 | 10 | 5 | 5 | 0.019% | 1.17 | 0.15 | 2.0E-01 | Red |
| WP_011603240.1 | FRAAL2074 | inositol-phosphate phosphatase | 27414 | 4.72 | 5 | 16 | Frankia-8 | F201371.dat | 159 | 17 | 3 | 3 | 3 | 1 | 1 | 2 | 2 | 1 | 0.009% | 1 | 1 | 3 | 2 | 2 | 0.011% | 1.17 | 0.15 | 2.2E-01 | Red |
| WP_011607828.1 | FRAAL6795 | nucleotide-diphosphate-sugar epimerase | 29147 | 4.81 | 3 | 16 | Frankia-10 | F201373.dat | 142 | 13 | 3 | 3 | 3 | 1 | 1 | 1 | 2 | 2 | 0.009% | 1 | 1 | 2 | 2 | 3 | 0.010% | 1.17 | 0.15 | 2.2E-01 | Red |
| WP_011603250.1 | FRAAL2084 | portal protein | 23751 | 4.47 | 4 | 16 | Frankia-9 | F201372.dat | 187 | 30 | 4 | 4 | 4 | 1 | 1 | 2 | 1 | 2 | 0.011% | 1 | 1 | 2 | 4 | 1 | 0.013% | 1.17 | 0.15 | 2.8E-01 | Red |
| WP_011603064.1 | FRAAL1887 | glutamate--ammonia ligase | 46884 | 5.29 | 5 | 29 | Frankia-8 | F201371.dat | 220 | 13 | 4 | 4 | 6 | 0 | 3 | 3 | 2 | 5 | 0.010% | 2 | 2 | 6 | 3 | 3 | 0.011% | 1.17 | 0.15 | 3.0E-01 | Red |
| WP_011606842.1 | FRAAL5769 | sulfate ABC transporter ATP-binding protein | 27706 | 6.99 | 7 | 16 | Frankia-5 | F201368.dat | 88 | 21 | 4 | 4 | 4 | 0 | 1 | 0 | 2 | 4 | 0.009% | 0 | 3 | 2 | 2 | 2 | 0.011% | 1.17 | 0.15 | 3.3E-01 | Red |
| WP_041939895.1 | FRAAL6433 | methyltransferase FkbM | 27789 | 5.91 | 2 | 3 | Frankia-8 | F201371.dat | 51 | 3 | 1 | 1 | 1 | 1 | 0 | 0 | 0 | 0 | 0.001% | 0 | 1 | 1 | 0 | 0 | 0.002% | 1.17 | 0.15 | 3.4E-01 | Red |
| WP_041941102.1 | FRAAL6426 | glutamate--ammonia ligase | 49574 | 5.97 | 2 | 3 | Frankia-7 | F201370.dat | 47 | 3 | 1 | 1 | 1 | 0 | 0 | 0 | 1 | 0 | 0.001% | 0 | 1 | 0 | 0 | 1 | 0.001% | 1.17 | 0.15 | 3.4E-01 | Red |
| WP_011605648.1 | FRAAL4529 | hypothetical protein | 40707 | 5.50 | 2 | 3 | Frankia-9 | F201372.dat | 42 | 2 | 1 | 1 | 1 | 0 | 0 | 0 | 0 | 1 | 0.001% | 0 | 0 | 1 | 1 | 0 | 0.002% | 1.17 | 0.15 | 3.4E-01 | Red |
| WP_011603484.1 | FRAAL2320 | hypothetical protein | 71967 | 5.59 | 3 | 3 | Frankia-8 | F201371.dat | 67 | 5 | 2 | 2 | 2 | 1 | 0 | 0 | 0 | 0 | 0.000% | 0 | 0 | 2 | 0 | 0 | 0.001% | 1.17 | 0.15 | 3.6E-01 | Red |
| WP_050997123.1 | FRAAL3247 | hypothetical protein | 21328 | 9.02 | 2 | 3 | Frankia-8 | F201371.dat | 98 | 11 | 2 | 2 | 2 | 0 | 0 | 0 | 1 | 0 | 0.002% | 0 | 0 | 2 | 0 | 0 | 0.003% | 1.17 | 0.15 | 3.6E-01 | Red |
| WP_041939310.1 | FRAAL3217 | hypothetical protein | 29967 | 5.27 | 2 | 3 | Frankia-8 | F201371.dat | 97 | 8 | 2 | 2 | 2 | 0 | 0 | 0 | 0 | 1 | 0.001% | 0 | 0 | 2 | 0 | 0 | 0.002% | 1.17 | 0.15 | 3.6E-01 | Red |
| WP_011607266.1 | FRAAL6216 | PadR family transcriptional regulator | 24966 | 6.33 | 2 | 3 | Frankia-9 | F201372.dat | 62 | 10 | 2 | 2 | 2 | 0 | 0 | 0 | 0 | 1 | 0.001% | 0 | 0 | 0 | 2 | 0 | 0.003% | 1.17 | 0.15 | 3.6E-01 | Red |
| WP_011607841.1 | FRAAL6807 | hypothetical protein | 16204 | 4.90 | 14 | 174 | Frankia-8 | F201371.dat | 765 | 84 | 12 | 12 | 20 | 17 | 17 | 18 | 17 | 11 | 0.176% | 16 | 18 | 20 | 22 | 18 | 0.195% | 1.16 | 0.15 | 6.2E-02 | Red |
| WP_011605889.1 | FRAAL4774 | F420-dependent oxidoreductase | 36420 | 5.78 | 19 | 122 | Frankia-8 | F201371.dat | 794 | 56 | 14 | 14 | 19 | 12 | 11 | 8 | 12 | 13 | 0.055% | 8 | 13 | 19 | 12 | 14 | 0.061% | 1.16 | 0.15 | 1.7E-01 | Red |
| WP_050997207.1 | FRAAL4885 | FMNH2-dependent monooxygenase | 44240 | 5.19 | 12 | 83 | Frankia-7 | F201370.dat | 359 | 31 | 10 | 10 | 11 | 8 | 6 | 7 | 7 | 10 | 0.031% | 7 | 11 | 10 | 7 | 10 | 0.034% | 1.16 | 0.15 | 1.1E-01 | Red |
| WP_041941008.1 | FRAAL5896 | membrane protein | 41275 | 5.10 | 6 | 57 | Frankia-6 | F201369.dat | 318 | 16 | 5 | 5 | 7 | 5 | 5 | 6 | 5 | 5 | 0.022% | 7 | 4 | 7 | 8 | 5 | 0.025% | 1.16 | 0.15 | 1.2E-01 | Red |
| WP_011606306.1 | FRAAL5212 | PAS domain-containing two-component system sensor histidine kinase | 40549 | 5.50 | 17 | 124 | Frankia-7 | F201370.dat | 675 | 42 | 16 | 16 | 19 | 12 | 11 | 11 | 10 | 13 | 0.050% | 12 | 19 | 10 | 15 | 11 | 0.056% | 1.16 | 0.15 | 1.4E-01 | Red |
| WP_050997308.1 | FRAAL0453 | CRISPR-associated protein Cse1 | 57542 | 6.06 | 8 | 44 | Frankia-1 | F201364.dat | 234 | 11 | 4 | 4 | 5 | 5 | 4 | 4 | 3 | 4 | 0.012% | 4 | 4 | 5 | 6 | 5 | 0.014% | 1.16 | 0.15 | 7.4E-02 | Red |
| WP_011606258.1 | FRAAL5154 | serine/threonine protein kinase | 71381 | 10.50 | 8 | 44 | Frankia-8 | F201371.dat | 304 | 14 | 7 | 7 | 8 | 4 | 3 | 5 | 5 | 3 | 0.010% | 2 | 3 | 8 | 5 | 6 | 0.011% | 1.16 | 0.15 | 2.5E-01 | Red |
| WP_041938907.1 | FRAAL1402 | integrase | 39873 | 4.72 | 5 | 31 | Frankia-7 | F201370.dat | 215 | 18 | 5 | 5 | 5 | 4 | 2 | 3 | 2 | 3 | 0.013% | 2 | 5 | 3 | 3 | 4 | 0.014% | 1.16 | 0.15 | 1.9E-01 | Red |
| WP_011607892.1 | FRAAL6857 | glycosyl transferase | 134478 | 10.05 | 7 | 31 | Frankia-8 | F201371.dat | 256 | 4 | 5 | 5 | 5 | 3 | 3 | 5 | 2 | 1 | 0.004% | 3 | 2 | 5 | 4 | 3 | 0.004% | 1.16 | 0.15 | 2.5E-01 | Red |
| WP_041940188.1 | FRAAL1230 | dTDP-4-dehydrorhamnose reductase | 31312 | 5.21 | 7 | 59 | Frankia-10 | F201373.dat | 278 | 28 | 6 | 6 | 7 | 6 | 5 | 4 | 5 | 7 | 0.031% | 6 | 5 | 8 | 6 | 7 | 0.034% | 1.16 | 0.15 | 1.0E-01 | Red |
| WP_011603384.1 | FRAAL2215 | peptidase S16 | 24251 | 5.66 | 10 | 59 | Frankia-8 | F201371.dat | 350 | 48 | 8 | 8 | 9 | 5 | 5 | 6 | 6 | 5 | 0.040% | 6 | 6 | 9 | 5 | 6 | 0.044% | 1.16 | 0.15 | 1.1E-01 | Red |
| WP_009740324.1 | FRAAL1304 | chemotaxis protein CheY | 26241 | 4.90 | 12 | 87 | Frankia-8 | F201371.dat | 404 | 37 | 8 | 8 | 11 | 9 | 9 | 6 | 9 | 7 | 0.054% | 10 | 6 | 11 | 10 | 10 | 0.060% | 1.16 | 0.14 | 1.1E-01 | Red |
| WP_050997011.1 | FRAAL1090 | 30S ribosomal protein S17 | 11369 | 9.91 | 15 | 240 | Frankia-5 | F201368.dat | 772 | 87 | 14 | 14 | 27 | 18 | 25 | 18 | 23 | 27 | 0.348% | 23 | 21 | 20 | 30 | 35 | 0.382% | 1.16 | 0.14 | 1.6E-01 | Red |
| WP_041939924.1 | FRAAL6579 | DNA topoisomerase I | 114174 | 9.31 | 44 | 311 | Frankia-1 | F201364.dat | 1836 | 38 | 31 | 31 | 38 | 38 | 29 | 25 | 28 | 24 | 0.045% | 40 | 26 | 41 | 30 | 30 | 0.049% | 1.15 | 0.14 | 1.4E-01 | Red |
| WP_009738606.1 | FRAAL3771 | membrane protein | 12882 | 5.73 | 6 | 46 | Frankia-6 | F201369.dat | 300 | 47 | 6 | 6 | 6 | 4 | 6 | 4 | 5 | 2 | 0.058% | 6 | 5 | 5 | 5 | 4 | 0.065% | 1.15 | 0.14 | 1.6E-01 | Red |
| WP_041939762.1 | FRAAL5807 | metal-binding protein | 21914 | 4.77 | 11 | 74 | Frankia-9 | F201372.dat | 398 | 50 | 7 | 7 | 9 | 7 | 8 | 7 | 9 | 3 | 0.055% | 7 | 9 | 8 | 9 | 7 | 0.061% | 1.15 | 0.14 | 1.6E-01 | Red |
| WP_011605368.1 | FRAAL4242 | alpha/beta hydrolase | 35299 | 6.19 | 6 | 46 | Frankia-8 | F201371.dat | 245 | 20 | 6 | 6 | 7 | 4 | 4 | 5 | 4 | 4 | 0.021% | 5 | 3 | 7 | 6 | 4 | 0.024% | 1.15 | 0.14 | 1.7E-01 | Red |
| WP_011603038.1 | FRAAL1861 | DSBA oxidoreductase | 23822 | 5.06 | 6 | 46 | Frankia-10 | F201373.dat | 236 | 39 | 6 | 6 | 7 | 5 | 3 | 4 | 4 | 5 | 0.031% | 3 | 4 | 5 | 6 | 7 | 0.035% | 1.15 | 0.14 | 1.7E-01 | Red |
| WP_011601672.1 | FRAAL0416 | (2Fe-2S)-binding protein | 40446 | 5.94 | 8 | 46 | Frankia-7 | F201370.dat | 194 | 18 | 6 | 6 | 7 | 5 | 5 | 5 | 2 | 4 | 0.019% | 5 | 7 | 5 | 3 | 5 | 0.021% | 1.15 | 0.14 | 1.9E-01 | Red |
| WP_011602453.1 | FRAAL1252 | glycosyl transferase family 1 | 49465 | 10.75 | 10 | 46 | Frankia-7 | F201370.dat | 307 | 18 | 7 | 7 | 7 | 5 | 4 | 5 | 5 | 2 | 0.015% | 3 | 7 | 6 | 4 | 5 | 0.017% | 1.15 | 0.14 | 2.0E-01 | Red |
| WP_011607131.1 | FRAAL6079 | diacylglycerol kinase | 32753 | 11.10 | 5 | 18 | Frankia-8 | F201371.dat | 157 | 10 | 3 | 3 | 4 | 2 | 2 | 1 | 1 | 2 | 0.009% | 1 | 1 | 4 | 1 | 3 | 0.010% | 1.15 | 0.14 | 3.0E-01 | Red |
| WP_011607033.1 | FRAAL5977 | DNA helicase | 177988 | 5.33 | 5 | 18 | Frankia-8 | F201371.dat | 258 | 3 | 4 | 4 | 4 | 3 | 3 | 1 | 0 | 1 | 0.002% | 2 | 0 | 4 | 2 | 2 | 0.002% | 1.15 | 0.14 | 3.3E-01 | Red |
| WP_041939598.1 | FRAAL4949 | protein kinase | 36089 | 7.08 | 9 | 18 | Frankia-8 | F201371.dat | 168 | 14 | 5 | 4 | 5 | 1 | 0 | 1 | 2 | 4 | 0.008% | 0 | 1 | 5 | 2 | 2 | 0.009% | 1.15 | 0.14 | 3.6E-01 | Red |
| WP_009739506.1 | FRAAL2271 | hypothetical protein | 19341 | 5.37 | 8 | 89 | Frankia-8 | F201371.dat | 470 | 41 | 6 | 6 | 11 | 8 | 7 | 9 | 8 | 9 | 0.076% | 8 | 8 | 11 | 11 | 10 | 0.084% | 1.15 | 0.14 | 5.4E-02 | Red |
| WP_011604911.1 | FRAAL3773 | methionine sulfoxide reductase B | 15665 | 5.07 | 6 | 61 | Frankia-10 | F201373.dat | 236 | 47 | 5 | 5 | 6 | 4 | 4 | 6 | 8 | 6 | 0.064% | 7 | 7 | 6 | 7 | 6 | 0.071% | 1.15 | 0.14 | 1.3E-01 | Red |
| WP_041939352.1 | FRAAL3528 | short-chain dehydrogenase | 28215 | 6.11 | 11 | 61 | Frankia-8 | F201371.dat | 337 | 32 | 7 | 7 | 8 | 5 | 5 | 7 | 6 | 5 | 0.035% | 6 | 7 | 8 | 8 | 4 | 0.039% | 1.15 | 0.14 | 1.4E-01 | Red |
| WP_011602524.1 | FRAAL1327 | hypothetical protein | 36313 | 10.77 | 21 | 246 | Frankia-7 | F201370.dat | 1054 | 54 | 19 | 19 | 35 | 24 | 22 | 25 | 20 | 23 | 0.112% | 29 | 35 | 16 | 29 | 23 | 0.122% | 1.15 | 0.14 | 1.6E-01 | Red |
| WP_011607636.1 | FRAAL6599 | hypothetical protein | 20797 | 4.97 | 9 | 104 | Frankia-2 | F201365.dat | 396 | 57 | 7 | 7 | 12 | 10 | 12 | 9 | 10 | 7 | 0.082% | 10 | 12 | 12 | 11 | 11 | 0.091% | 1.15 | 0.14 | 5.6E-02 | Red |
| WP_050997420.1 | FRAAL4768 | carnitine dehydratase | 39607 | 5.13 | 4 | 33 | Frankia-5 | F201368.dat | 287 | 16 | 4 | 4 | 4 | 2 | 3 | 4 | 2 | 4 | 0.014% | 3 | 3 | 4 | 3 | 5 | 0.015% | 1.15 | 0.14 | 1.7E-01 | Red |
| WP_011607711.1 | FRAAL6674 | type III pantothenate kinase | 26553 | 5.98 | 5 | 33 | Frankia-5 | F201368.dat | 182 | 23 | 5 | 5 | 6 | 2 | 3 | 2 | 2 | 6 | 0.020% | 3 | 5 | 2 | 4 | 4 | 0.023% | 1.15 | 0.14 | 2.7E-01 | Red |
| WP_011604715.1 | FRAAL3574 | hypothetical protein | 19879 | 4.84 | 4 | 48 | Frankia-7 | F201370.dat | 160 | 18 | 4 | 4 | 7 | 5 | 5 | 2 | 6 | 4 | 0.039% | 4 | 7 | 5 | 5 | 5 | 0.044% | 1.15 | 0.14 | 1.8E-01 | Red |
| WP_041938749.1 | FRAAL0698 | hypothetical protein | 80627 | 5.36 | 10 | 48 | Frankia-8 | F201371.dat | 270 | 13 | 8 | 8 | 8 | 6 | 5 | 3 | 4 | 4 | 0.010% | 3 | 3 | 8 | 6 | 6 | 0.011% | 1.15 | 0.14 | 2.4E-01 | Red |
| WP_011607294.1 | FRAAL6245 | short-chain dehydrogenase | 26457 | 6.38 | 9 | 63 | Frankia-7 | F201370.dat | 311 | 40 | 7 | 7 | 7 | 3 | 6 | 4 | 7 | 9 | 0.039% | 6 | 7 | 7 | 6 | 8 | 0.043% | 1.15 | 0.14 | 2.0E-01 | Red |
| WP_041939074.1 | FRAAL2200 | cell division protein FtsZ | 51757 | 4.78 | 19 | 166 | Frankia-4 | F201367.dat | 634 | 39 | 13 | 13 | 20 | 19 | 12 | 16 | 20 | 10 | 0.053% | 15 | 18 | 21 | 17 | 18 | 0.058% | 1.15 | 0.14 | 1.5E-01 | Red |
| WP_011606851.1 | FRAAL5779 | elongation factor Ts | 22368 | 6.77 | 30 | 635 | Frankia-10 | F201373.dat | 1533 | 87 | 27 | 27 | 78 | 54 | 56 | 56 | 64 | 66 | 0.472% | 54 | 64 | 74 | 69 | 78 | 0.510% | 1.14 | 0.13 | 5.6E-02 | Red |
| WP_011602492.1 | FRAAL1293 | glyoxalase | 13634 | 5.67 | 7 | 50 | Frankia-9 | F201372.dat | 255 | 46 | 5 | 5 | 6 | 5 | 5 | 5 | 4 | 4 | 0.060% | 4 | 6 | 6 | 6 | 5 | 0.067% | 1.14 | 0.13 | 8.1E-02 | Red |
| WP_011602713.1 | FRAAL1523 | peroxiredoxin | 17442 | 7.73 | 9 | 65 | Frankia-7 | F201370.dat | 286 | 52 | 7 | 7 | 8 | 5 | 6 | 6 | 5 | 8 | 0.061% | 4 | 8 | 7 | 7 | 9 | 0.068% | 1.14 | 0.13 | 1.7E-01 | Red |
| WP_011603952.1 | FRAAL2800 | luciferase | 34107 | 5.29 | 6 | 35 | Frankia-1 | F201364.dat | 266 | 16 | 5 | 5 | 5 | 5 | 4 | 3 | 2 | 2 | 0.017% | 3 | 4 | 4 | 5 | 3 | 0.019% | 1.14 | 0.13 | 2.1E-01 | Red |
| WP_011604565.1 | FRAAL3420 | thiolase | 39930 | 5.32 | 2 | 20 | Frankia-2 | F201365.dat | 105 | 8 | 2 | 2 | 2 | 2 | 2 | 1 | 2 | 2 | 0.008% | 2 | 2 | 3 | 2 | 2 | 0.009% | 1.14 | 0.13 | 2.1E-01 | Red |
| WP_011604517.1 | FRAAL3370 | hypothetical protein | 11663 | 5.32 | 10 | 95 | Frankia-10 | F201373.dat | 450 | 73 | 9 | 9 | 14 | 8 | 11 | 7 | 9 | 9 | 0.135% | 11 | 5 | 8 | 13 | 14 | 0.147% | 1.14 | 0.13 | 2.3E-01 | Red |
| WP_011604702.1 | FRAAL3561 | acyl-CoA dehydrogenase | 48307 | 5.94 | 6 | 20 | Frankia-8 | F201371.dat | 170 | 7 | 3 | 3 | 3 | 3 | 2 | 1 | 1 | 2 | 0.007% | 2 | 3 | 3 | 1 | 2 | 0.008% | 1.14 | 0.13 | 2.4E-01 | Red |
| WP_041940750.1 | FRAAL4381 | hypothetical protein | 46001 | 5.94 | 5 | 20 | Frankia-8 | F201371.dat | 214 | 14 | 4 | 4 | 4 | 1 | 2 | 2 | 2 | 2 | 0.007% | 1 | 2 | 4 | 2 | 2 | 0.008% | 1.14 | 0.13 | 2.6E-01 | Red |
| WP_041939663.1 | FRAAL5239 | uracil phosphoribosyltransferase | 21975 | 8.16 | 6 | 20 | Frankia-10 | F201373.dat | 265 | 37 | 5 | 5 | 5 | 1 | 2 | 1 | 2 | 3 | 0.015% | 1 | 1 | 2 | 2 | 5 | 0.017% | 1.14 | 0.13 | 3.2E-01 | Red |
| WP_011604031.1 | FRAAL2885 | RNA helicase | 108006 | 9.61 | 4 | 20 | Frankia-1 | F201364.dat | 214 | 6 | 4 | 4 | 4 | 4 | 0 | 2 | 2 | 1 | 0.003% | 2 | 0 | 3 | 3 | 3 | 0.003% | 1.14 | 0.13 | 3.3E-01 | Red |
| WP_041938977.1 | FRAAL1728 | GntR family transcriptional regulator | 27786 | 6.11 | 2 | 5 | Frankia-8 | F201371.dat | 94 | 8 | 2 | 2 | 1 | 0 | 1 | 0 | 1 | 0 | 0.003% | 1 | 1 | 1 | 0 | 0 | 0.004% | 1.14 | 0.13 | 3.4E-01 | Red |
| WP_041938796.1 | FRAAL0872 | hypothetical protein | 71003 | 5.13 | 2 | 5 | Frankia-9 | F201372.dat | 64 | 3 | 1 | 1 | 1 | 0 | 0 | 1 | 0 | 1 | 0.001% | 0 | 0 | 1 | 1 | 1 | 0.001% | 1.14 | 0.13 | 3.4E-01 | Red |
| WP_041939254.1 | FRAAL2939 | protein-L-isoaspartate O-methyltransferase | 40755 | 6.70 | 2 | 5 | Frankia-7 | F201370.dat | 48 | 3 | 1 | 1 | 1 | 0 | 0 | 0 | 1 | 1 | 0.002% | 0 | 1 | 1 | 0 | 1 | 0.002% | 1.14 | 0.13 | 3.4E-01 | Red |
| WP_050997459.1 | FRAAL6098 | dihydropteroate synthase | 28295 | 5.28 | 6 | 20 | Frankia-8 | F201371.dat | 213 | 17 | 5 | 5 | 5 | 1 | 2 | 2 | 2 | 2 | 0.011% | 0 | 1 | 5 | 1 | 4 | 0.013% | 1.14 | 0.13 | 3.5E-01 | Red |
| WP_050997260.1 | FRAAL6117 | hypothetical protein | 46352 | 6.81 | 2 | 5 | Frankia-8 | F201371.dat | 123 | 8 | 2 | 2 | 2 | 1 | 0 | 0 | 0 | 1 | 0.002% | 0 | 1 | 2 | 0 | 0 | 0.002% | 1.14 | 0.13 | 3.6E-01 | Red |
| WP_011601748.1 | FRAAL0495 | hypothetical protein | 28145 | 7.79 | 2 | 5 | Frankia-8 | F201371.dat | 103 | 10 | 2 | 2 | 2 | 0 | 0 | 1 | 0 | 1 | 0.003% | 0 | 1 | 2 | 0 | 0 | 0.004% | 1.14 | 0.13 | 3.6E-01 | Red |
| WP_011607065.1 | FRAAL6012 | type 11 methyltransferase | 28289 | 4.84 | 2 | 5 | Frankia-4 | F201367.dat | 100 | 11 | 2 | 2 | 2 | 0 | 0 | 0 | 2 | 0 | 0.003% | 1 | 0 | 1 | 1 | 0 | 0.004% | 1.14 | 0.13 | 3.6E-01 | Red |
| WP_041939710.1 | FRAAL5462 | TetR family transcriptional regulator | 19922 | 6.84 | 2 | 5 | Frankia-10 | F201373.dat | 75 | 12 | 2 | 2 | 2 | 1 | 0 | 1 | 0 | 0 | 0.004% | 0 | 1 | 0 | 0 | 2 | 0.005% | 1.14 | 0.13 | 3.6E-01 | Red |
| WP_041939527.1 | FRAAL4524 | potassium transporter TrkA | 16980 | 5.00 | 2 | 5 | Frankia-6 | F201369.dat | 68 | 17 | 2 | 2 | 2 | 0 | 0 | 1 | 1 | 0 | 0.004% | 2 | 0 | 0 | 0 | 1 | 0.006% | 1.14 | 0.13 | 3.6E-01 | Red |
| WP_011603298.1 | FRAAL2133 | lauroyl acyltransferase | 31081 | 10.22 | 3 | 5 | Frankia-4 | F201367.dat | 82 | 7 | 2 | 2 | 2 | 0 | 0 | 0 | 2 | 0 | 0.002% | 0 | 0 | 0 | 2 | 1 | 0.003% | 1.14 | 0.13 | 3.7E-01 | Red |
| WP_011601334.1 | FRAAL0064 | patatin | 134061 | 7.99 | 3 | 5 | Frankia-6 | F201369.dat | 77 | 2 | 3 | 3 | 3 | 2 | 0 | 0 | 0 | 0 | 0.001% | 3 | 0 | 0 | 0 | 0 | 0.001% | 1.14 | 0.13 | 3.9E-01 | Red |
| WP_041939754.1 | FRAAL5747 | epimerase | 34894 | 5.92 | 15 | 127 | Frankia-5 | F201368.dat | 534 | 51 | 12 | 12 | 14 | 11 | 9 | 14 | 11 | 14 | 0.060% | 8 | 15 | 16 | 14 | 15 | 0.066% | 1.14 | 0.13 | 1.6E-01 | Red |
| WP_011604548.1 | FRAAL3402 | dioxygenase | 47853 | 5.53 | 16 | 97 | Frankia-8 | F201371.dat | 529 | 37 | 11 | 11 | 13 | 10 | 7 | 10 | 9 | 9 | 0.034% | 6 | 12 | 13 | 11 | 10 | 0.037% | 1.14 | 0.13 | 1.6E-01 | Red |
| WP_041939755.1 | FRAAL5751 | 4-hydroxy-tetrahydrodipicolinate reductase | 25765 | 5.22 | 11 | 82 | Frankia-9 | F201372.dat | 357 | 48 | 8 | 8 | 10 | 5 | 8 | 8 | 7 | 10 | 0.053% | 6 | 9 | 10 | 10 | 9 | 0.058% | 1.14 | 0.13 | 1.5E-01 | Red |
| WP_011603505.1 | FRAAL2341 | ATPase AAA | 45529 | 5.04 | 9 | 67 | Frankia-3 | F201366.dat | 239 | 20 | 7 | 7 | 7 | 5 | 5 | 7 | 6 | 8 | 0.024% | 7 | 5 | 8 | 9 | 7 | 0.027% | 1.14 | 0.13 | 1.5E-01 | Red |
| WP_041938851.1 | FRAAL1114 | 50S ribosomal protein L13 | 16228 | 10.10 | 11 | 193 | Frankia-5 | F201368.dat | 430 | 72 | 10 | 10 | 20 | 15 | 21 | 16 | 18 | 20 | 0.198% | 22 | 19 | 22 | 22 | 18 | 0.214% | 1.14 | 0.13 | 5.4E-02 | Red |
| WP_011603032.1 | FRAAL1854 | hypothetical protein | 14558 | 9.29 | 2 | 37 | Frankia-5 | F201368.dat | 181 | 29 | 2 | 2 | 3 | 3 | 3 | 4 | 4 | 3 | 0.042% | 4 | 4 | 4 | 4 | 4 | 0.046% | 1.14 | 0.13 | 1.2E-01 | Red |
| WP_011606326.1 | FRAAL5233 | orotidine 5'-phosphate decarboxylase | 26756 | 7.98 | 7 | 54 | Frankia-10 | F201373.dat | 349 | 41 | 7 | 7 | 8 | 6 | 5 | 5 | 5 | 4 | 0.033% | 4 | 5 | 7 | 5 | 8 | 0.037% | 1.13 | 0.13 | 1.7E-01 | Red |
| WP_011603487.1 | FRAAL2323 | hypothetical protein | 42123 | 6.03 | 11 | 54 | Frankia-7 | F201370.dat | 319 | 30 | 7 | 7 | 7 | 4 | 4 | 4 | 7 | 6 | 0.021% | 6 | 7 | 7 | 5 | 4 | 0.023% | 1.13 | 0.13 | 1.9E-01 | Red |
| WP_050997041.1 | FRAAL1761 | oligoribonuclease | 25902 | 5.15 | 7 | 54 | Frankia-10 | F201373.dat | 247 | 33 | 6 | 6 | 8 | 5 | 6 | 3 | 6 | 5 | 0.034% | 4 | 4 | 7 | 6 | 8 | 0.038% | 1.13 | 0.13 | 2.2E-01 | Red |
| WP_011607277.1 | FRAAL6227 | NADH dehydrogenase | 54149 | 6.69 | 16 | 118 | Frankia-3 | F201366.dat | 730 | 33 | 14 | 14 | 17 | 13 | 9 | 17 | 8 | 8 | 0.036% | 14 | 10 | 15 | 11 | 13 | 0.039% | 1.13 | 0.13 | 2.2E-01 | Red |
| WP_041938820.1 | FRAAL0952 | Appr-1-p processing protein | 17805 | 6.05 | 3 | 22 | Frankia-8 | F201371.dat | 225 | 24 | 3 | 3 | 3 | 2 | 2 | 2 | 2 | 2 | 0.020% | 2 | 1 | 3 | 3 | 3 | 0.023% | 1.13 | 0.13 | 2.3E-01 | Red |
| WP_011601974.1 | FRAAL0733 | hypothetical protein | 34160 | 6.73 | 3 | 22 | Frankia-1 | F201364.dat | 95 | 9 | 2 | 2 | 3 | 3 | 1 | 2 | 2 | 2 | 0.010% | 2 | 2 | 4 | 2 | 2 | 0.012% | 1.13 | 0.13 | 2.3E-01 | Red |
| WP_011603949.1 | FRAAL2797 | tRNA N6-adenosine threonylcarbamoyltransferase | 36354 | 5.53 | 7 | 22 | Frankia-8 | F201371.dat | 178 | 23 | 4 | 4 | 4 | 1 | 2 | 2 | 2 | 3 | 0.010% | 1 | 3 | 4 | 2 | 2 | 0.011% | 1.13 | 0.13 | 2.6E-01 | Red |
| WP_011602695.1 | FRAAL1505 | glutamate racemase | 32140 | 5.33 | 6 | 22 | Frankia-8 | F201371.dat | 186 | 22 | 4 | 4 | 4 | 3 | 2 | 2 | 2 | 1 | 0.011% | 2 | 2 | 4 | 1 | 3 | 0.013% | 1.13 | 0.13 | 2.6E-01 | Red |
| WP_011607774.1 | FRAAL6737 | sulfurtransferase | 12259 | 4.55 | 3 | 22 | Frankia-9 | F201372.dat | 187 | 48 | 3 | 3 | 3 | 0 | 2 | 3 | 3 | 2 | 0.029% | 2 | 3 | 2 | 3 | 2 | 0.033% | 1.13 | 0.13 | 2.7E-01 | Red |
| WP_011604540.1 | FRAAL3393 | cyclohexanecarboxylate-CoA ligase | 58418 | 4.97 | 5 | 22 | Frankia-10 | F201373.dat | 118 | 7 | 4 | 4 | 4 | 2 | 2 | 0 | 4 | 2 | 0.006% | 2 | 2 | 2 | 2 | 4 | 0.007% | 1.13 | 0.13 | 3.0E-01 | Red |
| WP_011607678.1 | FRAAL6641 | molecular chaperone DnaJ | 41565 | 8.91 | 15 | 135 | Frankia-8 | F201371.dat | 662 | 43 | 13 | 13 | 20 | 13 | 11 | 13 | 14 | 12 | 0.054% | 11 | 15 | 20 | 11 | 15 | 0.058% | 1.13 | 0.12 | 1.7E-01 | Red |
| WP_011606523.1 | FRAAL5442 | hypothetical protein | 42607 | 5.25 | 14 | 103 | Frankia-8 | F201371.dat | 440 | 29 | 10 | 10 | 14 | 6 | 9 | 11 | 9 | 13 | 0.040% | 10 | 13 | 14 | 8 | 10 | 0.043% | 1.13 | 0.12 | 2.0E-01 | Red |
| WP_011605773.1 | FRAAL4657 | succinyl-diaminopimelate desuccinylase | 41429 | 6.27 | 17 | 103 | Frankia-8 | F201371.dat | 613 | 42 | 12 | 12 | 14 | 9 | 9 | 8 | 11 | 11 | 0.041% | 5 | 9 | 14 | 12 | 15 | 0.045% | 1.13 | 0.12 | 2.4E-01 | Red |
| WP_041939040.1 | FRAAL2024 | deoxyguanosinetriphosphate triphosphohydrolase | 45568 | 6.21 | 11 | 71 | Frankia-10 | F201373.dat | 354 | 20 | 8 | 8 | 10 | 7 | 7 | 6 | 6 | 7 | 0.026% | 8 | 6 | 7 | 7 | 10 | 0.028% | 1.13 | 0.12 | 1.1E-01 | Red |
| WP_011602068.1 | FRAAL0833 | mRNA interferase PemK | 23774 | 6.87 | 8 | 71 | Frankia-8 | F201371.dat | 389 | 41 | 6 | 6 | 9 | 6 | 5 | 7 | 7 | 8 | 0.050% | 5 | 6 | 9 | 6 | 12 | 0.054% | 1.13 | 0.12 | 2.5E-01 | Red |
| WP_041940342.1 | FRAAL2209 | pseudouridine synthase | 33119 | 7.21 | 5 | 39 | Frankia-7 | F201370.dat | 224 | 21 | 4 | 4 | 4 | 4 | 3 | 3 | 4 | 4 | 0.019% | 2 | 4 | 6 | 4 | 5 | 0.021% | 1.13 | 0.12 | 2.2E-01 | Red |
| WP_050997145.1 | FRAAL3760 | 5'-3' exonuclease | 38496 | 5.37 | 8 | 39 | Frankia-10 | F201373.dat | 234 | 17 | 6 | 6 | 6 | 3 | 3 | 3 | 5 | 4 | 0.017% | 2 | 5 | 4 | 4 | 6 | 0.018% | 1.13 | 0.12 | 2.3E-01 | Red |
| WP_050997195.1 | FRAAL4661 | hypothetical protein | 32618 | 4.94 | 19 | 188 | Frankia-10 | F201373.dat | 850 | 65 | 14 | 14 | 22 | 17 | 13 | 16 | 17 | 25 | 0.096% | 16 | 20 | 22 | 20 | 22 | 0.103% | 1.13 | 0.12 | 1.6E-01 | Red |
| WP_041941121.1 | FRAAL6549 | ATPase | 39658 | 5.25 | 11 | 56 | Frankia-7 | F201370.dat | 340 | 24 | 7 | 7 | 7 | 6 | 3 | 7 | 5 | 5 | 0.023% | 6 | 7 | 7 | 4 | 6 | 0.025% | 1.13 | 0.12 | 1.9E-01 | Red |
| WP_011607038.1 | FRAAL5983 | molecular chaperone GroES | 33195 | 6.25 | 11 | 107 | Frankia-8 | F201371.dat | 589 | 46 | 9 | 9 | 14 | 9 | 9 | 12 | 9 | 11 | 0.054% | 8 | 11 | 14 | 12 | 12 | 0.058% | 1.13 | 0.12 | 1.3E-01 | Red |
| WP_041939089.1 | FRAAL2253 | cytidylate kinase | 27293 | 4.97 | 15 | 160 | Frankia-10 | F201373.dat | 604 | 71 | 12 | 12 | 19 | 15 | 12 | 13 | 16 | 19 | 0.098% | 14 | 17 | 19 | 16 | 19 | 0.105% | 1.13 | 0.12 | 1.2E-01 | Red |
| WP_041939099.1 | FRAAL2292 | hypothetical protein | 42528 | 4.21 | 6 | 41 | Frankia-10 | F201373.dat | 448 | 18 | 5 | 5 | 5 | 3 | 4 | 3 | 4 | 5 | 0.016% | 4 | 4 | 6 | 3 | 5 | 0.017% | 1.13 | 0.12 | 1.9E-01 | Red |
| WP_011606426.1 | FRAAL5339 | membrane protein | 10715 | 11.27 | 2 | 24 | Frankia-6 | F201369.dat | 139 | 35 | 2 | 2 | 3 | 2 | 3 | 2 | 2 | 2 | 0.037% | 3 | 2 | 2 | 3 | 3 | 0.041% | 1.13 | 0.12 | 2.1E-01 | Red |
| WP_011605495.1 | FRAAL4373 | hypothetical protein | 16557 | 4.62 | 3 | 24 | Frankia-10 | F201373.dat | 226 | 35 | 3 | 3 | 3 | 1 | 1 | 3 | 3 | 3 | 0.024% | 3 | 2 | 2 | 3 | 3 | 0.026% | 1.13 | 0.12 | 2.6E-01 | Red |
| WP_011601621.1 | FRAAL0364 | hypothetical protein | 22053 | 11.63 | 13 | 160 | Frankia-5 | F201368.dat | 683 | 37 | 11 | 11 | 24 | 12 | 13 | 13 | 13 | 24 | 0.121% | 14 | 12 | 19 | 17 | 23 | 0.130% | 1.13 | 0.12 | 2.6E-01 | Red |
| WP_050997008.1 | FRAAL1001 | oxidoreductase | 19338 | 8.53 | 4 | 24 | Frankia-8 | F201371.dat | 129 | 28 | 4 | 4 | 4 | 3 | 2 | 3 | 0 | 3 | 0.020% | 2 | 2 | 4 | 2 | 3 | 0.023% | 1.13 | 0.12 | 2.9E-01 | Red |
| WP_011601290.1 | FRAAL0022 | hypothetical protein | 22034 | 5.20 | 4 | 24 | Frankia-2 | F201365.dat | 91 | 16 | 4 | 4 | 4 | 2 | 4 | 3 | 2 | 0 | 0.018% | 3 | 3 | 0 | 4 | 3 | 0.020% | 1.13 | 0.12 | 3.4E-01 | Red |
| WP_050997448.1 | FRAAL5776 | 23S rRNA (adenine(2503)-C2)-methyltransferase | 44675 | 9.28 | 3 | 7 | Frankia-8 | F201371.dat | 119 | 6 | 2 | 2 | 2 | 1 | 1 | 0 | 0 | 1 | 0.002% | 1 | 1 | 2 | 0 | 0 | 0.003% | 1.13 | 0.12 | 3.5E-01 | Red |
| WP_011603133.1 | FRAAL1960 | histidine kinase | 54209 | 4.79 | 3 | 7 | Frankia-9 | F201372.dat | 69 | 7 | 2 | 2 | 2 | 1 | 0 | 0 | 1 | 1 | 0.002% | 1 | 0 | 0 | 2 | 1 | 0.002% | 1.13 | 0.12 | 3.5E-01 | Red |
| WP_050997435.1 | FRAAL5293 | oxidoreductase | 26716 | 6.24 | 2 | 7 | Frankia-9 | F201372.dat | 52 | 10 | 2 | 2 | 2 | 0 | 1 | 1 | 0 | 1 | 0.004% | 0 | 1 | 1 | 2 | 0 | 0.005% | 1.13 | 0.12 | 3.5E-01 | Red |
| WP_011605866.1 | FRAAL4751 | TetR family transcriptional regulator | 19870 | 5.08 | 2 | 7 | Frankia-2 | F201365.dat | 58 | 18 | 2 | 2 | 2 | 0 | 2 | 0 | 0 | 1 | 0.005% | 1 | 1 | 0 | 1 | 1 | 0.007% | 1.13 | 0.12 | 3.6E-01 | Red |
| WP_011606769.1 | FRAAL5696 | acetoacetyl-CoA synthetase | 69966 | 4.90 | 4 | 7 | Frankia-3 | F201366.dat | 93 | 3 | 2 | 2 | 2 | 1 | 0 | 2 | 0 | 0 | 0.002% | 0 | 1 | 0 | 1 | 2 | 0.002% | 1.13 | 0.12 | 3.6E-01 | Red |
| WP_011604009.1 | FRAAL2863 | hypothetical protein | 86684 | 10.32 | 3 | 7 | Frankia-8 | F201371.dat | 65 | 3 | 2 | 2 | 2 | 1 | 0 | 2 | 0 | 0 | 0.001% | 1 | 1 | 2 | 0 | 0 | 0.002% | 1.13 | 0.12 | 3.6E-01 | Red |
| WP_011605157.1 | FRAAL4026 | short-chain dehydrogenase | 28300 | 5.32 | 2 | 7 | Frankia-10 | F201373.dat | 108 | 8 | 2 | 2 | 2 | 0 | 0 | 0 | 2 | 1 | 0.004% | 0 | 0 | 1 | 1 | 2 | 0.005% | 1.13 | 0.12 | 3.6E-01 | Red |
| WP_011604494.1 | FRAAL3347 | hypothetical protein | 27251 | 5.40 | 2 | 7 | Frankia-10 | F201373.dat | 90 | 12 | 2 | 2 | 2 | 0 | 1 | 0 | 0 | 2 | 0.004% | 0 | 0 | 1 | 1 | 2 | 0.005% | 1.13 | 0.12 | 3.6E-01 | Red |
| WP_011606344.1 | FRAAL5253 | hypothetical protein | 12556 | 4.68 | 2 | 7 | Frankia-3 | F201366.dat | 75 | 29 | 2 | 2 | 2 | 0 | 0 | 2 | 0 | 1 | 0.009% | 0 | 1 | 1 | 0 | 2 | 0.011% | 1.13 | 0.12 | 3.6E-01 | Red |
| WP_041939361.1 | FRAAL3582 | branched-chain amino acid ABC transporter substrate-binding protein | 41224 | 5.96 | 3 | 7 | Frankia-7 | F201370.dat | 96 | 10 | 2 | 2 | 2 | 1 | 0 | 1 | 1 | 0 | 0.003% | 0 | 2 | 0 | 2 | 0 | 0.003% | 1.13 | 0.12 | 3.7E-01 | Red |
| WP_041939372.1 | FRAAL3617 | hypothetical protein | 133384 | 5.52 | 8 | 24 | Frankia-1 | F201364.dat | 218 | 6 | 6 | 6 | 6 | 6 | 2 | 0 | 2 | 1 | 0.003% | 3 | 2 | 5 | 0 | 3 | 0.003% | 1.13 | 0.12 | 3.8E-01 | Red |
| WP_011605674.1 | FRAAL4557 | hypothetical protein | 15779 | 3.82 | 3 | 7 | Frankia-5 | F201368.dat | 141 | 27 | 2 | 2 | 3 | 0 | 0 | 0 | 0 | 3 | 0.007% | 2 | 1 | 0 | 0 | 1 | 0.009% | 1.13 | 0.12 | 3.9E-01 | Red |
| WP_011607890.1 | FRAAL6855 | hypothetical protein | 45105 | 10.09 | 3 | 7 | Frankia-8 | F201371.dat | 101 | 11 | 3 | 3 | 3 | 2 | 0 | 0 | 0 | 1 | 0.002% | 0 | 0 | 3 | 1 | 0 | 0.003% | 1.13 | 0.12 | 3.9E-01 | Red |
| WP_011606746.1 | FRAAL5671 | 3-oxoacyl-ACP synthase | 41389 | 5.64 | 8 | 60 | Frankia-8 | F201371.dat | 338 | 24 | 5 | 5 | 8 | 6 | 7 | 6 | 4 | 5 | 0.024% | 6 | 7 | 8 | 6 | 5 | 0.026% | 1.12 | 0.11 | 1.5E-01 | Red |
| WP_041940628.1 | FRAAL3759 | haloacid dehalogenase | 84139 | 9.57 | 7 | 43 | Frankia-9 | F201372.dat | 307 | 11 | 7 | 7 | 6 | 3 | 4 | 5 | 5 | 3 | 0.008% | 5 | 3 | 4 | 6 | 5 | 0.009% | 1.12 | 0.11 | 2.0E-01 | Red |
| WP_041939852.1 | FRAAL6236 | hypothetical protein | 27702 | 9.98 | 5 | 43 | Frankia-8 | F201371.dat | 215 | 25 | 5 | 5 | 6 | 3 | 4 | 3 | 6 | 4 | 0.026% | 3 | 4 | 6 | 5 | 5 | 0.028% | 1.12 | 0.11 | 2.2E-01 | Red |
| WP_041939072.1 | FRAAL2186 | ATPase | 37290 | 6.01 | 11 | 43 | Frankia-5 | F201368.dat | 191 | 25 | 6 | 6 | 6 | 2 | 5 | 3 | 4 | 6 | 0.019% | 3 | 4 | 5 | 5 | 6 | 0.021% | 1.12 | 0.11 | 2.6E-01 | Red |
| WP_011606959.1 | FRAAL5898 | chemical-damaging agent resistance protein C | 20183 | 4.53 | 14 | 257 | Frankia-5 | F201368.dat | 887 | 71 | 13 | 12 | 29 | 21 | 22 | 22 | 27 | 29 | 0.214% | 23 | 29 | 29 | 28 | 27 | 0.227% | 1.12 | 0.11 | 8.1E-02 | Red |
| WP_011602974.1 | FRAAL1792 | glycosidase | 36958 | 8.95 | 10 | 79 | Frankia-7 | F201370.dat | 349 | 29 | 8 | 8 | 10 | 7 | 8 | 8 | 7 | 7 | 0.036% | 6 | 10 | 9 | 9 | 8 | 0.038% | 1.12 | 0.11 | 1.1E-01 | Red |
| WP_009742445.1 | FRAAL5670 | acyl carrier protein | 8515 | 3.95 | 5 | 79 | Frankia-2 | F201365.dat | 257 | 64 | 5 | 5 | 9 | 6 | 9 | 9 | 8 | 5 | 0.155% | 10 | 6 | 9 | 9 | 8 | 0.166% | 1.12 | 0.11 | 1.9E-01 | Red |
| WP_011603309.1 | FRAAL2144 | preprotein translocase subunit SecD | 65568 | 9.99 | 16 | 151 | Frankia-6 | F201369.dat | 918 | 28 | 13 | 13 | 19 | 14 | 14 | 16 | 13 | 14 | 0.039% | 19 | 15 | 14 | 18 | 14 | 0.041% | 1.12 | 0.11 | 7.9E-02 | Red |
| WP_050997446.1 | FRAAL5695 | acetoin utilization protein AcuC | 41613 | 5.47 | 4 | 26 | Frankia-10 | F201373.dat | 143 | 11 | 3 | 3 | 3 | 1 | 3 | 3 | 3 | 2 | 0.010% | 3 | 3 | 3 | 2 | 3 | 0.011% | 1.12 | 0.11 | 2.3E-01 | Red |
| WP_041938594.1 | FRAAL0026 | osmotically inducible protein C | 14691 | 4.83 | 5 | 26 | Frankia-8 | F201371.dat | 217 | 37 | 4 | 4 | 4 | 1 | 3 | 2 | 3 | 3 | 0.029% | 3 | 2 | 4 | 2 | 3 | 0.032% | 1.12 | 0.11 | 2.4E-01 | Red |
| WP_011604390.1 | FRAAL3241 | short-chain dehydrogenase | 26110 | 6.36 | 4 | 26 | Frankia-3 | F201366.dat | 241 | 28 | 4 | 4 | 4 | 2 | 2 | 4 | 2 | 2 | 0.016% | 3 | 2 | 6 | 2 | 1 | 0.018% | 1.12 | 0.11 | 3.4E-01 | Red |
| WP_011602688.1 | FRAAL1498 | peptidase | 15352 | 4.91 | 5 | 26 | Frankia-10 | F201373.dat | 150 | 43 | 5 | 5 | 6 | 1 | 3 | 3 | 3 | 2 | 0.028% | 0 | 1 | 4 | 3 | 6 | 0.031% | 1.12 | 0.11 | 3.7E-01 | Red |
| WP_011602449.1 | FRAAL1248 | glycosyl transferase family 2 | 36779 | 9.00 | 6 | 45 | Frankia-9 | F201372.dat | 257 | 13 | 5 | 5 | 6 | 5 | 5 | 3 | 4 | 4 | 0.020% | 6 | 3 | 5 | 6 | 4 | 0.022% | 1.12 | 0.11 | 2.1E-01 | Red |
| WP_041939463.1 | FRAAL4180 | hypothetical protein | 12637 | 5.01 | 10 | 102 | Frankia-9 | F201372.dat | 365 | 46 | 10 | 10 | 13 | 10 | 8 | 11 | 12 | 7 | 0.136% | 8 | 7 | 14 | 13 | 12 | 0.144% | 1.11 | 0.11 | 2.5E-01 | Red |
| WP_011601679.1 | FRAAL0423 | membrane protein | 80111 | 8.59 | 14 | 121 | Frankia-3 | F201366.dat | 563 | 25 | 13 | 13 | 14 | 14 | 12 | 14 | 10 | 7 | 0.025% | 16 | 11 | 14 | 13 | 10 | 0.027% | 1.11 | 0.11 | 2.2E-01 | Red |
| WP_011602345.1 | FRAAL1140 | inosine-5-monophosphate dehydrogenase | 38764 | 5.59 | 14 | 142 | Frankia-7 | F201370.dat | 579 | 39 | 11 | 11 | 16 | 15 | 12 | 14 | 13 | 13 | 0.062% | 14 | 16 | 17 | 15 | 13 | 0.065% | 1.11 | 0.11 | 5.2E-02 | Red |
| WP_011602315.1 | FRAAL1102 | adenylate kinase | 23589 | 4.94 | 10 | 85 | Frankia-8 | F201371.dat | 573 | 56 | 9 | 9 | 10 | 8 | 8 | 8 | 8 | 8 | 0.061% | 9 | 10 | 10 | 8 | 8 | 0.064% | 1.11 | 0.11 | 5.5E-02 | Red |
| WP_011607803.1 | FRAAL6767 | hypothetical protein | 26134 | 11.13 | 18 | 275 | Frankia-5 | F201368.dat | 1107 | 69 | 18 | 18 | 33 | 21 | 26 | 25 | 25 | 33 | 0.178% | 26 | 24 | 32 | 31 | 32 | 0.187% | 1.11 | 0.11 | 1.4E-01 | Red |
| WP_041939930.1 | FRAAL6597 | peptidase C69 | 55996 | 5.11 | 23 | 161 | Frankia-8 | F201371.dat | 870 | 40 | 16 | 16 | 22 | 16 | 13 | 16 | 15 | 16 | 0.048% | 13 | 18 | 22 | 15 | 17 | 0.051% | 1.11 | 0.11 | 1.5E-01 | Red |
| WP_041939223.1 | FRAAL2796 | urease accessory protein UreG | 24191 | 5.13 | 5 | 28 | Frankia-2 | F201365.dat | 191 | 23 | 3 | 3 | 4 | 3 | 4 | 2 | 2 | 2 | 0.019% | 3 | 3 | 3 | 2 | 4 | 0.021% | 1.11 | 0.11 | 2.3E-01 | Red |
| WP_050997313.1 | FRAAL0652 | histidine kinase | 62237 | 6.83 | 7 | 28 | Frankia-10 | F201373.dat | 150 | 10 | 5 | 5 | 5 | 4 | 2 | 2 | 3 | 2 | 0.007% | 2 | 3 | 3 | 2 | 5 | 0.008% | 1.11 | 0.11 | 2.9E-01 | Red |
| WP_011605158.1 | FRAAL4027 | dihydrodipicolinate reductase | 37433 | 4.66 | 2 | 9 | Frankia-7 | F201370.dat | 112 | 7 | 2 | 2 | 2 | 1 | 1 | 1 | 0 | 1 | 0.004% | 1 | 2 | 0 | 1 | 1 | 0.004% | 1.11 | 0.11 | 3.4E-01 | Red |
| WP_011602395.1 | FRAAL1192 | serine/threonine protein kinase | 117378 | 5.81 | 2 | 9 | Frankia-5 | F201368.dat | 87 | 2 | 2 | 2 | 1 | 1 | 1 | 0 | 1 | 1 | 0.001% | 1 | 0 | 1 | 2 | 1 | 0.001% | 1.11 | 0.11 | 3.4E-01 | Red |
| WP_011604635.1 | FRAAL3492 | DNA-binding protein | 18005 | 5.14 | 2 | 9 | Frankia-8 | F201371.dat | 78 | 18 | 2 | 2 | 2 | 1 | 1 | 0 | 1 | 1 | 0.008% | 0 | 1 | 2 | 1 | 1 | 0.009% | 1.11 | 0.11 | 3.4E-01 | Red |
| WP_011605872.1 | FRAAL4757 | aldehyde dehydrogenase | 50808 | 5.59 | 2 | 9 | Frankia-10 | F201373.dat | 60 | 6 | 2 | 2 | 2 | 1 | 0 | 1 | 1 | 1 | 0.003% | 1 | 0 | 1 | 1 | 2 | 0.003% | 1.11 | 0.11 | 3.4E-01 | Red |
| WP_011601808.1 | FRAAL0555 | epoxide hydrolase | 40621 | 6.06 | 4 | 9 | Frankia-10 | F201373.dat | 82 | 9 | 2 | 2 | 2 | 0 | 0 | 1 | 1 | 2 | 0.004% | 0 | 1 | 1 | 1 | 2 | 0.004% | 1.11 | 0.11 | 3.5E-01 | Red |
| WP_011605613.1 | FRAAL4492 | alkyl hydroperoxide reductase | 20259 | 4.30 | 3 | 9 | Frankia-2 | F201365.dat | 104 | 24 | 2 | 2 | 2 | 0 | 2 | 0 | 1 | 1 | 0.007% | 1 | 2 | 1 | 1 | 0 | 0.008% | 1.11 | 0.11 | 3.5E-01 | Red |
| WP_041939823.1 | FRAAL6083 | metal-dependent phosphoesterase | 29951 | 5.86 | 3 | 9 | Frankia-8 | F201371.dat | 64 | 11 | 2 | 2 | 2 | 1 | 1 | 0 | 0 | 2 | 0.005% | 1 | 1 | 2 | 0 | 1 | 0.006% | 1.11 | 0.11 | 3.5E-01 | Red |
| WP_011603602.1 | FRAAL2445 | hypothetical protein. partial | 82167 | 6.17 | 3 | 9 | Frankia-5 | F201368.dat | 105 | 4 | 2 | 2 | 2 | 0 | 1 | 1 | 0 | 2 | 0.002% | 0 | 0 | 1 | 2 | 2 | 0.002% | 1.11 | 0.11 | 3.7E-01 | Red |
| WP_041940782.1 | FRAAL4597 | deaminase/reductase | 24958 | 5.09 | 2 | 9 | Frankia-9 | F201372.dat | 84 | 10 | 2 | 2 | 3 | 0 | 1 | 1 | 1 | 1 | 0.006% | 1 | 0 | 0 | 3 | 1 | 0.007% | 1.11 | 0.11 | 3.8E-01 | Red |
| WP_011602247.1 | FRAAL1020 | uroporphyrinogen-III C-methyltransferase | 52021 | 5.39 | 3 | 9 | Frankia-10 | F201373.dat | 127 | 8 | 3 | 3 | 3 | 0 | 1 | 1 | 2 | 0 | 0.003% | 0 | 0 | 0 | 2 | 3 | 0.003% | 1.11 | 0.11 | 4.0E-01 | Red |
| WP_011606090.1 | FRAAL4976 | imidazole glycerol phosphate synthase subunit HisH | 26624 | 4.91 | 5 | 9 | Frankia-8 | F201371.dat | 77 | 12 | 3 | 3 | 3 | 0 | 0 | 0 | 2 | 2 | 0.005% | 0 | 2 | 3 | 0 | 0 | 0.006% | 1.11 | 0.11 | 4.0E-01 | Red |
| WP_011604377.1 | FRAAL3228 | pyruvate dehydrogenase E1 subunit alpha | 41557 | 5.32 | 9 | 68 | Frankia-8 | F201371.dat | 283 | 19 | 7 | 7 | 9 | 7 | 5 | 8 | 7 | 5 | 0.027% | 7 | 7 | 9 | 6 | 7 | 0.029% | 1.11 | 0.10 | 1.7E-01 | Red |
| WP_011605166.1 | FRAAL4035 | hypothetical protein | 31103 | 4.81 | 17 | 207 | Frankia-8 | F201371.dat | 891 | 64 | 15 | 14 | 27 | 19 | 17 | 19 | 19 | 24 | 0.112% | 20 | 17 | 27 | 21 | 24 | 0.118% | 1.11 | 0.10 | 1.6E-01 | Red |
| WP_011605946.1 | FRAAL4831 | hypothetical protein | 33200 | 5.33 | 13 | 89 | Frankia-8 | F201371.dat | 516 | 48 | 10 | 7 | 10 | 9 | 8 | 7 | 7 | 11 | 0.045% | 10 | 7 | 10 | 8 | 12 | 0.048% | 1.11 | 0.10 | 2.0E-01 | Red |
| WP_011602256.1 | FRAAL1029 | carboxymethylenebutenolidase | 25220 | 4.67 | 4 | 30 | Frankia-8 | F201371.dat | 199 | 12 | 3 | 3 | 3 | 2 | 4 | 3 | 3 | 2 | 0.020% | 3 | 4 | 3 | 3 | 3 | 0.021% | 1.11 | 0.10 | 2.2E-01 | Red |
| WP_011603287.1 | FRAAL2122 | dUTPase | 10719 | 4.30 | 5 | 30 | Frankia-10 | F201373.dat | 281 | 75 | 4 | 4 | 4 | 2 | 3 | 3 | 4 | 2 | 0.047% | 4 | 3 | 2 | 3 | 4 | 0.050% | 1.11 | 0.10 | 2.4E-01 | Red |
| WP_011604006.1 | FRAAL2860 | carboxylate-amine ligase | 30243 | 4.80 | 5 | 30 | Frankia-8 | F201371.dat | 203 | 26 | 4 | 4 | 5 | 3 | 3 | 3 | 1 | 4 | 0.017% | 2 | 2 | 5 | 3 | 4 | 0.018% | 1.11 | 0.10 | 3.1E-01 | Red |
| WP_011602072.1 | FRAAL0837 | hypothetical protein | 37211 | 5.45 | 12 | 131 | Frankia-7 | F201370.dat | 572 | 38 | 11 | 11 | 17 | 14 | 13 | 14 | 11 | 10 | 0.059% | 14 | 17 | 13 | 15 | 10 | 0.062% | 1.10 | 0.10 | 1.8E-01 | Red |
| WP_041938927.1 | FRAAL1487 | transporter | 16136 | 5.14 | 3 | 51 | Frankia-8 | F201371.dat | 170 | 20 | 3 | 3 | 6 | 5 | 4 | 5 | 5 | 5 | 0.053% | 4 | 5 | 6 | 7 | 5 | 0.056% | 1.10 | 0.10 | 1.8E-01 | Red |
| WP_041939080.1 | FRAAL2221 | acid phosphatase | 40625 | 5.70 | 19 | 156 | Frankia-7 | F201370.dat | 631 | 50 | 15 | 15 | 20 | 16 | 11 | 16 | 16 | 15 | 0.065% | 14 | 20 | 17 | 17 | 14 | 0.068% | 1.10 | 0.10 | 1.6E-01 | Red |
| WP_041940973.1 | FRAAL5653 | succinate dehydrogenase | 23379 | 9.88 | 2 | 32 | Frankia-6 | F201369.dat | 103 | 10 | 2 | 2 | 5 | 4 | 3 | 4 | 2 | 2 | 0.023% | 5 | 3 | 3 | 3 | 3 | 0.024% | 1.10 | 0.10 | 2.6E-01 | Red |
| WP_011603047.1 | FRAAL1870 | glycosyl transferase family 1 | 62398 | 10.29 | 11 | 53 | Frankia-1 | F201364.dat | 355 | 18 | 8 | 8 | 8 | 8 | 3 | 4 | 6 | 4 | 0.014% | 6 | 4 | 7 | 7 | 4 | 0.015% | 1.10 | 0.10 | 3.0E-01 | Red |
| WP_011607377.1 | FRAAL6329 | DNA helicase | 78498 | 6.25 | 22 | 95 | Frankia-8 | F201371.dat | 500 | 20 | 13 | 13 | 15 | 13 | 7 | 5 | 10 | 10 | 0.020% | 10 | 9 | 15 | 7 | 9 | 0.021% | 1.10 | 0.10 | 3.1E-01 | Red |
| WP_011606049.1 | FRAAL4935 | TetR family transcriptional regulator | 22716 | 8.02 | 2 | 11 | Frankia-7 | F201370.dat | 109 | 9 | 2 | 2 | 2 | 1 | 1 | 1 | 1 | 1 | 0.008% | 1 | 2 | 1 | 1 | 1 | 0.009% | 1.10 | 0.10 | 3.4E-01 | Red |
| WP_011601885.1 | FRAAL0636 | tRNA adenosine deaminase | 17618 | 3.86 | 2 | 11 | Frankia-10 | F201373.dat | 97 | 12 | 2 | 2 | 2 | 1 | 1 | 1 | 1 | 1 | 0.010% | 1 | 1 | 1 | 1 | 2 | 0.011% | 1.10 | 0.10 | 3.4E-01 | Red |
| WP_011605253.1 | FRAAL4125 | pyridoxamine 5-phosphate oxidase | 17217 | 5.49 | 2 | 11 | Frankia-9 | F201372.dat | 96 | 13 | 2 | 2 | 2 | 1 | 1 | 1 | 1 | 1 | 0.010% | 1 | 1 | 1 | 2 | 1 | 0.012% | 1.10 | 0.10 | 3.4E-01 | Red |
| WP_011603112.1 | FRAAL1939 | phosphoglycerate mutase | 25326 | 6.39 | 3 | 11 | Frankia-8 | F201371.dat | 96 | 11 | 2 | 2 | 2 | 0 | 1 | 1 | 2 | 1 | 0.007% | 0 | 1 | 2 | 2 | 1 | 0.008% | 1.10 | 0.10 | 3.5E-01 | Red |
| WP_011601764.1 | FRAAL0511 | DNA hydrolase | 30407 | 6.07 | 3 | 11 | Frankia-9 | F201372.dat | 64 | 7 | 2 | 2 | 2 | 1 | 1 | 1 | 1 | 1 | 0.006% | 0 | 1 | 1 | 2 | 2 | 0.007% | 1.10 | 0.10 | 3.5E-01 | Red |
| WP_041939380.1 | FRAAL3694 | methyltransferase | 23995 | 6.22 | 2 | 11 | Frankia-9 | F201372.dat | 67 | 13 | 2 | 2 | 2 | 1 | 1 | 1 | 1 | 1 | 0.007% | 0 | 1 | 2 | 2 | 1 | 0.008% | 1.10 | 0.10 | 3.5E-01 | Red |
| WP_041939669.1 | FRAAL5287 | glycosyl transferase family 1 | 48391 | 9.09 | 3 | 11 | Frankia-3 | F201366.dat | 66 | 4 | 2 | 2 | 2 | 2 | 1 | 2 | 0 | 0 | 0.004% | 2 | 2 | 1 | 0 | 1 | 0.004% | 1.10 | 0.10 | 3.7E-01 | Red |
| WP_011606276.1 | FRAAL5173 | oxidoreductase | 23292 | 5.73 | 11 | 162 | Frankia-10 | F201373.dat | 569 | 48 | 10 | 10 | 19 | 16 | 14 | 15 | 16 | 16 | 0.118% | 11 | 18 | 19 | 18 | 19 | 0.123% | 1.10 | 0.09 | 1.7E-01 | Red |
| WP_041940251.1 | FRAAL1615 | zf-TFIIB domain containing protein | 11005 | 7.80 | 3 | 55 | Frankia-7 | F201370.dat | 201 | 30 | 3 | 3 | 5 | 4 | 6 | 3 | 7 | 6 | 0.084% | 6 | 5 | 5 | 6 | 7 | 0.089% | 1.10 | 0.09 | 2.4E-01 | Red |
| WP_009742788.1 | FRAAL5240 | XRE family transcriptional regulator | 17998 | 5.75 | 16 | 273 | Frankia-5 | F201368.dat | 677 | 74 | 14 | 14 | 30 | 25 | 28 | 21 | 26 | 30 | 0.258% | 23 | 28 | 26 | 34 | 32 | 0.268% | 1.10 | 0.09 | 1.6E-01 | Red |
| WP_011607442.1 | FRAAL6397 | UTP--glucose-1-phosphate uridylyltransferase | 33514 | 5.17 | 17 | 99 | Frankia-4 | F201367.dat | 503 | 48 | 11 | 11 | 11 | 8 | 9 | 9 | 11 | 10 | 0.050% | 8 | 11 | 12 | 10 | 11 | 0.052% | 1.10 | 0.09 | 1.4E-01 | Red |
| WP_011602255.1 | FRAAL1028 | ubiquinone/menaquinone biosynthesis methyltransferase | 30220 | 8.53 | 11 | 78 | Frankia-7 | F201370.dat | 473 | 35 | 8 | 8 | 10 | 4 | 7 | 8 | 9 | 9 | 0.044% | 9 | 10 | 6 | 9 | 7 | 0.046% | 1.10 | 0.09 | 2.6E-01 | Red |
| WP_009740521.1 | FRAAL1088 | 50S ribosomal protein L16 | 15426 | 11.27 | 6 | 82 | Frankia-8 | F201371.dat | 377 | 49 | 5 | 5 | 11 | 7 | 6 | 9 | 7 | 10 | 0.090% | 7 | 8 | 11 | 9 | 8 | 0.094% | 1.09 | 0.09 | 2.2E-01 | Red |
| WP_041940785.1 | FRAAL4610 | peptidase M4 | 37594 | 4.84 | 11 | 59 | Frankia-8 | F201371.dat | 302 | 27 | 9 | 9 | 10 | 6 | 5 | 6 | 4 | 7 | 0.027% | 4 | 6 | 10 | 5 | 6 | 0.028% | 1.09 | 0.09 | 3.1E-01 | Red |
| WP_050997252.1 | FRAAL5889 | phosphoribosyl transferase | 52688 | 5.94 | 5 | 13 | Frankia-7 | F201370.dat | 121 | 5 | 2 | 2 | 2 | 2 | 1 | 1 | 1 | 1 | 0.004% | 2 | 2 | 1 | 1 | 1 | 0.004% | 1.09 | 0.09 | 3.4E-01 | Red |
| WP_011606170.1 | FRAAL5060 | cytotoxic translational repressor of toxin-antitoxin stability system | 11673 | 11.02 | 3 | 13 | Frankia-7 | F201370.dat | 111 | 22 | 2 | 2 | 2 | 1 | 1 | 1 | 2 | 1 | 0.018% | 1 | 2 | 2 | 1 | 1 | 0.020% | 1.09 | 0.09 | 3.4E-01 | Red |
| WP_009739884.1 | FRAAL1848 | HNH endonuclease | 18712 | 10.33 | 3 | 13 | Frankia-9 | F201372.dat | 91 | 12 | 2 | 2 | 2 | 1 | 1 | 1 | 2 | 1 | 0.011% | 1 | 1 | 2 | 2 | 1 | 0.013% | 1.09 | 0.09 | 3.4E-01 | Red |
| WP_011606877.1 | FRAAL5805 | phosphate starvation protein PhoH | 36305 | 6.15 | 2 | 13 | Frankia-7 | F201370.dat | 78 | 6 | 2 | 2 | 2 | 1 | 1 | 1 | 2 | 1 | 0.006% | 2 | 2 | 1 | 1 | 1 | 0.006% | 1.09 | 0.09 | 3.4E-01 | Red |
| WP_041939892.1 | FRAAL6427 | membrane protein | 83256 | 8.56 | 4 | 13 | Frankia-1 | F201364.dat | 175 | 4 | 2 | 2 | 2 | 2 | 1 | 1 | 2 | 0 | 0.003% | 1 | 1 | 2 | 2 | 1 | 0.003% | 1.09 | 0.09 | 3.5E-01 | Red |
| WP_011602719.1 | FRAAL1529 | hypothetical protein | 14610 | 11.76 | 3 | 13 | Frankia-8 | F201371.dat | 153 | 26 | 3 | 3 | 3 | 2 | 1 | 1 | 1 | 1 | 0.015% | 1 | 1 | 3 | 1 | 1 | 0.016% | 1.09 | 0.09 | 3.6E-01 | Red |
| WP_011606815.1 | FRAAL5741 | acetate kinase | 37331 | 5.76 | 2 | 13 | Frankia-3 | F201366.dat | 87 | 11 | 2 | 2 | 2 | 1 | 1 | 2 | 1 | 1 | 0.006% | 0 | 2 | 2 | 2 | 1 | 0.006% | 1.09 | 0.09 | 3.6E-01 | Red |
| WP_050997395.1 | FRAAL3908 | acetyltransferase | 16773 | 6.58 | 2 | 13 | Frankia-8 | F201371.dat | 71 | 16 | 2 | 2 | 3 | 1 | 1 | 1 | 1 | 2 | 0.013% | 1 | 1 | 3 | 1 | 1 | 0.014% | 1.09 | 0.09 | 3.6E-01 | Red |
| WP_041939010.1 | FRAAL1869 | hypothetical protein | 51527 | 9.96 | 4 | 13 | Frankia-1 | F201364.dat | 117 | 6 | 3 | 3 | 2 | 2 | 2 | 2 | 0 | 0 | 0.004% | 1 | 2 | 2 | 1 | 1 | 0.005% | 1.09 | 0.09 | 3.7E-01 | Red |
| WP_050997351.1 | FRAAL2311 | formyltetrahydrofolate deformylase | 33163 | 6.33 | 3 | 13 | Frankia-9 | F201372.dat | 86 | 8 | 2 | 2 | 2 | 2 | 1 | 0 | 2 | 1 | 0.006% | 0 | 0 | 2 | 2 | 3 | 0.007% | 1.09 | 0.09 | 3.9E-01 | Red |
| WP_011601474.1 | FRAAL0215 | hypothetical protein | 38878 | 6.81 | 4 | 13 | Frankia-8 | F201371.dat | 151 | 9 | 3 | 3 | 3 | 0 | 1 | 3 | 0 | 2 | 0.006% | 1 | 2 | 3 | 1 | 0 | 0.006% | 1.09 | 0.09 | 4.0E-01 | Red |
| WP_041939519.1 | FRAAL4488 | asparagine synthase | 64564 | 5.65 | 28 | 253 | Frankia-8 | F201371.dat | 1105 | 47 | 24 | 24 | 35 | 30 | 21 | 25 | 24 | 21 | 0.067% | 18 | 29 | 35 | 23 | 27 | 0.069% | 1.09 | 0.08 | 2.6E-01 | Red |
| WP_011602244.1 | FRAAL1017 | sulfite reductase | 64023 | 5.93 | 15 | 86 | Frankia-8 | F201371.dat | 540 | 27 | 12 | 12 | 13 | 12 | 8 | 7 | 5 | 9 | 0.023% | 7 | 8 | 13 | 8 | 9 | 0.024% | 1.09 | 0.08 | 3.1E-01 | Red |
| WP_011606197.1 | FRAAL5088 | esterase | 28061 | 5.63 | 6 | 38 | Frankia-8 | F201371.dat | 251 | 35 | 6 | 6 | 7 | 5 | 4 | 2 | 3 | 4 | 0.023% | 3 | 3 | 7 | 3 | 4 | 0.024% | 1.09 | 0.08 | 3.4E-01 | Red |
| WP_041938957.1 | FRAAL1641 | enoyl-CoA hydratase | 26889 | 5.65 | 9 | 38 | Frankia-8 | F201371.dat | 364 | 41 | 7 | 7 | 7 | 3 | 5 | 3 | 3 | 4 | 0.024% | 2 | 2 | 7 | 4 | 5 | 0.025% | 1.09 | 0.08 | 3.5E-01 | Red |
| WP_011602690.1 | FRAAL1500 | cysteine synthase B | 34208 | 5.24 | 8 | 63 | Frankia-10 | F201373.dat | 317 | 26 | 7 | 7 | 8 | 7 | 6 | 5 | 6 | 6 | 0.031% | 4 | 7 | 7 | 7 | 8 | 0.032% | 1.09 | 0.08 | 2.2E-01 | Red |
| WP_011605465.1 | FRAAL4342 | hypothetical protein | 23736 | 7.78 | 10 | 88 | Frankia-8 | F201371.dat | 410 | 41 | 9 | 9 | 12 | 9 | 9 | 6 | 9 | 9 | 0.063% | 8 | 8 | 12 | 10 | 8 | 0.065% | 1.09 | 0.08 | 2.2E-01 | Red |
| WP_011607670.1 | FRAAL6633 | glycosyl hydrolase | 92375 | 5.28 | 25 | 138 | Frankia-10 | F201373.dat | 728 | 23 | 16 | 16 | 17 | 15 | 14 | 10 | 12 | 15 | 0.025% | 14 | 12 | 17 | 12 | 17 | 0.026% | 1.08 | 0.08 | 2.2E-01 | Red |
| WP_011607252.1 | FRAAL6202 | ATP-binding protein | 46438 | 5.64 | 12 | 90 | Frankia-7 | F201370.dat | 421 | 25 | 9 | 9 | 11 | 9 | 8 | 8 | 9 | 9 | 0.033% | 8 | 11 | 10 | 10 | 8 | 0.034% | 1.08 | 0.08 | 1.4E-01 | Red |
| WP_011604668.1 | FRAAL3527 | branched-chain amino acid ABC transporter substrate-binding protein | 44914 | 9.30 | 18 | 165 | Frankia-7 | F201370.dat | 793 | 47 | 14 | 14 | 21 | 14 | 18 | 17 | 16 | 14 | 0.063% | 18 | 21 | 13 | 18 | 16 | 0.064% | 1.08 | 0.08 | 2.0E-01 | Red |
| WP_041940977.1 | FRAAL5669 | 3-oxoacyl-ACP synthase | 32268 | 4.90 | 8 | 90 | Frankia-1 | F201364.dat | 414 | 27 | 7 | 7 | 9 | 9 | 7 | 8 | 9 | 10 | 0.048% | 9 | 11 | 11 | 7 | 9 | 0.049% | 1.08 | 0.08 | 2.0E-01 | Red |
| WP_011605536.1 | FRAAL4413 | aldehyde dehydrogenase | 57615 | 6.05 | 9 | 40 | Frankia-6 | F201369.dat | 279 | 15 | 5 | 5 | 5 | 4 | 3 | 5 | 3 | 4 | 0.012% | 5 | 4 | 4 | 3 | 5 | 0.012% | 1.08 | 0.08 | 2.4E-01 | Red |
| WP_011604385.1 | FRAAL3236 | nitroreductase | 15774 | 5.03 | 2 | 15 | Frankia-9 | F201372.dat | 149 | 18 | 2 | 2 | 2 | 1 | 1 | 2 | 1 | 2 | 0.016% | 2 | 1 | 2 | 2 | 1 | 0.017% | 1.08 | 0.08 | 3.4E-01 | Red |
| WP_041939106.1 | FRAAL2332 | precorrin-8X methylmutase | 22138 | 5.27 | 2 | 15 | Frankia-10 | F201373.dat | 120 | 13 | 2 | 2 | 2 | 1 | 2 | 1 | 2 | 1 | 0.011% | 2 | 1 | 2 | 1 | 2 | 0.012% | 1.08 | 0.08 | 3.4E-01 | Red |
| WP_011603279.1 | FRAAL2114 | ATPase | 37437 | 9.15 | 2 | 15 | Frankia-7 | F201370.dat | 79 | 9 | 2 | 2 | 2 | 1 | 2 | 2 | 1 | 1 | 0.007% | 2 | 2 | 1 | 2 | 1 | 0.007% | 1.08 | 0.08 | 3.4E-01 | Red |
| WP_041938885.1 | FRAAL1260 | 2-phospho-L-lactate transferase | 35985 | 4.69 | 4 | 15 | Frankia-9 | F201372.dat | 69 | 12 | 3 | 3 | 3 | 0 | 1 | 2 | 2 | 2 | 0.007% | 0 | 1 | 2 | 3 | 2 | 0.007% | 1.08 | 0.08 | 3.8E-01 | Red |
| WP_011602459.1 | FRAAL1258 | hypothetical protein | 28078 | 9.10 | 11 | 167 | Frankia-2 | F201365.dat | 572 | 53 | 10 | 10 | 16 | 15 | 16 | 16 | 15 | 18 | 0.102% | 18 | 20 | 16 | 17 | 16 | 0.104% | 1.08 | 0.08 | 8.5E-02 | Red |
| WP_050997070.1 | FRAAL2455 | hypothetical protein | 132467 | 6.09 | 52 | 475 | Frankia-1 | F201364.dat | 1865 | 41 | 42 | 42 | 55 | 55 | 43 | 44 | 44 | 42 | 0.061% | 46 | 39 | 57 | 50 | 55 | 0.063% | 1.08 | 0.08 | 1.9E-01 | Red |
| WP_011605750.1 | FRAAL4634 | tryptophan halogenase | 55316 | 5.04 | 14 | 94 | Frankia-8 | F201371.dat | 612 | 29 | 11 | 11 | 12 | 10 | 7 | 9 | 11 | 8 | 0.029% | 8 | 11 | 12 | 9 | 9 | 0.030% | 1.08 | 0.08 | 2.3E-01 | Red |
| WP_011607515.1 | FRAAL6473 | hypothetical protein | 18100 | 9.88 | 4 | 42 | Frankia-8 | F201371.dat | 226 | 33 | 4 | 4 | 7 | 3 | 4 | 5 | 4 | 4 | 0.039% | 2 | 4 | 7 | 4 | 5 | 0.041% | 1.08 | 0.08 | 3.3E-01 | Red |
| WP_011607682.1 | FRAAL6645 | pseudouridine-5'-phosphate glycosidase | 36931 | 4.80 | 7 | 42 | Frankia-8 | F201371.dat | 337 | 24 | 6 | 6 | 6 | 6 | 2 | 5 | 3 | 4 | 0.019% | 2 | 5 | 6 | 4 | 5 | 0.020% | 1.08 | 0.08 | 3.5E-01 | Red |
| WP_011602618.1 | FRAAL1423 | hypothetical protein | 48785 | 5.65 | 8 | 42 | Frankia-7 | F201370.dat | 258 | 17 | 5 | 5 | 5 | 5 | 2 | 4 | 4 | 5 | 0.015% | 1 | 5 | 6 | 5 | 5 | 0.015% | 1.08 | 0.08 | 3.5E-01 | Red |
| WP_041939641.1 | FRAAL5122 | cytochrome C oxidase subunit I | 62503 | 8.90 | 10 | 69 | Frankia-3 | F201366.dat | 294 | 14 | 7 | 5 | 8 | 5 | 6 | 8 | 8 | 6 | 0.019% | 7 | 7 | 7 | 8 | 7 | 0.019% | 1.08 | 0.08 | 2.0E-01 | Red |
| WP_011606316.1 | FRAAL5221 | riboflavin synthase subunit alpha | 25232 | 4.86 | 9 | 69 | Frankia-5 | F201368.dat | 327 | 42 | 6 | 6 | 8 | 6 | 6 | 7 | 6 | 8 | 0.047% | 5 | 8 | 7 | 7 | 9 | 0.048% | 1.08 | 0.08 | 2.3E-01 | Red |
| WP_041940333.1 | FRAAL2107 | MFS transporter | 28345 | 5.32 | 6 | 44 | Frankia-8 | F201371.dat | 194 | 23 | 4 | 4 | 5 | 5 | 5 | 4 | 4 | 3 | 0.026% | 4 | 5 | 5 | 5 | 4 | 0.027% | 1.08 | 0.07 | 2.2E-01 | Red |
| WP_011601319.1 | FRAAL0051 | serine/threonine protein kinase | 89301 | 6.22 | 9 | 44 | Frankia-10 | F201373.dat | 283 | 10 | 6 | 6 | 6 | 5 | 4 | 4 | 5 | 3 | 0.008% | 3 | 5 | 5 | 4 | 6 | 0.009% | 1.08 | 0.07 | 2.7E-01 | Red |
| WP_011607241.1 | FRAAL6191 | hypothetical protein | 19135 | 10.11 | 5 | 71 | Frankia-10 | F201373.dat | 196 | 38 | 4 | 4 | 7 | 6 | 8 | 5 | 8 | 7 | 0.063% | 6 | 10 | 4 | 10 | 7 | 0.065% | 1.08 | 0.07 | 3.3E-01 | Red |
| WP_011605623.1 | FRAAL4504 | ferritin | 14890 | 4.45 | 4 | 17 | Frankia-8 | F201371.dat | 155 | 29 | 3 | 3 | 3 | 2 | 1 | 1 | 2 | 2 | 0.019% | 1 | 1 | 3 | 2 | 2 | 0.020% | 1.08 | 0.07 | 3.5E-01 | Red |
| WP_011607044.1 | FRAAL5989 | dTDP-4-dehydrorhamnose 3.5-epimerase | 22479 | 4.94 | 4 | 17 | Frankia-10 | F201373.dat | 145 | 24 | 3 | 3 | 3 | 2 | 1 | 2 | 2 | 1 | 0.013% | 1 | 2 | 2 | 1 | 3 | 0.013% | 1.08 | 0.07 | 3.5E-01 | Red |
| WP_041939479.1 | FRAAL4289 | acetyltransferase | 23413 | 6.92 | 6 | 17 | Frankia-4 | F201367.dat | 82 | 30 | 3 | 3 | 3 | 0 | 1 | 2 | 3 | 2 | 0.012% | 2 | 2 | 1 | 2 | 2 | 0.013% | 1.08 | 0.07 | 3.8E-01 | Red |
| WP_011607703.1 | FRAAL6665 | adenylosuccinate lyase | 46849 | 5.89 | 19 | 154 | Frankia-9 | F201372.dat | 593 | 38 | 13 | 13 | 17 | 14 | 13 | 16 | 15 | 16 | 0.056% | 13 | 18 | 18 | 17 | 14 | 0.058% | 1.08 | 0.07 | 1.7E-01 | Red |
| WP_050997390.1 | FRAAL3765 | ABC transporter substrate-binding protein | 42091 | 9.11 | 23 | 237 | Frankia-7 | F201370.dat | 971 | 53 | 18 | 18 | 29 | 22 | 20 | 26 | 23 | 23 | 0.097% | 23 | 29 | 24 | 25 | 22 | 0.098% | 1.08 | 0.07 | 1.4E-01 | Red |
| WP_041939065.1 | FRAAL2153 | seryl-tRNA synthetase | 46974 | 5.49 | 23 | 212 | Frankia-8 | F201371.dat | 966 | 59 | 20 | 20 | 27 | 21 | 17 | 24 | 22 | 18 | 0.077% | 14 | 24 | 27 | 21 | 24 | 0.079% | 1.07 | 0.07 | 2.7E-01 | Red |
| WP_011606032.1 | FRAAL4918 | acyl-CoA synthetase | 50825 | 5.73 | 6 | 46 | Frankia-1 | F201364.dat | 243 | 16 | 6 | 6 | 6 | 6 | 4 | 5 | 3 | 4 | 0.015% | 5 | 5 | 5 | 4 | 5 | 0.016% | 1.07 | 0.07 | 2.6E-01 | Red |
| WP_011606115.1 | FRAAL5003 | aspartate aminotransferase | 42387 | 5.50 | 11 | 46 | Frankia-8 | F201371.dat | 261 | 21 | 7 | 7 | 7 | 4 | 5 | 4 | 4 | 5 | 0.019% | 4 | 5 | 7 | 5 | 3 | 0.019% | 1.07 | 0.07 | 3.0E-01 | Red |
| WP_011607791.1 | FRAAL6755 | serine/threonine protein kinase | 72834 | 5.11 | 20 | 187 | Frankia-8 | F201371.dat | 794 | 33 | 15 | 15 | 18 | 22 | 16 | 20 | 17 | 15 | 0.044% | 19 | 22 | 18 | 20 | 18 | 0.045% | 1.07 | 0.07 | 1.9E-01 | Red |
| WP_011606865.1 | FRAAL5793 | signal recognition particle protein | 54354 | 9.68 | 24 | 216 | Frankia-10 | F201373.dat | 969 | 41 | 18 | 18 | 25 | 19 | 19 | 23 | 21 | 22 | 0.068% | 17 | 24 | 24 | 22 | 25 | 0.069% | 1.07 | 0.07 | 1.8E-01 | Red |
| WP_050997198.1 | FRAAL4775 | SnoaL-like polyketide cyclase | 26308 | 5.13 | 17 | 394 | Frankia-10 | F201373.dat | 953 | 64 | 15 | 15 | 46 | 38 | 35 | 46 | 37 | 34 | 0.258% | 33 | 39 | 47 | 39 | 46 | 0.261% | 1.07 | 0.07 | 2.1E-01 | Red |
| WP_011607734.1 | FRAAL6697 | trehalose phosphatase | 28784 | 5.02 | 11 | 106 | Frankia-5 | F201368.dat | 660 | 54 | 9 | 9 | 11 | 10 | 8 | 11 | 11 | 11 | 0.063% | 9 | 10 | 14 | 10 | 12 | 0.064% | 1.07 | 0.07 | 2.4E-01 | Red |
| WP_041938853.1 | FRAAL1118 | SWIM-type zinc finger-containing protein | 19816 | 4.20 | 4 | 19 | Frankia-7 | F201370.dat | 182 | 27 | 3 | 3 | 3 | 3 | 2 | 1 | 2 | 1 | 0.016% | 2 | 3 | 1 | 2 | 2 | 0.017% | 1.07 | 0.07 | 3.5E-01 | Red |
| WP_011603025.1 | FRAAL1846 | serine phosphatase | 78464 | 5.73 | 3 | 19 | Frankia-8 | F201371.dat | 158 | 5 | 3 | 3 | 3 | 2 | 2 | 2 | 1 | 2 | 0.004% | 1 | 1 | 3 | 2 | 3 | 0.004% | 1.07 | 0.07 | 3.6E-01 | Red |
| WP_041940145.1 | FRAAL0881 | methyltransferase | 30859 | 5.16 | 3 | 19 | Frankia-8 | F201371.dat | 264 | 18 | 6 | 2 | 2 | 1 | 2 | 2 | 2 | 2 | 0.010% | 5 | 1 | 2 | 1 | 1 | 0.011% | 1.07 | 0.07 | 4.1E-01 | Red |
| WP_050997143.1 | FRAAL3673 | hypothetical protein | 38436 | 5.91 | 8 | 19 | Frankia-7 | F201370.dat | 306 | 26 | 6 | 5 | 6 | 2 | 2 | 3 | 1 | 1 | 0.008% | 0 | 6 | 0 | 1 | 3 | 0.009% | 1.07 | 0.07 | 4.4E-01 | Red |
| WP_011604979.1 | FRAAL3843 | hypothetical protein | 52812 | 6.21 | 10 | 50 | Frankia-9 | F201372.dat | 353 | 15 | 6 | 6 | 6 | 4 | 6 | 5 | 5 | 4 | 0.016% | 5 | 4 | 5 | 6 | 6 | 0.017% | 1.07 | 0.07 | 2.4E-01 | Red |
| WP_011603486.1 | FRAAL2322 | ATP/GTP-binding protein | 145220 | 5.47 | 30 | 143 | Frankia-8 | F201371.dat | 1016 | 22 | 20 | 20 | 21 | 18 | 13 | 14 | 11 | 13 | 0.017% | 16 | 15 | 21 | 10 | 12 | 0.017% | 1.07 | 0.07 | 3.3E-01 | Red |
| WP_011604659.1 | FRAAL3518 | short-chain dehydrogenase | 26059 | 5.68 | 18 | 298 | Frankia-10 | F201373.dat | 999 | 83 | 16 | 16 | 33 | 28 | 30 | 30 | 25 | 31 | 0.197% | 30 | 28 | 34 | 29 | 33 | 0.199% | 1.07 | 0.06 | 1.2E-01 | Red |
| WP_050997320.1 | FRAAL0986 | delta-aminolevulinic acid dehydratase | 33172 | 4.58 | 7 | 52 | Frankia-8 | F201371.dat | 254 | 24 | 5 | 5 | 8 | 4 | 5 | 4 | 6 | 6 | 0.027% | 3 | 6 | 8 | 5 | 5 | 0.027% | 1.07 | 0.06 | 3.4E-01 | Red |
| WP_011606987.1 | FRAAL5927 | cob(I)yrinic acid a.c-diamide adenosyltransferase | 20270 | 5.01 | 2 | 21 | Frankia-3 | F201366.dat | 187 | 13 | 2 | 2 | 2 | 2 | 2 | 2 | 2 | 2 | 0.018% | 2 | 2 | 3 | 2 | 2 | 0.018% | 1.07 | 0.06 | 3.4E-01 | Red |
| WP_009739720.1 | FRAAL2026 | DNA-directed RNA polymerase sigma-70 factor | 44415 | 5.16 | 21 | 209 | Frankia-1 | F201364.dat | 715 | 41 | 17 | 17 | 26 | 26 | 20 | 20 | 19 | 16 | 0.081% | 16 | 22 | 23 | 23 | 24 | 0.082% | 1.07 | 0.06 | 2.7E-01 | Red |
| WP_011603626.1 | FRAAL2469 | hypothetical protein | 38555 | 4.63 | 7 | 54 | Frankia-7 | F201370.dat | 303 | 24 | 6 | 6 | 7 | 6 | 5 | 5 | 5 | 5 | 0.024% | 5 | 7 | 6 | 4 | 6 | 0.024% | 1.06 | 0.06 | 2.6E-01 | Red |
| WP_011601855.1 | FRAAL0606 | chemical-damaging agent resistance protein C | 20665 | 4.50 | 12 | 215 | Frankia-10 | F201373.dat | 905 | 88 | 12 | 12 | 22 | 20 | 21 | 21 | 20 | 22 | 0.180% | 21 | 20 | 24 | 24 | 22 | 0.181% | 1.06 | 0.06 | 7.6E-02 | Red |
| WP_041938989.1 | FRAAL1776 | GDP-mannose 4.6-dehydratase | 36540 | 5.33 | 13 | 122 | Frankia-7 | F201370.dat | 577 | 37 | 10 | 10 | 13 | 13 | 11 | 10 | 14 | 11 | 0.058% | 13 | 13 | 14 | 11 | 12 | 0.058% | 1.06 | 0.06 | 2.0E-01 | Red |
| WP_011602364.1 | FRAAL1161 | phosphoribosylglycinamide formyltransferase | 23675 | 6.53 | 6 | 56 | Frankia-7 | F201370.dat | 204 | 25 | 5 | 5 | 7 | 4 | 6 | 6 | 6 | 5 | 0.041% | 6 | 7 | 4 | 5 | 7 | 0.041% | 1.06 | 0.06 | 2.9E-01 | Red |
| WP_011602318.1 | FRAAL1111 | 50S ribosomal protein L17 | 21824 | 7.00 | 12 | 188 | Frankia-5 | F201368.dat | 567 | 50 | 12 | 12 | 23 | 14 | 18 | 17 | 19 | 23 | 0.149% | 17 | 15 | 19 | 22 | 24 | 0.150% | 1.06 | 0.06 | 3.0E-01 | Red |
| WP_011606440.1 | FRAAL5355 | thiosulfate sulfurtransferase | 28707 | 4.79 | 4 | 23 | Frankia-7 | F201370.dat | 154 | 19 | 3 | 3 | 3 | 2 | 2 | 2 | 2 | 3 | 0.014% | 2 | 3 | 2 | 3 | 2 | 0.014% | 1.06 | 0.06 | 3.4E-01 | Red |
| WP_011606227.1 | FRAAL5119 | chemotaxis protein CheY | 14751 | 4.90 | 3 | 23 | Frankia-8 | F201371.dat | 139 | 20 | 3 | 3 | 3 | 3 | 2 | 2 | 3 | 1 | 0.027% | 2 | 2 | 3 | 3 | 2 | 0.027% | 1.06 | 0.06 | 3.5E-01 | Red |
| WP_041938604.1 | FRAAL0072 | F420-dependent oxidoreductase | 32969 | 5.55 | 13 | 89 | Frankia-8 | F201371.dat | 545 | 52 | 11 | 10 | 14 | 9 | 8 | 10 | 7 | 9 | 0.047% | 5 | 10 | 14 | 8 | 9 | 0.047% | 1.06 | 0.06 | 3.5E-01 | Red |
| WP_041939086.1 | FRAAL2243 | Zn-dependent hydrolase | 22863 | 5.45 | 5 | 23 | Frankia-10 | F201373.dat | 201 | 36 | 5 | 5 | 5 | 3 | 3 | 1 | 2 | 2 | 0.017% | 3 | 1 | 2 | 1 | 5 | 0.018% | 1.06 | 0.06 | 4.1E-01 | Red |
| WP_011606949.1 | FRAAL5888 | hypothetical protein | 45168 | 5.02 | 5 | 23 | Frankia-5 | F201368.dat | 202 | 16 | 5 | 5 | 5 | 2 | 4 | 0 | 0 | 5 | 0.009% | 0 | 2 | 2 | 4 | 4 | 0.009% | 1.06 | 0.06 | 4.4E-01 | Red |
| WP_041938940.1 | FRAAL1556 | ABC transporter substrate-binding protein | 35145 | 5.30 | 18 | 256 | Frankia-2 | F201365.dat | 834 | 61 | 17 | 17 | 28 | 22 | 28 | 26 | 24 | 24 | 0.126% | 27 | 25 | 25 | 27 | 28 | 0.126% | 1.06 | 0.06 | 1.1E-01 | Red |
| WP_011606075.1 | FRAAL4961 | tellurium resistance protein | 16333 | 5.18 | 11 | 124 | Frankia-2 | F201365.dat | 553 | 76 | 10 | 10 | 14 | 12 | 14 | 11 | 12 | 11 | 0.131% | 11 | 12 | 15 | 12 | 14 | 0.132% | 1.06 | 0.06 | 2.0E-01 | Red |
| WP_011605664.1 | FRAAL4546 | hypothetical protein | 35322 | 5.95 | 10 | 58 | Frankia-5 | F201368.dat | 375 | 32 | 7 | 7 | 8 | 6 | 6 | 4 | 4 | 8 | 0.028% | 4 | 4 | 8 | 8 | 6 | 0.029% | 1.06 | 0.06 | 3.7E-01 | Red |
| WP_011602186.1 | FRAAL0957 | hypothetical protein | 47780 | 9.22 | 31 | 229 | Frankia-5 | F201368.dat | 1042 | 52 | 23 | 23 | 29 | 19 | 20 | 19 | 24 | 29 | 0.083% | 15 | 24 | 27 | 26 | 26 | 0.083% | 1.06 | 0.06 | 3.2E-01 | Red |
| WP_041938679.1 | FRAAL0399 | methyltransferase | 31253 | 5.31 | 10 | 93 | Frankia-6 | F201369.dat | 508 | 59 | 10 | 8 | 9 | 10 | 7 | 8 | 9 | 11 | 0.051% | 9 | 10 | 10 | 11 | 8 | 0.052% | 1.06 | 0.06 | 2.6E-01 | Red |
| WP_011603282.1 | FRAAL2117 | malto-oligosyltrehalose synthase | 89507 | 4.99 | 28 | 268 | Frankia-8 | F201371.dat | 1192 | 36 | 23 | 23 | 32 | 29 | 26 | 28 | 24 | 23 | 0.052% | 24 | 30 | 32 | 26 | 26 | 0.052% | 1.06 | 0.06 | 2.1E-01 | Red |
| WP_011602418.1 | FRAAL1216 | hypothetical protein | 40112 | 5.32 | 8 | 95 | Frankia-9 | F201372.dat | 327 | 32 | 8 | 8 | 10 | 8 | 8 | 11 | 11 | 8 | 0.041% | 9 | 8 | 11 | 10 | 11 | 0.041% | 1.06 | 0.06 | 2.7E-01 | Red |
| WP_011602425.1 | FRAAL1224 | hypothetical protein | 31350 | 5.11 | 7 | 60 | Frankia-5 | F201368.dat | 264 | 29 | 5 | 5 | 5 | 7 | 5 | 6 | 6 | 5 | 0.033% | 6 | 5 | 9 | 4 | 7 | 0.033% | 1.06 | 0.06 | 3.4E-01 | Red |
| WP_011607441.1 | FRAAL6396 | molybdopterin biosynthesis protein MoeA | 45191 | 6.09 | 6 | 25 | Frankia-7 | F201370.dat | 173 | 9 | 3 | 3 | 4 | 2 | 2 | 2 | 3 | 3 | 0.009% | 2 | 4 | 3 | 2 | 2 | 0.010% | 1.06 | 0.06 | 3.6E-01 | Red |
| WP_050996999.1 | FRAAL0794 | ABC transporter | 56574 | 5.44 | 5 | 25 | Frankia-8 | F201371.dat | 157 | 8 | 4 | 4 | 4 | 2 | 3 | 3 | 2 | 2 | 0.008% | 2 | 3 | 4 | 2 | 2 | 0.008% | 1.06 | 0.06 | 3.6E-01 | Red |
| WP_011601408.1 | FRAAL0147 | hypothetical protein | 29274 | 4.89 | 4 | 25 | Frankia-4 | F201367.dat | 159 | 14 | 3 | 3 | 3 | 2 | 3 | 2 | 3 | 2 | 0.015% | 3 | 3 | 1 | 3 | 3 | 0.015% | 1.06 | 0.06 | 3.6E-01 | Red |
| WP_011605538.1 | FRAAL4415 | serine/threonine protein kinase | 66994 | 5.36 | 14 | 95 | Frankia-8 | F201371.dat | 516 | 24 | 12 | 11 | 16 | 10 | 8 | 12 | 8 | 8 | 0.025% | 6 | 9 | 16 | 7 | 11 | 0.025% | 1.06 | 0.06 | 3.8E-01 | Red |
| WP_050997293.1 | FRAAL6675 | hydrolase | 33324 | 9.82 | 5 | 25 | Frankia-9 | F201372.dat | 227 | 15 | 4 | 4 | 4 | 1 | 3 | 2 | 4 | 2 | 0.013% | 3 | 2 | 2 | 4 | 2 | 0.013% | 1.06 | 0.06 | 3.8E-01 | Red |
| WP_011602619.1 | FRAAL1424 | membrane protein | 80621 | 6.11 | 18 | 276 | Frankia-3 | F201366.dat | 1094 | 36 | 18 | 18 | 36 | 30 | 24 | 36 | 24 | 20 | 0.059% | 33 | 33 | 29 | 27 | 20 | 0.059% | 1.06 | 0.06 | 3.4E-01 | Red |
| WP_041939893.1 | FRAAL6429 | hypothetical protein | 39403 | 6.09 | 9 | 62 | Frankia-4 | F201367.dat | 307 | 29 | 7 | 7 | 7 | 5 | 6 | 6 | 7 | 6 | 0.027% | 6 | 6 | 8 | 5 | 7 | 0.027% | 1.06 | 0.06 | 2.6E-01 | Red |
| WP_011602473.1 | FRAAL1273 | mannose-6-phosphate isomerase | 39356 | 5.17 | 8 | 62 | Frankia-1 | F201364.dat | 334 | 20 | 7 | 7 | 7 | 7 | 6 | 6 | 7 | 4 | 0.027% | 6 | 8 | 8 | 4 | 6 | 0.027% | 1.06 | 0.06 | 3.4E-01 | Red |
| WP_011606824.1 | FRAAL5750 | heat-shock protein Hsp70 | 62758 | 5.27 | 5 | 27 | Frankia-7 | F201370.dat | 250 | 8 | 3 | 3 | 3 | 2 | 2 | 2 | 3 | 4 | 0.007% | 2 | 3 | 3 | 2 | 4 | 0.008% | 1.06 | 0.05 | 3.6E-01 | Red |
| WP_011603182.1 | FRAAL2010 | endoribonuclease YbeY | 19462 | 4.51 | 4 | 27 | Frankia-8 | F201371.dat | 300 | 29 | 4 | 4 | 4 | 2 | 2 | 4 | 3 | 2 | 0.024% | 3 | 2 | 4 | 3 | 2 | 0.024% | 1.06 | 0.05 | 3.6E-01 | Red |
| WP_011605269.1 | FRAAL4141 | aminoglycoside phosphotransferase | 50440 | 5.62 | 4 | 27 | Frankia-7 | F201370.dat | 209 | 14 | 4 | 4 | 4 | 3 | 3 | 2 | 3 | 2 | 0.009% | 3 | 4 | 3 | 3 | 1 | 0.009% | 1.06 | 0.05 | 3.7E-01 | Red |
| WP_011604554.1 | FRAAL3408 | alpha/beta hydrolase | 33218 | 5.30 | 5 | 27 | Frankia-8 | F201371.dat | 235 | 22 | 5 | 5 | 5 | 2 | 1 | 3 | 4 | 3 | 0.014% | 1 | 4 | 5 | 2 | 2 | 0.014% | 1.06 | 0.05 | 4.1E-01 | Red |
| WP_011601558.1 | FRAAL0300 | s-adenosylmethionine transferase | 29780 | 5.04 | 19 | 251 | Frankia-6 | F201369.dat | 934 | 58 | 15 | 15 | 24 | 23 | 25 | 27 | 26 | 21 | 0.146% | 24 | 30 | 26 | 25 | 24 | 0.146% | 1.06 | 0.05 | 2.0E-01 | Red |
| WP_041939627.1 | FRAAL5064 | chemical-damaging agent resistance protein C | 20431 | 4.64 | 13 | 177 | Frankia-5 | F201368.dat | 723 | 69 | 13 | 12 | 19 | 16 | 15 | 17 | 19 | 19 | 0.150% | 18 | 17 | 19 | 18 | 19 | 0.150% | 1.05 | 0.05 | 1.5E-01 | Red |
| WP_011602292.1 | FRAAL1068 | 50S ribosomal protein L10 | 19936 | 5.06 | 16 | 216 | Frankia-8 | F201371.dat | 600 | 61 | 13 | 13 | 24 | 19 | 24 | 19 | 22 | 21 | 0.188% | 18 | 24 | 24 | 23 | 22 | 0.188% | 1.05 | 0.05 | 2.2E-01 | Red |
| WP_041939260.1 | FRAAL2956 | aldo/keto reductase | 34565 | 4.88 | 18 | 179 | Frankia-7 | F201370.dat | 655 | 50 | 12 | 10 | 21 | 20 | 15 | 16 | 18 | 18 | 0.090% | 15 | 21 | 19 | 17 | 20 | 0.090% | 1.05 | 0.05 | 2.5E-01 | Red |
| WP_011603086.1 | FRAAL1911 | ATP-dependent Clp protease proteolytic subunit | 25346 | 5.53 | 17 | 255 | Frankia-8 | F201371.dat | 693 | 40 | 16 | 13 | 34 | 21 | 21 | 26 | 30 | 26 | 0.175% | 21 | 27 | 34 | 22 | 27 | 0.174% | 1.05 | 0.05 | 3.2E-01 | Red |
| WP_041940857.1 | FRAAL4977 | phosphoribosyl isomerase | 24956 | 4.40 | 13 | 105 | Frankia-8 | F201371.dat | 617 | 48 | 10 | 10 | 12 | 11 | 12 | 9 | 9 | 10 | 0.073% | 10 | 10 | 12 | 10 | 12 | 0.073% | 1.05 | 0.05 | 2.3E-01 | Red |
| WP_011604989.1 | FRAAL3854 | zinc-binding dehydrogenase | 37249 | 5.14 | 13 | 105 | Frankia-7 | F201370.dat | 506 | 40 | 11 | 11 | 12 | 9 | 9 | 11 | 13 | 9 | 0.049% | 10 | 12 | 9 | 11 | 12 | 0.049% | 1.05 | 0.05 | 2.8E-01 | Red |
| WP_041939959.1 | FRAAL6703 | helicase | 63809 | 5.51 | 24 | 185 | Frankia-8 | F201371.dat | 994 | 40 | 20 | 20 | 25 | 19 | 16 | 22 | 18 | 15 | 0.050% | 18 | 18 | 25 | 18 | 16 | 0.050% | 1.05 | 0.05 | 3.1E-01 | Red |
| WP_041939088.1 | FRAAL2247 | chromosome partitioning ATPase | 35289 | 4.88 | 8 | 68 | Frankia-7 | F201370.dat | 308 | 29 | 7 | 7 | 9 | 7 | 5 | 6 | 8 | 7 | 0.033% | 5 | 9 | 4 | 9 | 8 | 0.033% | 1.05 | 0.05 | 3.7E-01 | Red |
| WP_041939062.1 | FRAAL2147 | GTP pyrophosphokinase | 93371 | 7.79 | 5 | 29 | Frankia-10 | F201373.dat | 138 | 6 | 4 | 4 | 4 | 0 | 4 | 3 | 3 | 4 | 0.005% | 2 | 2 | 3 | 4 | 4 | 0.005% | 1.05 | 0.05 | 4.1E-01 | Red |
| WP_011603088.1 | FRAAL1913 | bifunctional folylpolyglutamate synthase/dihydrofolate synthase | 45145 | 7.11 | 9 | 29 | Frankia-4 | F201367.dat | 138 | 15 | 4 | 4 | 4 | 4 | 4 | 0 | 4 | 2 | 0.011% | 3 | 3 | 3 | 3 | 3 | 0.011% | 1.05 | 0.05 | 4.1E-01 | Red |
| WP_041940105.1 | FRAAL0640 | glycoside hydrolase | 33713 | 6.64 | 14 | 226 | Frankia-7 | F201370.dat | 875 | 59 | 12 | 12 | 27 | 18 | 23 | 21 | 22 | 26 | 0.116% | 26 | 27 | 20 | 25 | 18 | 0.116% | 1.05 | 0.05 | 3.0E-01 | Red |
| WP_011607111.1 | FRAAL6059 | DEAD/DEAH box helicase | 56606 | 6.30 | 18 | 111 | Frankia-5 | F201368.dat | 533 | 26 | 12 | 12 | 13 | 12 | 11 | 10 | 8 | 13 | 0.034% | 10 | 11 | 13 | 10 | 13 | 0.034% | 1.05 | 0.05 | 3.0E-01 | Red |
| WP_041939650.1 | FRAAL5153 | epimerase | 31330 | 10.45 | 5 | 31 | Frankia-7 | F201370.dat | 218 | 17 | 4 | 4 | 4 | 3 | 2 | 4 | 3 | 3 | 0.017% | 3 | 4 | 3 | 2 | 4 | 0.017% | 1.05 | 0.05 | 3.5E-01 | Red |
| WP_041938930.1 | FRAAL1503 | ribonuclease PH | 25984 | 5.33 | 4 | 31 | Frankia-8 | F201371.dat | 271 | 27 | 4 | 4 | 5 | 3 | 2 | 3 | 3 | 4 | 0.021% | 2 | 3 | 5 | 3 | 3 | 0.021% | 1.05 | 0.05 | 3.7E-01 | Red |
| WP_041939808.1 | FRAAL6028 | hypothetical protein | 18044 | 4.15 | 5 | 31 | Frankia-9 | F201372.dat | 215 | 46 | 4 | 4 | 4 | 4 | 3 | 1 | 3 | 4 | 0.030% | 2 | 4 | 2 | 4 | 4 | 0.030% | 1.05 | 0.05 | 4.0E-01 | Red |
| WP_011602581.1 | FRAAL1385 | hypothetical protein | 10153 | 5.04 | 6 | 31 | Frankia-4 | F201367.dat | 192 | 40 | 4 | 4 | 4 | 4 | 2 | 3 | 4 | 2 | 0.053% | 3 | 1 | 3 | 4 | 5 | 0.053% | 1.05 | 0.05 | 4.0E-01 | Red |
| WP_011601369.1 | FRAAL0103 | hypothetical protein | 26597 | 9.23 | 4 | 31 | Frankia-4 | F201367.dat | 219 | 29 | 4 | 4 | 6 | 2 | 2 | 3 | 6 | 2 | 0.020% | 2 | 3 | 4 | 3 | 4 | 0.020% | 1.05 | 0.05 | 4.1E-01 | Red |
| WP_011604983.1 | FRAAL3848 | thiolase | 41941 | 5.45 | 9 | 31 | Frankia-5 | F201368.dat | 298 | 18 | 5 | 5 | 5 | 3 | 2 | 2 | 3 | 5 | 0.013% | 1 | 4 | 6 | 2 | 3 | 0.013% | 1.05 | 0.05 | 4.2E-01 | Red |
| WP_011605527.1 | FRAAL4403 | helicase | 45656 | 10.17 | 12 | 74 | Frankia-8 | F201371.dat | 317 | 26 | 8 | 8 | 10 | 8 | 8 | 6 | 6 | 8 | 0.028% | 7 | 7 | 10 | 6 | 8 | 0.028% | 1.05 | 0.05 | 3.2E-01 | Red |
| WP_011606945.1 | FRAAL5884 | alpha-glucan phosphorylase | 93114 | 5.52 | 18 | 74 | Frankia-10 | F201373.dat | 535 | 18 | 11 | 11 | 11 | 7 | 10 | 4 | 6 | 9 | 0.014% | 5 | 7 | 7 | 8 | 11 | 0.014% | 1.05 | 0.05 | 3.9E-01 | Red |
| WP_041940155.1 | FRAAL0954 | aldo/keto reductase | 34973 | 5.29 | 21 | 330 | Frankia-10 | F201373.dat | 908 | 64 | 16 | 16 | 38 | 29 | 32 | 33 | 34 | 33 | 0.164% | 31 | 34 | 32 | 34 | 38 | 0.163% | 1.05 | 0.05 | 1.6E-01 | Red |
| WP_011606524.1 | FRAAL5443 | XRE family transcriptional regulator | 35477 | 6.19 | 6 | 33 | Frankia-4 | F201367.dat | 225 | 21 | 4 | 4 | 5 | 2 | 3 | 3 | 5 | 3 | 0.016% | 4 | 4 | 4 | 4 | 1 | 0.016% | 1.05 | 0.05 | 4.0E-01 | Red |
| WP_041941025.1 | FRAAL5992 | RNA polymerase sigma factor | 27680 | 5.57 | 8 | 33 | Frankia-8 | F201371.dat | 321 | 37 | 7 | 7 | 7 | 1 | 3 | 2 | 5 | 5 | 0.021% | 3 | 3 | 7 | 2 | 2 | 0.021% | 1.05 | 0.05 | 4.4E-01 | Red |
| WP_011602317.1 | FRAAL1109 | 30S ribosomal protein S4 | 23864 | 9.72 | 27 | 465 | Frankia-5 | F201368.dat | 1285 | 81 | 25 | 25 | 50 | 42 | 46 | 45 | 44 | 50 | 0.339% | 40 | 42 | 52 | 49 | 55 | 0.336% | 1.05 | 0.05 | 2.5E-01 | Red |
| WP_011603324.1 | FRAAL2159 | ATPase | 39545 | 5.64 | 21 | 209 | Frankia-4 | F201367.dat | 1027 | 68 | 19 | 19 | 23 | 22 | 20 | 19 | 23 | 18 | 0.092% | 16 | 24 | 24 | 22 | 21 | 0.091% | 1.05 | 0.05 | 2.9E-01 | Red |
| WP_041938968.1 | FRAAL1683 | alcohol dehydrogenase | 32476 | 5.27 | 16 | 213 | Frankia-5 | F201368.dat | 775 | 52 | 15 | 15 | 23 | 19 | 21 | 19 | 22 | 23 | 0.114% | 21 | 20 | 24 | 22 | 22 | 0.113% | 1.05 | 0.04 | 1.8E-01 | Red |
| WP_011605637.1 | FRAAL4518 | 3-oxoacyl-ACP reductase | 24160 | 5.93 | 12 | 125 | Frankia-7 | F201370.dat | 589 | 45 | 10 | 10 | 14 | 13 | 11 | 12 | 12 | 13 | 0.090% | 13 | 14 | 13 | 11 | 13 | 0.089% | 1.05 | 0.04 | 1.8E-01 | Red |
| WP_041939270.1 | FRAAL3042 | short-chain dehydrogenase | 28928 | 5.19 | 7 | 35 | Frankia-10 | F201373.dat | 172 | 25 | 5 | 5 | 5 | 2 | 4 | 4 | 3 | 4 | 0.021% | 3 | 3 | 3 | 4 | 5 | 0.021% | 1.05 | 0.04 | 3.7E-01 | Red |
| WP_011601901.1 | FRAAL0653 | chemotaxis protein CheY | 16578 | 4.76 | 3 | 35 | Frankia-4 | F201367.dat | 166 | 25 | 3 | 3 | 4 | 2 | 4 | 3 | 4 | 4 | 0.037% | 4 | 3 | 4 | 2 | 5 | 0.037% | 1.05 | 0.04 | 3.8E-01 | Red |
| WP_035921112.1 | FRAAL1133 | molecular chaperone GroES | 10940 | 4.95 | 15 | 262 | Frankia-10 | F201373.dat | 829 | 93 | 14 | 14 | 30 | 26 | 26 | 21 | 29 | 26 | 0.418% | 26 | 23 | 27 | 28 | 30 | 0.412% | 1.05 | 0.04 | 2.5E-01 | Red |
| WP_011606078.1 | FRAAL4964 | dihydropyrimidine dehydrogenase subunit A | 51836 | 5.67 | 25 | 262 | Frankia-8 | F201371.dat | 1132 | 56 | 21 | 21 | 34 | 29 | 23 | 27 | 24 | 25 | 0.088% | 23 | 25 | 34 | 22 | 30 | 0.087% | 1.05 | 0.04 | 3.2E-01 | Red |
| WP_011606601.1 | FRAAL5517 | hypothetical protein | 19815 | 4.65 | 8 | 127 | Frankia-2 | F201365.dat | 474 | 60 | 8 | 8 | 12 | 13 | 12 | 13 | 13 | 11 | 0.112% | 13 | 13 | 13 | 14 | 12 | 0.110% | 1.04 | 0.04 | 1.4E-01 | Red |
| WP_011605730.1 | FRAAL4613 | DNA polymerase I | 95347 | 5.08 | 14 | 82 | Frankia-1 | F201364.dat | 494 | 14 | 11 | 11 | 13 | 13 | 6 | 6 | 9 | 6 | 0.015% | 9 | 6 | 9 | 10 | 8 | 0.015% | 1.04 | 0.04 | 4.0E-01 | Red |
| WP_011605917.1 | FRAAL4802 | F420-dependent oxidoreductase | 33310 | 5.09 | 5 | 37 | Frankia-8 | F201371.dat | 265 | 28 | 5 | 5 | 5 | 3 | 3 | 4 | 3 | 5 | 0.019% | 4 | 3 | 5 | 3 | 4 | 0.019% | 1.04 | 0.04 | 3.6E-01 | Red |
| WP_011606781.1 | FRAAL5707 | diaminopimelate epimerase | 37001 | 4.70 | 6 | 37 | Frankia-8 | F201371.dat | 274 | 19 | 5 | 5 | 5 | 3 | 4 | 4 | 5 | 2 | 0.017% | 4 | 3 | 5 | 3 | 4 | 0.017% | 1.04 | 0.04 | 3.8E-01 | Red |
| WP_011601902.1 | FRAAL0654 | hypothetical protein | 24157 | 5.82 | 7 | 37 | Frankia-3 | F201366.dat | 161 | 26 | 4 | 4 | 5 | 3 | 4 | 5 | 4 | 2 | 0.027% | 4 | 5 | 3 | 5 | 2 | 0.026% | 1.04 | 0.04 | 4.0E-01 | Red |
| WP_011605103.1 | FRAAL3971 | short-chain dehydrogenase | 35750 | 5.58 | 6 | 39 | Frankia-5 | F201368.dat | 172 | 17 | 5 | 5 | 6 | 3 | 3 | 4 | 3 | 6 | 0.019% | 3 | 3 | 5 | 5 | 4 | 0.019% | 1.04 | 0.04 | 4.0E-01 | Red |
| WP_011607561.1 | FRAAL6520 | hypothetical protein | 56032 | 4.85 | 36 | 384 | Frankia-8 | F201371.dat | 1109 | 56 | 28 | 28 | 59 | 41 | 29 | 42 | 42 | 34 | 0.120% | 30 | 32 | 59 | 33 | 42 | 0.118% | 1.04 | 0.04 | 4.0E-01 | Red |
| WP_041940003.1 | FRAAL6877 | chromosome partitioning protein | 35360 | 6.26 | 11 | 90 | Frankia-7 | F201370.dat | 525 | 35 | 9 | 9 | 11 | 9 | 8 | 8 | 10 | 9 | 0.044% | 7 | 11 | 10 | 8 | 10 | 0.044% | 1.04 | 0.04 | 3.2E-01 | Red |
| WP_011606928.1 | FRAAL5860 | hypothetical protein | 36262 | 5.33 | 12 | 90 | Frankia-5 | F201368.dat | 573 | 47 | 9 | 9 | 11 | 7 | 10 | 6 | 10 | 11 | 0.043% | 9 | 10 | 8 | 8 | 11 | 0.043% | 1.04 | 0.04 | 3.7E-01 | Red |
| WP_011602768.1 | FRAAL1581 | hypothetical protein | 92656 | 9.01 | 33 | 243 | Frankia-9 | F201372.dat | 1182 | 38 | 25 | 24 | 28 | 31 | 24 | 28 | 21 | 15 | 0.046% | 29 | 15 | 31 | 28 | 21 | 0.045% | 1.04 | 0.04 | 4.1E-01 | Red |
| WP_041939708.1 | FRAAL5458 | S-adenosyl methyltransferase | 29454 | 4.74 | 13 | 194 | Frankia-8 | F201371.dat | 510 | 37 | 10 | 10 | 21 | 20 | 17 | 20 | 19 | 19 | 0.115% | 18 | 21 | 21 | 20 | 19 | 0.113% | 1.04 | 0.04 | 1.7E-01 | Red |
| WP_041939413.1 | FRAAL3918 | hypothetical protein | 17226 | 11.24 | 3 | 41 | Frankia-8 | F201371.dat | 100 | 19 | 3 | 3 | 5 | 4 | 4 | 4 | 4 | 4 | 0.041% | 4 | 4 | 5 | 4 | 4 | 0.041% | 1.04 | 0.04 | 3.4E-01 | Red |
| WP_011607312.1 | FRAAL6262 | glycosyl transferase | 33058 | 9.13 | 12 | 94 | Frankia-5 | F201368.dat | 282 | 38 | 9 | 9 | 12 | 9 | 9 | 7 | 9 | 12 | 0.050% | 9 | 10 | 9 | 10 | 10 | 0.049% | 1.04 | 0.04 | 3.3E-01 | Red |
| WP_041939576.1 | FRAAL4824 | hypothetical protein | 29880 | 5.21 | 13 | 153 | Frankia-8 | F201371.dat | 661 | 47 | 11 | 11 | 20 | 12 | 14 | 17 | 17 | 15 | 0.090% | 12 | 14 | 20 | 14 | 18 | 0.088% | 1.04 | 0.04 | 3.7E-01 | Red |
| WP_011602332.1 | FRAAL1125 | serine hydroxymethyltransferase | 44110 | 6.10 | 27 | 210 | Frankia-1 | F201364.dat | 948 | 57 | 19 | 19 | 25 | 25 | 18 | 19 | 21 | 20 | 0.083% | 23 | 23 | 22 | 18 | 21 | 0.082% | 1.04 | 0.04 | 3.1E-01 | Red |
| WP_050997429.1 | FRAAL4936 | hypothetical protein | 20809 | 9.63 | 6 | 45 | Frankia-1 | F201364.dat | 270 | 50 | 6 | 6 | 6 | 6 | 4 | 4 | 3 | 5 | 0.038% | 3 | 6 | 2 | 7 | 5 | 0.037% | 1.04 | 0.04 | 4.3E-01 | Red |
| WP_011604961.1 | FRAAL3824 | branched-chain amino acid ABC transporter substrate-binding protein | 37791 | 8.97 | 17 | 161 | Frankia-5 | F201368.dat | 962 | 59 | 13 | 13 | 19 | 15 | 14 | 15 | 16 | 19 | 0.075% | 16 | 17 | 16 | 16 | 17 | 0.073% | 1.04 | 0.04 | 2.7E-01 | Red |
| WP_011601854.1 | FRAAL0605 | aspartokinase | 44023 | 4.98 | 20 | 161 | Frankia-9 | F201372.dat | 773 | 51 | 14 | 14 | 20 | 15 | 17 | 14 | 16 | 17 | 0.064% | 12 | 17 | 18 | 20 | 15 | 0.063% | 1.04 | 0.04 | 3.5E-01 | Red |
| WP_011601752.1 | FRAAL0499 | hypothetical protein | 18919 | 10.70 | 11 | 161 | Frankia-5 | F201368.dat | 489 | 49 | 10 | 10 | 21 | 14 | 14 | 14 | 16 | 21 | 0.149% | 14 | 16 | 19 | 15 | 18 | 0.146% | 1.04 | 0.04 | 3.6E-01 | Red |
| WP_041939785.1 | FRAAL5932 | ATP synthase F0F1 subunit gamma | 32294 | 9.06 | 19 | 334 | Frankia-5 | F201368.dat | 978 | 58 | 18 | 18 | 36 | 29 | 34 | 35 | 30 | 36 | 0.181% | 30 | 37 | 33 | 35 | 35 | 0.177% | 1.04 | 0.03 | 2.6E-01 | Red |
| WP_011606079.1 | FRAAL4965 | glutamate synthase | 164943 | 5.56 | 62 | 458 | Frankia-8 | F201371.dat | 2438 | 40 | 46 | 46 | 69 | 50 | 41 | 53 | 32 | 49 | 0.049% | 50 | 25 | 69 | 42 | 47 | 0.048% | 1.03 | 0.03 | 4.2E-01 | Red |
| WP_050997237.1 | FRAAL5502 | hypothetical protein | 31548 | 4.75 | 13 | 108 | Frankia-5 | F201368.dat | 565 | 57 | 12 | 12 | 12 | 9 | 11 | 10 | 11 | 12 | 0.060% | 10 | 13 | 11 | 11 | 10 | 0.059% | 1.03 | 0.03 | 3.0E-01 | Red |
| WP_011607866.1 | FRAAL6831 | maleylpyruvate isomerase | 28517 | 4.99 | 6 | 49 | Frankia-5 | F201368.dat | 216 | 27 | 6 | 6 | 7 | 5 | 4 | 4 | 4 | 7 | 0.030% | 4 | 4 | 7 | 5 | 5 | 0.030% | 1.03 | 0.03 | 4.0E-01 | Red |
| WP_041939428.1 | FRAAL3974 | 3-hydroxy-2-methylbutyryl-CoA dehydrogenase | 26461 | 5.17 | 9 | 49 | Frankia-8 | F201371.dat | 318 | 27 | 6 | 6 | 8 | 6 | 4 | 5 | 4 | 5 | 0.032% | 4 | 5 | 8 | 3 | 5 | 0.032% | 1.03 | 0.03 | 4.2E-01 | Red |
| WP_011607175.1 | FRAAL6124 | 2.3.4.5-tetrahydropyridine-2.6-carboxylate N-succinyltransferase | 29237 | 5.27 | 21 | 232 | Frankia-8 | F201371.dat | 873 | 53 | 16 | 16 | 28 | 20 | 22 | 22 | 25 | 25 | 0.139% | 19 | 23 | 28 | 22 | 26 | 0.136% | 1.03 | 0.03 | 3.4E-01 | Red |
| WP_011607741.1 | FRAAL6704 | hypothetical protein | 89374 | 5.47 | 9 | 53 | Frankia-3 | F201366.dat | 365 | 11 | 8 | 8 | 8 | 4 | 4 | 8 | 5 | 5 | 0.010% | 5 | 7 | 6 | 5 | 4 | 0.010% | 1.03 | 0.03 | 4.1E-01 | Red |
| WP_011604010.1 | FRAAL2864 | ABC transporter | 49134 | 5.02 | 10 | 53 | Frankia-10 | F201373.dat | 276 | 19 | 7 | 7 | 8 | 6 | 4 | 3 | 7 | 6 | 0.019% | 6 | 4 | 4 | 5 | 8 | 0.019% | 1.03 | 0.03 | 4.3E-01 | Red |
| WP_011605369.1 | FRAAL4243 | universal stress protein UspA | 30489 | 6.50 | 25 | 445 | Frankia-2 | F201365.dat | 1211 | 63 | 24 | 24 | 46 | 43 | 46 | 40 | 46 | 44 | 0.256% | 45 | 46 | 40 | 46 | 49 | 0.250% | 1.03 | 0.03 | 2.3E-01 | Red |
| WP_011603262.1 | FRAAL2096 | 3-hydroxyacyl-CoA dehydrogenase | 74819 | 5.18 | 18 | 120 | Frankia-8 | F201371.dat | 628 | 25 | 14 | 14 | 17 | 11 | 11 | 14 | 12 | 11 | 0.028% | 9 | 15 | 17 | 9 | 11 | 0.027% | 1.03 | 0.03 | 4.1E-01 | Red |
| WP_011602134.1 | FRAAL0903 | PII uridylyl-transferase | 85164 | 5.53 | 15 | 55 | Frankia-1 | F201364.dat | 397 | 15 | 8 | 8 | 8 | 8 | 5 | 4 | 5 | 5 | 0.011% | 4 | 2 | 9 | 6 | 7 | 0.011% | 1.03 | 0.03 | 4.4E-01 | Red |
| WP_011605811.1 | FRAAL4695 | oxidoreductase | 26627 | 6.31 | 14 | 252 | Frankia-9 | F201372.dat | 880 | 49 | 14 | 14 | 28 | 23 | 26 | 22 | 27 | 26 | 0.166% | 19 | 25 | 26 | 28 | 30 | 0.162% | 1.03 | 0.03 | 3.6E-01 | Red |
| WP_011606766.1 | FRAAL5693 | histidine kinase | 113164 | 5.05 | 22 | 126 | Frankia-8 | F201371.dat | 839 | 22 | 16 | 16 | 17 | 14 | 12 | 11 | 15 | 10 | 0.020% | 15 | 10 | 17 | 13 | 9 | 0.019% | 1.03 | 0.03 | 4.1E-01 | Red |
| WP_011606939.1 | FRAAL5878 | electron transfer flavoprotein subunit beta | 26847 | 5.36 | 24 | 471 | Frankia-10 | F201373.dat | 1430 | 93 | 21 | 21 | 53 | 46 | 43 | 43 | 49 | 51 | 0.308% | 38 | 43 | 64 | 41 | 53 | 0.300% | 1.03 | 0.03 | 3.9E-01 | Red |
| WP_050997136.1 | FRAAL3477 | short-chain dehydrogenase | 37203 | 10.21 | 11 | 59 | Frankia-5 | F201368.dat | 323 | 23 | 7 | 7 | 8 | 5 | 4 | 8 | 4 | 8 | 0.028% | 6 | 7 | 7 | 6 | 4 | 0.027% | 1.03 | 0.03 | 4.3E-01 | Red |
| WP_011603085.1 | FRAAL1910 | ATP-dependent Clp protease ClpP | 23093 | 4.92 | 9 | 130 | Frankia-5 | F201368.dat | 474 | 45 | 8 | 8 | 13 | 13 | 13 | 12 | 13 | 13 | 0.099% | 15 | 15 | 12 | 14 | 10 | 0.096% | 1.03 | 0.03 | 3.5E-01 | Red |
| WP_041940873.1 | FRAAL5081 | serine/threonine protein kinase | 76095 | 5.62 | 9 | 61 | Frankia-2 | F201365.dat | 221 | 12 | 6 | 6 | 6 | 6 | 6 | 7 | 5 | 6 | 0.014% | 6 | 7 | 7 | 5 | 6 | 0.014% | 1.03 | 0.03 | 3.5E-01 | Red |
| WP_011605379.1 | FRAAL4253 | nitrate ABC transporter substrate-binding protein | 42137 | 5.87 | 8 | 61 | Frankia-7 | F201370.dat | 465 | 32 | 7 | 7 | 8 | 7 | 7 | 5 | 7 | 4 | 0.025% | 6 | 8 | 7 | 5 | 5 | 0.025% | 1.03 | 0.03 | 4.1E-01 | Red |
| WP_009740954.1 | FRAAL0603 | cold-shock protein | 7280 | 5.08 | 7 | 278 | Frankia-4 | F201367.dat | 399 | 81 | 6 | 6 | 34 | 22 | 27 | 29 | 34 | 25 | 0.672% | 29 | 25 | 22 | 35 | 30 | 0.652% | 1.03 | 0.03 | 4.0E-01 | Red |
| WP_041940803.1 | FRAAL4739 | oxidoreductase | 27221 | 5.39 | 19 | 134 | Frankia-8 | F201371.dat | 739 | 63 | 15 | 15 | 16 | 11 | 12 | 16 | 12 | 15 | 0.087% | 11 | 17 | 16 | 12 | 12 | 0.084% | 1.03 | 0.03 | 4.0E-01 | Red |
| WP_041940230.1 | FRAAL1461 | bifunctional allantoicase/OHCU decarboxylase | 56205 | 5.12 | 27 | 211 | Frankia-1 | F201364.dat | 1020 | 52 | 19 | 19 | 23 | 23 | 20 | 19 | 20 | 22 | 0.066% | 21 | 20 | 24 | 22 | 20 | 0.064% | 1.03 | 0.03 | 2.9E-01 | Red |
| WP_041939604.1 | FRAAL4994 | restriction endonuclease subunit R | 122790 | 6.05 | 11 | 65 | Frankia-10 | F201373.dat | 352 | 7 | 8 | 8 | 8 | 8 | 7 | 5 | 6 | 6 | 0.009% | 6 | 5 | 8 | 6 | 8 | 0.009% | 1.03 | 0.03 | 4.0E-01 | Red |
| WP_050997175.1 | FRAAL4295 | NADH dehydrogenase | 53352 | 9.44 | 13 | 69 | Frankia-8 | F201371.dat | 377 | 22 | 9 | 9 | 10 | 7 | 7 | 5 | 7 | 8 | 0.023% | 7 | 3 | 10 | 8 | 7 | 0.022% | 1.03 | 0.03 | 4.4E-01 | Red |
| WP_011607118.1 | FRAAL6066 | aminotransferase | 43268 | 5.43 | 11 | 69 | Frankia-8 | F201371.dat | 410 | 26 | 9 | 9 | 11 | 6 | 5 | 9 | 5 | 9 | 0.028% | 6 | 4 | 11 | 7 | 7 | 0.027% | 1.03 | 0.03 | 4.5E-01 | Red |
| WP_050997049.1 | FRAAL1956 | NADPH:quinone oxidoreductase | 35852 | 5.20 | 9 | 71 | Frankia-7 | F201370.dat | 364 | 30 | 7 | 7 | 8 | 8 | 7 | 5 | 8 | 7 | 0.035% | 7 | 8 | 7 | 6 | 8 | 0.034% | 1.03 | 0.02 | 3.9E-01 | Red |
| WP_041940626.1 | FRAAL3757 | transcriptional regulator | 54163 | 5.55 | 10 | 71 | Frankia-7 | F201370.dat | 550 | 29 | 9 | 9 | 10 | 8 | 6 | 8 | 7 | 6 | 0.023% | 6 | 10 | 6 | 8 | 6 | 0.022% | 1.03 | 0.02 | 4.2E-01 | Red |
| WP_041940440.1 | FRAAL2702 | protein-L-isoaspartate O-methyltransferase | 44352 | 5.10 | 21 | 255 | Frankia-5 | F201368.dat | 995 | 66 | 18 | 18 | 27 | 26 | 23 | 23 | 27 | 27 | 0.101% | 24 | 25 | 26 | 24 | 30 | 0.098% | 1.02 | 0.02 | 3.4E-01 | Red |
| WP_011601711.1 | FRAAL0455 | CRISPR-associated protein Cse4 | 40751 | 6.03 | 19 | 168 | Frankia-8 | F201371.dat | 951 | 52 | 17 | 17 | 22 | 20 | 15 | 14 | 16 | 18 | 0.073% | 16 | 14 | 22 | 16 | 17 | 0.070% | 1.02 | 0.02 | 4.1E-01 | Red |
| WP_011607673.1 | FRAAL6636 | Fe-S oxidoreductase | 80270 | 5.41 | 34 | 350 | Frankia-6 | F201369.dat | 1483 | 47 | 28 | 28 | 42 | 39 | 36 | 40 | 31 | 27 | 0.077% | 42 | 30 | 41 | 33 | 31 | 0.074% | 1.02 | 0.02 | 4.1E-01 | Red |
| WP_041939799.1 | FRAAL6000 | ATPase | 53198 | 5.74 | 19 | 174 | Frankia-7 | F201370.dat | 737 | 34 | 17 | 17 | 22 | 18 | 20 | 17 | 16 | 15 | 0.058% | 17 | 22 | 16 | 17 | 16 | 0.056% | 1.02 | 0.02 | 3.9E-01 | Red |
| WP_041939087.1 | FRAAL2245 | alcohol dehydrogenase | 37892 | 5.10 | 17 | 176 | Frankia-8 | F201371.dat | 699 | 46 | 14 | 14 | 20 | 17 | 16 | 18 | 17 | 19 | 0.082% | 18 | 18 | 20 | 16 | 17 | 0.079% | 1.02 | 0.02 | 3.2E-01 | Red |
| WP_011607079.1 | FRAAL6027 | hypothetical protein | 110110 | 9.23 | 28 | 190 | Frankia-8 | F201371.dat | 1017 | 23 | 18 | 18 | 24 | 22 | 18 | 19 | 16 | 19 | 0.030% | 22 | 16 | 24 | 18 | 16 | 0.029% | 1.02 | 0.02 | 4.2E-01 | Red |
| WP_011607459.1 | FRAAL6414 | histidine kinase | 144198 | 4.82 | 35 | 194 | Frankia-1 | F201364.dat | 1433 | 25 | 26 | 26 | 27 | 27 | 17 | 23 | 14 | 15 | 0.024% | 20 | 14 | 26 | 20 | 18 | 0.023% | 1.02 | 0.02 | 4.5E-01 | Red |
| WP_011606994.1 | FRAAL5934 | ATP synthase subunit delta | 29589 | 9.61 | 21 | 515 | Frankia-8 | F201371.dat | 970 | 61 | 20 | 20 | 51 | 47 | 51 | 51 | 54 | 52 | 0.308% | 45 | 52 | 51 | 54 | 58 | 0.296% | 1.02 | 0.02 | 3.4E-01 | Red |
| WP_011601270.1 | FRAAL0002 | chromosomal replication initiator protein DnaA | 58456 | 6.01 | 14 | 99 | Frankia-3 | F201366.dat | 337 | 25 | 10 | 10 | 13 | 11 | 8 | 13 | 10 | 7 | 0.030% | 8 | 8 | 13 | 11 | 10 | 0.029% | 1.02 | 0.02 | 4.5E-01 | Red |
| WP_041939580.1 | FRAAL4852 | GntR family transcriptional regulator | 29330 | 5.69 | 16 | 101 | Frankia-10 | F201373.dat | 443 | 43 | 10 | 10 | 11 | 9 | 11 | 10 | 10 | 10 | 0.061% | 9 | 11 | 12 | 8 | 11 | 0.059% | 1.02 | 0.02 | 4.1E-01 | Red |
| WP_011607313.1 | FRAAL6263 | glycosyl transferase | 43090 | 8.68 | 13 | 101 | Frankia-7 | F201370.dat | 537 | 39 | 9 | 9 | 13 | 10 | 12 | 9 | 11 | 8 | 0.041% | 12 | 13 | 8 | 10 | 8 | 0.040% | 1.02 | 0.02 | 4.4E-01 | Red |
| WP_041940643.1 | FRAAL3850 | oxidoreductase | 32837 | 5.34 | 10 | 107 | Frankia-2 | F201365.dat | 548 | 39 | 8 | 8 | 12 | 8 | 12 | 8 | 13 | 12 | 0.058% | 9 | 12 | 10 | 11 | 12 | 0.055% | 1.02 | 0.02 | 4.4E-01 | Red |
| WP_011606773.1 | FRAAL5699 | vitamin B12-dependent ribonucleotide reductase | 107165 | 5.60 | 26 | 226 | Frankia-1 | F201364.dat | 1185 | 26 | 23 | 23 | 26 | 26 | 21 | 25 | 20 | 20 | 0.037% | 25 | 16 | 29 | 22 | 22 | 0.036% | 1.02 | 0.02 | 4.4E-01 | Red |
| WP_041939424.1 | FRAAL3959 | phosphoesterase | 60904 | 6.31 | 13 | 111 | Frankia-7 | F201370.dat | 517 | 26 | 11 | 11 | 14 | 11 | 10 | 11 | 12 | 11 | 0.032% | 13 | 14 | 7 | 14 | 8 | 0.031% | 1.02 | 0.02 | 4.5E-01 | Red |
| WP_011601374.1 | FRAAL0109 | citrate synthase | 40098 | 5.71 | 19 | 357 | Frankia-10 | F201373.dat | 882 | 54 | 18 | 18 | 40 | 33 | 38 | 33 | 34 | 39 | 0.158% | 33 | 37 | 36 | 34 | 40 | 0.151% | 1.02 | 0.02 | 3.7E-01 | Red |
| WP_041940986.1 | FRAAL5743 | FAD-dependent thymidylate synthase | 26705 | 6.20 | 11 | 117 | Frankia-4 | F201367.dat | 441 | 52 | 10 | 10 | 13 | 10 | 10 | 13 | 13 | 12 | 0.078% | 11 | 13 | 13 | 9 | 13 | 0.074% | 1.02 | 0.02 | 4.3E-01 | Red |
| WP_041938595.1 | FRAAL0033 | PEBP family protein | 18987 | 5.08 | 5 | 119 | Frankia-9 | F201372.dat | 328 | 41 | 5 | 5 | 11 | 14 | 11 | 10 | 12 | 12 | 0.111% | 12 | 12 | 13 | 11 | 12 | 0.106% | 1.02 | 0.02 | 4.0E-01 | Red |
| WP_041939644.1 | FRAAL5126 | ribokinase | 34935 | 4.76 | 16 | 252 | Frankia-8 | F201371.dat | 907 | 63 | 15 | 15 | 29 | 28 | 25 | 22 | 26 | 24 | 0.128% | 23 | 24 | 29 | 24 | 27 | 0.122% | 1.02 | 0.02 | 4.0E-01 | Red |
| WP_011602290.1 | FRAAL1066 | 50S ribosomal protein L1 | 25392 | 9.54 | 21 | 419 | Frankia-10 | F201373.dat | 1383 | 74 | 21 | 21 | 48 | 43 | 41 | 41 | 41 | 42 | 0.292% | 38 | 41 | 40 | 44 | 48 | 0.280% | 1.01 | 0.01 | 3.7E-01 | Red |
| WP_011604697.1 | FRAAL3556 | oxidoreductase | 38041 | 5.26 | 11 | 133 | Frankia-8 | F201371.dat | 735 | 45 | 10 | 10 | 19 | 14 | 13 | 9 | 14 | 16 | 0.062% | 9 | 15 | 19 | 12 | 12 | 0.059% | 1.01 | 0.01 | 4.6E-01 | Red |
| WP_041938587.1 | FRAAL0004 | DNA polymerase III subunit beta | 40963 | 4.73 | 18 | 153 | Frankia-8 | F201371.dat | 749 | 54 | 13 | 12 | 20 | 18 | 13 | 14 | 16 | 15 | 0.066% | 16 | 15 | 20 | 11 | 15 | 0.063% | 1.01 | 0.01 | 4.5E-01 | Red |
| WP_041939642.1 | FRAAL5123 | cytochrome C oxidase subunit II | 35251 | 9.47 | 15 | 324 | Frankia-5 | F201368.dat | 584 | 48 | 14 | 14 | 35 | 29 | 33 | 32 | 32 | 35 | 0.163% | 29 | 36 | 33 | 33 | 32 | 0.156% | 1.01 | 0.01 | 4.0E-01 | Red |
| WP_011607801.1 | FRAAL6765 | peptidyl-prolyl cis-trans isomerase | 19003 | 6.06 | 16 | 344 | Frankia-4 | F201367.dat | 1018 | 70 | 15 | 15 | 35 | 33 | 35 | 33 | 35 | 35 | 0.321% | 36 | 32 | 30 | 36 | 39 | 0.307% | 1.01 | 0.01 | 4.1E-01 | Red |
| WP_011607309.1 | FRAAL6259 | methoxymalonyl-ACP biosynthesis protein FkbH | 75962 | 5.37 | 24 | 169 | Frankia-8 | F201371.dat | 731 | 32 | 18 | 18 | 22 | 18 | 16 | 14 | 20 | 16 | 0.039% | 14 | 15 | 22 | 17 | 17 | 0.038% | 1.01 | 0.01 | 4.6E-01 | Red |
| WP_041939529.1 | FRAAL4528 | hydroxyglutarate oxidase | 45687 | 9.29 | 27 | 396 | Frankia-4 | F201367.dat | 1156 | 73 | 26 | 26 | 44 | 34 | 38 | 38 | 44 | 43 | 0.154% | 32 | 40 | 46 | 37 | 44 | 0.147% | 1.01 | 0.01 | 4.5E-01 | Red |
| WP_011607887.1 | FRAAL6852 | single-stranded DNA-binding protein | 18915 | 5.51 | 9 | 239 | Frankia-5 | F201368.dat | 620 | 49 | 8 | 8 | 28 | 22 | 24 | 22 | 23 | 28 | 0.225% | 24 | 23 | 23 | 24 | 26 | 0.214% | 1.01 | 0.01 | 4.4E-01 | Red |
| WP_011603301.1 | FRAAL2136 | pyridoxal biosynthesis lyase | 32220 | 5.47 | 14 | 251 | Frankia-5 | F201368.dat | 872 | 47 | 13 | 13 | 25 | 26 | 26 | 23 | 25 | 25 | 0.138% | 21 | 26 | 28 | 26 | 25 | 0.132% | 1.01 | 0.01 | 4.4E-01 | Red |
| WP_041939549.1 | FRAAL4660 | hypothetical protein | 50721 | 6.29 | 30 | 264 | Frankia-8 | F201371.dat | 1199 | 56 | 24 | 24 | 34 | 28 | 25 | 25 | 24 | 30 | 0.093% | 22 | 24 | 34 | 24 | 28 | 0.088% | 1.00 | 0.00 | 5.0E-01 | Red |
| WP_011607284.1 | FRAAL6234 | signal peptidase | 78098 | 7.13 | 28 | 246 | Frankia-7 | F201370.dat | 860 | 33 | 20 | 20 | 26 | 25 | 25 | 25 | 24 | 24 | 0.056% | 20 | 26 | 26 | 26 | 25 | 0.053% | 1.00 | 0.00 | 5.0E-01 | Red |
| WP_011606237.1 | FRAAL5130 | aldo/keto reductase | 34882 | 5.25 | 27 | 318 | Frankia-7 | F201370.dat | 1254 | 75 | 23 | 23 | 35 | 32 | 33 | 33 | 30 | 31 | 0.163% | 31 | 35 | 35 | 31 | 27 | 0.154% | 1.00 | 0.00 | 5.0E-01 | Red |
| WP_011602212.1 | FRAAL0985 | uroporphyrinogen III methyltransferase | 61954 | 6.01 | 24 | 322 | Frankia-8 | F201371.dat | 1341 | 49 | 22 | 22 | 34 | 37 | 34 | 32 | 32 | 26 | 0.093% | 31 | 30 | 34 | 32 | 34 | 0.088% | 1.00 | 0.00 | 5.0E-01 | Red |
| WP_041939058.1 | FRAAL2118 | glycogen debranching protein | 82541 | 5.01 | 22 | 212 | Frankia-8 | F201371.dat | 869 | 32 | 18 | 18 | 25 | 25 | 19 | 24 | 20 | 18 | 0.046% | 17 | 20 | 25 | 19 | 25 | 0.043% | 1.00 | 0.00 | 5.0E-01 | Red |
| WP_011605559.1 | FRAAL4438 | oxidoreductase | 36433 | 5.90 | 18 | 122 | Frankia-8 | F201371.dat | 669 | 43 | 14 | 14 | 19 | 11 | 9 | 12 | 11 | 18 | 0.060% | 8 | 12 | 19 | 9 | 13 | 0.056% | 1.00 | 0.00 | 5.0E-01 | Red |
| WP_011606265.1 | FRAAL5161 | glutamine synthetase | 53879 | 5.06 | 17 | 308 | Frankia-3 | F201366.dat | 896 | 39 | 17 | 17 | 35 | 29 | 29 | 35 | 31 | 30 | 0.102% | 37 | 28 | 25 | 33 | 31 | 0.096% | 1.00 | 0.00 | 5.0E-01 | Red |
| WP_011602262.1 | FRAAL1035 | NADH-quinone oxidoreductase subunit D | 51221 | 5.26 | 17 | 146 | Frankia-1 | F201364.dat | 528 | 41 | 15 | 15 | 19 | 19 | 13 | 18 | 12 | 11 | 0.051% | 12 | 13 | 19 | 14 | 15 | 0.048% | 1.00 | 0.00 | 5.0E-01 | Red |
| WP_041939860.1 | FRAAL6267 | 50S ribosomal protein L25 | 21812 | 5.15 | 16 | 230 | Frankia-9 | F201372.dat | 768 | 88 | 13 | 13 | 29 | 22 | 22 | 24 | 22 | 25 | 0.188% | 20 | 19 | 23 | 29 | 24 | 0.178% | 1.00 | 0.00 | 5.0E-01 | Red |
| WP_011606082.1 | FRAAL4968 | tryptophan synthase subunit beta | 46026 | 5.36 | 15 | 144 | Frankia-8 | F201371.dat | 768 | 37 | 13 | 11 | 17 | 16 | 12 | 15 | 16 | 13 | 0.056% | 12 | 15 | 17 | 14 | 14 | 0.053% | 1.00 | 0.00 | 5.0E-01 | Red |
| WP_011607031.1 | FRAAL5975 | type 11 methyltransferase | 27364 | 9.54 | 14 | 138 | Frankia-9 | F201372.dat | 738 | 58 | 13 | 13 | 17 | 12 | 13 | 14 | 16 | 14 | 0.090% | 14 | 15 | 9 | 17 | 14 | 0.085% | 1.00 | 0.00 | 5.0E-01 | Red |
| WP_041940316.1 | FRAAL1983 | 3-hydroxybutyryl-CoA dehydrogenase | 31620 | 5.28 | 14 | 98 | Frankia-3 | F201366.dat | 445 | 46 | 11 | 11 | 11 | 9 | 10 | 11 | 10 | 9 | 0.055% | 9 | 9 | 10 | 8 | 13 | 0.052% | 1.00 | 0.00 | 5.0E-01 | Red |
| WP_050997253.1 | FRAAL5902 | glycogen branching protein | 94733 | 5.63 | 13 | 64 | Frankia-8 | F201371.dat | 457 | 13 | 9 | 9 | 11 | 8 | 8 | 7 | 5 | 4 | 0.012% | 6 | 4 | 11 | 3 | 8 | 0.011% | 1.00 | 0.00 | 5.0E-01 | Red |
| WP_011606828.1 | FRAAL5755 | bifunctional riboflavin kinase/FMN adenylyltransferase | 33538 | 5.95 | 12 | 120 | Frankia-8 | F201371.dat | 527 | 35 | 9 | 9 | 14 | 11 | 13 | 13 | 12 | 11 | 0.064% | 8 | 13 | 14 | 13 | 12 | 0.060% | 1.00 | 0.00 | 5.0E-01 | Red |
| WP_011602264.1 | FRAAL1037 | NADH dehydrogenase subunit F | 47344 | 6.03 | 12 | 64 | Frankia-8 | F201371.dat | 415 | 19 | 8 | 8 | 8 | 7 | 6 | 6 | 6 | 7 | 0.024% | 6 | 7 | 8 | 5 | 6 | 0.023% | 1.00 | 0.00 | 5.0E-01 | Red |
| WP_041938879.1 | FRAAL1228 | glucose-1-phosphate thymidylyltransferase | 38068 | 5.25 | 11 | 56 | Frankia-8 | F201371.dat | 355 | 34 | 8 | 8 | 9 | 8 | 6 | 4 | 4 | 6 | 0.026% | 3 | 6 | 9 | 5 | 5 | 0.025% | 1.00 | 0.00 | 5.0E-01 | Red |
| WP_011606014.1 | FRAAL4900 | CoA-binding protein | 94833 | 5.90 | 11 | 32 | Frankia-8 | F201371.dat | 268 | 9 | 5 | 5 | 5 | 3 | 3 | 4 | 3 | 3 | 0.006% | 2 | 2 | 5 | 5 | 2 | 0.006% | 1.00 | 0.00 | 5.0E-01 | Red |
| WP_011605807.1 | FRAAL4691 | hypothetical protein | 82638 | 7.72 | 10 | 36 | Frankia-8 | F201371.dat | 203 | 8 | 7 | 7 | 8 | 7 | 0 | 4 | 4 | 3 | 0.008% | 0 | 3 | 8 | 3 | 4 | 0.007% | 1.00 | 0.00 | 5.0E-01 | Red |
| WP_011603079.1 | FRAAL1902 | dihydroorotate oxidase | 32693 | 4.84 | 9 | 72 | Frankia-8 | F201371.dat | 475 | 36 | 8 | 8 | 9 | 7 | 6 | 8 | 9 | 6 | 0.039% | 6 | 6 | 9 | 8 | 7 | 0.037% | 1.00 | 0.00 | 5.0E-01 | Red |
| WP_011607624.1 | FRAAL6587 | tRNA(Ile)-lysidine synthetase | 65075 | 5.87 | 9 | 86 | Frankia-1 | F201364.dat | 435 | 16 | 9 | 9 | 10 | 10 | 8 | 8 | 11 | 6 | 0.024% | 9 | 8 | 9 | 9 | 8 | 0.022% | 1.00 | 0.00 | 5.0E-01 | Red |
| WP_011604967.1 | FRAAL3830 | branched-chain amino acid ABC transporter substrate-binding protein | 38927 | 5.39 | 9 | 70 | Frankia-7 | F201370.dat | 431 | 32 | 8 | 8 | 8 | 8 | 6 | 7 | 8 | 6 | 0.032% | 5 | 8 | 8 | 9 | 5 | 0.030% | 1.00 | 0.00 | 5.0E-01 | Red |
| WP_011603584.1 | FRAAL2426 | aldose-1-epimerase | 32830 | 5.29 | 9 | 70 | Frankia-3 | F201366.dat | 393 | 29 | 7 | 7 | 8 | 7 | 7 | 8 | 8 | 5 | 0.038% | 8 | 7 | 8 | 7 | 5 | 0.036% | 1.00 | 0.00 | 5.0E-01 | Red |
| WP_011603056.1 | FRAAL1879 | hypothetical protein | 25246 | 8.02 | 9 | 56 | Frankia-5 | F201368.dat | 289 | 38 | 7 | 7 | 7 | 5 | 5 | 6 | 5 | 7 | 0.040% | 5 | 5 | 8 | 5 | 5 | 0.037% | 1.00 | 0.00 | 5.0E-01 | Red |
| WP_011602439.1 | FRAAL1238 | ABC transporter | 54169 | 5.14 | 8 | 52 | Frankia-8 | F201371.dat | 321 | 23 | 7 | 7 | 8 | 6 | 4 | 5 | 7 | 4 | 0.017% | 3 | 6 | 8 | 6 | 3 | 0.016% | 1.00 | 0.00 | 5.0E-01 | Red |
| WP_011605804.1 | FRAAL4688 | clavaminate synthase | 34963 | 5.54 | 8 | 32 | Frankia-4 | F201367.dat | 224 | 18 | 4 | 4 | 4 | 1 | 3 | 3 | 4 | 5 | 0.016% | 2 | 4 | 4 | 3 | 3 | 0.015% | 1.00 | 0.00 | 5.0E-01 | Red |
| WP_011606086.1 | FRAAL4972 | anthranilate synthase subunit I | 54178 | 5.00 | 8 | 34 | Frankia-2 | F201365.dat | 213 | 15 | 5 | 5 | 5 | 3 | 5 | 3 | 2 | 4 | 0.011% | 4 | 4 | 3 | 4 | 2 | 0.011% | 1.00 | 0.00 | 5.0E-01 | Red |
| WP_011604670.1 | FRAAL3529 | ABC transporter | 95869 | 7.25 | 7 | 46 | Frankia-6 | F201369.dat | 286 | 11 | 7 | 7 | 7 | 5 | 5 | 5 | 4 | 4 | 0.009% | 7 | 2 | 5 | 5 | 4 | 0.008% | 1.00 | 0.00 | 5.0E-01 | Red |
| WP_011602420.1 | FRAAL1218 | hypothetical protein | 51554 | 4.42 | 7 | 46 | Frankia-8 | F201371.dat | 281 | 20 | 6 | 6 | 6 | 5 | 4 | 4 | 5 | 5 | 0.016% | 4 | 5 | 6 | 4 | 4 | 0.015% | 1.00 | 0.00 | 5.0E-01 | Red |
| WP_011601303.1 | FRAAL0035 | conjugal transfer protein TraC | 72198 | 4.82 | 7 | 40 | Frankia-4 | F201367.dat | 272 | 10 | 5 | 5 | 5 | 4 | 5 | 3 | 5 | 3 | 0.010% | 5 | 3 | 5 | 3 | 4 | 0.009% | 1.00 | 0.00 | 5.0E-01 | Red |
| WP_011606631.1 | FRAAL5547 | enoyl-CoA hydratase | 27147 | 5.89 | 7 | 58 | Frankia-3 | F201366.dat | 255 | 42 | 7 | 7 | 8 | 7 | 4 | 8 | 5 | 5 | 0.038% | 5 | 8 | 6 | 4 | 6 | 0.036% | 1.00 | 0.00 | 5.0E-01 | Red |
| WP_011606552.1 | FRAAL5311 | ATPase AAA | 75730 | 6.18 | 7 | 26 | Frankia-3 | F201366.dat | 228 | 8 | 4 | 4 | 4 | 3 | 2 | 4 | 2 | 2 | 0.006% | 1 | 5 | 3 | 1 | 3 | 0.006% | 1.00 | 0.00 | 5.0E-01 | Red |
| WP_041939613.1 | FRAAL5023 | 2-methylcitrate dehydratase | 54215 | 6.40 | 7 | 36 | Frankia-9 | F201372.dat | 225 | 15 | 6 | 6 | 6 | 5 | 4 | 3 | 3 | 3 | 0.012% | 4 | 4 | 3 | 6 | 1 | 0.011% | 1.00 | 0.00 | 5.0E-01 | Red |
| WP_041940220.1 | FRAAL1391 | methyltransferase | 31939 | 5.42 | 6 | 24 | Frankia-10 | F201373.dat | 449 | 36 | 8 | 3 | 4 | 2 | 2 | 2 | 3 | 3 | 0.013% | 1 | 2 | 3 | 2 | 4 | 0.013% | 1.00 | 0.00 | 5.0E-01 | Red |
| WP_011605712.1 | FRAAL4596 | Zn-dependent hydrolase | 24228 | 4.88 | 6 | 52 | Frankia-5 | F201368.dat | 238 | 28 | 5 | 5 | 6 | 5 | 6 | 4 | 5 | 6 | 0.038% | 4 | 5 | 5 | 5 | 7 | 0.036% | 1.00 | 0.00 | 5.0E-01 | Red |
| WP_011606568.1 | FRAAL5483 | enoyl-CoA hydratase | 28022 | 5.84 | 6 | 20 | Frankia-8 | F201371.dat | 177 | 18 | 4 | 4 | 3 | 4 | 0 | 2 | 4 | 0 | 0.013% | 1 | 4 | 3 | 1 | 1 | 0.012% | 1.00 | 0.00 | 5.0E-01 | Red |
| WP_011604836.1 | FRAAL3697 | methyltransferase | 28584 | 5.19 | 6 | 12 | Frankia-7 | F201370.dat | 123 | 10 | 2 | 2 | 2 | 1 | 2 | 2 | 1 | 0 | 0.007% | 0 | 2 | 2 | 1 | 1 | 0.007% | 1.00 | 0.00 | 5.0E-01 | Red |
| WP_011602874.1 | FRAAL1687 | hypothetical protein | 35955 | 4.80 | 5 | 44 | Frankia-5 | F201368.dat | 234 | 15 | 4 | 4 | 5 | 4 | 4 | 5 | 4 | 5 | 0.022% | 4 | 4 | 6 | 4 | 4 | 0.021% | 1.00 | 0.00 | 5.0E-01 | Red |
| WP_041940366.1 | FRAAL2343 | methylcrotonoyl-CoA carboxylase | 56916 | 6.51 | 5 | 30 | Frankia-8 | F201371.dat | 214 | 10 | 4 | 4 | 4 | 3 | 3 | 3 | 3 | 3 | 0.009% | 2 | 3 | 4 | 3 | 3 | 0.009% | 1.00 | 0.00 | 5.0E-01 | Red |
| WP_041939296.1 | FRAAL3171 | oxidoreductase | 41599 | 4.79 | 5 | 32 | Frankia-2 | F201365.dat | 198 | 16 | 4 | 4 | 4 | 3 | 4 | 3 | 3 | 3 | 0.014% | 3 | 3 | 4 | 3 | 3 | 0.013% | 1.00 | 0.00 | 5.0E-01 | Red |
| WP_050997318.1 | FRAAL0961 | ABC transporter | 42523 | 6.46 | 5 | 22 | Frankia-3 | F201366.dat | 126 | 8 | 3 | 3 | 3 | 1 | 2 | 3 | 2 | 3 | 0.009% | 2 | 2 | 3 | 3 | 1 | 0.009% | 1.00 | 0.00 | 5.0E-01 | Red |
| WP_011604436.1 | FRAAL3287 | hypothetical protein | 48723 | 8.64 | 5 | 18 | Frankia-1 | F201364.dat | 98 | 9 | 3 | 3 | 3 | 3 | 2 | 1 | 1 | 2 | 0.007% | 3 | 1 | 2 | 2 | 1 | 0.006% | 1.00 | 0.00 | 5.0E-01 | Red |
| WP_011604280.1 | FRAAL3131 | amidohydrolase | 43910 | 5.23 | 5 | 10 | Frankia-5 | F201368.dat | 96 | 11 | 3 | 3 | 3 | 0 | 0 | 2 | 0 | 3 | 0.004% | 0 | 1 | 0 | 2 | 2 | 0.004% | 1.00 | 0.00 | 5.0E-01 | Red |
| WP_011607530.1 | FRAAL6489 | hypothetical protein | 37376 | 5.23 | 5 | 10 | Frankia-5 | F201368.dat | 69 | 11 | 3 | 3 | 3 | 0 | 0 | 1 | 1 | 3 | 0.005% | 0 | 0 | 2 | 1 | 2 | 0.005% | 1.00 | 0.00 | 5.0E-01 | Red |
| WP_011602675.1 | FRAAL1485 | ABC transporter permease | 77946 | 9.93 | 4 | 28 | Frankia-6 | F201369.dat | 204 | 10 | 4 | 4 | 4 | 3 | 2 | 3 | 3 | 3 | 0.006% | 4 | 3 | 2 | 3 | 2 | 0.006% | 1.00 | 0.00 | 5.0E-01 | Red |
| WP_011606979.1 | FRAAL5919 | endonuclease NucS | 23832 | 5.38 | 4 | 24 | Frankia-7 | F201370.dat | 193 | 17 | 3 | 3 | 3 | 3 | 3 | 2 | 2 | 2 | 0.018% | 2 | 3 | 3 | 2 | 2 | 0.017% | 1.00 | 0.00 | 5.0E-01 | Red |
| WP_011603089.1 | FRAAL1914 | dihydrofolate synthase | 49547 | 4.36 | 4 | 18 | Frankia-9 | F201372.dat | 164 | 6 | 3 | 3 | 3 | 3 | 2 | 2 | 1 | 1 | 0.006% | 1 | 3 | 2 | 3 | 0 | 0.006% | 1.00 | 0.00 | 5.0E-01 | Red |
| WP_011607076.1 | FRAAL6024 | glutamate-1-semialdehyde 2.1-aminomutase | 50372 | 5.30 | 4 | 6 | Frankia-5 | F201368.dat | 132 | 9 | 3 | 3 | 3 | 0 | 0 | 0 | 0 | 3 | 0.002% | 0 | 0 | 2 | 1 | 0 | 0.002% | 1.00 | 0.00 | 5.0E-01 | Red |
| WP_041940919.1 | FRAAL5353 | ATP-binding protein | 8157 | 10.20 | 4 | 10 | Frankia-9 | F201372.dat | 123 | 45 | 3 | 3 | 4 | 1 | 0 | 0 | 2 | 2 | 0.022% | 0 | 1 | 0 | 4 | 0 | 0.021% | 1.00 | 0.00 | 5.0E-01 | Red |
| WP_011607525.1 | FRAAL6484 | hypothetical protein | 29990 | 5.76 | 4 | 10 | Frankia-2 | F201365.dat | 111 | 9 | 2 | 2 | 2 | 2 | 2 | 1 | 0 | 0 | 0.006% | 1 | 1 | 2 | 1 | 0 | 0.006% | 1.00 | 0.00 | 5.0E-01 | Red |
| WP_011602464.1 | FRAAL1263 | radical SAM protein | 96806 | 5.69 | 4 | 6 | Frankia-5 | F201368.dat | 96 | 3 | 2 | 2 | 2 | 0 | 0 | 1 | 0 | 2 | 0.001% | 0 | 0 | 2 | 0 | 1 | 0.001% | 1.00 | 0.00 | 5.0E-01 | Red |
| WP_011602346.1 | FRAAL1141 | hypothetical protein | 45328 | 4.98 | 4 | 14 | Frankia-7 | F201370.dat | 87 | 5 | 2 | 2 | 2 | 0 | 2 | 2 | 2 | 1 | 0.006% | 0 | 2 | 2 | 1 | 2 | 0.005% | 1.00 | 0.00 | 5.0E-01 | Red |
| WP_050997151.1 | FRAAL3954 | hypothetical protein | 177520 | 5.58 | 4 | 8 | Frankia-5 | F201368.dat | 84 | 1 | 2 | 2 | 2 | 2 | 0 | 0 | 0 | 2 | 0.001% | 1 | 0 | 2 | 1 | 0 | 0.001% | 1.00 | 0.00 | 5.0E-01 | Red |
| WP_050997319.1 | FRAAL0969 | AP endonuclease | 30552 | 5.76 | 3 | 28 | Frankia-3 | F201366.dat | 223 | 15 | 3 | 3 | 3 | 2 | 3 | 3 | 3 | 3 | 0.016% | 3 | 3 | 2 | 3 | 3 | 0.015% | 1.00 | 0.00 | 5.0E-01 | Red |
| WP_011604567.1 | FRAAL3422 | short-chain dehydrogenase | 32168 | 6.40 | 3 | 34 | Frankia-4 | F201367.dat | 214 | 18 | 3 | 3 | 3 | 4 | 3 | 4 | 3 | 3 | 0.019% | 3 | 4 | 3 | 3 | 4 | 0.018% | 1.00 | 0.00 | 5.0E-01 | Red |
| WP_041940521.1 | FRAAL3166 | amidase | 21984 | 5.60 | 3 | 18 | Frankia-6 | F201369.dat | 163 | 16 | 3 | 3 | 3 | 1 | 3 | 2 | 1 | 2 | 0.015% | 3 | 2 | 2 | 0 | 2 | 0.014% | 1.00 | 0.00 | 5.0E-01 | Red |
| WP_050997154.1 | FRAAL3962 | hypothetical protein | 28805 | 4.53 | 3 | 12 | Frankia-8 | F201371.dat | 152 | 11 | 3 | 3 | 3 | 0 | 1 | 3 | 1 | 1 | 0.007% | 0 | 1 | 3 | 1 | 1 | 0.007% | 1.00 | 0.00 | 5.0E-01 | Red |
| WP_041939292.1 | FRAAL3150 | hypothetical protein | 34782 | 8.62 | 3 | 12 | Frankia-8 | F201371.dat | 128 | 10 | 3 | 3 | 3 | 2 | 0 | 1 | 1 | 2 | 0.006% | 0 | 2 | 3 | 1 | 0 | 0.006% | 1.00 | 0.00 | 5.0E-01 | Red |
| WP_011604571.1 | FRAAL3426 | short-chain dehydrogenase | 25895 | 5.27 | 3 | 4 | Frankia-8 | F201371.dat | 126 | 11 | 2 | 2 | 2 | 0 | 0 | 0 | 0 | 2 | 0.003% | 0 | 0 | 2 | 0 | 0 | 0.003% | 1.00 | 0.00 | 5.0E-01 | Red |
| WP_050997408.1 | FRAAL4240 | Puromycin resistance protein pur8 | 49966 | 7.81 | 3 | 26 | Frankia-7 | F201370.dat | 122 | 6 | 3 | 3 | 4 | 2 | 3 | 3 | 3 | 2 | 0.009% | 3 | 4 | 2 | 2 | 2 | 0.009% | 1.00 | 0.00 | 5.0E-01 | Red |
| WP_011605364.1 | FRAAL4238 | chromosome partitioning protein ParA | 34405 | 9.54 | 3 | 14 | Frankia-4 | F201367.dat | 117 | 6 | 2 | 2 | 2 | 2 | 1 | 2 | 2 | 0 | 0.007% | 2 | 2 | 1 | 1 | 1 | 0.007% | 1.00 | 0.00 | 5.0E-01 | Red |
| WP_050997475.1 | FRAAL6710 | dihydropteroate synthase | 34038 | 6.00 | 3 | 10 | Frankia-2 | F201365.dat | 115 | 10 | 2 | 2 | 2 | 1 | 2 | 1 | 1 | 0 | 0.005% | 1 | 0 | 2 | 1 | 1 | 0.005% | 1.00 | 0.00 | 5.0E-01 | Red |
| WP_011602270.1 | FRAAL1043 | NADH:ubiquinone oxidoreductase subunit L | 69833 | 8.56 | 3 | 24 | Frankia-1 | F201364.dat | 106 | 4 | 3 | 3 | 4 | 4 | 1 | 4 | 2 | 1 | 0.006% | 3 | 2 | 3 | 2 | 2 | 0.006% | 1.00 | 0.00 | 5.0E-01 | Red |
| WP_050997191.1 | FRAAL4598 | hypothetical protein | 88201 | 7.32 | 3 | 14 | Frankia-2 | F201365.dat | 105 | 4 | 3 | 3 | 2 | 1 | 2 | 2 | 1 | 1 | 0.003% | 1 | 2 | 2 | 1 | 1 | 0.003% | 1.00 | 0.00 | 5.0E-01 | Red |
| WP_011601312.1 | FRAAL0044 | hypothetical protein | 23091 | 5.12 | 3 | 16 | Frankia-7 | F201370.dat | 103 | 8 | 2 | 2 | 2 | 1 | 1 | 2 | 2 | 2 | 0.012% | 1 | 2 | 2 | 2 | 1 | 0.012% | 1.00 | 0.00 | 5.0E-01 | Red |
| WP_011604483.1 | FRAAL3336 | hypothetical protein | 45979 | 6.61 | 3 | 10 | Frankia-7 | F201370.dat | 99 | 4 | 2 | 2 | 2 | 1 | 0 | 1 | 2 | 1 | 0.004% | 0 | 2 | 2 | 1 | 0 | 0.004% | 1.00 | 0.00 | 5.0E-01 | Red |
| WP_011603184.1 | FRAAL2012 | cytidine deaminase | 13358 | 4.76 | 3 | 10 | Frankia-8 | F201371.dat | 98 | 24 | 2 | 2 | 2 | 1 | 1 | 1 | 1 | 1 | 0.013% | 1 | 1 | 2 | 0 | 1 | 0.013% | 1.00 | 0.00 | 5.0E-01 | Red |
| WP_050997360.1 | FRAAL2805 | 3-hydroxyisobutyryl-CoA hydrolase | 38196 | 5.25 | 3 | 10 | Frankia-8 | F201371.dat | 97 | 5 | 2 | 2 | 1 | 1 | 1 | 1 | 1 | 1 | 0.005% | 1 | 2 | 1 | 0 | 1 | 0.004% | 1.00 | 0.00 | 5.0E-01 | Red |
| WP_011603076.1 | FRAAL1899 | transcriptional regulator | 49413 | 10.14 | 3 | 12 | Frankia-3 | F201366.dat | 86 | 5 | 2 | 2 | 2 | 1 | 1 | 2 | 1 | 1 | 0.004% | 1 | 2 | 1 | 1 | 1 | 0.004% | 1.00 | 0.00 | 5.0E-01 | Red |
| WP_011601546.1 | FRAAL0287 | GntR family transcriptional regulator | 28590 | 5.84 | 3 | 8 | Frankia-8 | F201371.dat | 85 | 10 | 2 | 2 | 2 | 1 | 0 | 0 | 2 | 1 | 0.005% | 0 | 0 | 2 | 1 | 1 | 0.005% | 1.00 | 0.00 | 5.0E-01 | Red |
| WP_011602513.1 | FRAAL1316 | hypothetical protein | 17806 | 4.03 | 3 | 14 | Frankia-1 | F201364.dat | 80 | 19 | 3 | 3 | 3 | 3 | 0 | 2 | 2 | 0 | 0.014% | 0 | 1 | 2 | 2 | 2 | 0.013% | 1.00 | 0.00 | 5.0E-01 | Red |
| WP_041939726.1 | FRAAL5558 | 3-ketoacyl-ACP reductase | 26349 | 4.83 | 3 | 8 | Frankia-8 | F201371.dat | 73 | 15 | 2 | 2 | 2 | 0 | 1 | 1 | 1 | 1 | 0.005% | 0 | 0 | 2 | 0 | 2 | 0.005% | 1.00 | 0.00 | 5.0E-01 | Red |
| WP_011602223.1 | FRAAL0996 | 3-oxoacyl-ACP synthase | 32359 | 5.65 | 3 | 4 | Frankia-2 | F201365.dat | 73 | 6 | 1 | 1 | 1 | 1 | 1 | 0 | 0 | 0 | 0.002% | 0 | 0 | 2 | 0 | 0 | 0.002% | 1.00 | 0.00 | 5.0E-01 | Red |
| WP_041939760.1 | FRAAL5796 | chromosome segregation protein SMC | 136328 | 5.31 | 3 | 6 | Frankia-8 | F201371.dat | 70 | 1 | 2 | 2 | 2 | 0 | 2 | 1 | 0 | 0 | 0.001% | 1 | 0 | 2 | 0 | 0 | 0.001% | 1.00 | 0.00 | 5.0E-01 | Red |
| WP_041939682.1 | FRAAL5364 | ethyl tert-butyl ether degradation protein EthD | 10506 | 4.95 | 3 | 16 | Frankia-10 | F201373.dat | 67 | 20 | 2 | 2 | 2 | 1 | 1 | 2 | 2 | 2 | 0.027% | 2 | 2 | 1 | 1 | 2 | 0.026% | 1.00 | 0.00 | 5.0E-01 | Red |
| WP_041939634.1 | FRAAL5099 | GDP-mannose-dependent alpha-(1-6)-phosphatidylinositol monomannoside mannosyltransferase | 39985 | 9.63 | 3 | 6 | Frankia-10 | F201373.dat | 56 | 6 | 2 | 2 | 2 | 0 | 1 | 0 | 1 | 1 | 0.003% | 1 | 0 | 0 | 0 | 2 | 0.003% | 1.00 | 0.00 | 5.0E-01 | Red |
| WP_011606619.1 | FRAAL5535 | mechanosensitive ion channel protein MscS | 32063 | 6.11 | 2 | 8 | Frankia-7 | F201370.dat | 166 | 10 | 2 | 2 | 2 | 1 | 1 | 1 | 1 | 0 | 0.004% | 0 | 2 | 0 | 1 | 1 | 0.004% | 1.00 | 0.00 | 5.0E-01 | Red |
| WP_041938918.1 | FRAAL1439 | glycosyl transferase | 54412 | 9.44 | 2 | 10 | Frankia-3 | F201366.dat | 149 | 8 | 2 | 2 | 2 | 1 | 1 | 2 | 1 | 0 | 0.003% | 1 | 1 | 1 | 1 | 1 | 0.003% | 1.00 | 0.00 | 5.0E-01 | Red |
| WP_011601949.1 | FRAAL0707 | molybdopterin-guanine dinucleotide biosynthesis protein MobA | 34983 | 5.50 | 2 | 8 | Frankia-10 | F201373.dat | 138 | 10 | 2 | 2 | 2 | 1 | 1 | 0 | 1 | 1 | 0.004% | 0 | 0 | 1 | 1 | 2 | 0.004% | 1.00 | 0.00 | 5.0E-01 | Red |
| WP_011607825.1 | FRAAL6792 | chemotaxis protein CheY | 16790 | 4.56 | 2 | 8 | Frankia-7 | F201370.dat | 130 | 20 | 2 | 2 | 2 | 0 | 1 | 1 | 1 | 1 | 0.009% | 0 | 2 | 0 | 1 | 1 | 0.008% | 1.00 | 0.00 | 5.0E-01 | Red |
| WP_011603960.1 | FRAAL2809 | xylanase | 20612 | 6.07 | 2 | 24 | Frankia-8 | F201371.dat | 123 | 14 | 2 | 2 | 3 | 2 | 2 | 2 | 3 | 3 | 0.021% | 2 | 2 | 3 | 3 | 2 | 0.020% | 1.00 | 0.00 | 5.0E-01 | Red |
| WP_009738257.1 | FRAAL3418 | acyl-CoA dehydrogenase | 43562 | 5.18 | 2 | 8 | Frankia-5 | F201368.dat | 121 | 6 | 2 | 2 | 2 | 1 | 0 | 0 | 1 | 2 | 0.003% | 0 | 1 | 1 | 1 | 1 | 0.003% | 1.00 | 0.00 | 5.0E-01 | Red |
| WP_041940835.1 | FRAAL4883 | acyl-CoA dehydrogenase | 36457 | 5.04 | 2 | 18 | Frankia-5 | F201368.dat | 119 | 9 | 2 | 2 | 2 | 1 | 2 | 2 | 2 | 2 | 0.009% | 2 | 2 | 2 | 2 | 1 | 0.008% | 1.00 | 0.00 | 5.0E-01 | Red |
| WP_050997474.1 | FRAAL6709 | hypothetical protein | 14246 | 5.76 | 2 | 12 | Frankia-7 | F201370.dat | 100 | 18 | 2 | 2 | 2 | 1 | 1 | 1 | 1 | 2 | 0.015% | 1 | 2 | 1 | 1 | 1 | 0.014% | 1.00 | 0.00 | 5.0E-01 | Red |
| WP_011604661.1 | FRAAL3520 | hypothetical protein | 30497 | 5.20 | 2 | 2 | Frankia-8 | F201371.dat | 93 | 8 | 1 | 1 | 1 | 0 | 0 | 0 | 0 | 1 | 0.001% | 0 | 0 | 1 | 0 | 0 | 0.001% | 1.00 | 0.00 | 5.0E-01 | Red |
| WP_011602095.1 | FRAAL0861 | hypothetical protein | 182163 | 5.14 | 2 | 4 | Frankia-1 | F201364.dat | 88 | 1 | 2 | 2 | 2 | 2 | 0 | 0 | 0 | 0 | 0.000% | 0 | 0 | 1 | 1 | 0 | 0.000% | 1.00 | 0.00 | 5.0E-01 | Red |
| WP_011606785.1 | FRAAL5711 | hypothetical protein | 27659 | 4.97 | 2 | 10 | Frankia-10 | F201373.dat | 86 | 10 | 2 | 2 | 2 | 1 | 1 | 0 | 1 | 2 | 0.006% | 0 | 1 | 1 | 1 | 2 | 0.006% | 1.00 | 0.00 | 5.0E-01 | Red |
| WP_011605252.1 | FRAAL4124 | hypothetical protein | 19037 | 8.57 | 2 | 16 | Frankia-9 | F201372.dat | 85 | 13 | 2 | 2 | 2 | 2 | 1 | 2 | 2 | 1 | 0.015% | 1 | 1 | 2 | 2 | 2 | 0.014% | 1.00 | 0.00 | 5.0E-01 | Red |
| WP_050997076.1 | FRAAL2463 | NUDIX hydrolase | 18554 | 5.34 | 2 | 14 | Frankia-7 | F201370.dat | 83 | 14 | 2 | 2 | 2 | 2 | 2 | 1 | 1 | 1 | 0.013% | 1 | 2 | 1 | 1 | 2 | 0.013% | 1.00 | 0.00 | 5.0E-01 | Red |
| WP_011602803.1 | FRAAL1614 | hypothetical protein | 38289 | 10.01 | 2 | 8 | Frankia-7 | F201370.dat | 82 | 5 | 2 | 2 | 2 | 0 | 1 | 2 | 1 | 0 | 0.004% | 0 | 2 | 2 | 0 | 0 | 0.004% | 1.00 | 0.00 | 5.0E-01 | Red |
| WP_050997012.1 | FRAAL1189 | succinate dehydrogenase | 16683 | 10.81 | 2 | 10 | Frankia-8 | F201371.dat | 81 | 11 | 2 | 2 | 2 | 1 | 1 | 1 | 1 | 1 | 0.011% | 1 | 1 | 2 | 1 | 0 | 0.010% | 1.00 | 0.00 | 5.0E-01 | Red |
| WP_011603080.1 | FRAAL1903 | hypothetical protein | 20150 | 5.94 | 2 | 6 | Frankia-1 | F201364.dat | 79 | 13 | 1 | 1 | 1 | 1 | 0 | 1 | 0 | 1 | 0.005% | 1 | 0 | 1 | 0 | 1 | 0.005% | 1.00 | 0.00 | 5.0E-01 | Red |
| WP_041939309.1 | FRAAL3214 | polysaccharide deacetylase | 31509 | 10.08 | 2 | 8 | Frankia-3 | F201366.dat | 78 | 6 | 2 | 2 | 2 | 1 | 1 | 2 | 0 | 0 | 0.005% | 0 | 1 | 1 | 1 | 1 | 0.004% | 1.00 | 0.00 | 5.0E-01 | Red |
| WP_011606692.1 | FRAAL5611 | hypothetical protein | 46857 | 8.85 | 2 | 10 | Frankia-7 | F201370.dat | 78 | 4 | 2 | 2 | 2 | 1 | 1 | 1 | 1 | 1 | 0.004% | 1 | 2 | 0 | 1 | 1 | 0.004% | 1.00 | 0.00 | 5.0E-01 | Red |
| WP_041940927.1 | FRAAL5400 | glycosyl transferase | 46508 | 7.82 | 2 | 10 | Frankia-10 | F201373.dat | 77 | 4 | 2 | 2 | 1 | 1 | 1 | 1 | 1 | 1 | 0.004% | 1 | 1 | 1 | 1 | 1 | 0.004% | 1.00 | 0.00 | 5.0E-01 | Red |
| WP_011606194.1 | FRAAL5085 | hypothetical protein | 19928 | 5.35 | 2 | 18 | Frankia-1 | F201364.dat | 77 | 11 | 2 | 2 | 2 | 2 | 2 | 1 | 2 | 2 | 0.016% | 1 | 2 | 2 | 2 | 2 | 0.015% | 1.00 | 0.00 | 5.0E-01 | Red |
| WP_041939820.1 | FRAAL6064 | antibiotic biosynthesis monooxygenase | 15486 | 5.75 | 2 | 12 | Frankia-4 | F201367.dat | 76 | 15 | 2 | 2 | 2 | 1 | 1 | 1 | 2 | 1 | 0.014% | 2 | 0 | 1 | 2 | 1 | 0.013% | 1.00 | 0.00 | 5.0E-01 | Red |
| WP_041938714.1 | FRAAL0533 | hypothetical protein | 13972 | 4.72 | 2 | 10 | Frankia-4 | F201367.dat | 70 | 17 | 2 | 2 | 2 | 0 | 2 | 0 | 2 | 1 | 0.013% | 1 | 1 | 1 | 1 | 1 | 0.012% | 1.00 | 0.00 | 5.0E-01 | Red |
| WP_050997285.1 | FRAAL6482 | hypothetical protein | 43551 | 5.30 | 2 | 10 | Frankia-7 | F201370.dat | 53 | 7 | 2 | 2 | 2 | 1 | 1 | 1 | 1 | 1 | 0.004% | 1 | 2 | 1 | 1 | 0 | 0.004% | 1.00 | 0.00 | 5.0E-01 | Red |
| WP_041938954.1 | FRAAL1627 | two-component system sensor histidine kinase | 39543 | 6.11 | 2 | 4 | Frankia-4 | F201367.dat | 52 | 4 | 2 | 2 | 2 | 0 | 0 | 0 | 2 | 0 | 0.002% | 0 | 1 | 0 | 1 | 0 | 0.002% | 1.00 | 0.00 | 5.0E-01 | Red |
| WP_050997061.1 | FRAAL2313 | metallophosphoesterase | 33046 | 5.48 | 2 | 8 | Frankia-6 | F201369.dat | 52 | 5 | 2 | 2 | 2 | 0 | 0 | 1 | 2 | 1 | 0.004% | 2 | 0 | 1 | 0 | 1 | 0.004% | 1.00 | 0.00 | 5.0E-01 | Red |
| WP_011607564.1 | FRAAL6523 | 2-C-methyl-D-erythritol 2.4-cyclodiphosphate synthase | 15967 | 5.85 | 2 | 2 | Frankia-1 | F201364.dat | 51 | 9 | 1 | 1 | 1 | 1 | 0 | 0 | 0 | 0 | 0.002% | 0 | 0 | 1 | 0 | 0 | 0.002% | 1.00 | 0.00 | 5.0E-01 | Red |
| WP_011605451.1 | FRAAL4326 | iron utilization protein | 35815 | 10.84 | 2 | 2 | Frankia-8 | F201371.dat | 50 | 4 | 1 | 1 | 1 | 0 | 1 | 0 | 0 | 0 | 0.001% | 0 | 0 | 1 | 0 | 0 | 0.001% | 1.00 | 0.00 | 5.0E-01 | Red |
| WP_041940983.1 | FRAAL5700 | hypothetical protein | 28263 | 6.86 | 2 | 4 | Frankia-8 | F201371.dat | 48 | 7 | 2 | 2 | 2 | 0 | 0 | 1 | 1 | 0 | 0.003% | 0 | 0 | 2 | 0 | 0 | 0.002% | 1.00 | 0.00 | 5.0E-01 | Red |
| WP_050997428.1 | FRAAL4930 | monooxygenase | 41621 | 5.55 | 2 | 2 | Frankia-10 | F201373.dat | 36 | 5 | 1 | 1 | 1 | 0 | 1 | 0 | 0 | 0 | 0.001% | 0 | 0 | 0 | 0 | 1 | 0.001% | 1.00 | 0.00 | 5.0E-01 | Red |
| WP_041939105.1 | FRAAL2319 | hypothetical protein | 54468 | 6.44 | 2 | 2 | Frankia-2 | F201365.dat | 33 | 2 | 1 | 1 | 1 | 0 | 1 | 0 | 0 | 0 | 0.001% | 0 | 0 | 0 | 0 | 1 | 0.001% | 1.00 | 0.00 | 5.0E-01 | Red |
| WP_041939458.1 | FRAAL4150 | hypothetical protein | 163487 | 5.84 | 2 | 2 | Frankia-8 | F201371.dat | 32 | 1 | 1 | 1 | 1 | 1 | 0 | 0 | 0 | 0 | 0.000% | 0 | 0 | 1 | 0 | 0 | 0.000% | 1.00 | 0.00 | 5.0E-01 | Red |
| WP_011603541.1 | FRAAL2380 | citrate synthase | 53462 | 6.86 | 2 | 2 | Frankia-5 | F201368.dat | 32 | 3 | 1 | 1 | 1 | 0 | 0 | 0 | 0 | 1 | 0.001% | 0 | 0 | 1 | 0 | 0 | 0.001% | 1.00 | 0.00 | 5.0E-01 | Red |
| WP_011605693.1 | FRAAL4577 | glucose-6-phosphate dehydrogenase | 41566 | 5.77 | 23 | 265 | Frankia-8 | F201371.dat | 1165 | 57 | 21 | 21 | 30 | 27 | 28 | 26 | 27 | 25 | 0.114% | 22 | 28 | 30 | 25 | 27 | 0.107% | -1.01 | -0.01 | 4.5E-01 | Red |
| WP_011607146.1 | FRAAL6095 | leucyl aminopeptidase | 60843 | 4.74 | 29 | 243 | Frankia-10 | F201373.dat | 1150 | 43 | 19 | 19 | 29 | 25 | 24 | 24 | 22 | 27 | 0.072% | 20 | 24 | 28 | 20 | 29 | 0.067% | -1.01 | -0.01 | 4.6E-01 | Red |
| WP_041939957.1 | FRAAL6700 | threonine synthase | 44443 | 5.25 | 27 | 233 | Frankia-8 | F201371.dat | 1105 | 73 | 21 | 21 | 29 | 25 | 22 | 22 | 23 | 25 | 0.094% | 21 | 22 | 29 | 19 | 25 | 0.088% | -1.01 | -0.01 | 4.6E-01 | Red |
| WP_011602277.1 | FRAAL1050 | 2-oxoacid ferredoxin oxidoreductase subunit beta | 37606 | 5.68 | 21 | 462 | Frankia-5 | F201368.dat | 1229 | 63 | 20 | 20 | 47 | 44 | 51 | 44 | 46 | 47 | 0.220% | 45 | 47 | 44 | 48 | 46 | 0.206% | -1.01 | -0.01 | 4.0E-01 | Red |
| WP_011602303.1 | FRAAL1087 | 30S ribosomal protein S3 | 37419 | 9.78 | 25 | 424 | Frankia-10 | F201373.dat | 1176 | 46 | 24 | 24 | 46 | 42 | 42 | 44 | 41 | 44 | 0.203% | 41 | 40 | 42 | 42 | 46 | 0.190% | -1.01 | -0.01 | 3.7E-01 | Red |
| WP_050997359.1 | FRAAL2801 | dehydrogenase | 60887 | 5.89 | 22 | 195 | Frankia-7 | F201370.dat | 780 | 47 | 17 | 17 | 22 | 20 | 22 | 19 | 18 | 19 | 0.057% | 17 | 22 | 20 | 20 | 18 | 0.054% | -1.01 | -0.01 | 4.3E-01 | Red |
| WP_011603097.1 | FRAAL1923 | ribonuclease | 139592 | 5.00 | 29 | 193 | Frankia-8 | F201371.dat | 1161 | 24 | 22 | 22 | 28 | 25 | 20 | 22 | 15 | 15 | 0.025% | 25 | 9 | 28 | 17 | 17 | 0.023% | -1.01 | -0.01 | 4.8E-01 | Red |
| WP_041938860.1 | FRAAL1144 | twin-arginine translocation pathway signal protein | 49528 | 7.68 | 17 | 171 | Frankia-1 | F201364.dat | 760 | 41 | 14 | 14 | 18 | 18 | 19 | 14 | 17 | 18 | 0.062% | 18 | 18 | 14 | 19 | 16 | 0.058% | -1.01 | -0.01 | 4.4E-01 | Red |
| WP_011605662.1 | FRAAL4544 | ABC transporter | 58214 | 6.08 | 19 | 167 | Frankia-3 | F201366.dat | 834 | 44 | 18 | 18 | 19 | 17 | 14 | 19 | 19 | 15 | 0.051% | 16 | 16 | 14 | 17 | 20 | 0.048% | -1.01 | -0.01 | 4.5E-01 | Red |
| WP_011601904.1 | FRAAL0656 | beta-mannanase | 46705 | 9.69 | 11 | 161 | Frankia-5 | F201368.dat | 658 | 26 | 11 | 11 | 17 | 14 | 17 | 17 | 16 | 17 | 0.062% | 17 | 18 | 16 | 15 | 14 | 0.058% | -1.01 | -0.01 | 4.2E-01 | Red |
| WP_050997262.1 | FRAAL6214 | magnesium chelatase | 52116 | 5.29 | 29 | 322 | Frankia-1 | F201364.dat | 1430 | 62 | 24 | 24 | 36 | 36 | 33 | 32 | 29 | 32 | 0.111% | 34 | 28 | 35 | 34 | 29 | 0.103% | -1.01 | -0.01 | 4.2E-01 | Red |
| WP_011601918.1 | FRAAL0670 | acyl-ACP desaturase | 35158 | 5.72 | 16 | 143 | Frankia-6 | F201369.dat | 505 | 50 | 13 | 13 | 15 | 16 | 13 | 14 | 15 | 14 | 0.073% | 15 | 14 | 13 | 16 | 13 | 0.068% | -1.01 | -0.01 | 4.0E-01 | Red |
| WP_011602442.1 | FRAAL1241 | hypothetical protein | 50154 | 5.95 | 19 | 137 | Frankia-1 | F201364.dat | 700 | 37 | 13 | 13 | 16 | 16 | 9 | 15 | 14 | 15 | 0.049% | 10 | 13 | 16 | 13 | 16 | 0.046% | -1.01 | -0.01 | 4.5E-01 | Red |
| WP_011602300.1 | FRAAL1081 | 50S ribosomal protein L3 | 24380 | 10.34 | 23 | 417 | Frankia-10 | F201373.dat | 1255 | 85 | 21 | 21 | 46 | 40 | 39 | 40 | 43 | 48 | 0.307% | 37 | 39 | 43 | 42 | 46 | 0.286% | -1.01 | -0.01 | 4.0E-01 | Red |
| WP_011606351.1 | FRAAL5260 | glutamate--tRNA ligase | 51203 | 5.54 | 16 | 131 | Frankia-8 | F201371.dat | 657 | 34 | 14 | 14 | 15 | 14 | 11 | 13 | 13 | 15 | 0.046% | 13 | 15 | 15 | 9 | 13 | 0.043% | -1.01 | -0.01 | 4.4E-01 | Red |
| WP_011605772.1 | FRAAL4656 | hypothetical protein | 51447 | 4.86 | 24 | 268 | Frankia-8 | F201371.dat | 1333 | 59 | 20 | 20 | 36 | 27 | 26 | 27 | 24 | 31 | 0.094% | 20 | 24 | 36 | 27 | 26 | 0.087% | -1.01 | -0.01 | 4.5E-01 | Red |
| WP_011607551.1 | FRAAL6510 | lipase | 48932 | 7.08 | 16 | 123 | Frankia-3 | F201366.dat | 464 | 29 | 12 | 12 | 16 | 12 | 10 | 16 | 10 | 14 | 0.045% | 10 | 12 | 16 | 12 | 11 | 0.042% | -1.02 | -0.02 | 4.5E-01 | Red |
| WP_011601888.1 | FRAAL0639 | plasmid partitioning protein | 23921 | 9.18 | 10 | 119 | Frankia-4 | F201367.dat | 427 | 36 | 8 | 8 | 13 | 13 | 12 | 12 | 13 | 10 | 0.090% | 12 | 11 | 10 | 13 | 13 | 0.083% | -1.02 | -0.02 | 4.0E-01 | Red |
| WP_011602497.1 | FRAAL1298 | ATPase AAA | 34672 | 6.10 | 12 | 117 | Frankia-4 | F201367.dat | 464 | 40 | 11 | 11 | 15 | 8 | 11 | 12 | 15 | 13 | 0.061% | 7 | 18 | 12 | 9 | 12 | 0.056% | -1.02 | -0.02 | 4.6E-01 | Red |
| WP_041938588.1 | FRAAL0008 | DNA gyrase subunit B | 70974 | 6.32 | 36 | 458 | Frankia-8 | F201371.dat | 1445 | 50 | 29 | 29 | 50 | 51 | 46 | 47 | 46 | 41 | 0.116% | 45 | 43 | 50 | 49 | 40 | 0.108% | -1.02 | -0.02 | 3.8E-01 | Red |
| WP_041939759.1 | FRAAL5780 | 30S ribosomal protein S2 | 31792 | 5.37 | 34 | 759 | Frankia-4 | F201367.dat | 1733 | 87 | 30 | 30 | 80 | 72 | 74 | 77 | 80 | 80 | 0.430% | 68 | 80 | 70 | 78 | 80 | 0.398% | -1.02 | -0.02 | 3.3E-01 | Red |
| WP_041938947.1 | FRAAL1583 | serine/threonine protein kinase | 79479 | 5.03 | 16 | 97 | Frankia-8 | F201371.dat | 549 | 21 | 11 | 10 | 15 | 12 | 7 | 12 | 10 | 8 | 0.022% | 8 | 8 | 15 | 6 | 11 | 0.020% | -1.02 | -0.02 | 4.6E-01 | Red |
| WP_050997046.1 | FRAAL1882 | heat-shock protein Hsp20 | 21929 | 6.77 | 8 | 95 | Frankia-9 | F201372.dat | 264 | 44 | 7 | 7 | 12 | 8 | 9 | 10 | 10 | 11 | 0.078% | 9 | 9 | 8 | 12 | 9 | 0.072% | -1.02 | -0.02 | 4.1E-01 | Red |
| WP_041940979.1 | FRAAL5689 | Xaa-Pro aminopeptidase | 50908 | 5.07 | 14 | 95 | Frankia-2 | F201365.dat | 447 | 30 | 12 | 12 | 12 | 11 | 12 | 8 | 8 | 9 | 0.034% | 9 | 8 | 10 | 9 | 11 | 0.031% | -1.02 | -0.02 | 4.2E-01 | Red |
| WP_041941148.1 | FRAAL6734 | nucleoside phosphorylase | 32628 | 6.45 | 13 | 93 | Frankia-2 | F201365.dat | 453 | 47 | 11 | 11 | 12 | 9 | 12 | 7 | 10 | 9 | 0.051% | 10 | 9 | 10 | 9 | 8 | 0.047% | -1.02 | -0.02 | 4.1E-01 | Red |
| WP_011607689.1 | FRAAL6652 | epimerase | 43758 | 10.35 | 21 | 182 | Frankia-4 | F201367.dat | 819 | 45 | 17 | 17 | 20 | 20 | 18 | 18 | 20 | 16 | 0.075% | 17 | 19 | 16 | 18 | 20 | 0.069% | -1.02 | -0.02 | 3.5E-01 | Red |
| WP_041940874.1 | FRAAL5111 | cytochrome C | 31291 | 8.79 | 8 | 182 | Frankia-7 | F201370.dat | 549 | 34 | 8 | 8 | 19 | 18 | 18 | 22 | 17 | 17 | 0.105% | 17 | 19 | 20 | 16 | 18 | 0.097% | -1.02 | -0.02 | 3.7E-01 | Red |
| WP_011607625.1 | FRAAL6588 | cell division protein FtsH | 79700 | 6.00 | 22 | 180 | Frankia-8 | F201371.dat | 909 | 29 | 16 | 16 | 21 | 17 | 18 | 18 | 19 | 19 | 0.041% | 16 | 18 | 21 | 17 | 17 | 0.038% | -1.02 | -0.02 | 3.4E-01 | Red |
| WP_041939648.1 | FRAAL5149 | aminopeptidase A | 52109 | 4.71 | 20 | 362 | Frankia-4 | F201367.dat | 1256 | 47 | 18 | 18 | 37 | 39 | 37 | 34 | 37 | 36 | 0.125% | 33 | 36 | 36 | 35 | 39 | 0.116% | -1.02 | -0.02 | 2.7E-01 | Red |
| WP_041938817.1 | FRAAL0943 | thiosulfate sulfurtransferase | 30874 | 4.93 | 19 | 263 | Frankia-8 | F201371.dat | 710 | 72 | 16 | 16 | 32 | 26 | 25 | 30 | 26 | 26 | 0.154% | 21 | 24 | 32 | 24 | 29 | 0.142% | -1.02 | -0.02 | 3.9E-01 | Red |
| WP_011607081.1 | FRAAL6029 | signal protein PDZ | 35483 | 8.90 | 10 | 81 | Frankia-9 | F201372.dat | 311 | 36 | 9 | 9 | 10 | 8 | 7 | 10 | 8 | 8 | 0.041% | 7 | 7 | 8 | 10 | 8 | 0.038% | -1.02 | -0.02 | 4.0E-01 | Red |
| WP_011606305.1 | FRAAL5211 | phenylalanyl-tRNA synthetase subunit alpha | 40009 | 5.46 | 18 | 168 | Frankia-10 | F201373.dat | 625 | 46 | 14 | 14 | 18 | 17 | 13 | 15 | 19 | 21 | 0.076% | 16 | 16 | 17 | 16 | 18 | 0.070% | -1.02 | -0.02 | 4.0E-01 | Red |
| WP_011606217.1 | FRAAL5109 | menaquinol-cytochrome C reductase cytochrome b subunit | 58655 | 9.20 | 22 | 334 | Frankia-3 | F201366.dat | 1015 | 33 | 20 | 20 | 38 | 40 | 33 | 38 | 32 | 26 | 0.103% | 38 | 34 | 31 | 36 | 26 | 0.095% | -1.02 | -0.02 | 4.1E-01 | Red |
| WP_011603339.1 | FRAAL2174 | SARP family transcriptional regulator | 142457 | 5.78 | 14 | 73 | Frankia-9 | F201372.dat | 393 | 9 | 9 | 9 | 9 | 8 | 5 | 8 | 7 | 9 | 0.009% | 8 | 3 | 8 | 9 | 8 | 0.009% | -1.02 | -0.02 | 4.4E-01 | Red |
| WP_011602294.1 | FRAAL1072 | DNA-directed RNA polymerase subunit beta | 126625 | 4.98 | 93 | 1608 | Frankia-3 | F201366.dat | 4154 | 71 | 79 | 79 | 161 | 170 | 176 | 161 | 149 | 158 | 0.229% | 170 | 134 | 167 | 163 | 160 | 0.211% | -1.03 | -0.02 | 3.1E-01 | Red |
| WP_011606910.1 | FRAAL5843 | 3-isopropylmalate dehydrogenase | 36181 | 5.46 | 19 | 231 | Frankia-7 | F201370.dat | 884 | 47 | 15 | 15 | 25 | 22 | 20 | 24 | 26 | 25 | 0.115% | 23 | 25 | 22 | 21 | 23 | 0.106% | -1.03 | -0.02 | 3.2E-01 | Red |
| WP_009742786.1 | FRAAL5244 | elongation factor P | 20115 | 4.91 | 6 | 69 | Frankia-4 | F201367.dat | 282 | 32 | 6 | 6 | 9 | 6 | 6 | 6 | 9 | 8 | 0.062% | 7 | 5 | 6 | 9 | 7 | 0.057% | -1.03 | -0.03 | 4.2E-01 | Red |
| WP_041939023.1 | FRAAL1932 | ATPase | 32517 | 4.88 | 14 | 144 | Frankia-4 | F201367.dat | 610 | 37 | 12 | 12 | 15 | 15 | 14 | 14 | 15 | 15 | 0.080% | 14 | 16 | 17 | 12 | 12 | 0.074% | -1.03 | -0.03 | 3.6E-01 | Red |
| WP_041939784.1 | FRAAL5931 | ATP synthase subunit beta | 52413 | 4.71 | 38 | 1131 | Frankia-5 | F201368.dat | 2393 | 86 | 34 | 34 | 119 | 114 | 112 | 112 | 116 | 119 | 0.390% | 112 | 125 | 105 | 111 | 105 | 0.359% | -1.03 | -0.03 | 2.3E-01 | Red |
| WP_011607343.1 | FRAAL6295 | glycosyl hydrolase family 15 | 66580 | 5.33 | 18 | 142 | Frankia-8 | F201371.dat | 805 | 35 | 15 | 15 | 17 | 16 | 16 | 16 | 14 | 10 | 0.039% | 11 | 16 | 17 | 12 | 14 | 0.035% | -1.03 | -0.03 | 4.1E-01 | Red |
| WP_011603386.1 | FRAAL2217 | hypothetical protein | 77470 | 6.22 | 26 | 217 | Frankia-8 | F201371.dat | 1113 | 47 | 22 | 22 | 27 | 23 | 20 | 22 | 25 | 20 | 0.051% | 15 | 20 | 27 | 20 | 25 | 0.047% | -1.03 | -0.03 | 4.0E-01 | Red |
| WP_011606056.1 | FRAAL4942 | phosphate ABC transporter substrate-binding protein | 89154 | 6.17 | 15 | 65 | Frankia-5 | F201368.dat | 519 | 15 | 8 | 8 | 8 | 9 | 3 | 4 | 9 | 8 | 0.013% | 6 | 7 | 9 | 5 | 5 | 0.012% | -1.03 | -0.03 | 4.5E-01 | Red |
| WP_011602267.1 | FRAAL1040 | NADH-quinone oxidoreductase subunit I | 24491 | 5.10 | 9 | 63 | Frankia-8 | F201371.dat | 361 | 53 | 8 | 8 | 11 | 6 | 7 | 5 | 7 | 7 | 0.047% | 4 | 6 | 11 | 5 | 5 | 0.043% | -1.03 | -0.03 | 4.4E-01 | Red |
| WP_041940213.1 | FRAAL1352 | peptide chain release factor 2 | 40367 | 4.78 | 12 | 63 | Frankia-8 | F201371.dat | 338 | 34 | 10 | 10 | 11 | 6 | 7 | 7 | 6 | 6 | 0.028% | 4 | 6 | 11 | 2 | 8 | 0.026% | -1.03 | -0.03 | 4.5E-01 | Red |
| WP_041939484.1 | FRAAL4337 | superoxide dismutase | 22141 | 5.01 | 8 | 205 | Frankia-8 | F201371.dat | 504 | 51 | 7 | 7 | 21 | 17 | 20 | 21 | 22 | 24 | 0.168% | 18 | 20 | 21 | 19 | 23 | 0.154% | -1.03 | -0.03 | 3.4E-01 | Red |
| WP_050997182.1 | FRAAL4400 | hypothetical protein | 47058 | 6.04 | 12 | 61 | Frankia-3 | F201366.dat | 241 | 18 | 8 | 8 | 8 | 8 | 5 | 8 | 4 | 6 | 0.024% | 7 | 7 | 5 | 7 | 4 | 0.021% | -1.03 | -0.03 | 4.2E-01 | Red |
| WP_041939845.1 | FRAAL6201 | methionine sulfoxide reductase A | 24149 | 4.84 | 6 | 59 | Frankia-4 | F201367.dat | 329 | 25 | 6 | 6 | 9 | 6 | 5 | 6 | 9 | 4 | 0.044% | 5 | 5 | 6 | 7 | 6 | 0.040% | -1.03 | -0.03 | 4.2E-01 | Red |
| WP_011607034.1 | FRAAL5978 | acetamidase | 52491 | 5.64 | 7 | 59 | Frankia-1 | F201364.dat | 342 | 17 | 6 | 6 | 8 | 8 | 6 | 6 | 6 | 4 | 0.020% | 5 | 7 | 5 | 8 | 4 | 0.019% | -1.03 | -0.03 | 4.2E-01 | Red |
| WP_009740212.1 | FRAAL1435 | hopanoid biosynthesis associated radical SAM protein HpnH | 37814 | 8.40 | 22 | 331 | Frankia-8 | F201371.dat | 1120 | 59 | 17 | 17 | 38 | 32 | 35 | 35 | 34 | 32 | 0.159% | 31 | 32 | 38 | 32 | 30 | 0.145% | -1.03 | -0.03 | 2.7E-01 | Red |
| WP_011601507.1 | FRAAL0249 | hypothetical protein | 157416 | 5.87 | 26 | 122 | Frankia-6 | F201369.dat | 980 | 18 | 18 | 18 | 19 | 15 | 12 | 10 | 12 | 13 | 0.014% | 19 | 9 | 16 | 10 | 6 | 0.013% | -1.03 | -0.03 | 4.4E-01 | Red |
| WP_011606114.1 | FRAAL5002 | imidazoleglycerol-phosphate dehydratase | 20891 | 6.03 | 5 | 55 | Frankia-9 | F201372.dat | 242 | 24 | 5 | 5 | 6 | 6 | 5 | 4 | 6 | 7 | 0.048% | 4 | 5 | 6 | 6 | 6 | 0.044% | -1.03 | -0.03 | 3.8E-01 | Red |
| WP_011603632.1 | FRAAL2475 | hemin receptor | 12077 | 4.77 | 3 | 55 | Frankia-1 | F201364.dat | 187 | 24 | 3 | 3 | 6 | 6 | 5 | 7 | 6 | 4 | 0.083% | 5 | 5 | 4 | 7 | 6 | 0.075% | -1.03 | -0.03 | 3.9E-01 | Red |
| WP_041939316.1 | FRAAL3254 | hypothetical protein | 20575 | 4.83 | 7 | 116 | Frankia-5 | F201368.dat | 466 | 58 | 7 | 7 | 12 | 12 | 12 | 11 | 12 | 12 | 0.102% | 12 | 10 | 11 | 12 | 12 | 0.093% | -1.03 | -0.03 | 2.3E-01 | Red |
| WP_011607895.1 | FRAAL6860 | inositol-3-phosphate synthase | 38832 | 5.07 | 20 | 242 | Frankia-8 | F201371.dat | 1085 | 55 | 17 | 17 | 29 | 25 | 23 | 25 | 24 | 26 | 0.113% | 21 | 26 | 29 | 20 | 23 | 0.103% | -1.03 | -0.03 | 3.3E-01 | Red |
| WP_011603165.1 | FRAAL1993 | protein-tyrosine phosphatase | 27360 | 5.82 | 14 | 116 | Frankia-8 | F201371.dat | 537 | 49 | 11 | 11 | 18 | 11 | 12 | 11 | 11 | 14 | 0.077% | 6 | 11 | 18 | 10 | 12 | 0.070% | -1.03 | -0.03 | 4.2E-01 | Red |
| WP_041938678.1 | FRAAL0398 | NADH pyrophosphatase | 35816 | 4.82 | 6 | 53 | Frankia-10 | F201373.dat | 371 | 26 | 6 | 6 | 7 | 6 | 3 | 6 | 7 | 5 | 0.027% | 3 | 4 | 8 | 4 | 7 | 0.024% | -1.03 | -0.03 | 4.3E-01 | Red |
| WP_041938772.1 | FRAAL0792 | branched-chain amino acid ABC transporter substrate-binding protein | 42207 | 7.62 | 17 | 175 | Frankia-3 | F201366.dat | 744 | 53 | 14 | 14 | 21 | 18 | 17 | 21 | 18 | 15 | 0.075% | 17 | 16 | 19 | 18 | 16 | 0.069% | -1.03 | -0.03 | 3.1E-01 | Red |
| WP_011606299.1 | FRAAL5205 | acetylornithine aminotransferase | 41599 | 5.56 | 18 | 173 | Frankia-5 | F201368.dat | 821 | 61 | 15 | 15 | 21 | 16 | 18 | 15 | 18 | 21 | 0.075% | 14 | 15 | 23 | 14 | 19 | 0.069% | -1.03 | -0.03 | 3.9E-01 | Red |
| WP_011603185.1 | FRAAL2013 | GTPase Era | 33776 | 9.00 | 9 | 51 | Frankia-3 | F201366.dat | 306 | 28 | 6 | 6 | 6 | 3 | 4 | 6 | 6 | 7 | 0.027% | 6 | 5 | 6 | 3 | 5 | 0.025% | -1.03 | -0.03 | 4.2E-01 | Red |
| WP_011606322.1 | FRAAL5227 | S-adenosylmethionine synthase | 42504 | 4.95 | 29 | 645 | Frankia-5 | F201368.dat | 1686 | 62 | 27 | 27 | 72 | 67 | 58 | 67 | 64 | 72 | 0.275% | 55 | 63 | 77 | 59 | 63 | 0.251% | -1.03 | -0.03 | 3.1E-01 | Red |
| WP_041939236.1 | FRAAL2869 | proteasome-associated ATPase | 66449 | 5.01 | 54 | 641 | Frankia-6 | F201369.dat | 2134 | 69 | 41 | 41 | 69 | 70 | 57 | 71 | 68 | 60 | 0.175% | 69 | 62 | 62 | 63 | 59 | 0.160% | -1.03 | -0.03 | 2.6E-01 | Red |
| WP_011605667.1 | FRAAL4549 | dTDP-glucose 4.6-dehydratase | 37328 | 5.23 | 13 | 106 | Frankia-8 | F201371.dat | 484 | 37 | 11 | 11 | 12 | 10 | 10 | 14 | 11 | 9 | 0.052% | 9 | 12 | 12 | 10 | 9 | 0.047% | -1.04 | -0.03 | 3.6E-01 | Red |
| WP_050997449.1 | FRAAL5886 | acetyl-CoA synthetase | 72374 | 5.26 | 39 | 1085 | Frankia-4 | F201367.dat | 2287 | 56 | 37 | 37 | 108 | 116 | 111 | 104 | 108 | 113 | 0.272% | 100 | 96 | 123 | 102 | 112 | 0.248% | -1.04 | -0.03 | 2.5E-01 | Red |
| WP_011607189.1 | FRAAL6139 | GTP-binding protein | 38160 | 4.68 | 13 | 104 | Frankia-5 | F201368.dat | 596 | 35 | 10 | 10 | 13 | 11 | 9 | 9 | 11 | 13 | 0.050% | 10 | 10 | 11 | 9 | 11 | 0.045% | -1.04 | -0.04 | 3.2E-01 | Red |
| WP_041939970.1 | FRAAL6751 | serine/threonine protein phosphatase | 50190 | 4.98 | 17 | 161 | Frankia-2 | F201365.dat | 759 | 36 | 14 | 14 | 18 | 16 | 18 | 15 | 18 | 15 | 0.058% | 11 | 17 | 17 | 14 | 20 | 0.053% | -1.04 | -0.04 | 3.6E-01 | Red |
| WP_011602403.1 | FRAAL1200 | purine nucleoside phosphorylase | 28020 | 5.99 | 8 | 47 | Frankia-8 | F201371.dat | 438 | 41 | 8 | 8 | 10 | 5 | 3 | 5 | 6 | 5 | 0.031% | 2 | 2 | 10 | 3 | 6 | 0.028% | -1.04 | -0.04 | 4.5E-01 | Red |
| WP_011604316.1 | FRAAL3167 | catalase | 58051 | 5.82 | 32 | 497 | Frankia-5 | F201368.dat | 1278 | 63 | 27 | 27 | 51 | 54 | 48 | 50 | 50 | 51 | 0.156% | 48 | 50 | 49 | 44 | 53 | 0.142% | -1.04 | -0.04 | 1.7E-01 | Red |
| WP_011602673.1 | FRAAL1483 | ABC transporter | 24202 | 8.16 | 9 | 45 | Frankia-2 | F201365.dat | 249 | 46 | 6 | 6 | 7 | 4 | 7 | 4 | 4 | 4 | 0.034% | 4 | 5 | 4 | 5 | 4 | 0.031% | -1.04 | -0.04 | 3.9E-01 | Red |
| WP_011606319.1 | FRAAL5224 | methyltransferase | 51460 | 8.55 | 8 | 45 | Frankia-10 | F201373.dat | 246 | 11 | 6 | 6 | 6 | 5 | 5 | 6 | 3 | 4 | 0.016% | 5 | 3 | 4 | 4 | 6 | 0.014% | -1.04 | -0.04 | 3.9E-01 | Red |
| WP_041940002.1 | FRAAL6874 | N-acetylmuramoyl-L-alanine amidase | 45531 | 6.04 | 7 | 45 | Frankia-4 | F201367.dat | 251 | 24 | 7 | 7 | 7 | 6 | 3 | 2 | 7 | 5 | 0.018% | 4 | 4 | 4 | 5 | 5 | 0.016% | -1.04 | -0.04 | 4.2E-01 | Red |
| WP_011606812.1 | FRAAL5738 | 4-hydroxy-tetrahydrodipicolinate synthase | 33973 | 5.43 | 12 | 98 | Frankia-5 | F201368.dat | 530 | 53 | 10 | 10 | 11 | 10 | 8 | 9 | 12 | 11 | 0.053% | 9 | 8 | 14 | 8 | 9 | 0.048% | -1.04 | -0.04 | 3.9E-01 | Red |
| WP_011602193.1 | FRAAL0964 | 2.3-bisphosphoglycerate-dependent phosphoglycerate mutase | 26825 | 5.18 | 18 | 308 | Frankia-1 | F201364.dat | 768 | 64 | 14 | 14 | 31 | 31 | 32 | 31 | 31 | 32 | 0.209% | 26 | 30 | 35 | 30 | 30 | 0.190% | -1.04 | -0.04 | 2.2E-01 | Red |
| WP_011606850.1 | FRAAL5778 | ribosome-recycling factor | 20742 | 5.37 | 10 | 96 | Frankia-9 | F201372.dat | 378 | 41 | 7 | 7 | 11 | 10 | 10 | 9 | 11 | 9 | 0.084% | 9 | 9 | 10 | 11 | 8 | 0.076% | -1.04 | -0.04 | 2.7E-01 | Red |
| WP_011605641.1 | FRAAL4522 | enoyl-ACP reductase | 26818 | 5.24 | 9 | 149 | Frankia-8 | F201371.dat | 501 | 37 | 8 | 8 | 16 | 13 | 17 | 15 | 14 | 17 | 0.101% | 13 | 15 | 16 | 15 | 14 | 0.092% | -1.04 | -0.04 | 2.7E-01 | Red |
| WP_011602726.1 | FRAAL1536 | hypothetical protein | 22967 | 4.53 | 7 | 43 | Frankia-4 | F201367.dat | 265 | 42 | 5 | 5 | 6 | 5 | 3 | 5 | 6 | 3 | 0.034% | 3 | 4 | 6 | 4 | 4 | 0.031% | -1.04 | -0.04 | 4.0E-01 | Red |
| WP_041939578.1 | FRAAL4847 | NADPH:quinone reductase | 31034 | 4.75 | 5 | 43 | Frankia-4 | F201367.dat | 208 | 14 | 3 | 3 | 6 | 4 | 3 | 4 | 6 | 5 | 0.025% | 5 | 2 | 5 | 5 | 4 | 0.023% | -1.04 | -0.04 | 4.0E-01 | Red |
| WP_041940780.1 | FRAAL4576 | glucose-6-phosphate dehydrogenase | 56642 | 5.26 | 37 | 461 | Frankia-1 | F201364.dat | 1688 | 70 | 34 | 34 | 52 | 52 | 46 | 50 | 45 | 42 | 0.148% | 38 | 50 | 47 | 47 | 44 | 0.134% | -1.04 | -0.04 | 2.6E-01 | Red |
| WP_011603389.1 | FRAAL2220 | hypothetical protein | 27048 | 5.04 | 14 | 94 | Frankia-4 | F201367.dat | 637 | 60 | 11 | 11 | 11 | 10 | 10 | 9 | 11 | 8 | 0.063% | 9 | 8 | 9 | 10 | 10 | 0.057% | -1.04 | -0.04 | 2.7E-01 | Red |
| WP_011604801.1 | FRAAL3662 | PPOX class F420-dependent enzyme | 15846 | 6.41 | 7 | 94 | Frankia-3 | F201366.dat | 443 | 81 | 7 | 7 | 11 | 8 | 10 | 11 | 10 | 9 | 0.108% | 11 | 9 | 7 | 11 | 8 | 0.098% | -1.04 | -0.04 | 3.4E-01 | Red |
| WP_041939239.1 | FRAAL2878 | Pup--protein ligase | 51984 | 8.72 | 34 | 400 | Frankia-8 | F201371.dat | 1301 | 61 | 27 | 27 | 41 | 42 | 41 | 38 | 44 | 39 | 0.140% | 38 | 39 | 41 | 40 | 38 | 0.127% | -1.04 | -0.04 | 1.1E-01 | Red |
| WP_011607790.1 | FRAAL6754 | serine/threonine protein kinase | 57717 | 8.83 | 7 | 41 | Frankia-4 | F201367.dat | 176 | 14 | 5 | 5 | 5 | 5 | 3 | 3 | 5 | 5 | 0.013% | 3 | 4 | 5 | 3 | 5 | 0.012% | -1.04 | -0.04 | 3.9E-01 | Red |
| WP_011602012.1 | FRAAL0773 | acyl-CoA dehydrogenase | 62138 | 7.78 | 7 | 41 | Frankia-8 | F201371.dat | 264 | 11 | 5 | 5 | 6 | 5 | 4 | 5 | 4 | 3 | 0.012% | 3 | 3 | 6 | 5 | 3 | 0.011% | -1.04 | -0.04 | 4.0E-01 | Red |
| WP_011604673.1 | FRAAL3532 | branched-chain amino acid ABC transporter substrate-binding protein | 43436 | 9.33 | 14 | 92 | Frankia-8 | F201371.dat | 644 | 38 | 12 | 12 | 14 | 12 | 8 | 8 | 11 | 8 | 0.039% | 6 | 9 | 14 | 8 | 8 | 0.035% | -1.04 | -0.04 | 4.0E-01 | Red |
| WP_050997289.1 | FRAAL6524 | 2-C-methyl-D-erythritol 4-phosphate cytidylyltransferase | 27007 | 9.02 | 5 | 41 | Frankia-5 | F201368.dat | 182 | 27 | 5 | 5 | 6 | 4 | 5 | 2 | 4 | 6 | 0.028% | 5 | 3 | 5 | 3 | 4 | 0.025% | -1.04 | -0.04 | 4.0E-01 | Red |
| WP_011607757.1 | FRAAL6720 | preprotein translocase SecA | 31793 | 4.76 | 5 | 41 | Frankia-3 | F201366.dat | 200 | 25 | 5 | 5 | 7 | 5 | 3 | 7 | 4 | 2 | 0.024% | 4 | 4 | 5 | 3 | 4 | 0.021% | -1.04 | -0.04 | 4.2E-01 | Red |
| WP_009740501.1 | FRAAL1110 | DNA-directed RNA polymerase subunit alpha | 37807 | 4.58 | 36 | 739 | Frankia-2 | F201365.dat | 1789 | 83 | 34 | 34 | 75 | 78 | 75 | 78 | 71 | 75 | 0.356% | 75 | 67 | 80 | 70 | 70 | 0.322% | -1.04 | -0.04 | 1.4E-01 | Red |
| WP_011602287.1 | FRAAL1061 | MaoC family dehydratase | 14980 | 4.95 | 5 | 39 | Frankia-2 | F201365.dat | 365 | 66 | 5 | 5 | 5 | 4 | 5 | 3 | 4 | 4 | 0.048% | 3 | 5 | 4 | 4 | 3 | 0.043% | -1.04 | -0.04 | 3.5E-01 | Red |
| WP_011605752.1 | FRAAL4636 | secreted FAD-linked oxidase | 58615 | 6.46 | 26 | 231 | Frankia-3 | F201366.dat | 1066 | 55 | 18 | 18 | 28 | 22 | 19 | 28 | 24 | 25 | 0.072% | 22 | 28 | 15 | 27 | 21 | 0.065% | -1.04 | -0.04 | 3.6E-01 | Red |
| WP_009740529.1 | FRAAL1079 | elongation factor Tu | 43948 | 5.41 | 41 | 1955 | Frankia-5 | F201368.dat | 2541 | 96 | 40 | 40 | 219 | 197 | 185 | 192 | 205 | 219 | 0.810% | 180 | 193 | 209 | 182 | 193 | 0.733% | -1.04 | -0.04 | 1.6E-01 | Red |
| WP_041939015.1 | FRAAL1901 | glycosyl transferase family 1 | 45611 | 9.50 | 20 | 274 | Frankia-9 | F201372.dat | 843 | 42 | 18 | 18 | 28 | 28 | 25 | 30 | 29 | 28 | 0.110% | 22 | 26 | 30 | 28 | 28 | 0.099% | -1.04 | -0.04 | 2.4E-01 | Red |
| WP_011604676.1 | FRAAL3535 | RCC1 repeat- and reductase domain-containing protein | 42131 | 4.83 | 4 | 37 | Frankia-7 | F201370.dat | 339 | 16 | 4 | 4 | 5 | 4 | 5 | 3 | 4 | 3 | 0.016% | 4 | 5 | 2 | 4 | 3 | 0.014% | -1.04 | -0.04 | 3.8E-01 | Red |
| WP_050997431.1 | FRAAL5054 | PA-phosphatase | 54929 | 10.99 | 8 | 37 | Frankia-8 | F201371.dat | 219 | 11 | 5 | 5 | 5 | 4 | 4 | 4 | 4 | 3 | 0.012% | 3 | 2 | 5 | 5 | 3 | 0.011% | -1.04 | -0.04 | 3.9E-01 | Red |
| WP_011604693.1 | FRAAL3552 | aldehyde dehydrogenase | 52321 | 4.92 | 18 | 176 | Frankia-1 | F201364.dat | 901 | 51 | 16 | 15 | 21 | 21 | 16 | 16 | 17 | 20 | 0.061% | 15 | 17 | 18 | 17 | 19 | 0.055% | -1.04 | -0.04 | 2.7E-01 | Red |
| WP_011602504.1 | FRAAL1306 | protein translocase subunit SecA | 108713 | 5.01 | 51 | 446 | Frankia-8 | F201371.dat | 2036 | 45 | 41 | 41 | 63 | 52 | 44 | 49 | 39 | 44 | 0.075% | 37 | 35 | 63 | 39 | 44 | 0.068% | -1.04 | -0.04 | 3.6E-01 | Red |
| WP_011606732.1 | FRAAL5655 | succinate dehydrogenase | 26255 | 5.02 | 12 | 309 | Frankia-8 | F201371.dat | 801 | 65 | 11 | 11 | 31 | 27 | 30 | 31 | 33 | 37 | 0.215% | 28 | 31 | 31 | 30 | 31 | 0.194% | -1.04 | -0.04 | 2.2E-01 | Red |
| WP_011606338.1 | FRAAL5247 | chorismate synthase | 41136 | 6.28 | 17 | 172 | Frankia-5 | F201368.dat | 731 | 43 | 14 | 14 | 16 | 17 | 16 | 21 | 18 | 16 | 0.076% | 19 | 16 | 16 | 18 | 15 | 0.069% | -1.04 | -0.04 | 2.6E-01 | Red |
| WP_011606606.1 | FRAAL5522 | (3S)-malyl-CoA thiolesterase | 29421 | 5.91 | 17 | 125 | Frankia-5 | F201368.dat | 676 | 64 | 14 | 14 | 16 | 13 | 13 | 11 | 11 | 16 | 0.078% | 10 | 11 | 16 | 10 | 14 | 0.070% | -1.05 | -0.04 | 3.5E-01 | Red |
| WP_011607265.1 | FRAAL6215 | hypothetical protein | 72877 | 5.27 | 7 | 35 | Frankia-3 | F201366.dat | 311 | 13 | 6 | 6 | 6 | 2 | 4 | 6 | 3 | 3 | 0.009% | 2 | 6 | 4 | 3 | 2 | 0.008% | -1.05 | -0.04 | 4.2E-01 | Red |
| WP_041939749.1 | FRAAL5692 | iron ABC transporter ATP-binding protein | 29290 | 7.86 | 16 | 168 | Frankia-4 | F201367.dat | 789 | 55 | 15 | 15 | 19 | 14 | 16 | 20 | 19 | 17 | 0.105% | 15 | 17 | 17 | 17 | 16 | 0.094% | -1.05 | -0.04 | 2.5E-01 | Red |
| WP_041938830.1 | FRAAL0988 | heavy metal transporter | 7225 | 4.47 | 6 | 78 | Frankia-9 | F201372.dat | 472 | 99 | 6 | 6 | 13 | 9 | 9 | 8 | 8 | 6 | 0.198% | 8 | 6 | 4 | 13 | 7 | 0.177% | -1.05 | -0.05 | 4.0E-01 | Red |
| WP_041939394.1 | FRAAL3801 | hypothetical protein | 32912 | 5.65 | 6 | 76 | Frankia-8 | F201371.dat | 332 | 28 | 6 | 6 | 9 | 7 | 7 | 8 | 8 | 9 | 0.042% | 6 | 6 | 9 | 9 | 7 | 0.038% | -1.05 | -0.05 | 3.1E-01 | Red |
| WP_041938931.1 | FRAAL1504 | MBL fold metallo-hydrolase | 26821 | 4.89 | 7 | 76 | Frankia-10 | F201373.dat | 392 | 32 | 6 | 6 | 9 | 6 | 7 | 10 | 8 | 8 | 0.052% | 6 | 7 | 9 | 6 | 9 | 0.046% | -1.05 | -0.05 | 3.4E-01 | Red |
| WP_011606416.1 | FRAAL5329 | aminoglycoside phosphotransferase | 42270 | 5.50 | 6 | 33 | Frankia-9 | F201372.dat | 179 | 10 | 4 | 4 | 4 | 4 | 3 | 3 | 4 | 3 | 0.014% | 3 | 4 | 2 | 4 | 3 | 0.013% | -1.05 | -0.05 | 3.5E-01 | Red |
| WP_011607839.1 | FRAAL6805 | nitrogenase-stabilizing/protective protein NifW | 14545 | 5.64 | 4 | 33 | Frankia-4 | F201367.dat | 260 | 40 | 4 | 4 | 5 | 3 | 3 | 3 | 5 | 3 | 0.042% | 3 | 4 | 2 | 4 | 3 | 0.037% | -1.05 | -0.05 | 3.6E-01 | Red |
| WP_011601991.1 | FRAAL0751 | type 11 methyltransferase | 33091 | 4.56 | 4 | 33 | Frankia-4 | F201367.dat | 267 | 23 | 4 | 4 | 4 | 4 | 3 | 2 | 4 | 4 | 0.018% | 4 | 3 | 4 | 1 | 4 | 0.016% | -1.05 | -0.05 | 3.9E-01 | Red |
| WP_011607102.1 | FRAAL6050 | molybdopterin biosynthesis protein MoeZ | 41922 | 4.95 | 18 | 203 | Frankia-3 | F201366.dat | 751 | 53 | 16 | 16 | 26 | 20 | 18 | 26 | 22 | 18 | 0.089% | 18 | 20 | 24 | 19 | 18 | 0.080% | -1.05 | -0.05 | 3.0E-01 | Red |
| WP_011603179.1 | FRAAL2007 | molecular chaperone DnaJ | 39931 | 7.62 | 19 | 154 | Frankia-10 | F201373.dat | 741 | 45 | 14 | 14 | 17 | 16 | 14 | 15 | 15 | 19 | 0.071% | 11 | 18 | 16 | 13 | 17 | 0.063% | -1.05 | -0.05 | 3.1E-01 | Red |
| WP_041939966.1 | FRAAL6730 | phosphoribosylformylglycinamidine synthase | 80394 | 4.72 | 24 | 193 | Frankia-10 | F201373.dat | 1029 | 32 | 17 | 17 | 21 | 23 | 17 | 20 | 21 | 18 | 0.044% | 18 | 18 | 21 | 16 | 21 | 0.039% | -1.05 | -0.05 | 2.5E-01 | Red |
| WP_011602365.1 | FRAAL1162 | purine biosynthesis protein purH | 57184 | 5.33 | 24 | 312 | Frankia-8 | F201371.dat | 1364 | 49 | 21 | 21 | 41 | 37 | 29 | 29 | 32 | 33 | 0.100% | 24 | 29 | 41 | 24 | 34 | 0.090% | -1.05 | -0.05 | 3.3E-01 | Red |
| WP_011604000.1 | FRAAL2854 | phosphoglycerate mutase | 28209 | 5.18 | 6 | 70 | Frankia-9 | F201372.dat | 325 | 29 | 6 | 6 | 9 | 7 | 7 | 8 | 7 | 7 | 0.046% | 7 | 7 | 7 | 9 | 4 | 0.041% | -1.05 | -0.05 | 3.3E-01 | Red |
| WP_011601277.1 | FRAAL0009 | DNA gyrase subunit A | 92041 | 5.60 | 63 | 893 | Frankia-2 | F201365.dat | 2681 | 65 | 52 | 52 | 96 | 103 | 96 | 93 | 81 | 85 | 0.178% | 92 | 90 | 91 | 82 | 80 | 0.159% | -1.05 | -0.05 | 1.8E-01 | Red |
| WP_011602352.1 | FRAAL1148 | histidine kinase | 143019 | 5.47 | 23 | 146 | Frankia-1 | F201364.dat | 770 | 15 | 17 | 17 | 18 | 18 | 16 | 14 | 13 | 14 | 0.019% | 13 | 11 | 16 | 13 | 18 | 0.017% | -1.05 | -0.05 | 3.1E-01 | Red |
| WP_041940121.1 | FRAAL0737 | ATP-dependent DNA helicase RecQ | 59358 | 5.60 | 5 | 29 | Frankia-5 | F201368.dat | 225 | 8 | 4 | 4 | 4 | 3 | 3 | 3 | 2 | 4 | 0.009% | 3 | 3 | 2 | 2 | 4 | 0.008% | -1.05 | -0.05 | 3.5E-01 | Red |
| WP_011604694.1 | FRAAL3553 | acyl-CoA synthetase | 49851 | 5.57 | 5 | 29 | Frankia-3 | F201366.dat | 214 | 14 | 4 | 4 | 4 | 3 | 2 | 4 | 2 | 4 | 0.011% | 2 | 2 | 3 | 3 | 4 | 0.009% | -1.05 | -0.05 | 3.7E-01 | Red |
| WP_011606071.1 | FRAAL4957 | hypothetical protein | 58016 | 5.54 | 6 | 29 | Frankia-7 | F201370.dat | 229 | 13 | 4 | 4 | 4 | 3 | 3 | 3 | 3 | 3 | 0.009% | 4 | 4 | 2 | 2 | 2 | 0.008% | -1.05 | -0.05 | 3.7E-01 | Red |
| WP_041938988.1 | FRAAL1770 | hypothetical protein | 32265 | 5.06 | 6 | 29 | Frankia-8 | F201371.dat | 168 | 19 | 5 | 5 | 5 | 4 | 4 | 1 | 3 | 3 | 0.017% | 1 | 1 | 5 | 3 | 4 | 0.015% | -1.05 | -0.05 | 4.2E-01 | Red |
| WP_041938674.1 | FRAAL0381 | propionyl-CoA synthetase | 66024 | 5.34 | 13 | 105 | Frankia-4 | F201367.dat | 478 | 21 | 10 | 10 | 11 | 12 | 12 | 11 | 11 | 8 | 0.029% | 10 | 11 | 11 | 8 | 11 | 0.026% | -1.05 | -0.05 | 2.7E-01 | Red |
| WP_011603057.1 | FRAAL1880 | AMP-dependent synthetase | 99457 | 5.34 | 15 | 105 | Frankia-7 | F201370.dat | 552 | 17 | 11 | 11 | 13 | 12 | 11 | 9 | 10 | 12 | 0.019% | 12 | 13 | 9 | 11 | 6 | 0.017% | -1.05 | -0.05 | 3.4E-01 | Red |
| WP_011601540.1 | FRAAL0280 | polynucleotide kinase-phosphatase | 97028 | 6.22 | 24 | 181 | Frankia-1 | F201364.dat | 966 | 24 | 19 | 19 | 24 | 24 | 15 | 22 | 19 | 13 | 0.034% | 17 | 16 | 24 | 14 | 17 | 0.031% | -1.05 | -0.05 | 3.6E-01 | Red |
| WP_011602438.1 | FRAAL1237 | glycosyl transferase | 51619 | 8.87 | 12 | 103 | Frankia-3 | F201366.dat | 363 | 19 | 9 | 9 | 12 | 10 | 10 | 12 | 12 | 9 | 0.037% | 9 | 12 | 12 | 9 | 8 | 0.033% | -1.05 | -0.05 | 2.9E-01 | Red |
| WP_009742888.1 | FRAAL5127 | hypothetical protein | 12909 | 4.37 | 10 | 138 | Frankia-5 | F201368.dat | 602 | 95 | 9 | 9 | 13 | 15 | 15 | 11 | 17 | 13 | 0.196% | 13 | 13 | 12 | 15 | 14 | 0.175% | -1.06 | -0.05 | 2.5E-01 | Red |
| WP_050997471.1 | FRAAL6598 | peptidase | 50647 | 5.03 | 10 | 64 | Frankia-1 | F201364.dat | 332 | 17 | 7 | 7 | 9 | 9 | 6 | 4 | 7 | 7 | 0.023% | 6 | 6 | 6 | 6 | 7 | 0.021% | -1.06 | -0.05 | 3.3E-01 | Red |
| WP_011606829.1 | FRAAL5756 | tRNA pseudouridine synthase B | 31558 | 8.55 | 3 | 27 | Frankia-5 | F201368.dat | 138 | 16 | 3 | 3 | 3 | 2 | 3 | 3 | 3 | 3 | 0.016% | 2 | 2 | 3 | 3 | 3 | 0.014% | -1.06 | -0.05 | 3.4E-01 | Red |
| WP_011601572.1 | FRAAL0316 | 3-ketoacyl-ACP reductase | 30949 | 4.93 | 3 | 27 | Frankia-5 | F201368.dat | 143 | 11 | 3 | 3 | 4 | 2 | 2 | 3 | 3 | 4 | 0.016% | 2 | 2 | 4 | 2 | 3 | 0.014% | -1.06 | -0.05 | 3.6E-01 | Red |
| WP_011601755.1 | FRAAL0502 | alpha/beta hydrolase | 32250 | 5.62 | 5 | 27 | Frankia-4 | F201367.dat | 224 | 20 | 4 | 4 | 4 | 3 | 1 | 3 | 4 | 3 | 0.015% | 3 | 3 | 4 | 2 | 1 | 0.014% | -1.06 | -0.05 | 3.9E-01 | Red |
| WP_041939657.1 | FRAAL5208 | N-acetyl-gamma-glutamyl-phosphate reductase | 34364 | 5.56 | 8 | 64 | Frankia-8 | F201371.dat | 438 | 38 | 8 | 8 | 12 | 6 | 7 | 4 | 7 | 9 | 0.034% | 4 | 4 | 12 | 4 | 7 | 0.030% | -1.06 | -0.05 | 4.1E-01 | Red |
| WP_041938948.1 | FRAAL1586 | nitroreductase | 37072 | 4.88 | 22 | 278 | Frankia-1 | F201364.dat | 966 | 72 | 19 | 19 | 27 | 27 | 26 | 29 | 31 | 30 | 0.138% | 23 | 28 | 32 | 23 | 29 | 0.123% | -1.06 | -0.06 | 2.2E-01 | Red |
| WP_041940106.1 | FRAAL0644 | hypothetical protein | 41614 | 6.37 | 11 | 62 | Frankia-4 | F201367.dat | 174 | 18 | 7 | 7 | 8 | 6 | 5 | 7 | 8 | 6 | 0.027% | 4 | 6 | 7 | 6 | 7 | 0.024% | -1.06 | -0.06 | 3.0E-01 | Red |
| WP_011602990.1 | FRAAL1810 | alanine-phosphoribitol ligase | 44496 | 5.03 | 18 | 169 | Frankia-1 | F201364.dat | 809 | 54 | 15 | 15 | 19 | 19 | 15 | 18 | 19 | 16 | 0.070% | 15 | 13 | 23 | 15 | 16 | 0.062% | -1.06 | -0.06 | 3.1E-01 | Red |
| WP_041939787.1 | FRAAL5948 | transcription termination factor Rho | 84191 | 9.38 | 37 | 657 | Frankia-1 | F201364.dat | 1691 | 46 | 32 | 32 | 74 | 74 | 68 | 63 | 65 | 68 | 0.143% | 70 | 58 | 63 | 64 | 64 | 0.128% | -1.06 | -0.06 | 9.6E-02 | Red |
| WP_011606015.1 | FRAAL4901 | prephenate dehydratase | 31162 | 5.47 | 8 | 60 | Frankia-8 | F201371.dat | 356 | 26 | 6 | 6 | 6 | 6 | 7 | 7 | 6 | 5 | 0.036% | 7 | 6 | 6 | 5 | 5 | 0.031% | -1.06 | -0.06 | 2.4E-01 | Red |
| WP_041938733.1 | FRAAL0604 | aspartate-semialdehyde dehydrogenase | 37583 | 4.97 | 9 | 60 | Frankia-1 | F201364.dat | 247 | 21 | 7 | 7 | 7 | 7 | 5 | 5 | 7 | 7 | 0.029% | 5 | 6 | 7 | 5 | 6 | 0.026% | -1.06 | -0.06 | 2.7E-01 | Red |
| WP_011601299.1 | FRAAL0031 | membrane protein | 31879 | 9.92 | 3 | 25 | Frankia-8 | F201371.dat | 138 | 17 | 3 | 3 | 3 | 3 | 3 | 2 | 2 | 3 | 0.015% | 2 | 2 | 3 | 3 | 2 | 0.013% | -1.06 | -0.06 | 3.4E-01 | Red |
| WP_011602437.1 | FRAAL1236 | glycosyl transferase family 1 | 58803 | 7.78 | 19 | 163 | Frankia-2 | F201365.dat | 572 | 29 | 14 | 14 | 20 | 16 | 20 | 16 | 15 | 17 | 0.051% | 14 | 15 | 18 | 17 | 15 | 0.045% | -1.06 | -0.06 | 2.0E-01 | Red |
| WP_011605694.1 | FRAAL4578 | 6-phosphogluconolactonase | 27440 | 5.27 | 16 | 161 | Frankia-4 | F201367.dat | 771 | 67 | 14 | 14 | 20 | 16 | 16 | 17 | 20 | 14 | 0.108% | 14 | 13 | 19 | 15 | 17 | 0.096% | -1.06 | -0.06 | 2.6E-01 | Red |
| WP_011603027.1 | FRAAL1849 | mechanosensitive ion channel protein MscS | 45415 | 4.88 | 7 | 58 | Frankia-1 | F201364.dat | 323 | 21 | 7 | 7 | 7 | 7 | 6 | 6 | 6 | 5 | 0.024% | 6 | 7 | 4 | 6 | 5 | 0.021% | -1.06 | -0.06 | 2.6E-01 | Red |
| WP_041939649.1 | FRAAL5152 | dihydrolipoamide succinyltransferase | 49742 | 4.91 | 33 | 1043 | Frankia-5 | F201368.dat | 2276 | 63 | 31 | 31 | 113 | 106 | 109 | 106 | 103 | 113 | 0.385% | 94 | 116 | 97 | 102 | 97 | 0.343% | -1.06 | -0.06 | 9.2E-02 | Red |
| WP_011603370.1 | FRAAL2205 | cell division protein DivIVA | 35094 | 5.30 | 19 | 223 | Frankia-9 | F201372.dat | 792 | 48 | 16 | 16 | 23 | 22 | 22 | 20 | 25 | 26 | 0.117% | 21 | 20 | 21 | 23 | 23 | 0.104% | -1.06 | -0.06 | 1.5E-01 | Red |
| WP_011607298.1 | FRAAL6249 | LPS biosynthesis protein | 54061 | 7.84 | 30 | 384 | Frankia-5 | F201368.dat | 1630 | 60 | 27 | 27 | 42 | 42 | 34 | 39 | 41 | 42 | 0.131% | 32 | 37 | 39 | 41 | 37 | 0.116% | -1.06 | -0.06 | 1.5E-01 | Red |
| WP_011603123.1 | FRAAL1950 | O-succinylbenzoate--CoA ligase | 52824 | 4.79 | 18 | 153 | Frankia-8 | F201371.dat | 616 | 33 | 13 | 13 | 17 | 17 | 15 | 15 | 14 | 18 | 0.053% | 13 | 15 | 17 | 14 | 15 | 0.047% | -1.06 | -0.06 | 1.7E-01 | Red |
| WP_011606295.1 | FRAAL5201 | argininosuccinate lyase | 53617 | 5.20 | 15 | 120 | Frankia-4 | F201367.dat | 642 | 34 | 12 | 12 | 13 | 12 | 12 | 14 | 13 | 11 | 0.041% | 9 | 11 | 13 | 10 | 15 | 0.036% | -1.06 | -0.06 | 2.6E-01 | Red |
| WP_011601636.1 | FRAAL0379 | acyl-CoA dehydrogenase | 43356 | 4.71 | 16 | 151 | Frankia-4 | F201367.dat | 752 | 38 | 14 | 14 | 18 | 17 | 13 | 16 | 18 | 14 | 0.064% | 13 | 15 | 17 | 13 | 15 | 0.057% | -1.06 | -0.06 | 2.1E-01 | Red |
| WP_041938852.1 | FRAAL1116 | phosphoglucosamine mutase | 46473 | 5.08 | 9 | 54 | Frankia-2 | F201365.dat | 308 | 22 | 6 | 6 | 8 | 5 | 8 | 5 | 6 | 4 | 0.022% | 5 | 6 | 4 | 4 | 7 | 0.019% | -1.06 | -0.06 | 3.3E-01 | Red |
| WP_041938861.1 | FRAAL1154 | acetyl-CoA carboxylase | 52551 | 9.11 | 9 | 54 | Frankia-1 | F201364.dat | 272 | 20 | 7 | 7 | 7 | 7 | 4 | 6 | 7 | 4 | 0.019% | 3 | 5 | 6 | 6 | 6 | 0.017% | -1.06 | -0.06 | 3.3E-01 | Red |
| WP_011607701.1 | FRAAL6663 | phosphoribosylamine--glycine ligase | 44907 | 5.28 | 12 | 54 | Frankia-8 | F201371.dat | 441 | 30 | 9 | 9 | 10 | 6 | 5 | 6 | 6 | 5 | 0.022% | 3 | 5 | 10 | 4 | 4 | 0.019% | -1.06 | -0.06 | 3.8E-01 | Red |
| WP_041939337.1 | FRAAL3390 | NADP oxidoreductase | 50519 | 5.38 | 16 | 116 | Frankia-8 | F201371.dat | 697 | 32 | 13 | 13 | 15 | 14 | 10 | 12 | 12 | 12 | 0.042% | 9 | 11 | 15 | 10 | 11 | 0.037% | -1.07 | -0.06 | 2.6E-01 | Red |
| WP_041938653.1 | FRAAL0279 | methyltransferase type 12 | 51312 | 6.15 | 20 | 147 | Frankia-3 | F201366.dat | 668 | 44 | 14 | 14 | 17 | 16 | 15 | 17 | 14 | 14 | 0.053% | 14 | 15 | 17 | 12 | 13 | 0.047% | -1.07 | -0.06 | 1.8E-01 | Red |
| WP_041938631.1 | FRAAL0172 | B12-dependent methionine synthase | 131950 | 4.95 | 23 | 147 | Frankia-1 | F201364.dat | 776 | 18 | 18 | 18 | 19 | 19 | 14 | 17 | 16 | 10 | 0.021% | 16 | 11 | 18 | 14 | 12 | 0.018% | -1.07 | -0.06 | 3.1E-01 | Red |
| WP_041939950.1 | FRAAL6686 | serine/threonine protein phosphatase | 46865 | 4.74 | 10 | 83 | Frankia-6 | F201369.dat | 437 | 31 | 8 | 8 | 9 | 9 | 7 | 7 | 12 | 8 | 0.033% | 9 | 9 | 9 | 7 | 6 | 0.029% | -1.07 | -0.06 | 3.0E-01 | Red |
| WP_011607593.1 | FRAAL6553 | NUDIX hydrolase | 30732 | 4.81 | 7 | 52 | Frankia-4 | F201367.dat | 311 | 18 | 6 | 6 | 6 | 8 | 5 | 4 | 6 | 4 | 0.031% | 5 | 5 | 6 | 3 | 6 | 0.027% | -1.07 | -0.06 | 3.4E-01 | Red |
| WP_041938834.1 | FRAAL1006 | hypothetical protein | 53596 | 5.99 | 5 | 21 | Frankia-6 | F201369.dat | 150 | 9 | 3 | 3 | 3 | 2 | 2 | 2 | 3 | 2 | 0.007% | 3 | 2 | 2 | 1 | 2 | 0.006% | -1.07 | -0.06 | 3.4E-01 | Red |
| WP_011607357.1 | FRAAL6309 | oxidoreductase | 32812 | 5.38 | 4 | 21 | Frankia-5 | F201368.dat | 153 | 13 | 3 | 3 | 3 | 2 | 2 | 2 | 2 | 3 | 0.012% | 2 | 2 | 3 | 1 | 2 | 0.010% | -1.07 | -0.06 | 3.4E-01 | Red |
| WP_011605104.1 | FRAAL3972 | NADP-dependent aryl-alcohol dehydrogenase | 35694 | 5.81 | 5 | 21 | Frankia-8 | F201371.dat | 126 | 11 | 3 | 3 | 3 | 2 | 2 | 1 | 3 | 3 | 0.011% | 1 | 1 | 3 | 3 | 2 | 0.009% | -1.07 | -0.06 | 3.7E-01 | Red |
| WP_011606341.1 | FRAAL5250 | aminodeoxychorismate lyase | 53624 | 4.84 | 6 | 21 | Frankia-8 | F201371.dat | 180 | 16 | 5 | 5 | 5 | 3 | 1 | 2 | 3 | 2 | 0.007% | 0 | 3 | 5 | 1 | 1 | 0.006% | -1.07 | -0.06 | 4.2E-01 | Red |
| WP_041941157.1 | FRAAL6844 | dihydroxy-acid dehydratase | 64564 | 5.24 | 29 | 321 | Frankia-8 | F201371.dat | 1210 | 51 | 24 | 24 | 33 | 36 | 30 | 31 | 34 | 35 | 0.092% | 27 | 30 | 33 | 32 | 33 | 0.081% | -1.07 | -0.07 | 1.1E-01 | Red |
| WP_041939597.1 | FRAAL4947 | molecular chaperone DnaK | 87931 | 5.08 | 15 | 79 | Frankia-1 | F201364.dat | 528 | 16 | 10 | 10 | 10 | 10 | 7 | 8 | 9 | 7 | 0.017% | 6 | 8 | 7 | 8 | 9 | 0.015% | -1.07 | -0.07 | 2.3E-01 | Red |
| WP_011602441.1 | FRAAL1240 | haloacid dehalogenase | 94066 | 5.54 | 39 | 433 | Frankia-1 | F201364.dat | 1702 | 43 | 31 | 31 | 51 | 51 | 43 | 46 | 44 | 40 | 0.085% | 42 | 40 | 43 | 42 | 42 | 0.075% | -1.07 | -0.07 | 7.6E-02 | Red |
| WP_011603022.1 | FRAAL1843 | ABC transporter ATP-binding protein | 61767 | 5.25 | 36 | 576 | Frankia-3 | F201366.dat | 1854 | 64 | 32 | 32 | 67 | 61 | 61 | 67 | 58 | 51 | 0.172% | 60 | 53 | 55 | 51 | 59 | 0.152% | -1.07 | -0.07 | 1.2E-01 | Red |
| WP_041939777.1 | FRAAL5893 | heat-shock protein Hsp70 | 58498 | 4.77 | 30 | 280 | Frankia-6 | F201369.dat | 1438 | 66 | 25 | 25 | 30 | 30 | 33 | 30 | 29 | 23 | 0.088% | 30 | 22 | 32 | 21 | 30 | 0.078% | -1.07 | -0.07 | 2.5E-01 | Red |
| WP_041939750.1 | FRAAL5698 | glycoside hydrolase | 50251 | 5.92 | 17 | 106 | Frankia-3 | F201366.dat | 619 | 32 | 12 | 12 | 14 | 8 | 12 | 14 | 10 | 11 | 0.039% | 7 | 10 | 12 | 10 | 12 | 0.034% | -1.07 | -0.07 | 2.9E-01 | Red |
| WP_041939480.1 | FRAAL4296 | hypothetical protein | 16687 | 9.13 | 2 | 19 | Frankia-5 | F201368.dat | 196 | 19 | 2 | 2 | 2 | 2 | 2 | 2 | 2 | 2 | 0.021% | 2 | 2 | 1 | 2 | 2 | 0.018% | -1.07 | -0.07 | 3.4E-01 | Red |
| WP_011603357.1 | FRAAL2192 | UDP-N-acetylmuramoyl-tripeptide--D-alanyl-D-alanine ligase | 46620 | 5.26 | 11 | 48 | Frankia-7 | F201370.dat | 366 | 23 | 6 | 6 | 7 | 5 | 6 | 4 | 6 | 4 | 0.019% | 5 | 7 | 6 | 2 | 3 | 0.017% | -1.07 | -0.07 | 3.5E-01 | Red |
| WP_011606289.1 | FRAAL5187 | haloacid dehalogenase | 39161 | 5.16 | 4 | 19 | Frankia-8 | F201371.dat | 145 | 7 | 3 | 3 | 4 | 2 | 2 | 2 | 1 | 3 | 0.009% | 1 | 1 | 4 | 1 | 2 | 0.008% | -1.07 | -0.07 | 3.9E-01 | Red |
| WP_011607569.1 | FRAAL6529 | dna integrity scanning protein disa | 38861 | 5.35 | 14 | 104 | Frankia-4 | F201367.dat | 608 | 33 | 9 | 9 | 12 | 9 | 9 | 13 | 12 | 11 | 0.050% | 8 | 13 | 10 | 9 | 10 | 0.043% | -1.07 | -0.07 | 2.5E-01 | Red |
| WP_041939008.1 | FRAAL1862 | ribose 5-phosphate isomerase | 17026 | 5.69 | 7 | 75 | Frankia-8 | F201371.dat | 332 | 54 | 6 | 6 | 11 | 8 | 7 | 7 | 8 | 9 | 0.082% | 5 | 5 | 11 | 8 | 7 | 0.071% | -1.07 | -0.07 | 3.1E-01 | Red |
| WP_041938877.1 | FRAAL1212 | methylmalonyl-CoA carboxyltransferase | 57369 | 5.04 | 25 | 521 | Frankia-5 | F201368.dat | 1497 | 50 | 23 | 23 | 57 | 58 | 48 | 51 | 56 | 57 | 0.168% | 41 | 49 | 56 | 45 | 60 | 0.147% | -1.07 | -0.07 | 1.8E-01 | Red |
| WP_011607660.1 | FRAAL6623 | F420-dependent oxidoreductase | 31256 | 5.28 | 10 | 73 | Frankia-8 | F201371.dat | 385 | 32 | 8 | 8 | 9 | 8 | 8 | 8 | 7 | 7 | 0.043% | 6 | 6 | 9 | 8 | 6 | 0.038% | -1.08 | -0.07 | 2.1E-01 | Red |
| WP_041938832.1 | FRAAL0997 | glutamate-1-semialdehyde aminotransferase | 46090 | 5.07 | 11 | 156 | Frankia-1 | F201364.dat | 750 | 33 | 10 | 10 | 20 | 20 | 17 | 17 | 15 | 12 | 0.063% | 13 | 15 | 17 | 15 | 15 | 0.055% | -1.08 | -0.07 | 2.2E-01 | Red |
| WP_011605676.1 | FRAAL4559 | cysteine desulfurase | 47566 | 5.59 | 18 | 152 | Frankia-4 | F201367.dat | 692 | 50 | 14 | 14 | 17 | 16 | 14 | 17 | 17 | 15 | 0.059% | 12 | 15 | 17 | 17 | 12 | 0.052% | -1.08 | -0.07 | 1.9E-01 | Red |
| WP_011606113.1 | FRAAL5001 | imidazole glycerol phosphate synthase subunit HisH | 21488 | 5.83 | 5 | 17 | Frankia-4 | F201367.dat | 97 | 16 | 2 | 2 | 2 | 2 | 1 | 2 | 2 | 2 | 0.015% | 1 | 2 | 2 | 2 | 1 | 0.013% | -1.08 | -0.07 | 3.4E-01 | Red |
| WP_011604764.1 | FRAAL3625 | ribose-phosphate pyrophosphokinase | 33973 | 6.46 | 4 | 17 | Frankia-3 | F201366.dat | 149 | 13 | 3 | 3 | 3 | 2 | 2 | 3 | 1 | 1 | 0.009% | 2 | 2 | 1 | 2 | 1 | 0.008% | -1.08 | -0.07 | 3.5E-01 | Red |
| WP_011607316.1 | FRAAL6266 | peptidyl-tRNA hydrolase | 21357 | 9.50 | 4 | 17 | Frankia-5 | F201368.dat | 117 | 12 | 3 | 3 | 3 | 1 | 2 | 1 | 2 | 3 | 0.015% | 1 | 1 | 3 | 1 | 2 | 0.013% | -1.08 | -0.07 | 3.6E-01 | Red |
| WP_041939833.1 | FRAAL6149 | exodeoxyribonuclease VII large subunit | 43089 | 10.85 | 4 | 17 | Frankia-8 | F201371.dat | 133 | 12 | 3 | 3 | 3 | 2 | 0 | 2 | 2 | 3 | 0.007% | 1 | 2 | 3 | 2 | 0 | 0.006% | -1.08 | -0.07 | 3.9E-01 | Red |
| WP_011606253.1 | FRAAL5148 | aminomethyltransferase | 38040 | 5.91 | 15 | 150 | Frankia-5 | F201368.dat | 817 | 50 | 13 | 13 | 18 | 11 | 18 | 16 | 15 | 18 | 0.073% | 12 | 14 | 19 | 13 | 14 | 0.064% | -1.08 | -0.08 | 2.6E-01 | Red |
| WP_011606819.1 | FRAAL5745 | UDP-glucose 6-dehydrogenase | 53354 | 5.05 | 18 | 96 | Frankia-8 | F201371.dat | 454 | 26 | 10 | 10 | 11 | 11 | 9 | 11 | 8 | 11 | 0.033% | 7 | 9 | 11 | 8 | 11 | 0.029% | -1.08 | -0.08 | 2.3E-01 | Red |
| WP_041940000.1 | FRAAL6867 | serine/threonine protein kinase | 68870 | 4.91 | 26 | 252 | Frankia-4 | F201367.dat | 1131 | 47 | 24 | 24 | 29 | 27 | 24 | 28 | 29 | 23 | 0.068% | 21 | 26 | 29 | 23 | 22 | 0.059% | -1.08 | -0.08 | 1.6E-01 | Red |
| WP_041939864.1 | FRAAL6287 | branched-chain amino acid-binding protein | 39042 | 9.87 | 9 | 42 | Frankia-3 | F201366.dat | 335 | 20 | 6 | 6 | 6 | 5 | 5 | 6 | 2 | 4 | 0.020% | 5 | 3 | 5 | 3 | 4 | 0.017% | -1.08 | -0.08 | 3.2E-01 | Red |
| WP_011607331.1 | FRAAL6283 | ribose-phosphate pyrophosphokinase | 35132 | 5.75 | 11 | 119 | Frankia-7 | F201370.dat | 394 | 27 | 9 | 9 | 14 | 12 | 12 | 11 | 13 | 14 | 0.063% | 9 | 14 | 12 | 10 | 12 | 0.055% | -1.08 | -0.08 | 1.8E-01 | Red |
| WP_011602130.1 | FRAAL0899 | hypothetical protein | 23660 | 5.50 | 8 | 144 | Frankia-5 | F201368.dat | 334 | 23 | 6 | 6 | 14 | 14 | 16 | 14 | 17 | 14 | 0.113% | 13 | 17 | 12 | 13 | 14 | 0.098% | -1.08 | -0.08 | 1.5E-01 | Red |
| WP_011601886.1 | FRAAL0637 | Mur ligase | 44618 | 8.99 | 10 | 67 | Frankia-5 | F201368.dat | 290 | 20 | 8 | 8 | 9 | 6 | 7 | 7 | 6 | 9 | 0.028% | 8 | 7 | 8 | 5 | 4 | 0.024% | -1.08 | -0.08 | 2.8E-01 | Red |
| WP_050996955.1 | FRAAL0152 | S-adenosyl methyltransferase | 28521 | 4.63 | 8 | 65 | Frankia-7 | F201370.dat | 349 | 27 | 6 | 6 | 7 | 8 | 7 | 6 | 8 | 5 | 0.043% | 6 | 7 | 6 | 6 | 6 | 0.037% | -1.08 | -0.08 | 2.0E-01 | Red |
| WP_011601505.1 | FRAAL0247 | hypothetical protein | 45686 | 6.78 | 7 | 40 | Frankia-8 | F201371.dat | 173 | 12 | 5 | 5 | 5 | 5 | 4 | 4 | 4 | 4 | 0.016% | 3 | 5 | 5 | 3 | 3 | 0.014% | -1.08 | -0.08 | 2.6E-01 | Red |
| WP_041940195.1 | FRAAL1254 | glycosyl transferase | 32518 | 9.98 | 10 | 90 | Frankia-8 | F201371.dat | 363 | 25 | 8 | 8 | 12 | 8 | 9 | 10 | 11 | 9 | 0.052% | 7 | 6 | 12 | 11 | 7 | 0.045% | -1.08 | -0.08 | 2.8E-01 | Red |
| WP_011607816.1 | FRAAL6782 | hypothetical protein | 19666 | 8.37 | 2 | 15 | Frankia-10 | F201373.dat | 61 | 8 | 2 | 2 | 2 | 1 | 2 | 1 | 2 | 2 | 0.015% | 1 | 1 | 1 | 2 | 2 | 0.012% | -1.08 | -0.08 | 3.4E-01 | Red |
| WP_011605406.1 | FRAAL4280 | pyruvate dehydrogenase | 39826 | 5.03 | 3 | 15 | Frankia-5 | F201368.dat | 140 | 8 | 3 | 3 | 3 | 2 | 1 | 1 | 1 | 3 | 0.007% | 0 | 1 | 2 | 2 | 2 | 0.006% | -1.08 | -0.08 | 3.7E-01 | Red |
| WP_041940362.1 | FRAAL2330 | cobalamin biosynthesis protein CobN | 131090 | 5.13 | 5 | 15 | Frankia-8 | F201371.dat | 144 | 4 | 4 | 4 | 4 | 2 | 2 | 2 | 1 | 1 | 0.002% | 1 | 1 | 4 | 1 | 0 | 0.002% | -1.08 | -0.08 | 4.0E-01 | Red |
| WP_011607137.1 | FRAAL6085 | sodium:proton antiporter | 40158 | 6.09 | 17 | 213 | Frankia-1 | F201364.dat | 736 | 44 | 14 | 14 | 25 | 25 | 22 | 19 | 20 | 25 | 0.099% | 21 | 19 | 19 | 22 | 21 | 0.086% | -1.08 | -0.08 | 1.1E-01 | Red |
| WP_011604005.1 | FRAAL2859 | hypothetical protein | 49474 | 5.18 | 14 | 63 | Frankia-1 | F201364.dat | 302 | 20 | 9 | 9 | 9 | 9 | 5 | 8 | 7 | 4 | 0.024% | 3 | 8 | 6 | 8 | 5 | 0.020% | -1.09 | -0.08 | 3.3E-01 | Red |
| WP_011607908.1 | FRAAL6873 | thioredoxin | 11727 | 4.91 | 7 | 86 | Frankia-2 | F201365.dat | 308 | 64 | 7 | 7 | 10 | 8 | 10 | 9 | 9 | 9 | 0.137% | 9 | 8 | 7 | 9 | 8 | 0.118% | -1.09 | -0.08 | 7.4E-02 | Red |
| WP_011607332.1 | FRAAL6284 | tryptophanyl-tRNA synthetase | 37220 | 5.66 | 9 | 86 | Frankia-7 | F201370.dat | 418 | 32 | 8 | 8 | 9 | 10 | 8 | 9 | 10 | 8 | 0.043% | 7 | 9 | 9 | 8 | 8 | 0.037% | -1.09 | -0.08 | 1.0E-01 | Red |
| WP_041938855.1 | FRAAL1126 | alpha/beta hydrolase | 41525 | 5.62 | 5 | 38 | Frankia-5 | F201368.dat | 233 | 14 | 4 | 4 | 5 | 3 | 3 | 4 | 5 | 5 | 0.017% | 4 | 3 | 4 | 2 | 5 | 0.015% | -1.09 | -0.08 | 2.9E-01 | Red |
| WP_011603030.1 | FRAAL1852 | 4-alpha-glucanotransferase | 82853 | 5.16 | 17 | 86 | Frankia-8 | F201371.dat | 554 | 14 | 10 | 10 | 10 | 10 | 9 | 8 | 9 | 9 | 0.019% | 6 | 4 | 10 | 9 | 12 | 0.017% | -1.09 | -0.08 | 3.0E-01 | Red |
| WP_041938835.1 | FRAAL1010 | radical SAM protein | 43222 | 5.99 | 21 | 180 | Frankia-3 | F201366.dat | 733 | 47 | 14 | 14 | 19 | 22 | 14 | 19 | 18 | 21 | 0.078% | 15 | 16 | 24 | 16 | 15 | 0.067% | -1.09 | -0.08 | 2.4E-01 | Red |
| WP_050997433.1 | FRAAL5235 | carbamoyl phosphate synthase small subunit | 40889 | 4.92 | 7 | 61 | Frankia-5 | F201368.dat | 343 | 30 | 7 | 7 | 8 | 7 | 6 | 5 | 6 | 8 | 0.028% | 7 | 7 | 5 | 5 | 5 | 0.024% | -1.09 | -0.08 | 2.1E-01 | Red |
| WP_011606826.1 | FRAAL5752 | zinc protease | 49190 | 4.92 | 11 | 61 | Frankia-1 | F201364.dat | 452 | 26 | 8 | 8 | 8 | 8 | 3 | 6 | 8 | 7 | 0.023% | 4 | 7 | 4 | 6 | 8 | 0.020% | -1.09 | -0.08 | 3.2E-01 | Red |
| WP_011605492.1 | FRAAL4370 | acetyltransferase | 32247 | 7.03 | 13 | 155 | Frankia-8 | F201371.dat | 690 | 49 | 11 | 11 | 19 | 15 | 13 | 18 | 17 | 18 | 0.090% | 14 | 14 | 19 | 14 | 13 | 0.077% | -1.09 | -0.08 | 1.8E-01 | Red |
| WP_011604542.1 | FRAAL3395 | 3-(3-hydroxyphenyl)propionate hydroxylase | 61795 | 7.17 | 20 | 84 | Frankia-8 | F201371.dat | 584 | 34 | 14 | 14 | 14 | 9 | 8 | 7 | 10 | 10 | 0.025% | 7 | 7 | 14 | 7 | 5 | 0.022% | -1.09 | -0.09 | 3.2E-01 | Red |
| WP_011607785.1 | FRAAL6749 | hypothetical protein | 50613 | 9.37 | 15 | 107 | Frankia-1 | F201364.dat | 549 | 37 | 12 | 12 | 12 | 12 | 12 | 12 | 9 | 11 | 0.039% | 11 | 10 | 9 | 11 | 10 | 0.034% | -1.09 | -0.09 | 9.3E-02 | Red |
| WP_041941070.1 | FRAAL6296 | hypothetical protein | 52870 | 4.56 | 14 | 130 | Frankia-3 | F201366.dat | 492 | 27 | 11 | 11 | 13 | 15 | 12 | 13 | 14 | 14 | 0.046% | 9 | 14 | 13 | 12 | 14 | 0.039% | -1.09 | -0.09 | 1.4E-01 | Red |
| WP_011601774.1 | FRAAL0521 | membrane protein | 40966 | 5.81 | 21 | 174 | Frankia-9 | F201372.dat | 885 | 45 | 14 | 14 | 19 | 17 | 19 | 17 | 19 | 19 | 0.079% | 12 | 18 | 16 | 19 | 18 | 0.068% | -1.09 | -0.09 | 1.3E-01 | Red |
| WP_041939687.1 | FRAAL5375 | oxidoreductase | 30823 | 5.97 | 7 | 59 | Frankia-8 | F201371.dat | 332 | 27 | 7 | 7 | 7 | 6 | 6 | 6 | 6 | 7 | 0.036% | 4 | 7 | 7 | 5 | 5 | 0.031% | -1.09 | -0.09 | 2.0E-01 | Red |
| WP_011605101.1 | FRAAL3969 | F420-dependent oxidoreductase | 37053 | 5.56 | 7 | 36 | Frankia-5 | F201368.dat | 204 | 15 | 4 | 4 | 4 | 4 | 4 | 3 | 4 | 4 | 0.018% | 3 | 4 | 3 | 4 | 3 | 0.015% | -1.09 | -0.09 | 2.1E-01 | Red |
| WP_041939806.1 | FRAAL6017 | histidinol phosphatase | 29818 | 4.76 | 4 | 36 | Frankia-8 | F201371.dat | 299 | 25 | 4 | 4 | 4 | 4 | 4 | 3 | 4 | 4 | 0.023% | 4 | 3 | 4 | 3 | 3 | 0.019% | -1.09 | -0.09 | 2.1E-01 | Red |
| WP_011601628.1 | FRAAL0371 | hypothetical protein | 69881 | 5.16 | 18 | 105 | Frankia-1 | F201364.dat | 656 | 29 | 14 | 14 | 15 | 15 | 7 | 10 | 10 | 13 | 0.028% | 8 | 8 | 12 | 11 | 11 | 0.024% | -1.09 | -0.09 | 2.8E-01 | Red |
| WP_050996969.1 | FRAAL0319 | hypothetical protein | 52045 | 9.00 | 10 | 82 | Frankia-5 | F201368.dat | 410 | 22 | 10 | 10 | 10 | 9 | 7 | 9 | 8 | 10 | 0.029% | 10 | 10 | 6 | 9 | 4 | 0.025% | -1.09 | -0.09 | 2.8E-01 | Red |
| WP_011606109.1 | FRAAL4996 | ATP/GTP-binding protein | 97744 | 4.99 | 3 | 13 | Frankia-8 | F201371.dat | 90 | 3 | 2 | 2 | 2 | 1 | 1 | 2 | 1 | 2 | 0.003% | 1 | 1 | 2 | 1 | 1 | 0.002% | -1.09 | -0.09 | 3.4E-01 | Red |
| WP_011603323.1 | FRAAL2158 | hypothetical protein | 36162 | 11.20 | 2 | 13 | Frankia-3 | F201366.dat | 131 | 5 | 2 | 2 | 2 | 1 | 1 | 2 | 1 | 2 | 0.007% | 1 | 2 | 1 | 1 | 1 | 0.006% | -1.09 | -0.09 | 3.4E-01 | Red |
| WP_011605124.1 | FRAAL3993 | hypothetical protein | 35319 | 6.81 | 2 | 13 | Frankia-5 | F201368.dat | 108 | 9 | 2 | 2 | 2 | 1 | 1 | 2 | 1 | 2 | 0.007% | 1 | 1 | 1 | 2 | 1 | 0.006% | -1.09 | -0.09 | 3.4E-01 | Red |
| WP_041939999.1 | FRAAL6862 | HDIG domain-containing protein | 54426 | 6.15 | 8 | 36 | Frankia-3 | F201366.dat | 274 | 16 | 7 | 7 | 7 | 3 | 4 | 7 | 3 | 2 | 0.012% | 2 | 3 | 5 | 4 | 3 | 0.011% | -1.09 | -0.09 | 3.5E-01 | Red |
| WP_011604700.1 | FRAAL3559 | acyl-CoA dehydrogenase | 38539 | 5.32 | 3 | 13 | Frankia-3 | F201366.dat | 144 | 8 | 2 | 2 | 2 | 2 | 1 | 2 | 1 | 1 | 0.006% | 1 | 1 | 3 | 0 | 1 | 0.005% | -1.09 | -0.09 | 3.7E-01 | Red |
[truncated: 80,069 more chars]
